# Mesoionic Carbenes in Low- to High-Valent Vanadium Chemistry

**DOI:** 10.1021/acs.inorgchem.1c02087

**Published:** 2021-09-30

**Authors:** Florian R. Neururer, Shenyu Liu, Daniel Leitner, Marc Baltrun, Katherine R. Fisher, Holger Kopacka, Klaus Wurst, Lena J. Daumann, Dominik Munz, Stephan Hohloch

**Affiliations:** † Institute of Inorganic, General and Theoretical Chemistry, University of Innsbruck, Innrain 80-82, 6020 Innsbruck, Austria; ‡ Faculty of Science, Department of Chemistry, University of Paderborn, Warburger Straße 100, 33098 Paderborn, Germany; § Department Chemie, Ludwigs-Maximilians-University Munich, Butenandtstraße 5-13 Haus D, 81377 Munich, Germany; ∥ Fakultät NT, Inorganic Chemistry: Coordination Chemistry, Saarland University, Campus C4.1, 66123 Saarbrücken, Germany

## Abstract

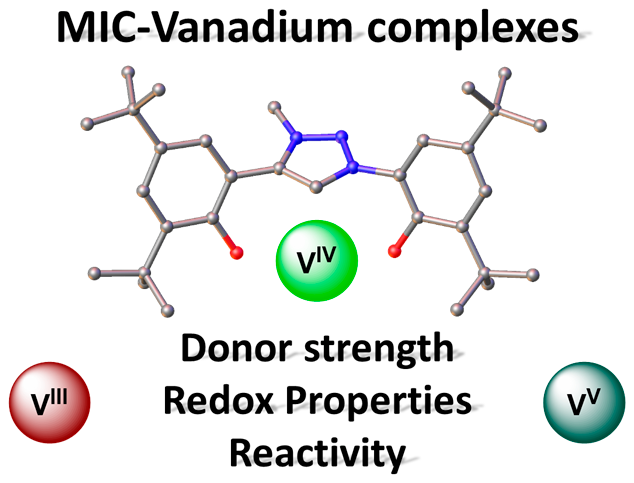

We report the synthesis of vanadium(V) oxo complex **1** with a pincer-type dianionic mesoionic carbene (MIC) ligand **L**^**1**^ and the general formula [VOCl(L^1^)]. A comparison of the structural (SC-XRD), electronic (UV–vis), and electrochemical (cyclic voltammetry) properties of **1** with the benzimidazolinylidene congener **2** (general formula [VOCl(L^2^)]) shows that the MIC is a stronger donor also for early transition metals with low d-electron population. Since electrochemical studies revealed both complexes to be reversibly reduced, the stronger donor character of MICs was not only demonstrated for the vanadium(V) but also for the vanadium(IV) oxidation state by isolating the reduced vanadium(IV) complexes [Co(Cp*)_**2**_**][1]** and [Co(Cp*)_**2**_**][2]** ([Co(Cp*)_2_] = decamethylcobaltocenium). The electronic structures of the compounds were investigated by computational methods. Complex **1** was found to be a moderate precursor for salt metathesis reactions, showing selective reactivity toward phenolates or secondary amides, but not toward primary amides and phosphides, thiophenols, or aryls/alkyls donors. Deoxygenation with electron-rich phosphines failed to give the desired vanadium(III) complex. However, treatment of the deprotonated ligand precursor with vanadium(III) trichloride resulted in the clean formation of the corresponding MIC vanadium(III) complex **6**, which undergoes a clean two-electron oxidation with organic azides yielding the corresponding imido complexes. The reaction with TMS-N_3_ did not afford a nitrido complex, but instead the imido complex **10**. This study reveals that, contrary to popular belief, MICs are capable of supporting early transition-metal complexes in a variety of oxidation states, thus making them promising candidates for the activation of small molecules and redox catalysis.

## Abstract 2

Dianionic mesoionic carbenes derived from 1,2,3-triazoles are introduced into low to high-valent vanadium chemistry. Their donor properties are compared to classical NHCs of the benzimidazolinylidene type, and the reactivity of the vanadium complexes in various oxidation states ranging from vanadium(III) to vanadium(V) is investigated.

## Introduction

Almost two decades after the first report of an abnormal 5-imidazolinylidene carbene complex,^1^ mesoionic carbenes have been developed into a distinguished ligand class.^2,3^ Among them, 1,2,3-triazole derived mesoionic carbenes, namely 1,2,3-triazolinylidenes,^4^ stand out by their modular synthesis via the copper-catalyzed [3 + 2] cycloaddition between azides and alkynes.^5−7^ After their initial reporting by Albrecht *et al.*,^8^ they quickly became prominent synthetic targets for (electro-)catalysis,^9−23^ supramolecular chemistry,^24−27^ magnetism,^28^ and photochemistry due to their versatile synthesis and comparatively straightforward handling.^29−36^ Throughout these studies, a great effort has been made to decipher their electronic structure. Mesoionic carbenes are commonly believed to be strong σ-donor ligands paralleling heteroaryls; however, recent reports emphasize their π-accepting properties^37−39^ as demonstrated by the isolation of a reduced triazolinylidene ligand.^40^ Nevertheless, most studies targeted hitherto late transition metals or main group elements, while early transition-metal complexes with mesoionic carbenes have been rarely explored.^41−44^ Arguably, this can be attributed to the relatively weak bond between *N*-heterocyclic carbenes and early transition metals.^45^ However, this weak bond may be enhanced by harnessing anionic linkers. This strategy has allowed the isolation of a number of interesting metal complexes^46−49^ of the early transition metals,^50−71^ the lanthanides,^72−80^ and the actinides.^81−86^

Among the early transition metals, vanadium chemistry has witnessed a remarkable activity over the past 50 years and has been applied in heterogeneous and homogeneous catalysis,^87^ small molecule activation,^88−92^ molecular magnetism,^93−96^ and spin qubits.^97−100^ Narrowing down the field to NHC vanadium complexes, since the first two reports of vanadium NHC complexes in 1994 by Roesky *et al.*(101) and 2003 by Abernethy *et al.*,^102^ the utility of these complexes has mostly been explored in polymerization catalysis.^103−107^ Beyond this, only a few other applications of vanadium-NHC complexes have been examined, including small molecule activation^108,109^ and the neutralization of chemical warfare agents.^110^ Still, most vanadium NHC complexes refer to diamagnetic vanadium(V) complexes, while low-valent vanadium complexes have been rarely investigated.^101,106,111−114^

Inspired by Bellemin-Laponnaz’s ligand design, employing two anionic redox-active phenolate linkers,^50−54,115−117^ we have recently reported the first mesoionic carbene complexes of groups IV, V, and VI based on a 1,2,3-triazolinylidene scaffold.^44^ Our initial report focused on niobium as a group V representative, and we thus expand this chemistry herein toward vanadium. We report the ligand’s σ-donor strength by comparing the structural, spectroscopic, and electrochemical properties of the triazolinylidene complex **1** with its benzimidazolinylidene (benzNHC) congener **2**, proving the triazolinylidene ligand **L**^**1**^ as the stronger donor. Furthermore, the salt metathesis reactivity of the new triazolinylidene complex **1** is presented, revealing moderate scope. While phenols and secondary amides give good and clean conversion, all other nucleophiles investigated gave no tractable reaction products. We furthermore report on the isolation of various vanadium complexes in the oxidation states +IV and +III, where the latter are potent precursors to vanadium(V) imido complexes.

## Results and Discussion

**Scheme 1:**
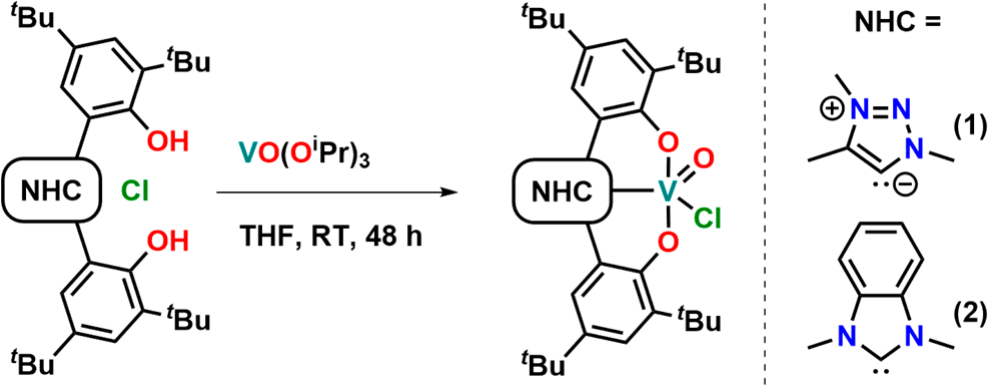
Synthesis of the Vanadium(V) NHC Complexes Following Protonolysis between the Corresponding Azolium Salts and VO(O^*i*^Pr)_3_

Despite our previous finding that protonolysis between the triazolium salt **[H**_**3**_**L**^**1**^**][Cl]** and Ti(O^*i*^Pr)_3_Cl did not lead to quantitative deprotonation of the triazolium salt,^44^ we decided to adopt this strategy using VO(O^*i*^Pr)_3_ as the vanadium source. To our delight, the reaction between the triazolium salt **[H**_**3**_**L**^**1**^**][Cl]** and VO(O^*i*^Pr)_3_, followed by the subsequent washing of the crude solids with hexane, afforded the MIC vanadium-oxo complex **1** as a dark green powder in yields of 85% (Scheme 1). The ^1^H NMR spectrum of **1** in benzene confirmed the desired transformation due to the absence of the O*H* and triazolium-*5H* protons which revealed a *C*_1_ symmetric species in solution. Unfortunately, due to the high quadrupolar moment of the ^51^V nucleus, we were not able to observe the characteristic ^13^C NMR resonance of the triazolinylidene carbon atom. Nevertheless, the absence of the triazolium-*5C* resonance at 131.9 ppm in the ^13^C NMR of **1** confirms the formation of a triazolinylidene complex of high-valent vanadium(V). Furthermore, a shift of the ^51^V resonance to −533 ppm (Figure S5) in the ^51^V NMR indicates the presence of a strong donor ligand. To set this value into context, we also synthesized the benzimidazolinylidene complex **2**, recently reported by LeRoux et al.^118,119^ The proton NMR of **2** shows a *C*_*s*_ symmetric species in solution where the absence of the O*H* and the benzimidazolium-2*H* protons indicates also the formation of an NHC complex of vanadium(V). Similar to **1**, we could not observe the carbene carbon resonance in the ^13^C NMR spectrum of **2**. The ^51^V NMR signal of **2** is (compared to complex **1**) strongly shifted to lower fields resonating at −503 ppm (Figure S10), indicating a lower donor strength of the benzimidazolinylidene compared to the triazolinylidene ligand.^120^ Unambiguous proof for the formation of the NHC and MIC complexes was obtained by single-crystal X-ray diffraction (SC-XRD) analysis. X-ray quality crystals of **1** and **2** could be grown by slow diffusion of pentane into a concentrated solution of the corresponding complexes in toluene or benzene, respectively (Figure 2). Complex **1** crystallized in the monoclinic space group *P*2_1_/*n* as a toluene solvate, while complex **2** crystallized without additional solvent molecules in the asymmetric unit in the orthorhombic space group *Pbca*. The most striking difference in the molecular conformations of the complexes is that in the case of **2**, the benzannulated heterocycle is shifted out of plane compared to the C1–V1 bond axis by 16.7(1)°, while for the triazolinylidene complex **1**, this pitch angle along the C1–V1 bond axis was found to be only 0.7(1)°. These structural parameters are well reproduced by density functional theory (DFT) calculations (Table S1) and are also discernible in the coordination environment around the vanadium center. While **1** adopts an almost perfect square pyramidal coordination environment in the solid state (τ_5_ = 0.05), complex **2** is distorted with τ_5_ = 0.20. The C1–V1 distances in **1** and **2** are 2.055(3) Å and 2.131(3) Å, suggesting a stronger metal carbene interaction in **1** compared to **2**. This agrees with the pronounced high-field shift of the ^51^V NMR resonances in **1** relative to **2**. The V1–O10 distances were determined to be 1.583(3) Å and 1.585(2) Å in **1** and **2**, showing only a minor influence of the NHC moiety toward the strength of the V=O bonds. However, the influence of the NHC unit on the V=O stretching frequencies in the IR is discernible with resonances at 986 cm^–1^ (calculated: 1026 cm^–1^) and 1000 cm^–1^ (calculated: 1035 cm^–1^) in **1** and **2**. These values are indicative for a weaker V=O multiple bond character in **1** in comparison to **2** due to arguably reduced π-donation from the terminal oxo ligand, and thus, corroborate stronger donor properties of the MIC ligand.

**Figure 1:**
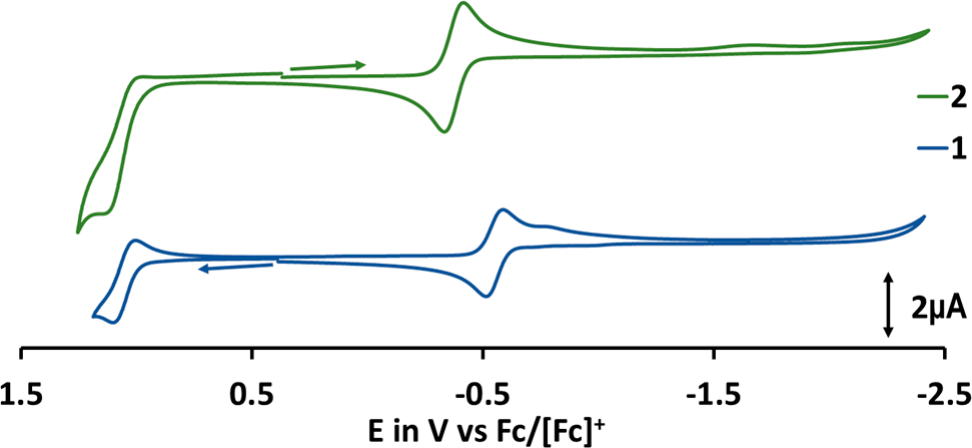
Cyclic voltammogram of **1** (blue) and **2** (green) in 0.1 M NBu_4_PF_6_ in CH_2_Cl_2_ at 298 K. Scan rate: 100 mV s^–1^.

To further probe the donor properties of the triazolin- versus the benzimidazolinylidene donor, we investigated the complexes by electrochemical methods. Cyclic voltammetry measured in dichloromethane revealed a reversible reduction corresponding to the V(V/IV) redox couple for both complexes (Figure 1). In agreement with the results from ^51^V NMR and IR spectroscopy, the reduction potential (the reductions are vanadium centered, *vide infra*) for **1** appears 0.19 V cathodically shifted compared to the reduction potential of **2** (Table 1). This suggests a higher electron density at the vanadium center in **1**, which is in line with the higher σ-donor character of MIC relative to benzNHC ligands. Additionally, complex **1** showed one reversible (ligand-centered) oxidation, whereas for **2** it is at the edge of the solvent window and thus could not be evaluated (Figures S69–S72).

**Table 1:** Oxidation and Reduction Potentials of the Vanadium(V)-NHC Complexes **1** and **2** Referenced vs Fc/[Fc]^+^ in 0.1 M NBu_4_PF_6_ Solutions in CH_2_Cl_2_ at 298 K

complex	*E*^1/2^ ox. (V)	Δ*E* (mV)	*E*^1/2^ red. (V)	Δ*E* (mV)
**1**	1.05	80	–0.56	70
**2**	n.a.	n.o.	–0.37	90

**Scheme 2:**
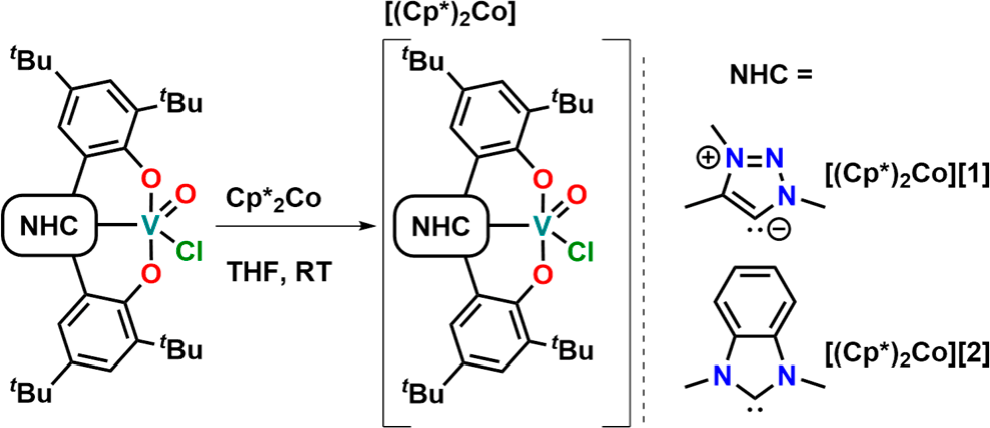
Reduction of Complexes **1** and **2** by Decamethylcobaltocene

**Figure 2:**
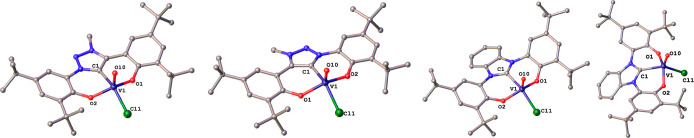
Molecular structures of **1**, [Co(Cp*)_**2**_**][1]**, **2**, and [Co(Cp*)_**2**_**][2]** (from left to right). Hydrogen atoms, solvent molecules, and counterions have been omitted for clarity.

To reveal the site of reduction for the two vanadium complexes, we reduced the complexes with decamethylcobaltocene (Scheme 2). While a THF solution turned greyish upon reduction of **1**, the desired product precipitated as a bright green powder for **2**. Evans method in CD_2_Cl_2_ revealed a magnetic moment of 1.74 and 1.67 μ_B_ for [Co(Cp*)_**2**_**][1]** and [Co(Cp*)_**2**_**][2]**, which is in agreement with a vanadium(IV) redox state.^121^ Accordingly, the V=O stretches shift from 986 to 932 cm^–1^ (calculated: 1026 to 1007 cm^–1^) and from 1000 to 968 cm^–1^ (calculated: 1035 to 1007 cm^–1^) in [Co(Cp*)_**2**_**][1]** and [Co(Cp*)_**2**_**][2]**, respectively (see Figures S49–S52). X-ray quality crystals of both reduced complexes[Co(Cp*)_**2**_**][1]** and [Co(Cp*)_**2**_**][2]** could be grown by the slow evaporation of dichloromethane out of a hexane/dichloromethane mixture (Figure 2).

**Figure 3:**
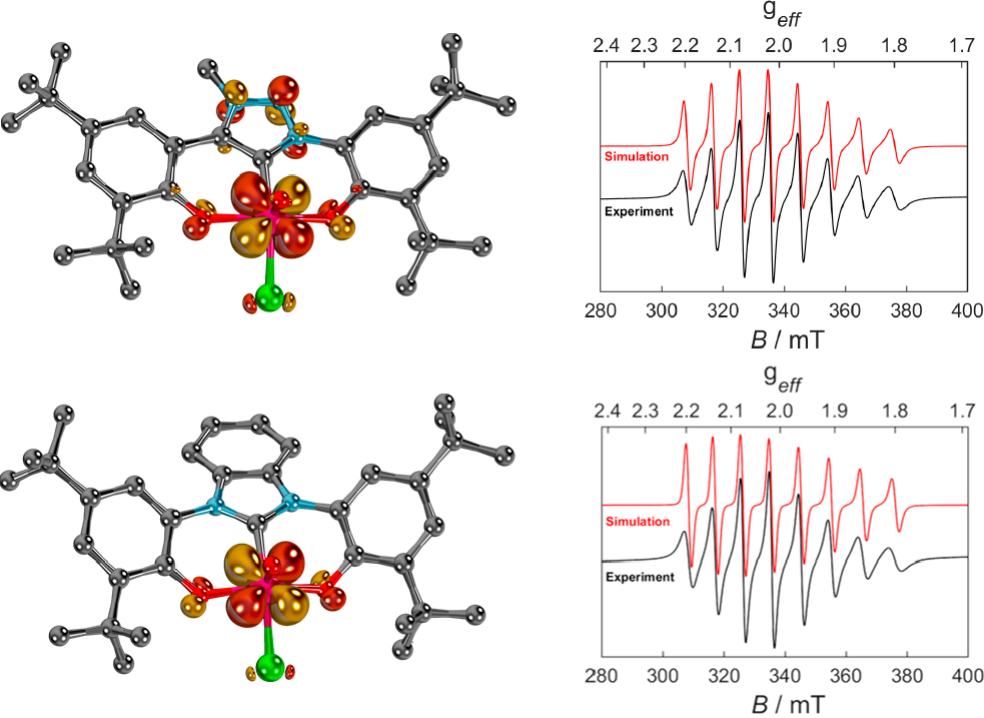
Compounds [Co(Cp*)_**2**_**][1]** (top left) and [Co(Cp*)_**2**_**][2]** (bottom left) feature unpaired electrons in the 3d(*yz*) orbitals (QROs). X-band EPR spectra of [Co(Cp*)_**2**_**][1]** (top right) and [Co(Cp*)_**2**_**][2]** (bottom right) of 5 mM solutions in CH_2_Cl_2_ at 300 K; black traces show the experimentally observed spectra and red traces the corresponding simulations. Hydrogen atoms have been omitted for clarity.

While the general structural factors (e.g., coordination environment) resemble the same trends as the parent vanadium(V) complexes **1** and **2**, the vanadium donor atom distances increase slightly. For example, the vanadium carbene distances expand from 2.055(3) Å to 2.070(3) Å and from 2.131(3) Å to 2.153(7) Å during the reduction to [Co(Cp*)_**2**_**][1]** and [Co(Cp*)_**2**_**][2]**, respectively. Similarly, the phenolate distances elongate by almost 0.1 Å (compare Table S3). This is in line with the larger ionic radius of a vanadium(IV) compared to a vanadium(V) ion, which suggests the reduction is vanadium centered. The center of the redox processes was further corroborated by EPR spectroscopy (Figure 3, right). Both complexes, [Co(Cp*)_**2**_**][1]** and [Co(Cp*)_**2**_**][2]** show a characteristic eight-line spectrum, which is consistent with a single unpaired electron located at the metal (^51^V, 99.75% natural abundance, *I* = 7/2). Values of *g*_iso_ = 1.9715 and *g*_iso_ = 1.9666 and *a* = [274.1915, 268.8744, 258.6040 MHz] and *a* = [256.8, 267.7, 261.9 MHz] for [Co(Cp*)_**2**_**][1]** and [Co(Cp*)_**2**_**][2]**, respectively, are comparable with other vanadium(IV) complexes, for example, a four-coordinate vanadium alkylidene.^121^ The electronic structure was corroborated by scalar relativistic DFT calculations at the ZORA-PBE-D3BJ/def2-TZVPP//ZORA-PBE-D3BJ/def2-SVP level of theory,^122−132^ which indicate a vanadium centered SOMO (quasi-restricted orbital QRO, Figure 3, left) with only small orbital overlap with the supporting ligand.

**Figure 4:**
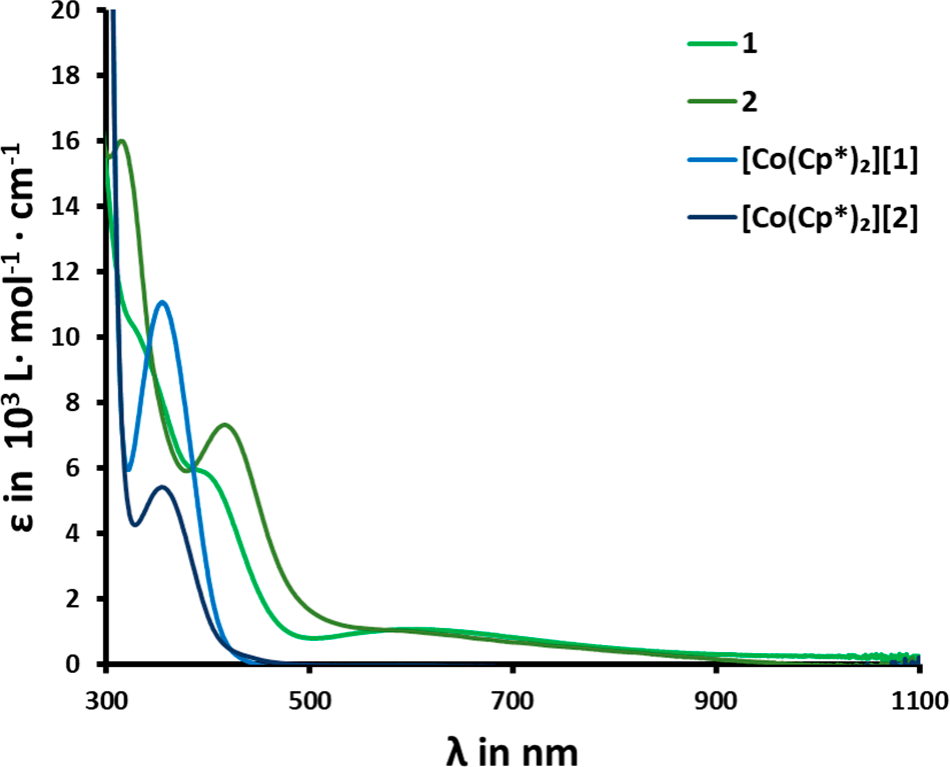
Stacked UV–vis spectra of the complexes **1**, **2**, [Co(Cp*)_**2**_**][1]**, and [Co(Cp*)_**2**_**][2]** in CH_2_Cl_2_ at 298 K.

Further evidence for the stronger donor character of the mesoionic carbene ligand **L**^**1**^ compared to the benzimidazolinylidene **L**^**2**^ can be extracted from UV–vis spectroscopy. When changing from the MIC complex **1** to the benzNHC complex **2**, the charge-transfer (CT) band located at 387 nm for **1** shifts to 420 nm for **2**. Based on time-dependent DFT calculations (Figures S74–S89), we assign this band as essentially ligand-to-ligand charge transfer (LLCT) from the phenolate moieties to the NHC bridge, with only minor contribution from the metal. As the benzNHC is a stronger acceptor ligand compared to the MIC, this band is red-shifted in **2** compared to **1**.^133^ The broad bands located between 500 and 800 nm in **1** and **2** are assigned to the respective ligand-to-metal charge transfers (LMCTs). This assignment is in line with their disappearance in the reduced complexes [Co(Cp*)_**2**_**][1]** and [Co(Cp*)_**2**_**][2]** (see Figure 4). Overall, the results from NMR, electrochemistry, and UV–vis absorption spectroscopy support the notion of MICs being stronger donors than benzNHCs in vanadium complexes.

**Figure 5:**
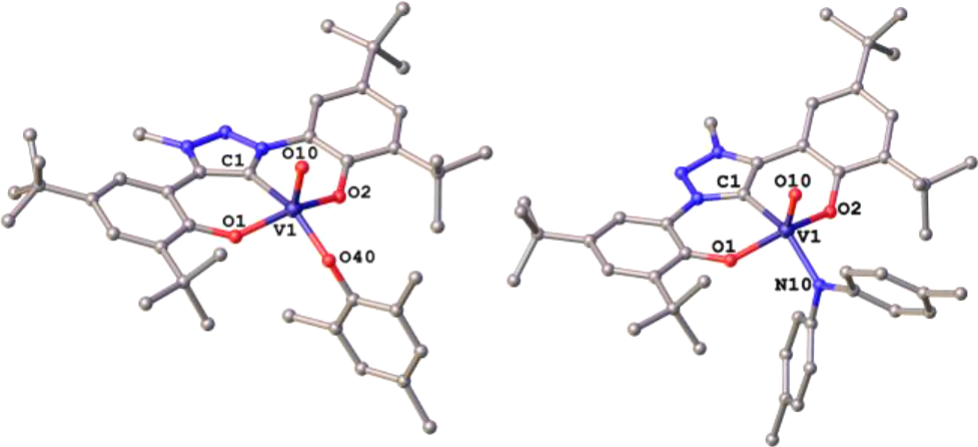
Molecular structures of **3** (left) and **5** (right). Hydrogen atoms and lattice solvent molecules have been omitted for clarity.

**Scheme 3:**
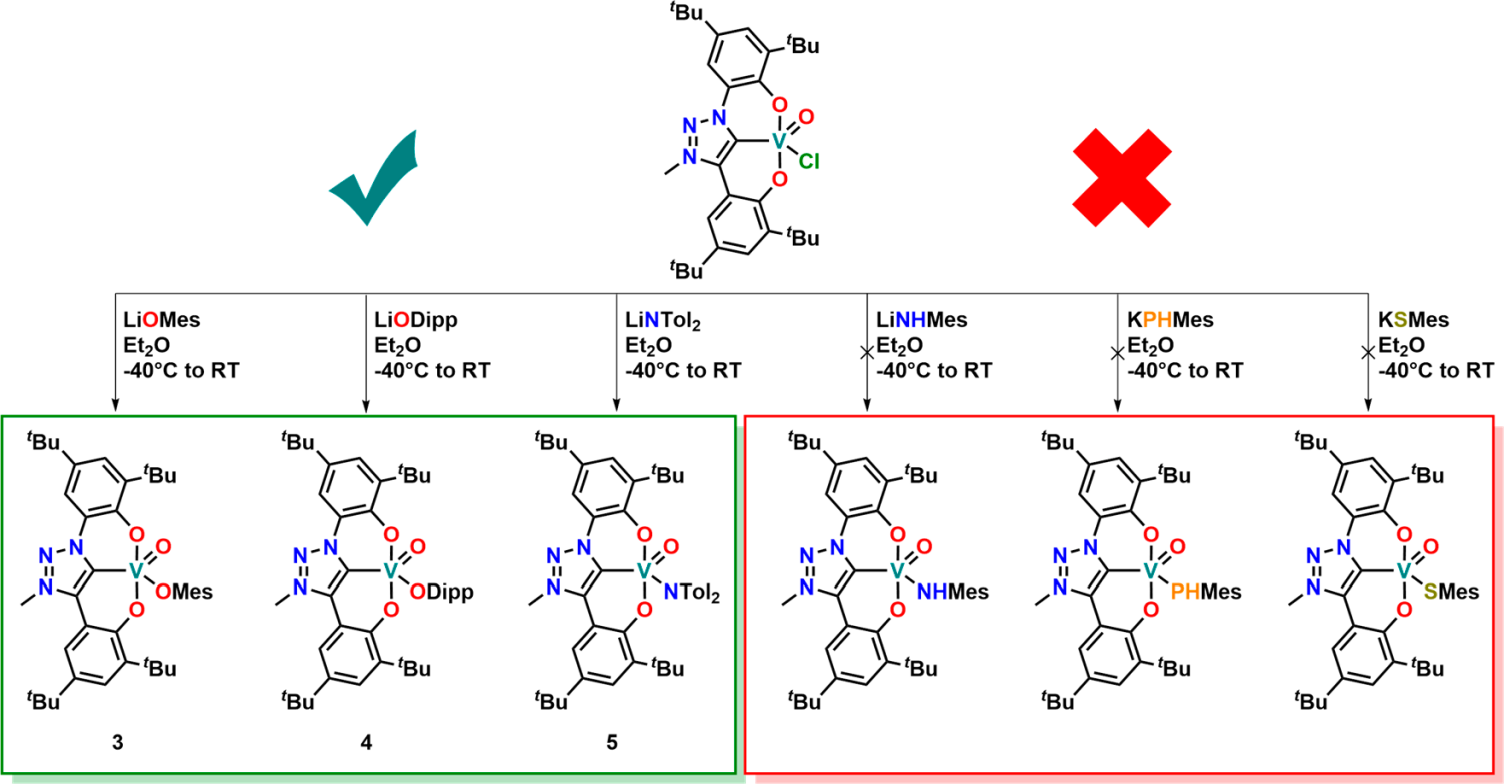
Salt Metathesis Reactivity of Complex **1** toward Various Chalcogen and Pnictogen-Based Nucleophiles as Well as Aryl Anion Donors

To further explore the chemical potential of the triazolinylidene complex **1**, we turned our interest toward salt metathesis replacing the remaining chloride ligand (Scheme 3). Mixing the parent lithium salts with **1** in –40 °C cold diethyl ether, followed by recrystallization from hexane, gave the pure mesitolate (**3**), 2,6-diisopropylphenolate (**4**), and 4,4′-ditolylamide (**5**) complexes in good to moderate yields. To our surprise, the reaction with primary amides (LiNHMes), thiophenolates (KSMes), and phosphanides (KPHMes) failed to give well-defined products under the above-described conditions. Similarly, anionic alkyl or aryl donors, unrelated to their source (lithium or Grignard reagents), resulted in complicated reaction mixtures from which no defined reaction products could be isolated. The NMR spectroscopic and structural analyses (Figure 5) of the complexes **3**–**5** resemble the expected characteristics. For further information we refer to Figures S11–S25 and Tables S2 and S3 in the Supporting Information. Notably, we found that the coligand had a strong influence on the UV–vis spectroscopic features of the complexes. While the halide complex **1** was deep green (λ_max_ = 582 nm; ε = 1000 L mol^–1^ cm^–1^), the phenolate complexes **3** and **4** are a deep purple color (λ_max_ = 534 nm; ε = 4400 L mol^–1^ cm^–1^ for complex **4**), and the amide complex **5** is dark teal (λ_max_ = 660 nm; ε = 7200 L mol^–1^ cm^–1^, compare also Figure S68).

By further probing the versatility of complex **1**, we examined oxo-exchange reactions, with emphasis on generating vanadium imido complexes. To reach this goal, we applied isocyanates as the imido source, liberating carbon dioxide to provide the driving force for this process. Although this strategy was successful for a plethora of vanadium imido complexes,^134,135^ in the present case, even at elevated temperatures (60, 80, or 120 °C), no conversion could be observed. In one experiment (120 °C) with 3,5-bis(trifluoromethyl)phenyl isocyanate, we observed its cyclotrimerization to yield the corresponding isocyanuric amide. It has been reported that electropositive metals, that is, Lewis acids, catalyze this process.^136−140^ However, NHCs and NHOs are also known to be potent catalysts for this transformation.^141,142^ As the presence of free carbenes at elevated temperatures (e.g., due to minimal thermal decomposition) cannot be fully ruled out, the catalytically active species remains unclear.

**Figure 6:**
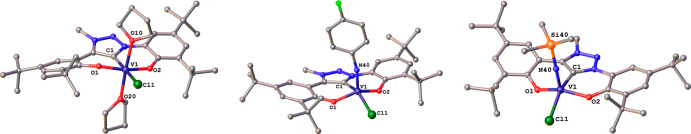
Molecular structures of the vanadium(III) complex **6** and the imido complexes **8** and **10** (from left to right). Hydrogen atoms and lattice solvent molecules have been omitted for clarity.

**Figure 7:**
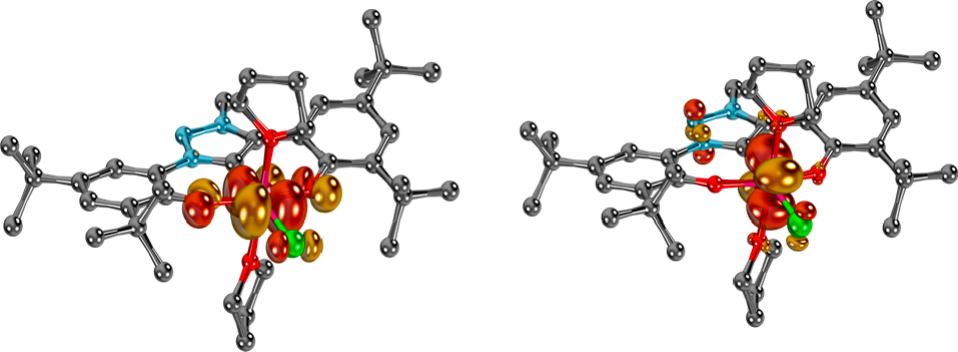
Compound **6** features unpaired electrons in the 3d(*xz*) and 3d(*xy*) orbitals (QROs). Hydrogen atoms have been omitted for clarity.

**Scheme 4:**
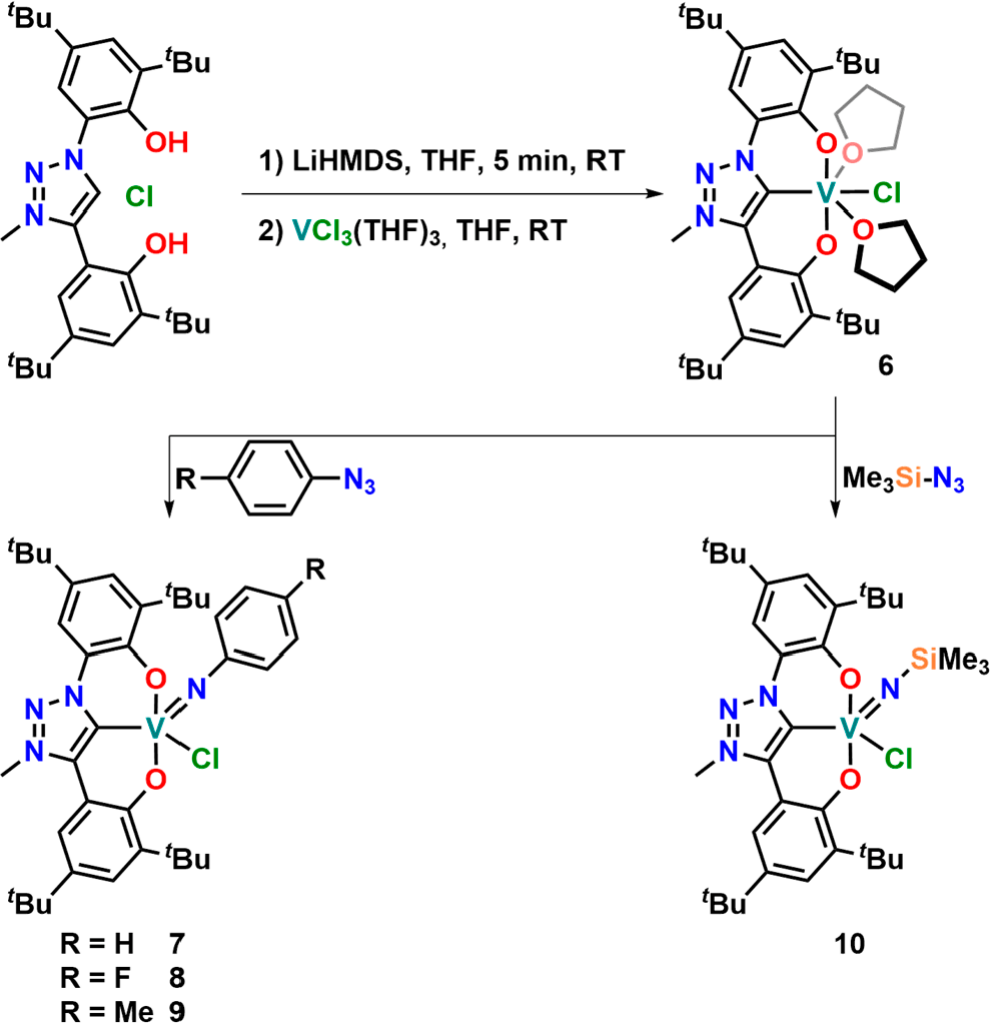
Synthesis of a Vanadium(III) Triazolinylidene Complex and Its Subsequent Conversion into Aryl and Alkyl Imido Complexes

Since the direct generation of imido complexes from the oxo complexes had failed, we sought other strategies. Another versatile access to metal imido complexes is the direct reduction of organic azides by low-valent metal complexes. Thus, we initially aimed to generate a low-valent vanadium(III) complex by deoxygenating the parent vanadium(V) complex **1** with triethylphosphine. Although this strategy met with success for the deoxygenation of molybdenum(VI) benzimidazolinylidene complexes,^143,144^ no useful reaction products could be isolated in the present case. Similarly, switching to other phosphines such as triphenylphosphine or trimethylphosphine turned out to be unproductive. We consequently turned our focus to installing the triazolinylidene ligand **L**^**1**^ directly on vanadium(III).^113^ Although our attempts to isolate the fully deprotonated and free triazolylidene **Li**_**2**_**[L**^**1**^**]** have failed so far, deprotonation of [**H**_**3**_**L**^**1**^**][Cl]** with LiHMDS (HMDS = hexamethyldisilazide) at room temperature, followed by the immediate addition of the deprotonated triazolinylidene to VCl_3_(THF)_3_ resulted in the formation of a brown suspension. After extraction with toluene and washing of the crude solids with hexane, we isolated the desired vanadium(III) complex **6** as an orange powder in 46% yield (Scheme 4). The complex is remarkably sensitive toward air and moisture and decomposes in the glovebox at room temperature within several days even in the solid state. However, it turned out to be stable for a couple of weeks at −40 °C. The formation of the desired vanadium(III) complex was initially evident by the strong paramagnetic nature of its ^1^H NMR, revealing an effective magnetic moment of 2.71 μ_B_, which is in line with the presence of a d^2^ electron configuration and is comparable to previously reported vanadium(III) complexes.^109^ Despite numerous attempts and due to the high sensitivity of the complex, only low-quality crystals of the complex could be obtained from concentrated diethyl ether solutions at −40 °C. In any case, the connectivity of the molecule was unambiguously determined, confirming the vanadium(III) oxidation state (Figure 6, left). In addition to the triazolinylidene and the halide ligands, complex **6** was found to further hold two tetrahydrofuran donors, creating an octahedral coordination environment around the vanadium center. As expected for an *S* = 1 spin system, the EPR spectrum at room temperature did not reveal any observable signals (Figure S90).^121,145^ Computational investigation revealed both SOMOs to be vanadium centered (Figure 7) with only small orbital overlap with the supporting ligand. Accordingly, and in agreement with the experiment, these calculations indicate an adiabatic singlet–triplet gap of Δ*E* = −47 kJ mol^–1^ in favor of the triplet state. Note that the prediction of two SOMOs is consistent with two irreversible waves of **6** observed in the cyclic voltammogram at 0.09 and 1.03 V vs Fc/Fc^+^ in acetonitrile (Figure S73).

Having the vanadium(III) complex **6** in hand, we turned back our interest to the generation of vanadium(V) imido complexes following an azide reduction strategy. As envisioned, complex **6** reacts smoothly with organic azides such as phenyl, 4-fluorophenyl, or 4-methylphenyl azide to form the corresponding vanadium(V) imido complexes **7**, **8,** and **9**. Upon addition of the azides to benzene solutions of complex **6** and heating the samples to 60 °C, gas evolution was observed, and the reddish-brown solution gradually changed to deep green. The reaction could be followed by ^1^H NMR spectroscopy, revealing the clean conversion to the desired complexes, which can be isolated by evaporation of benzene and washing the green powders with hexane in yields of 46–100% (Scheme 4). While the proton resonances of the complexes show the expected characteristics, the ^13^C carbene resonance could not be observed again due to the large quadrupole moment of the ^51^V nucleus. Similar problems occurred during our attempts to observe the imido nitrogen atom using ^1^H–^15^N HMBC NMR experiments. However, in the ^51^V NMR, the complexes show resonances at −398.0, −396.8, and −378.2 ppm for **7**, **8**, and to **9** respectively (Figures S32, S38, and S43). To unambiguously prove the structural identity, X-ray quality crystals of complex **8** were grown from concentrated diethyl ether solution (Figure 6, middle). The complex crystallizes in the monoclinic space group *P*2_1_/*n* with one molecule in the asymmetric unit. The vanadium center is penta-coordinate by the ligand, the imido nitrogen atom, and a chlorido ligand in a slightly distorted square pyramidal coordination environment (τ_5_ = 0.10). The V1–N40 distance was found to be 1.644(2) Å and is comparable to previously reported vanadium(V) imido complexes. The metal carbene distance V1–C1 was found to be 2.048(2) Å, which compares well to complex **1**. The structural parameters of the ligand are similar to complex **1** and can be found in the Supporting Information in Tables S2 and S3.

In sight of the swift reduction of organic azides, we next turned our interest toward the use of “inorganic” azides, which we envisioned to form vanadium nitrido complexes. These complexes are relevant intermediates in the context of dinitrogen activation and valorization. Indeed, reacting complex **6** with 1 equiv of TMS-N_3_ resulted in the clean conversion to diamagnetic **10** at 60 °C (Scheme 4). After workup, the ^1^H NMR spectrum of the new complex shows a single resonance at −0.31 ppm integrating nine protons, which is indicative of the remainder of the TMS group on the nitrogen atom and thus the formation of a TMS-imido complex. Indeed, Mindiola and co-workers recently reported that the cleavage of a TMS group from a TMS-imido ligand to form the corresponding nitride complex is not trivial also for other group 5 metals, for example, tantalum(V).^146^ The ^51^V-NMR shows a single resonance at −473.5 ppm (Figure S48) which is high-field shifted compared to the aryl-imido complexes **7**–**9**. However, this high-field shift can be attributed to the stronger donor character of alkyl/TMS imido versus and aryl imido ligands. Unfortunately, due to the quadrupolar moment of vanadium, the detection of both the imido nitrogen atom as well as of the silicon atom using ^29^Si NMR spectroscopy failed. Nevertheless, X-ray quality crystals of complex **10** could be grown from slow evaporation of a concentrated diethyl ether solution at room temperature (Figure 6, right). The structural analysis confirmed the formation of the terminal imido instead of the desired nitrido complex. The structural properties of complex **10** resemble those of complex **8** and are given in the Supporting Information in Tables S2 and S3. In contrast to previous reports,^147^ applying sodium azide as a nitrogen/azide source resulted in complicated (paramagnetic) mixtures, from which no defined products could be isolated.

## Conclusions

We have extended the use of mesoionic carbenes with an early transition metal, vanadium. Using combined spectroscopic, electrochemical, and computational methods, we have shown that mesoionic carbenes are stronger donors than classical NHCs in early transition-metal chemistry as well. The high-valent oxo-vanadium(V) complexes are of moderate use for salt metathesis, reacting cleanly only with phenolates and secondary amides. Remarkably, the mesoionic carbene ligand supports vanadium in three oxidations states (III/IV/V). This is a rare report of a structurally characterized low-valent vanadium(III) complex supported by an NHC ligand^112−114,148^ and the first of a mesoionic carbene stabilizing a low-valent early transition metal. Complex **6** is a powerful two-electron reductant and forms the corresponding high-valent vanadium(V) imido complexes with azides. In recent years, NHC-imido vanadium complexes have attracted a large interest as polymerization catalysts as well as in nitrene transfer reactions.^149^ Following the concept of extreme π-loading effects^150^ and given the numerous examples of superior activity of MIC based catalysts over their NHC congeners,^151^ we believe that our findings will create further interest in the use of mesoionic carbenes in early transition-metal-mediated reactions. Additionally, the redox-active nature of the phenolate tethers will also be of large interest in other catalytic reactions as well as small molecule activation.

## Experimental Section

### General Remarks

If not otherwise mentioned, all transformations were carried out in an argon-filled glovebox under inert conditions. Solvents were dried by an MBraun SPS system and stored over activated molecular sieves (3 Å) for at least 1 day. C_6_D_6_ was dried over sodium/benzophenone and CDCl_3_ and CD_2_Cl_2_ over calcium hydride, followed by vacuum transfer and three freeze–pump–thaw cycles. The proligand **[H**_**3**_**L**^**1**^**][Cl]** was synthesized following a literature known procedure.^44^ LiOMes, LiN(Tol)_2_, and LiNHMes were obtained by deprotonating the corresponding phenol or aniline in pentane using *n*-BuLi and filtering off the products. In a similar way, KSMes and KPHMes were obtained by deprotonating the corresponding thiophenol and primary phosphine using KHMDS in toluene.^152^ 4-Methylphenyl azide^153^ and 4-fluorophenyl azide^154^ were synthesized following previously reported methods using *tert*-butyl nitrite and trimethylsilyl azide in acetonitrile. Decamethylcobaltocene, triethylphosphine, trimethylsilyl azide, sodium azide, and VO(O^*i*^Pr)_3_ were used as received by commercial suppliers. NMR spectra were collected at ambient temperature on a Bruker AV-300, Ascent 400, AV-500, or an Ascent 700 spectrometer. ^1^H and ^13^C NMR chemical shifts (δ) are reported in ppm and were calibrated to residual solvent peaks. ^51^V NMR chemical shifts have been calibrated to VOCl_3_ in CDCl_3_ as an external standard. It needs to be mentioned at this point that ^51^V NMR is extremely sensitive and minor impurities (0.5% < ) can still be observed, even though the remaining characterization data appear to be clean. This explains the minor impurities observed in the ^51^V NMR spectra of the complexes **1**, **4**, **5**, **7**, **8**, **9** and **10**. Elemental analysis was performed using an Elementar vario microcube instrument. IR spectra were collected using a Bruker Alpha IR spectrometer. Cyclic voltammetry was recorded using a BioLogic potentiostat and a three-electrode array (working electrode: glassy carbon, counter electrode: platinum, reference electrode: silver). EPR spectra were recorded from 5 mM solutions at 300 K using a Bruker Magnettech 5000 EPR spectrometer (microwave frequency, 9.46 GHz; microwave power, 5 mW; modulation amplitude, 0.5 mT). CW spectra were processed using MATLAB and EasySpin software package (see Supporting Information for details).^155^

### Synthetic Procedures

#### General Procedure for the Synthesis of **1** and **2**

The synthesis of the complexes was adapted from the literature.^54^ If not otherwise stated, the corresponding azolium salt (1 equiv, 1 mmol) was mixed with [VO(O^*i*^Pr)] (1 or 1.2 equiv) in THF (30 mL) and stirred at room temperature for 2 days. The solvent was evaporated, and the resulting solids were suspended in hexane (20 mL) and stirred for 1 h at room temperature. The resulting suspension was filtered, and the solids were washed with minimal amounts of pentane (10 mL) and dried on the frit inside the glovebox to give the desired vanadium(V) complexes in yields of 78% and higher.

##### [VOCl(L^1^)] (**1**)

From **[H**_**3**_**L**^**1**^**][Cl]** (1 equiv., 1 mmol, 528 mg) and VO(O^*i*^Pr)_3_ (1 equiv., 1 mmol, 244 mg). Yield: 85% (0.85 mmol, 503 mg). ^1^H NMR (C_6_D_6_, 298 K, 700 MHz, in ppm): δ = 8.06 (d, *J* = 2.4 Hz, 1H, Aryl-*H*), 7.75 (d, *J* = 2.4 Hz, 1H, Aryl-*H*), 7.73 (d, *J* = 2.4 Hz, 1H, Aryl-*H*), 7.10 (d, *J* = 2.4 Hz, 1H, Aryl-*H*). 3.17 (s, 3H, N-C*H*_*3*_), 1.87 (s. 18H, C(C*H*_*3*_)_3_), 1.38 (s. 9H, C(C*H*_*3*_)_3_). 1.35 (s. 9H, C(C*H*_*3*_)_3_); ^13^C{^1^H} NMR(C_6_D_6_, 298 K, 176 MHz, in ppm): δ = 162.5 (Aryl-*C*-OH), 1.55 (Aryl-*C*-OH), 143.7 (Aryl-*C*), 142.8 (Aryl-*C*), 141.6 (Aryl-*C*), 140.7 (Aryl-*C*), 140.1 (Aryl-*C*), 127.1 (Aryl-*C*H), 126.3 (Aryl-*C*H), 124.3 (Aryl-*C*), 118.6 (Aryl-*C*H), 113.6 (Aryl-*C*H), 113.2 (Aryl-*C*), 39.7 (N-*C*H_3_), 36.9 (*C*(CH_3_)_3_), 36.7 (*C*(CH_3_)_3_), 35.4 (*C*(CH_3_)_3_), 35.2 (*C*(CH_3_)_3_), 32.2 (C(*C*H_3_)_3_), 32.1 (C(*C*H_3_)_3_), 30.8 (C(*C*H_3_)_3_), 30.7 (C(*C*H_3_)_3_); ^51^V NMR(C_6_D_6_, 298 K, 184 MHz, in ppm): δ = −533 (s). Elemental analysis (%) calcd for C_31_H_43_N_3_O_3_VCl: C, 62.89; H, 7.32; N, 7.10; found C, 62.53; H, 7.12; N, 6.86.

##### [VOCl(L^2^)] (**2**)

From [**H**_**3**_**L**^**2**^**][Cl]** (1 equiv., 1 mmol, 563 mg) and VO(O^*i*^Pr)_3_ (1.2 equiv., 1.2 mmol, 293 mg). Yield: 91% (0.91 mmol, 571 mg). ^1^H NMR (C_6_D_6_, 298 K, 700 MHz, in ppm): δ = 7.81 (m, 2H Aryl-*H*), 7.67 (d, *J* = 2.4 Hz, 2H, Aryl-*H*), 7.65 (d, *J* = 2.4 Hz, 2H, Aryl-*H*), 7.00 (m, 2H, Aryl-*H*), 1.87 (s, 18H, C(C*H*_*3*_)_3_), 1.36 (s, 18H, C(C*H*_*3*_)_3_); ^13^C{^1^H} NMR(C_6_D_6_, 298 K, 176 MHz, in ppm): δ = 155.4 (Aryl-*C*-OH), 143.3 (Aryl-*C*), 139.9 (Aryl-*C*), 133.2 (Aryl-*C*), 128.6 (Aryl-*C*), 125.6 (Aryl-*C*H), 123.6 (Aryl-*C*), 123.4 (Aryl-*C*H), 115.4 (Aryl-*C*H), 114.4 (Aryl-*C*H), 36.2 (*C*(CH_3_)_3_), 34.9 (*C*(CH_3_)_3_), 31.7 (C(*C*H_3_)_3_), 30.3 (C(*C*H_3_)_3_); ^51^V NMR (C_6_D_6_, 298 K, 184 MHz, in ppm): δ = −503 (s). Elemental analysis (%) calcd for C_35_H_44_N_2_O_3_VCl: C, 67.03; H, 7.07; N, 4.47; found C, 66.94; H, 7.12; N, 4.21.

#### General Procedure for [Co(Cp*)_2_][1] and [Co(Cp*)_2_][2]

The parent vanadium(V) complexes **1** or **2** (1 equiv.) were dissolved in THF and stirred for 10 min at room temperature. A solution of decamethylcobaltocene (1 equiv.) in THF was added, and the reaction mixtures were stirred for 5 h at room temperature.

##### [Co(Cp*)_2_][VOCl(L^1^)] ([Co(Cp*)_2_][1])

From complex **1** (1 equiv., 0.25 mmol, 148 mg) and Co(Cp*)_2_ (1 equiv., 0.25 mmol, 83 mg). After 5 h the reaction was filtered, and the solvent was evaporated. The greenish-gray residue was dissolved in CH_2_Cl_2_ and filtered again and concentrated to 1 mL. Hexane was added until the solution became turbid. One drop of dichloromethane was added to redissolve all solids, and the mixture was left to stand in an openly capped vial inside the glovebox for 2 days to let the dichloromethane evaporate. This formed large green blocks of [Co(Cp*)_**2**_**][1]**. Yield: 76% (0.19 mmol, 175.1 mg). Elemental analysis (%) calcd for C_51_H_74_N_3_O_3_VCoCl: C, 66.48; H, 7.99; N, 4.56; found C, 66.75; H, 7.66; N, 4.28, μ_eff_ = 1.74 μB.

##### [Co(Cp*)_2_][VOCl(L^2^)] ([Co(Cp*)_2_][2])

From complex **2** (1 equiv., 0.25 mmol, 157 mg) and Co(Cp*)_2_ (1 equiv., 0.25 mmol, 83 mg). After 5 h, the green suspension was filtered, and the green solids were washed with another 5 mL of THF and 5 mL of pentane. The green solids were then dissolved in 5 mL of dichloromethane, and the solution was concentrated to 1 mL. Hexane was added until the solution became turbid. One drop of dichloromethane was added to redissolve all solids, and the mixture was left to stand in an openly capped vial for 2 days inside the glovebox to let the dichloromethane evaporate. This formed large green blocks of [Co(Cp*)_**2**_**][1]**. Yield: 69% (0.172 mmol, 165 mg). Elemental analysis (%) calcd for C_55_H_74_N_2_O_3_VCoCl: C, 69.06; H, 7.80; N, 2.93; found C, 68.37; H, 7.54; N, 2.82, μ_eff_ = 1.68 μB.

#### General Procedure for the Salt Metathesis Reactions

In a 20 mL scintillation vial, vanadium complex **1** was dissolved in 5 mL Et_2_O and cooled to −40 °C. In a separate vial, the corresponding lithium salt (phenolate or amide) was dissolved/suspended in 2 mL Et_2_O and cooled to −40 °C as well. This solution was then added dropwise at −40 °C to the solution of the vanadium complex and slowly warmed to room temperature overnight while stirring. The deeply colored solutions are filtered to remove any lithium chloride formed during the reaction, and the solvent was evaporated under high vacuum. The colored residues were then dissolved in hexane, filtered again, and concentrated to approximately 0.5 mL. Storing these solutions at −40 °C resulted in the formation of the corresponding complexes in moderate to good yields overnight.

##### [VO(OMes)(L^1^)] (**3**)

Following the general procedure, LiOMes (1 equiv., 0.1 mmol, 14 mg) was added to **1** (1 equiv., 0.1 mmol, 59 mg). Dark purple solid. Yield: 98% (68 mg, 0.098 mmol). ^1^H NMR (C_6_D_6_, 298 K, 400 MHz, in ppm) δ 8.17 (d, *J* = 2.4 Hz, 1H, Aryl-*H*), 7.73 (d, *J* = 2.4 Hz, 1H, Aryl-*H*), 7.69 (d, *J* = 2.5 Hz, 1H, Aryl-*H*), 7.13 (d, *J* = 2.3 Hz, 1H, Aryl-*H*), 6.92 (s, 1H, Mesityl-*H*), 6.71 (s, 1H, Mesityl-*H*), 3.07 (s, 3H, N-C*H*_*3*_), 3.02 (s, br, 3H, C-C*H*_*3*_), 2.32 (s, br, 3H, C-C*H*_*3*_), 2.20 (s, 3H, C-C*H*_*3*_), 1.69 (s, 9H, C-(C*H*_*3*_)_3_), 1.62 (s, 9H, C-(C*H*_*3*_)_3_), 1.41 (s, 9H, C-(C*H*_*3*_)_3_), 1.38 (s, 9H, C-(C*H*_*3*_)_3_). ^13^C NMR (C_6_D_6_, 298 K, 101 MHz, in ppm) δ 162.02 (Aryl-*C*-OH), 155.16 (Aryl-*C*-OH), 141.59 (Aryl-*C*), 141.39 (Aryl-*C*), 140.49 (Aryl-*C*), 139.86 (Aryl-*C*), 132.23 (Aryl-*C*), 129.51 (Aryl-*C*), 128.99 (Aryl-*C*H), 128.87 (Aryl-*C*H), 126.18 (Aryl-*C*H), 125.53 (Aryl-*C*H), 125.27 (Aryl-*C*H), 122.96 (Aryl-*C*H), 118.52 (Aryl-*C*H), 113.82 (Aryl-*C*H), 113.68 (Aryl-*C*H), 38.82 (N-*C*H_3_), 36.21 (*C*(CH_3_)_3_), 36.11 (*C*(CH_3_)_3_), 34.81 (*C*(CH_3_)_3_), 34.66 (*C*(CH_3_)_3_), 31.86 (C(*C*H_3_)_3_), 31.77 (C(*C*H_3_)_3_), 30.04 (C(*C*H_3_)_3_), 29.90 (C(*C*H_3_)_3_), 21.13 (C-*C*H_3_), 18.49 (C-*C*H_3_), 17.99 (C-*C*H_3_). ^51^V NMR (C_6_D_6_, 298 K, 79 MHz, in ppm) δ −566.66. Elemental analysis (%) calcd for C_40_H_54_N_3_O_4_V_1_: C, 69.44; H, 7.87; N, 6.07; found C, 69.81; H, 8.16; N, 5.91.

##### [VO(ODipp)(L^1^)] (**4**)

Following the general procedure, LiODipp (1 equiv., 0.05 mmol, 10 mg) was added to **1** (1 equiv., 0.05 mmol, 30 mg). Dark purple solid. Yield: 96% (35 mg, 0.048 mmol). ^1^H NMR (C_6_D_6_, 298 K,700 MHz, in ppm) δ 8.26 (d, 1H, J = 2.3 Hz, Aryl-*H*), 7.84 (d, 1H, J = 2.0 Hz, Aryl-*H*), 7.79 (d, 1H, J = 2.4 Hz, Aryl-*H*), 7.40 (dd, 1H, J = 7.6 Hz, J = 1.6 Hz, Aryl-*H*), 7.22 (d, 1H, J = 2.3 Hz, Aryl-*H*), 7.15 (dd, 1H, J = 7.8 Hz, J = 2.0 Hz, Aryl-*H*), 7.00 (t, 1H, J = 7.6 Hz, Aryl-*H*), 4.95 (m, 1H, C*H*(CH_3_)_2_), 3.55 (m, 1H, C*H*(CH_3_)_2_), 3.16 (s, 3H, NC*H*_3_), 1.83 (d, 6H, J = 6.8 Hz, CHC*H*_3_), 1.82 (s, 9H, CC*H*_3_), 1.57 (d, 6H, J = 6.9 Hz, CHC*H*_3_), 1.72 (s, 9H, CC*H*_3_), 1.48 (s, 9H, CC*H*_3_), 1.47 (s, 9H, CC*H*_3_), 1.22 (d, 6H, J = 7.0 Hz, CHC*H*_3_), 1.22 (d, 6H, J = 6.8 Hz, CHC*H*_3_). ^13^C NMR (C_6_D_6_, 175 MHz, in ppm): δ 167.27 (*C*-OH), 162.52 (*C*-OH), 155.68, 142.07, 141.80, 141.67, 140.99, 140.38, 140.11, 139.21, 126.63, 126.00, 125.53, 124.02, 123.53, 123.46, 118.88, 114.14, 114.07, 39.20 (NC*H*_3_), 36.56 (*C*CH_3_), 36.51 (*C*CH_3_), 35.12 (*C*CH_3_), 34.99 (*C*CH_3_), 32.16 (C*C*H_3_), 32.05 (C*C*H_3_), 30.59 (C*C*H_3_), 30.38 (-C*C*H_3_), 28.58 (*C*HCH_3_), 28.55 (*C*HCH_3_), 24.57 (CH*C*H_3_), 24.40 (CH*C*H_3_), 24.06 (CH*C*H_3_), 23.37 (CH*C*H_3_). Elemental analysis (%) calcd for C_43_H_60_N_3_O_4_V_1_: C, 70.34; H, 8.24; N, 5.73; found C, 69.04; H, 8.21; N, 5.68. The low carbon value results from potential carbide formation, which is a common problem for early transition metals.

##### [VO(NTol_2_)(L^1^)] (**5**)

Following the general procedure, LiN(Tol)_2_ (1 equiv., 0.05 mmol, 10 mg) was added to **1** (1 equiv., 0.05 mmol, 30 mg). Dark blue solid. Yield: 56% (27 mg, 0.028 mmol). ^1^H NMR (C_6_D_6_, 298 K, 700 MHz, in ppm): δ 8.19 (d, J = 2.5 Hz, 1H, Aryl-*H*), 8.14 (d, J = 8.3 Hz, 2H, Aryl-*H*), 7.17 (s, 1H, Aryl-*H*), 7.16 (d, J = 8.3 Hz, 2H, Aryl-*H*), 7.13 (d, J = 8.6 Hz, 2H, Aryl-*H*), 7.11 (d, J = 2.3 Hz, 1H, Aryl-*H*), 6.83–6.80 (m, 2H, Aryl-*H*), 3.07 (s, 3H, NC*H*_3_), 2.23 (s, 3H, C*H*_3_), 1.88 (s, 3H, C*H*_3_), 1.70 (s, 18H, CC*H*3), 1.41 (s, 9H, CC*H*3), 1.39 (s, 9H, CC*H*3). ^13^C NMR (C_6_D_6_, 298 K,175 MHz, in ppm): δ 162.48 (*C*-OH), 155.51 (*C*-OH), 154.80, 151.36, 142.82, 141.70, 140.29, 140.11, 139.00, 136.01, 12961, 129.48, 128.42, 125.88, 125.86, 125.15, 122.82, 121.44, 118.80, 113.96, 38.65 (N*C*H_3_), 36.38 (*C*CH_3_), 36.25 (*C*CH_3_), 34.70 (*C*CH_3_), 34.54 (*C*CH_3_), 31.90 (C*C*H_3_), 31.81 (C*C*H_3_), 30.59 (C*C*H_3_), 30.50 (C*C*H_3_), 20.89 (*C*H_3_), 20.66 (*C*H_3_). Elemental analysis (%) calcd for C_45_H_57_N_4_O_3_V: C, 71.79; H, 7.63; N, 7.44; found C, 71.44; H, 7.28; N, 7.16.

##### [VCl(L^1^)(THF)_2_] (**6**)

In a 20 mL scintillation vial, triazolium proligand **[H**_**3**_**L**^**1**^**][Cl]** (1 equiv., 1.50 mmol, 792 mg) was dissolved in 5 mL THF. To the stirring, bright yellow solution, a THF solution of lithium hexamethyldisilazide (3.3 equiv., 4.95 mmol, 828 mg) was added dropwise over a period of 10 min. After 30 min at ambient temperature, a suspension of VCl_3_(THF)_3_ (1 equiv., 1.50 mmol, 560 mg) was added at once. The mixture turned dark red/brown after the addition. After 15 h the mixture was filtered, the solvent was removed by evaporation. The dark brown residue was redissolved in toluene, and precipitated lithium chloride was filtered off. Toluene was removed under reduced pressure, and the residue was washed several times with a small amount hexane to give **6** as an orange-brown powder (496 mg, 46%). μ_eff_ (Evans method, C_6_D_6_) = 2.71 μ_B_. Elemental analysis (%) calcd for C_35_H_51_N_3_O_3_V_1_·LiCl: C, 60.87; H, 7.44; N, 6.08; found C, 61.00; H, 7.13; N 6.04.

#### General Procedure for Imido Complexes

In a 10 mL J. Young Schlenk flask, vanadium(III) complex **6** (1.0 equiv., 0.10 mmol, 72 mg) was dissolved in 5 mL of benzene. The corresponding azide was added to the solution, and the mixture was heated to 60 °C. After 24 h, the solvent was lyophilized. Residues were washed several times with hexane to afford clean product. X-ray quality crystals were grown from concentrated diethyl ether solution at ambient temperature.

##### [V(N-Ph)Cl(L^1^)] (**7**)

From azidobenzene (1.2 equiv., 0.12 mmol, 14 mg). Dark green solid. Yield: 100% (67 mg, 0.10 mmol). ^1^H NMR (C_6_D_6_, 298 K, 400 MHz, in ppm) δ 8.18 (d, *J* = 2.4 Hz, 1H, Aryl-*H*), 7.80 (d, *J* = 2.3 Hz, 1H, Aryl-*H*), 7.78 (d, *J* = 2.4 Hz, 1H, Aryl-*H*), 7.18 (s, 1H, Aryl-*H*), 6.68–6.61 (m, 2H, Phenyl-*H*), 6.38 (dd, *J* = 8.5, 7.0 Hz, 2H, Phenyl-*H*), 6.31–6.25 (m, 1H, Phenyl-*H*), 3.16 (s, 3H, NC*H*_3_), 1.99 (s, 9H, C(C*H*_3_)_3_), 1.97 (s, 9H, C(C*H*_3_)_3_), 1.38 (s, 9H, C(C*H*_3_)_3_), 1.35 (s, 9H, C(C*H*_3_)_3_). ^13^C NMR (C_6_D_6_, 298 K, 101 MHz, in ppm) δ 142.22 (Aryl-*C*), 141.35 (Aryl-*C*), 140.13 (Aryl-*C*), 127.39 (Aryl-*C*H), 127.23 (Aryl-*C*H), 126.03 (Aryl-*C*H), 125.37 (Aryl-*C*H), 124.83 (Aryl-*C*H), 123.74 (Aryl-*C*H), 118.24 (Aryl-*C*H), 113.32 (Aryl-*C*H), 112.53 (Aryl-*C*H), 38.69 (N(*C*H_3_)), 36.56 (*C*(CH_3_)_3_), 36.44 (*C*(CH_3_)_3_), 34.87 (*C*(CH_3_)_3_), 34.71 (*C*(CH_3_)_3_), 31.82 (C(*C*H_3_)_3_), 31.74 (C(*C*H_3_)_3_), 30.26 (C(*C*H_3_)_3_), 30.20 (C(*C*H_3_)_3_). ^51^V NMR (C_6_D_6_, 298 K, 79 MHz, in ppm) δ −397.98. Elemental analysis (%) calcd for C_37_H_48_N_4_O_2_V_1_Cl_1_·C_6_H_6_: C, 69.30; H, 7.30; N, 7.52; found C, 69.18; H, 7.51; N 6.94.

##### [V(N-4-F-phenyl))Cl(L^1^)] (**8**)

From 1-azido-4-fluorobenzene (1.1 equiv., 0.11 mmol, 15 mg). Dark green solid. Yield: 83% (57 mg, 0.083 mmol). ^1^H NMR (C_6_D_6_, 298 K, 400 MHz, in ppm) δ 8.18 (d, *J* = 2.4 Hz, 1H, Aryl-*H*), 7.80 (d, *J* = 2.3 Hz, 1H, Aryl-*H*), 7.78 (d, *J* = 2.5 Hz, 1H, Aryl-*H*), 7.18 (d, *J* = 2.4 Hz, 1H, Aryl-*H*), 6.46 (dd, *J* = 9.0, 5.1 Hz, 2H, Aryl-*H*), 5.94 (t, *J* = 8.8 Hz, 2H, Aryl-*H*), 3.15 (s, 3H, NC*H*_3_), 1.98 (s, 9H, C(C*H*_3_)_3_), 1.97 (s, 9H, C(C*H*_3_)_3_), 1.37 (s, 9H, C(C*H*_3_)_3_), 1.34 (s, 9H, C(C*H*_3_)_3_). ^13^C NMR (C_6_D_6_, 298 K, 101 MHz, in ppm) δ 142.36 (Aryl-*C*), 141.47 (Aryl-*C*), 126.09 (Aryl-*C*H), 125.44 (Aryl-*C*H), 123.70 (Aryl-*C*H), 118.22 (Aryl-*C*H), 115.06 (Aryl-*C*H), 114.83 (Aryl-*C*H), 113.31 (Aryl-*C*H), 38.65 (N*C*H_3_), 36.56 (*C*(CH_3_)_3_), 36.43 (*C*(CH_3_)_3_), 31.81 (C(*C*H_3_)_3_), 31.73 (C(*C*H_3_)_3_), 30.23 (C(*C*H_3_)_3_), 30.17 (C(*C*H_3_)_3_). ^19^F NMR (C_6_D_6_, 298 K, 376 MHz, in ppm) δ −109.57. ^51^V NMR (C_6_D_6_, 298 K, 79 MHz, in ppm) δ −396.83. Elemental analysis (%) calcd for C_37_H_47_N_4_O_2_F_1_V_1_Cl_1_·0.5 C_6_H_6_: C, 66.34; H, 6.96; N, 7.74; found C, 63.15; H, 6.80; N 7.39. The low carbon value results from potential carbide formation, which is a common problem for early transition metals.

##### [V(N-4-Me-phenyl))Cl(L^1^)] (**9**)

From 1-azido-4-methylbenzene (1.1 equiv., 0.11 mmol, 15 mg). Dark green solid. Yield: 46% (31 mg, 0.046 mmol). ^1^H NMR (C_6_D_6_, 298 K, 400 MHz, in ppm) δ 8.18 (d, *J* = 2.4 Hz, 1H, Aryl-*H*), 7.80 (d, *J* = 2.3 Hz, 1H, Aryl-*H*), 7.78 (d, *J* = 2.5 Hz, 1H, Aryl-*H*), 7.18 (d, *J* = 2.3 Hz, 1H, Aryl-*H*), 6.59 (d, *J* = 8.4 Hz, 2H, Aryl-*H*), 6.19–6.16 (m, 2H, Aryl-*H*), 3.16 (s, 3H, NC*H*_3_), 2.00 (s, 9H, C(C*H*_3_)_3_), 1.99 (s, 9H, C(C*H*_3_)_3_), 1.62 (s, 3H, CC*H*_3_), 1.38 (s, 9H, C(C*H*_3_)_3_), 1.36 (s, 9H, C(C*H*_3_)_3_). ^13^C NMR (C_6_D_6_, 298 K, 101 MHz, in ppm) δ 142.07 (Aryl-*C*), 141.18 (Aryl-*C*), 137.54 (Aryl-*C*), 128.62 (Aryl-*C*H), 125.97 (Aryl-*C*H), 125.32 (Aryl-*C*H), 124.93 (Aryl-*C*H), 123.79 (Aryl-*C*H), 118.26 (Aryl-*C*H), 113.34 (Aryl-*C*H), 112.58 (Aryl-*C*H), 38.62 (N*C*H_3_), 36.58 (*C*(CH_3_)_3_), 36.46 (*C*(CH_3_)_3_), 31.84 (C(*C*H_3_)_3_), 31.76 (C(*C*H_3_)_3_), 30.27 (C(*C*H_3_)_3_), 30.21 (C(*C*H_3_)_3_), 20.88 (C*C*H_3_). ^51^V NMR (C_6_D_6_, 298 K, 79 MHz, in ppm) δ −378.16. Due to the high sensitivity of the complex, no satisfactory elementary analysis could be obtained.

##### [V(N-TMS))Cl(L^1^)] (**10**)

From azidotrimethylsilane (1.1 equiv., 0.11 mmol, 13 mg). Dark greyish-green solid. Yield: 83% (55 mg, 0.083 mmol). ^1^H NMR (C_6_D_6_, 298 K, 400 MHz, in ppm) δ 8.20 (d, *J* = 2.3 Hz, 1H, Aryl-*H*), 7.77 (d, *J* = 2.3 Hz, 1H, Aryl-*H*), 7.74 (d, *J* = 2.4 Hz, 1H, Aryl-*H*), 7.24 (d, *J* = 2.3 Hz, 1H, Aryl-*H*), 3.27 (s, 3H, NC*H*_3_), 1.96 (s, 9H, C(C*H*_3_)_3_), 1.95 (s, 9H, C(C*H*_3_)_3_), 1.37 (s, 9H, C(C*H*_3_)_3_), 1.34 (s, 9H, C(C*H*_3_)_3_), −0.31 (s, 9H, Si(C*H*_3_)_3_). ^13^C NMR (C_6_D_6_, 298 K, 101 MHz, in ppm) δ 141.73 (Aryl-*C*), 140.82 (Aryl-*C*), 126.09 (Aryl-*C*H), 125.37 (Aryl-*C*H), 117.93 (Aryl-*C*H), 113.11 (Aryl-*C*H), 112.55 (Aryl-*C*H), 38.77 (N*C*H_3_), 36.56 (*C*(CH_3_)_3_), 36.44 (*C*(CH_3_)_3_), 34.81 (*C*(CH_3_)_3_), 34.64 (*C*(CH_3_)_3_), 31.79 (C(*C*H_3_)_3_), 31.71 (C(*C*H_3_)_3_), 30.33 (C(*C*H_3_)_3_), 30.25 (C(*C*H_3_)_3_), 0.02 (Si(*C*H_3_)_3_). ^51^V NMR (C_6_D_6_, 298 K, 79 MHz, in ppm) δ −473.46. Elemental analysis (%) calcd for C_34_H_52_N_4_O_2_Si_1_V_1_Cl_1_: C, 61.57; H, 7.90; N, 8.45; found C, 59.05; H, 8.00; N 8.27. The low carbon value results from potential carbide formation, which is a common problem for early transition metals.

#### X-ray Crystallography

Single crystals for X-ray diffraction experiments were performed at the analytical facility of the University of Paderborn or at the University of Innsbruck. All crystals were kept at 120(2) K or 153(2) K throughout data collection. Data collection was performed using either the ApexIII software package on a Bruker D8 Venture (Paderborn) or on a Bruker D8 Quest instrument (Innsbruck). Data refinement and reduction were performed using the Bruker ApexIII suite 2021. All structures were solved with SHELXT^156^ and refined using the OLEX 2 software package.^157^ Strongly disordered solvent molecules were been removed using the SQUEEZE operation.^158^ All nonhydrogen atoms were refined anisotropically, and hydrogen atoms were included at the geometrically calculated positions and refined using a riding model. For further crystallographic details, see Tables S2 and S3 in the Supporting Information.

## Supporting Information Available

NMR spectra, IR spectra, UV–vis spectra, crystallographic details, cyclic voltammograms and computational details (PDF)

## Accession Codes

CCDC 2079467, 2079465, 2079472, 2079471, 2079474, 2079469, 2079473 and 2080686 contain the supplementary crystallographic data for this paper. These data can be obtained free of charge via www.ccdc.cam.ac.uk/data_request/cif, or by emailing data_request@ccdc.cam.ac.uk, or by contacting The Cambridge Crystallographic Data Centre, 12 Union Road, Cambridge CB2 1EZ, UK; fax: + 441223 336033.
