# Supplementary material for: Mesoionic Carbenes in Low- to High-Valent Vanadium Chemistry
Source: Inorg Chem. 2021 Sep 30;60(20):15421–34. doi: 10.1021/acs.inorgchem.1c02087 (PMC8527456; doi:10.1021/acs.inorgchem.1c02087)
Supplement: Supplementary file 1 — ic1c02087_si_001.pdf [file ic1c02087_si_001.pdf]

Mesoionic Carbenes in Low- to High-Valent Vanadium Chemistry

Florian R. Neururer,[a] Shenyu Liu,[b] Daniel Leitner,[a] Marc Baltrun,[b] Katherine R. Fisher,[c] Holger Kopacka,[a] Klaus Wurst,[a] Lena J. Daumann,[c] Dominik Munz*[d] and Stephan Hohloch*[a].

[a] University of Innsbruck, Institute of Inorganic, General and Theoretical Chemistry, Innrain 80-82, 6020 Innsbruck, Austria, E-Mail: Stephan.Hohloch@uibk.ac.at

[b] University of Paderborn, Faculty of Science, Department of Chemistry, Warburger Straße 100, 33098 Paderborn, Germany

[c] Ludwigs-Maximilians-University Munich, Department Chemie, Butenandtstraße 5-13 Haus D, 81377 Munich, Germany

[d] Saarland University, Fakultät NT, Inorganic Chemistry: Coordination Chemistry, Campus C4.1, 66123 Saarbrücken, Germany, E-Mail: Dominik.Munz@uni-saarland.de

Supporting Information

Table of contents

1. NMR spectra 2
2. IR spectroscopy 50
3. UV-Vis spectra 62
4. Electrochemistry (Cyclic Voltammetry) 70
5. Quantum Chemical Calculations 72
6. EPR Spectroscopy 104
7. Crystallographic details 105
8. Literature 116

1. NMR spectra

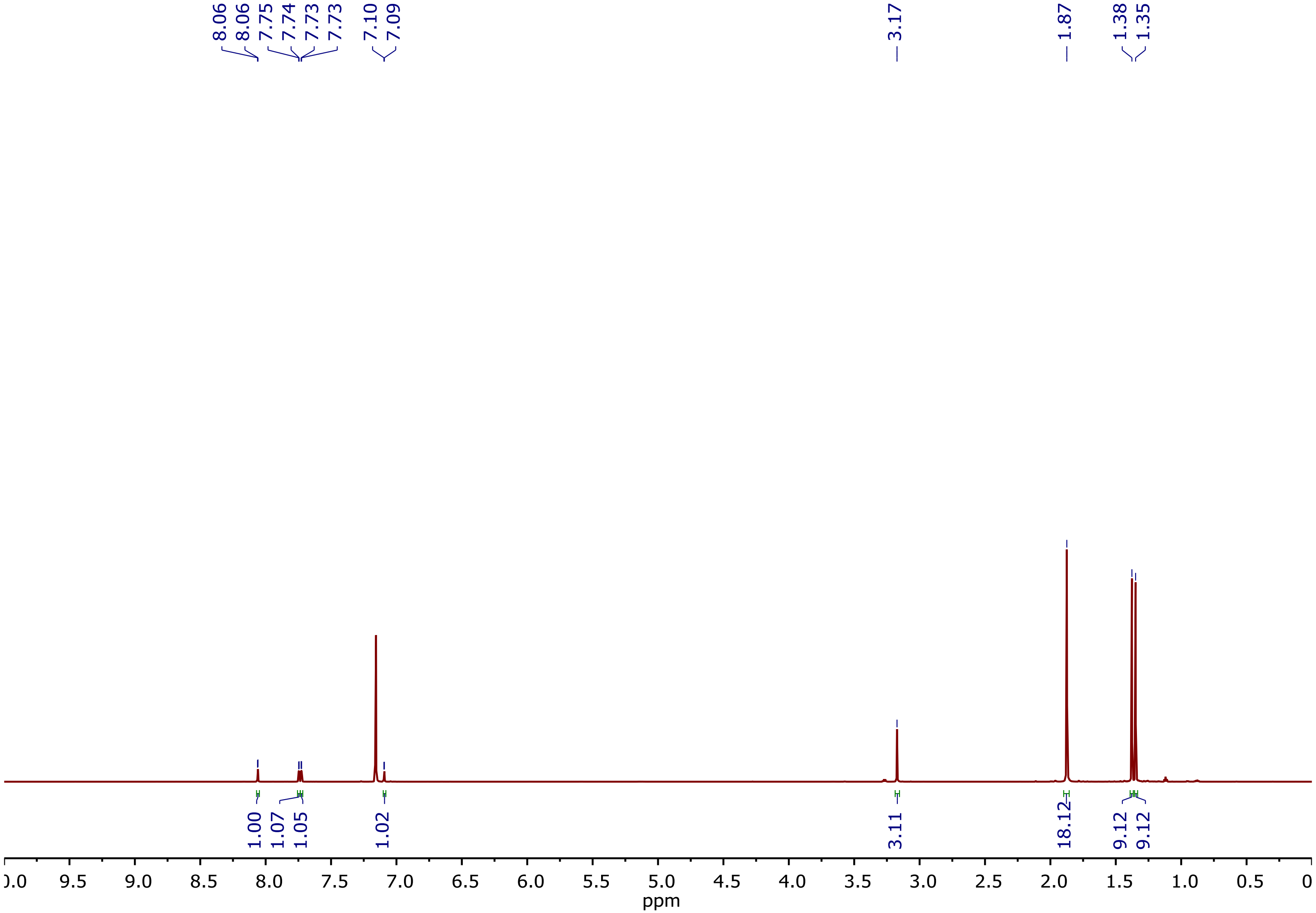

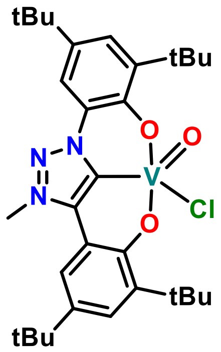

Figure S 1: ^{1}H NMR of **1** in C_6D_6 at 298K.

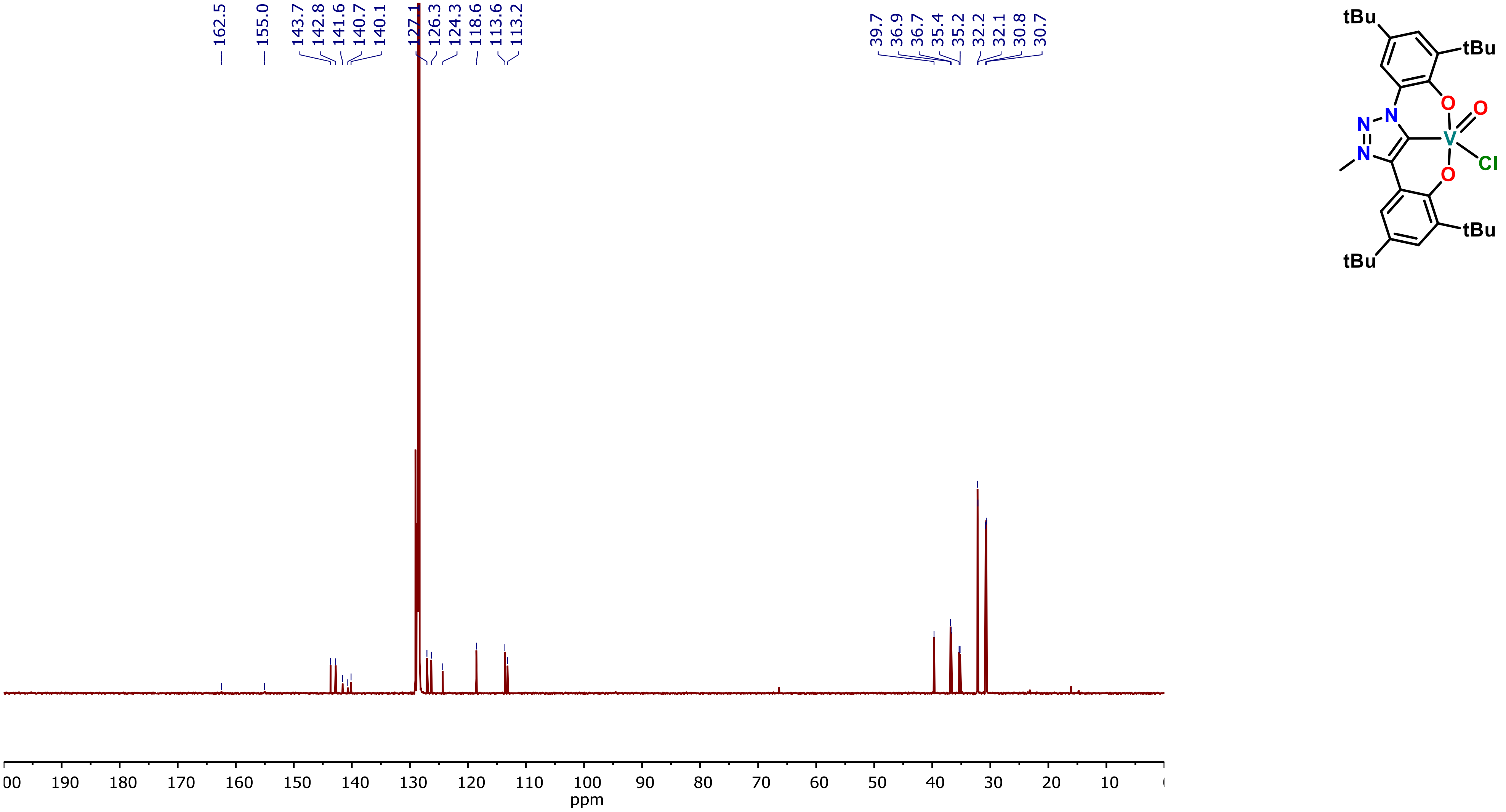

Figure S 2: ^{13}C NMR of **1** in C_6D_6 at 298K.

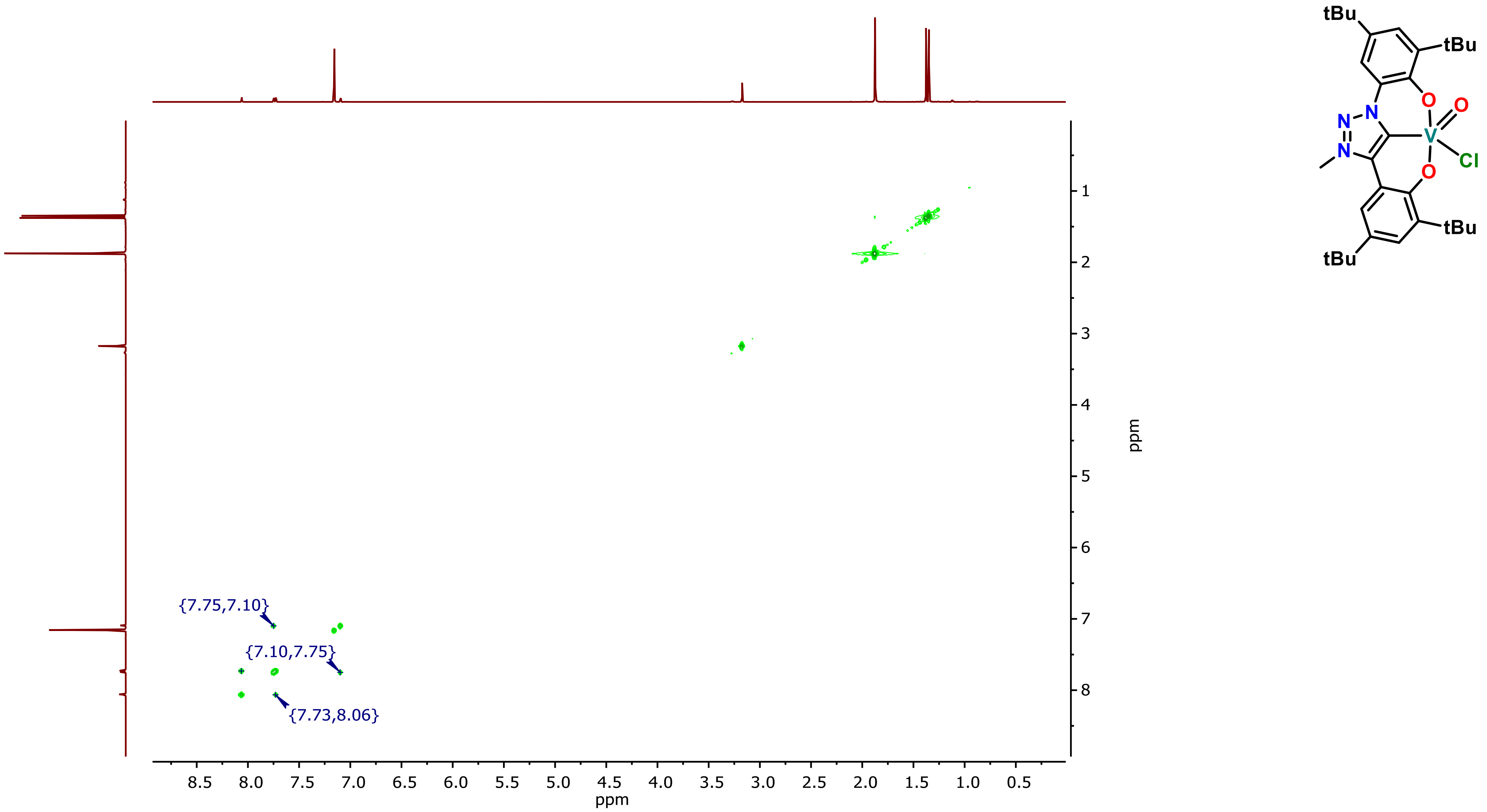

Figure S 3: ^{1}H-^{1}H COSY of **1** in C_6D_6 at 298K.

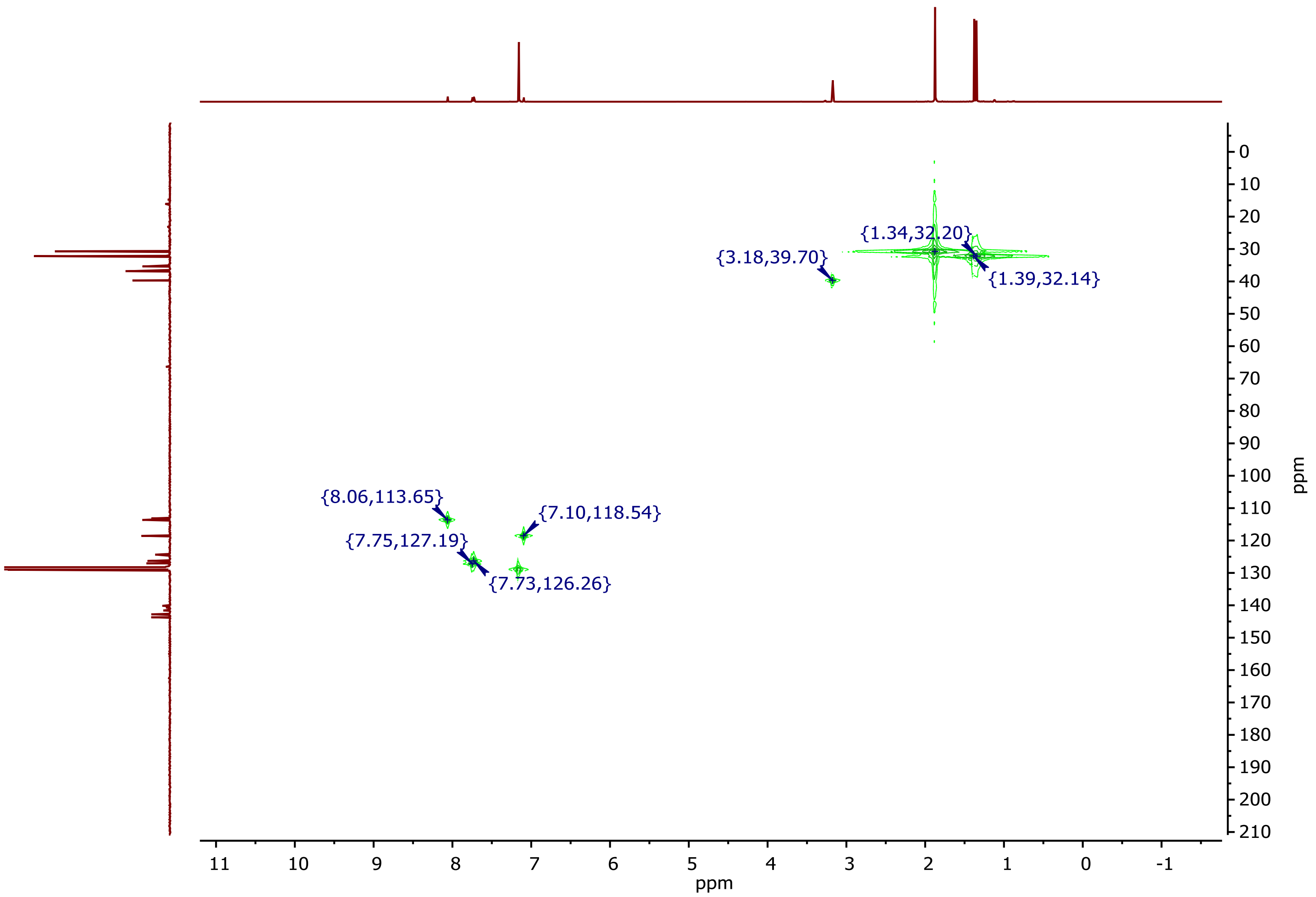

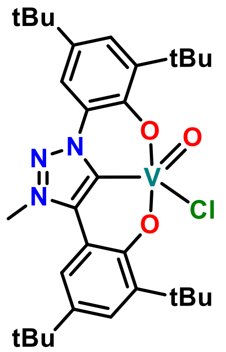

Figure S 4: ^{1}H-^{13}C HSQC of **1** in C_6D_6 at 298K.

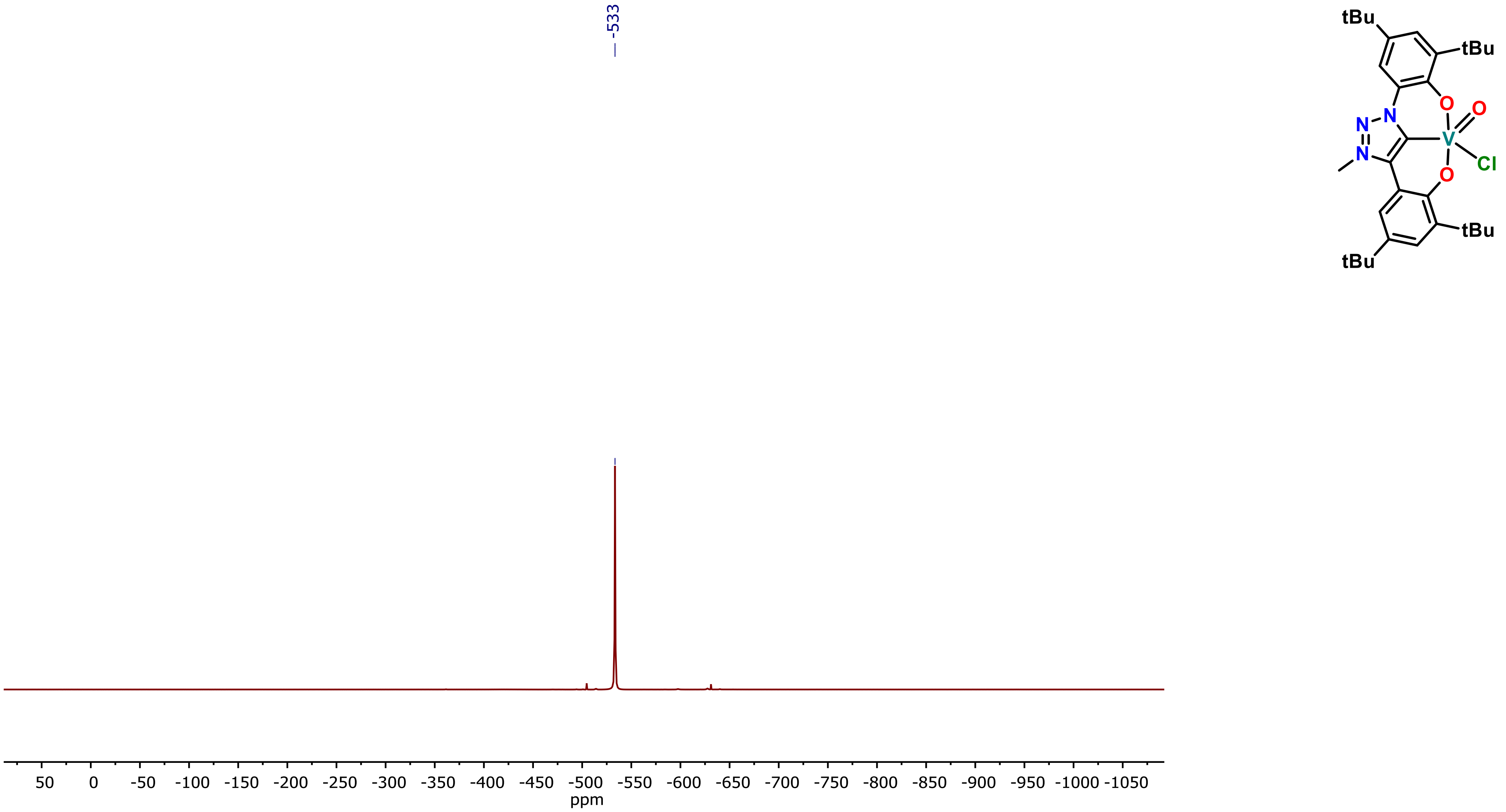

Figure S 5: ^{51}V NMR of **1** in C_6D_6 at 298K.

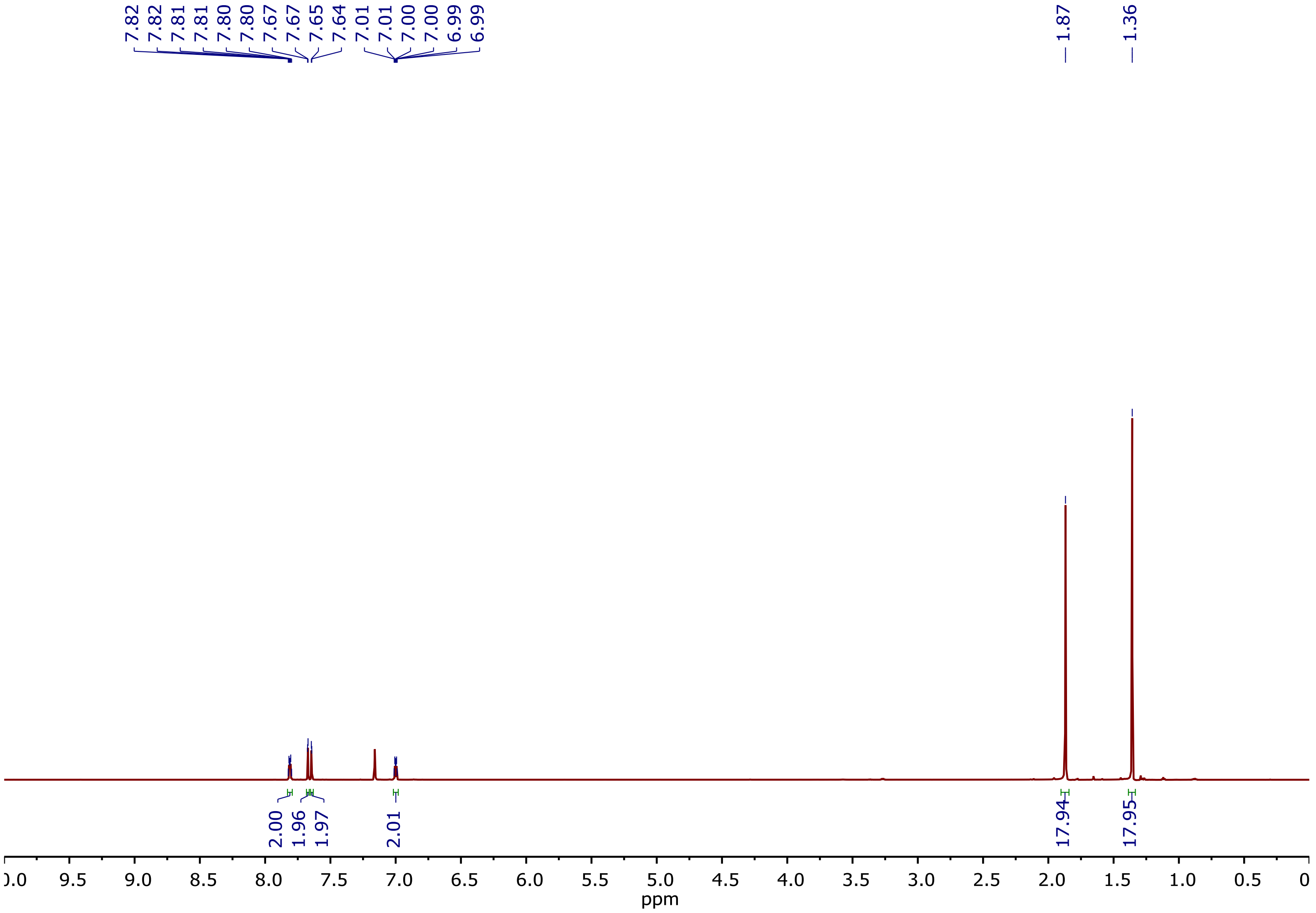

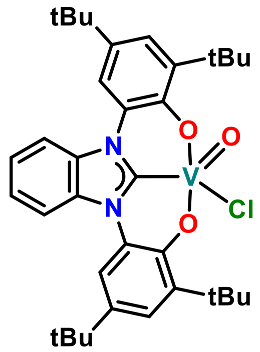

Figure S 6: ^{1}H NMR of **2** in C_6D_6 at 298K.

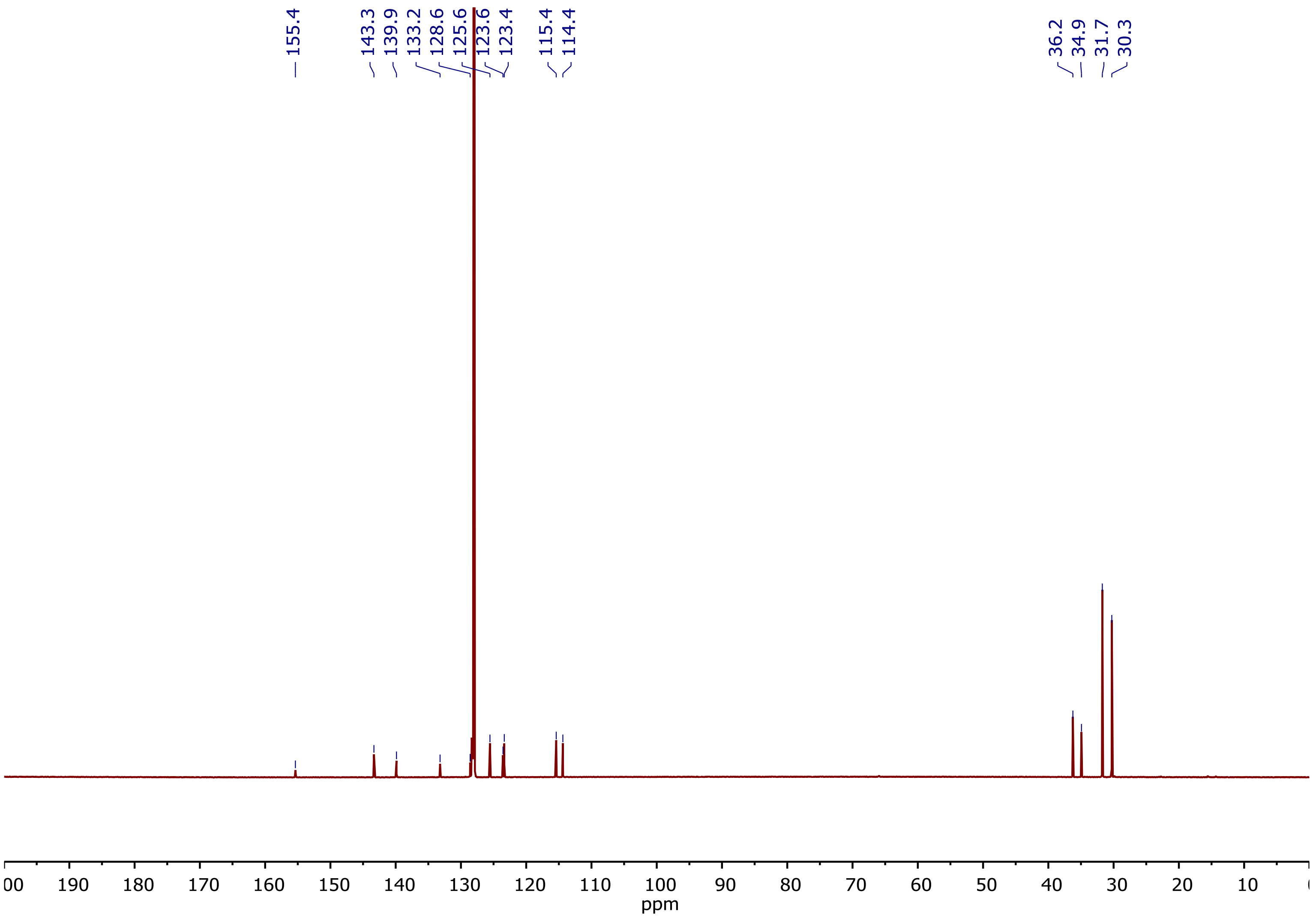

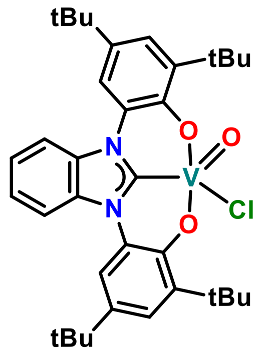

Figure S 7: ^{13}C NMR of **2** in C_6D_6 at 298K.

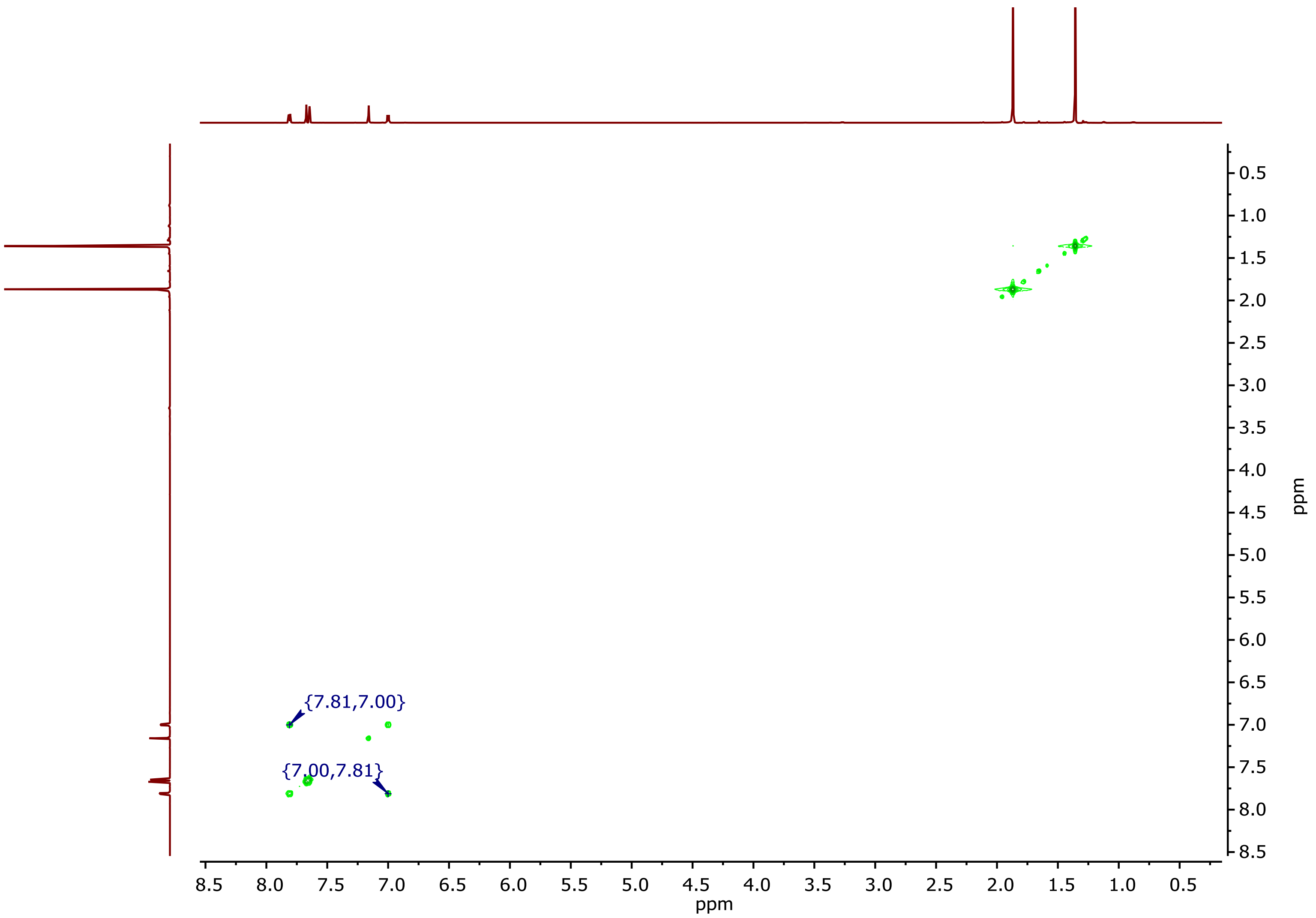

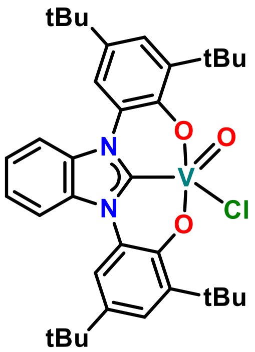

Figure S 8: ^1H-^1H COSY of **2** in C_6D_6 at 298K.

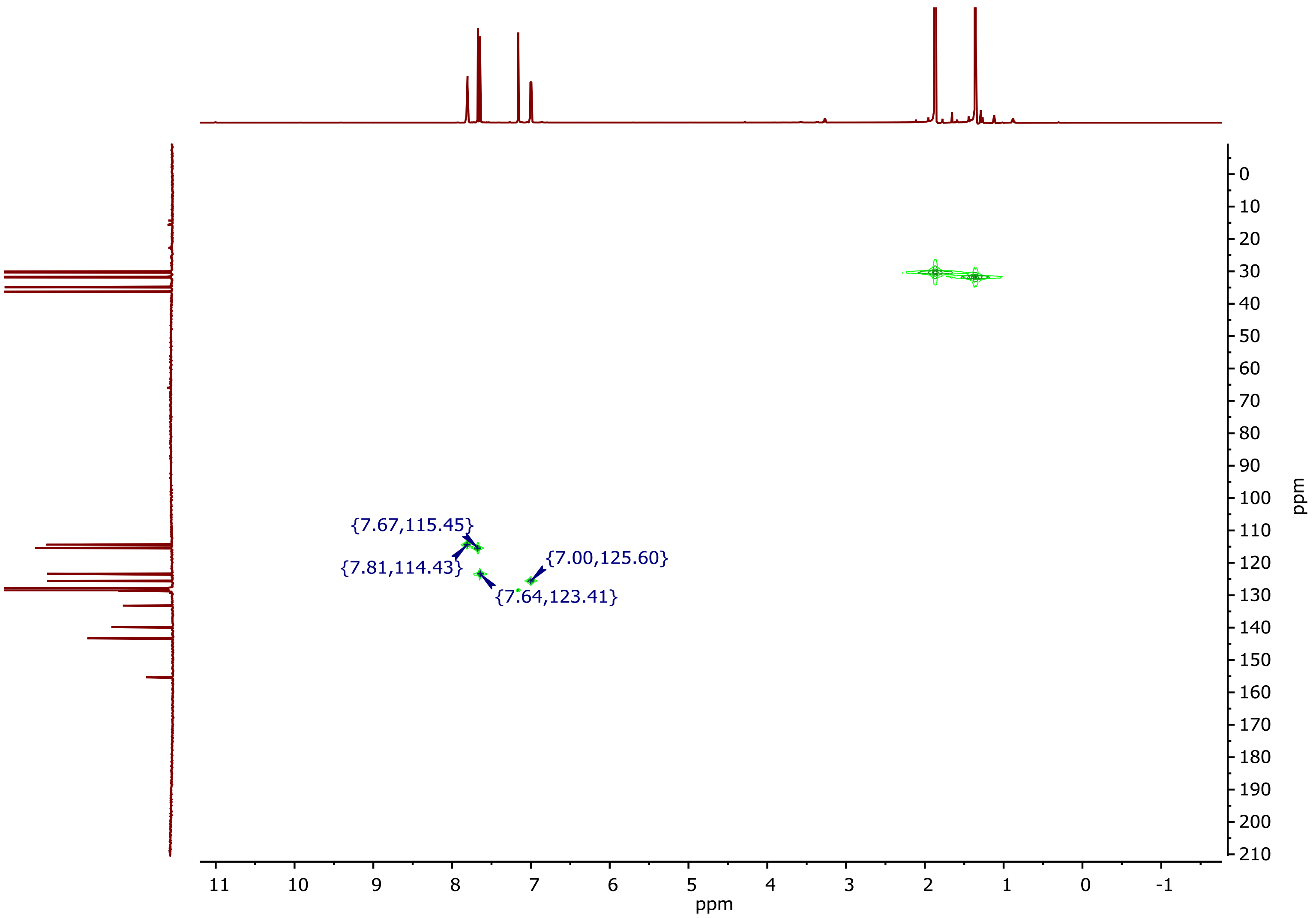

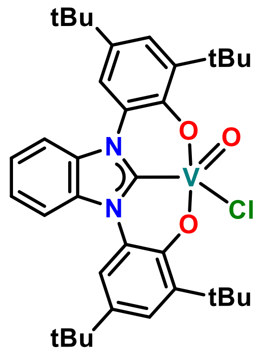

Figure S 9: ^{1}H-^{13}C HSQC of **2** in C_6D_6 at 298K.

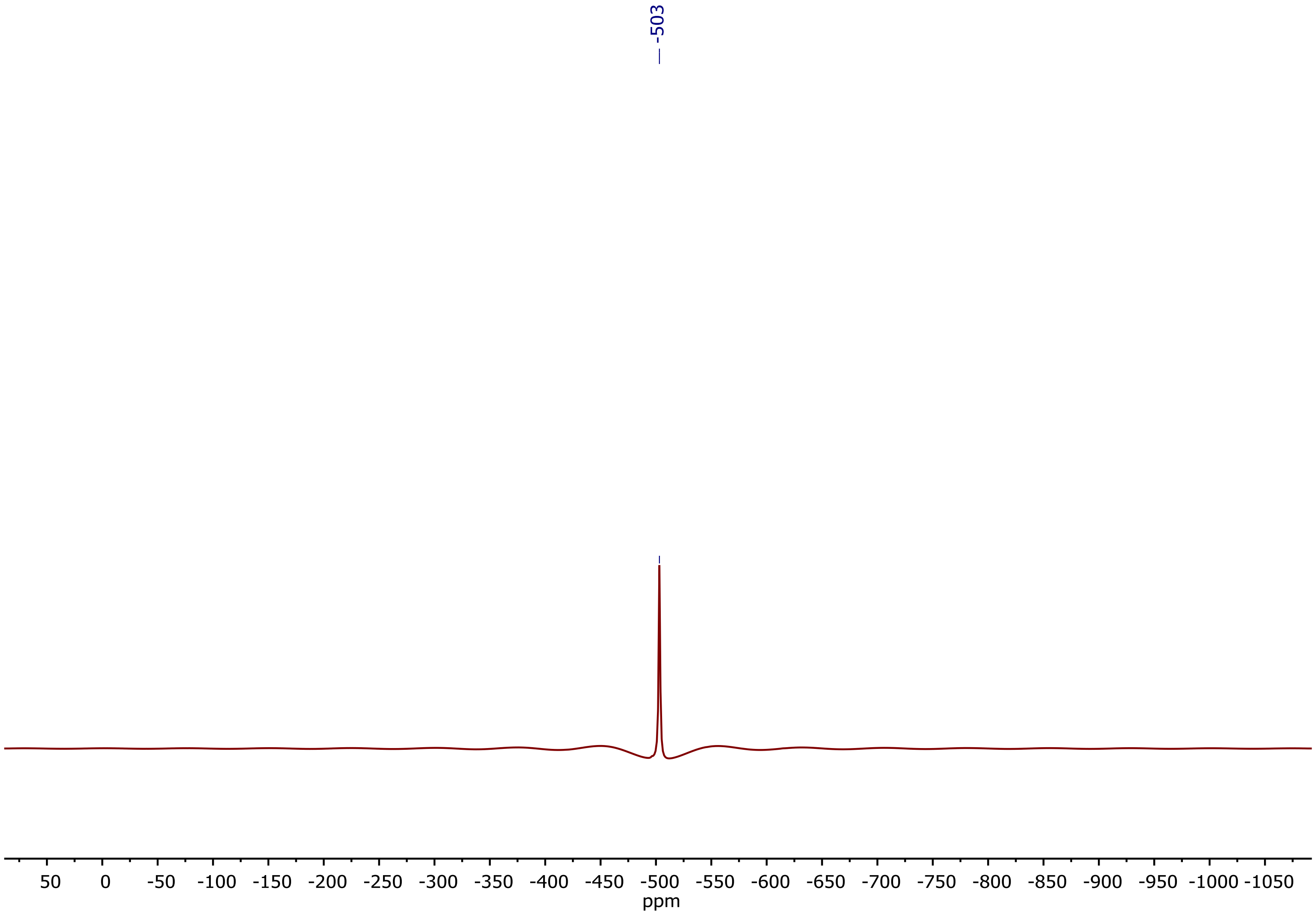

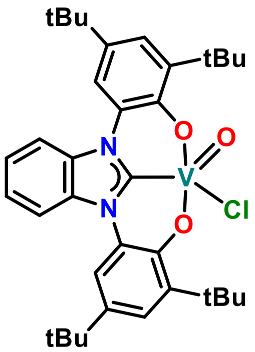

Figure S 10: ^{51}V NMR of **2** in C_6D_6 at 298K.

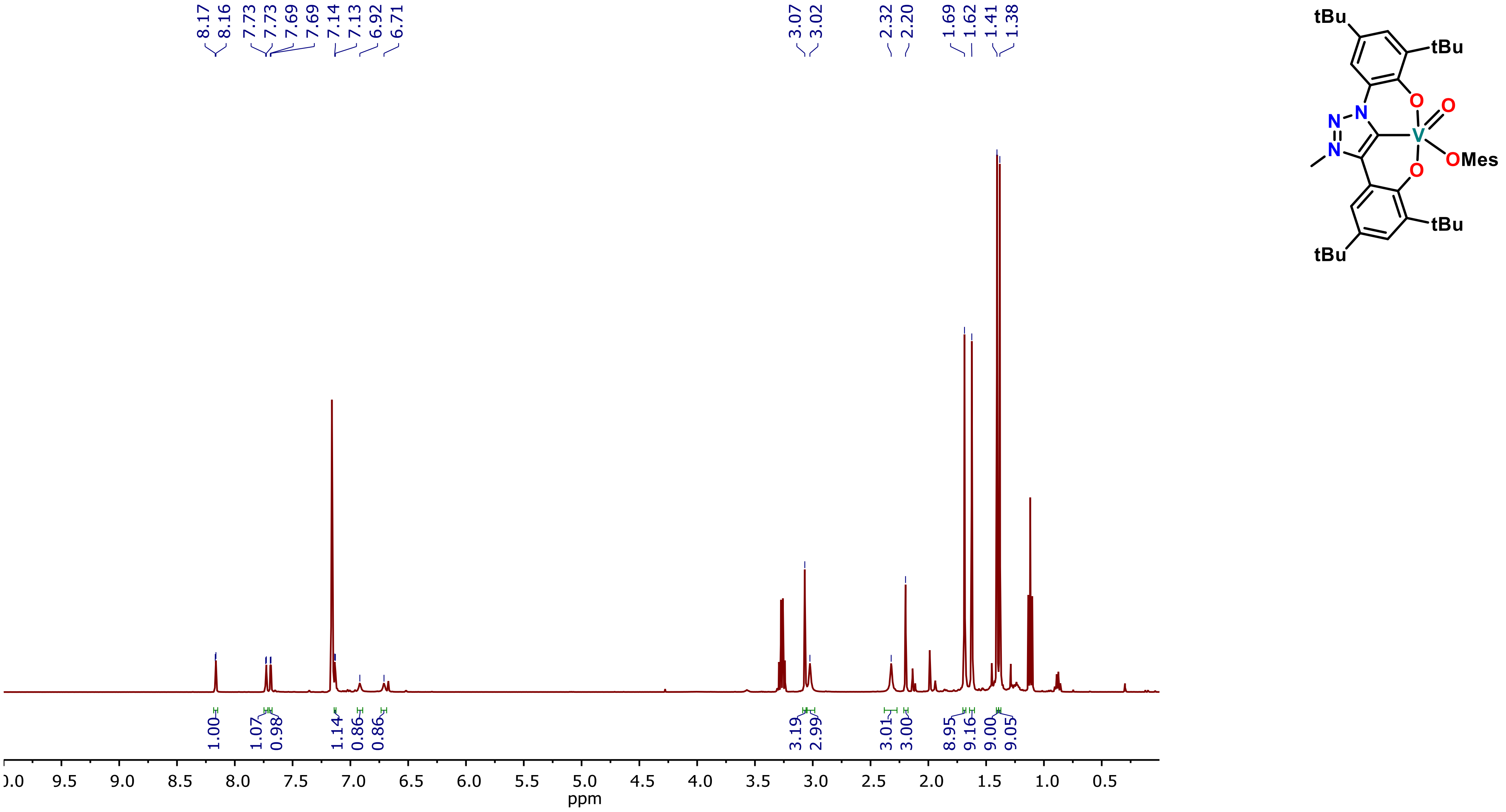

Figure S 11: ^{1}H NMR of **3** in C_6D_6. Peaks at 6.67 2.13 and 1.98 ppm results from residual Mesitol.

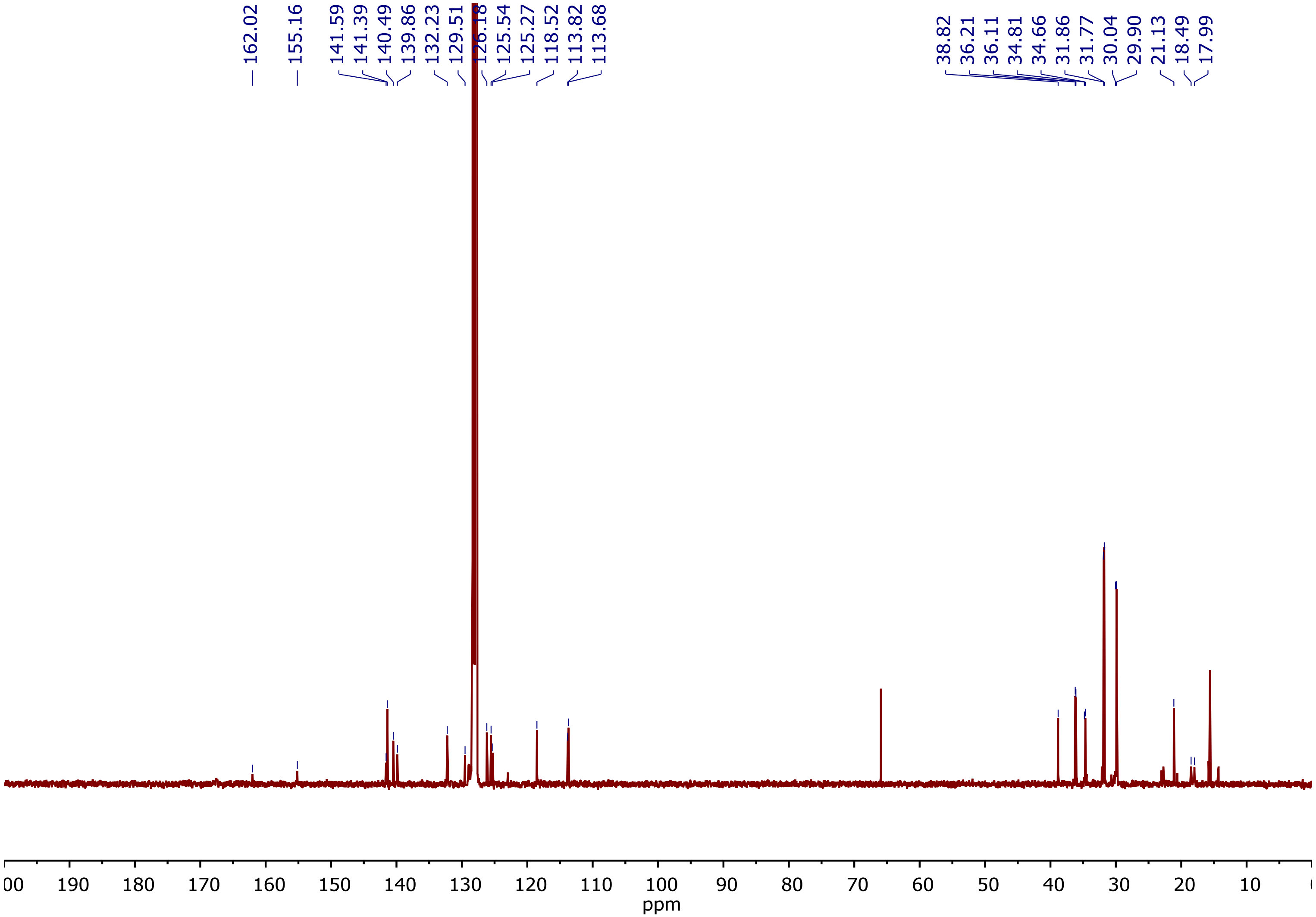

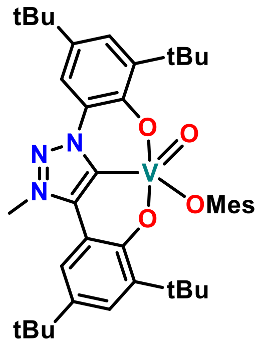

Figure S 12: ^{13}C NMR of **3** in C_6D_6

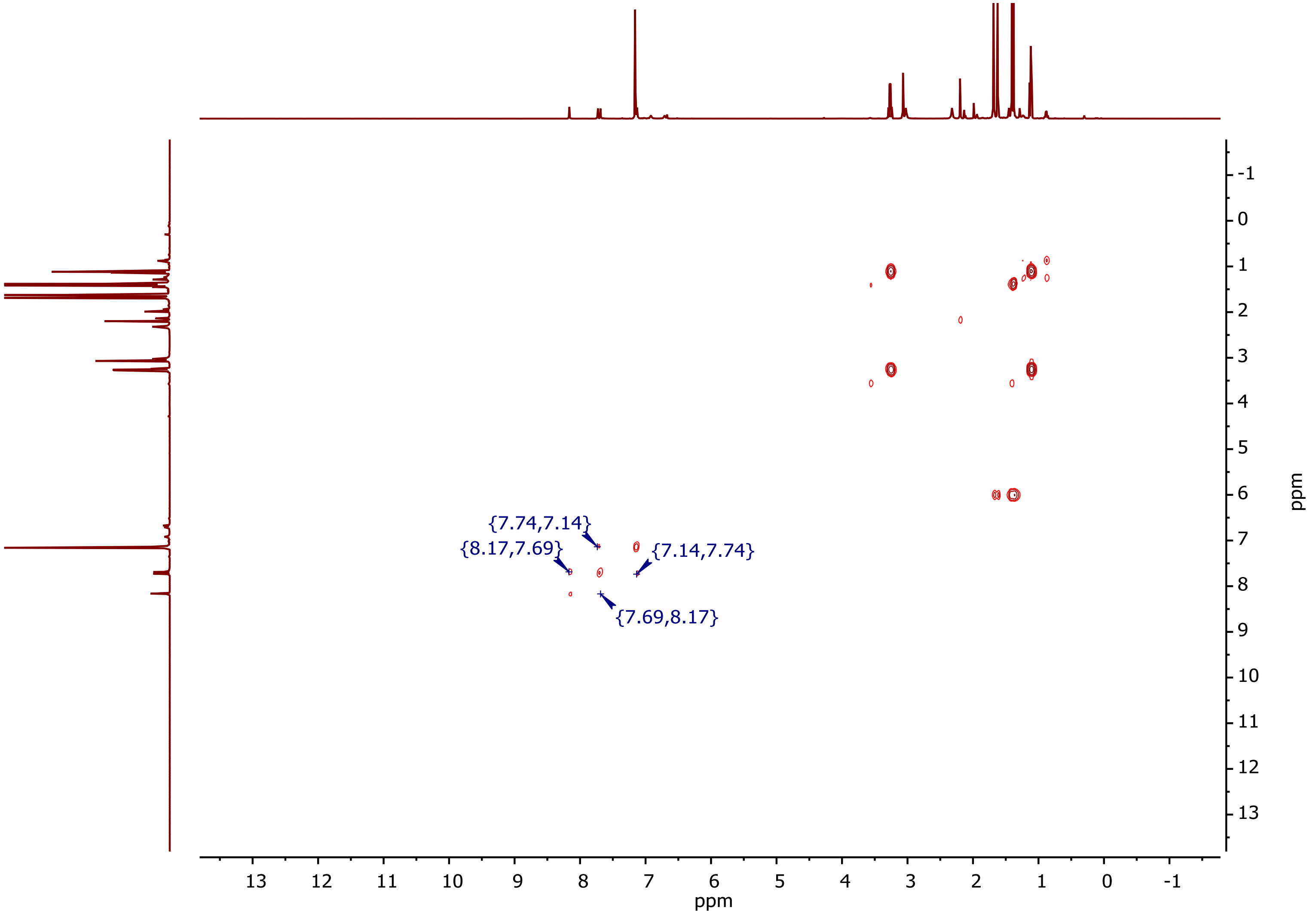

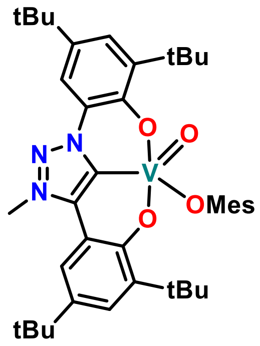

Figure S 13: ^{1}H ^{1}H COSY of **3** in C_6D_6

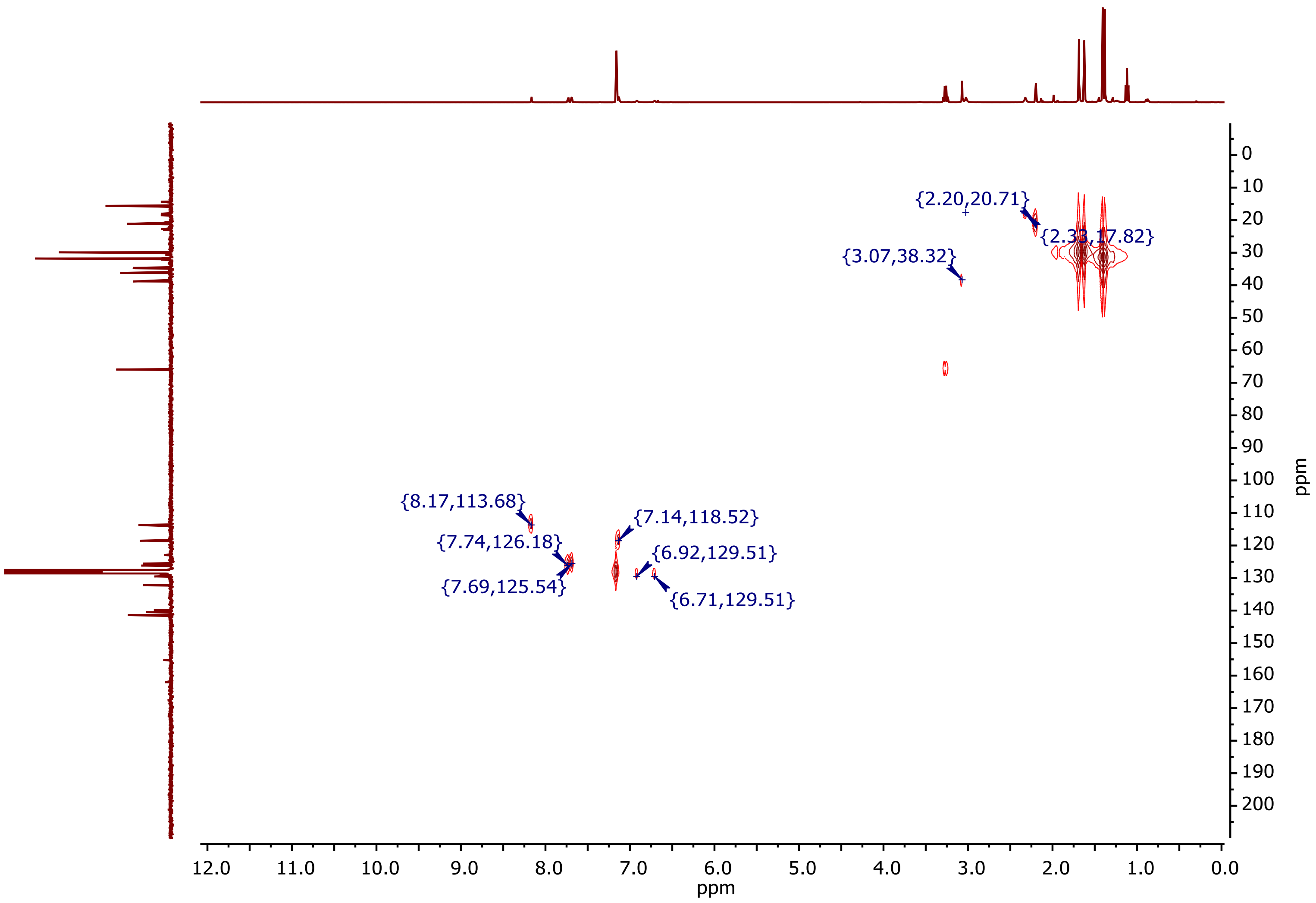

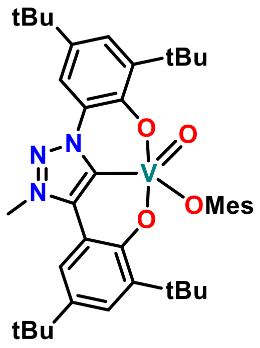

Figure S 14: ^{1}H ^{13}C HSQC of **3** in C_6D_6

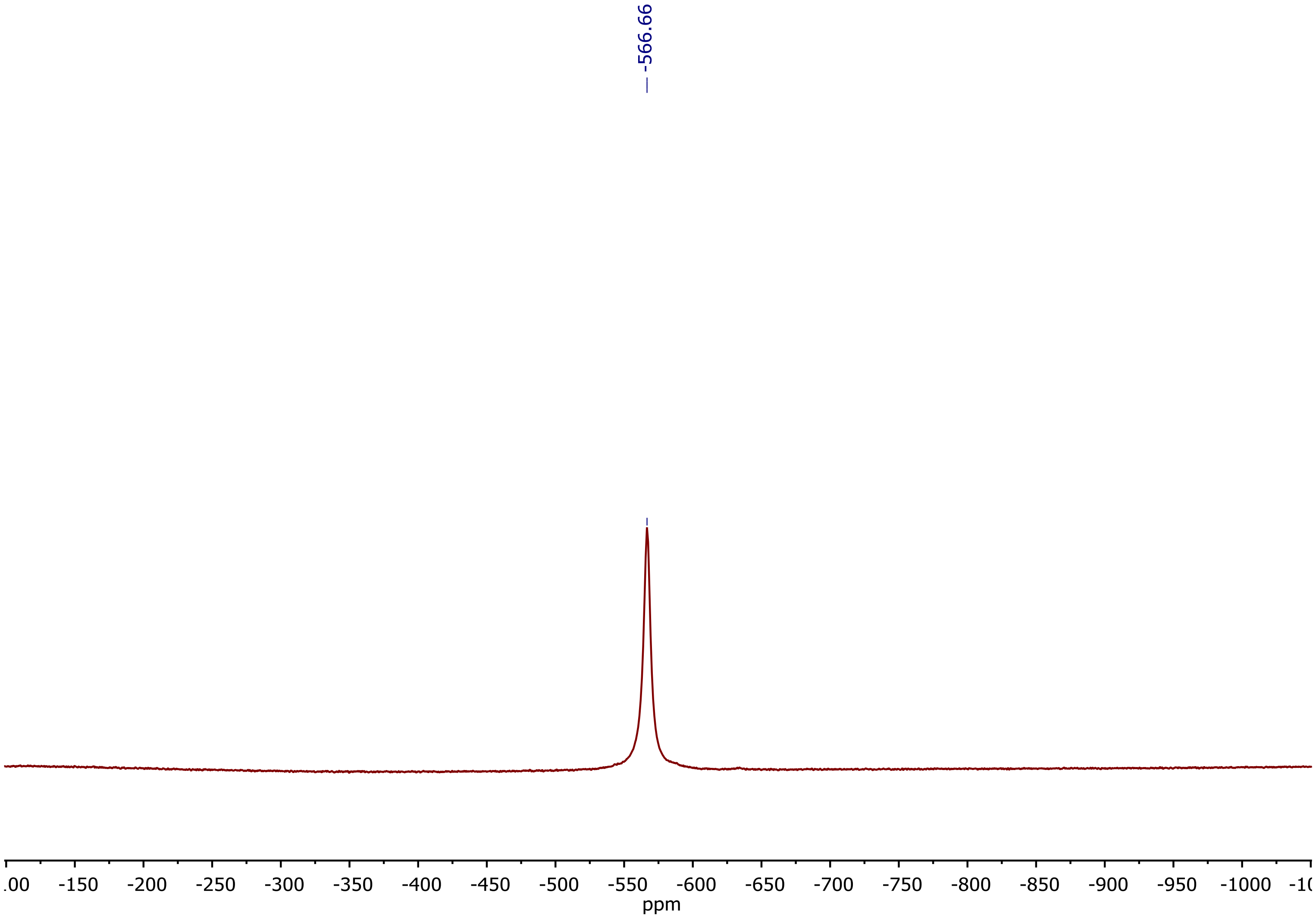

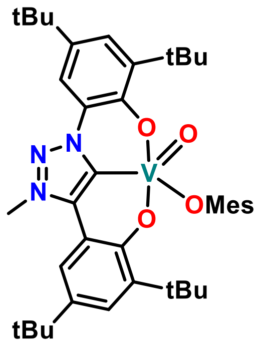

Figure S 15: ^{51}V NMR of **3** in C_6D_6

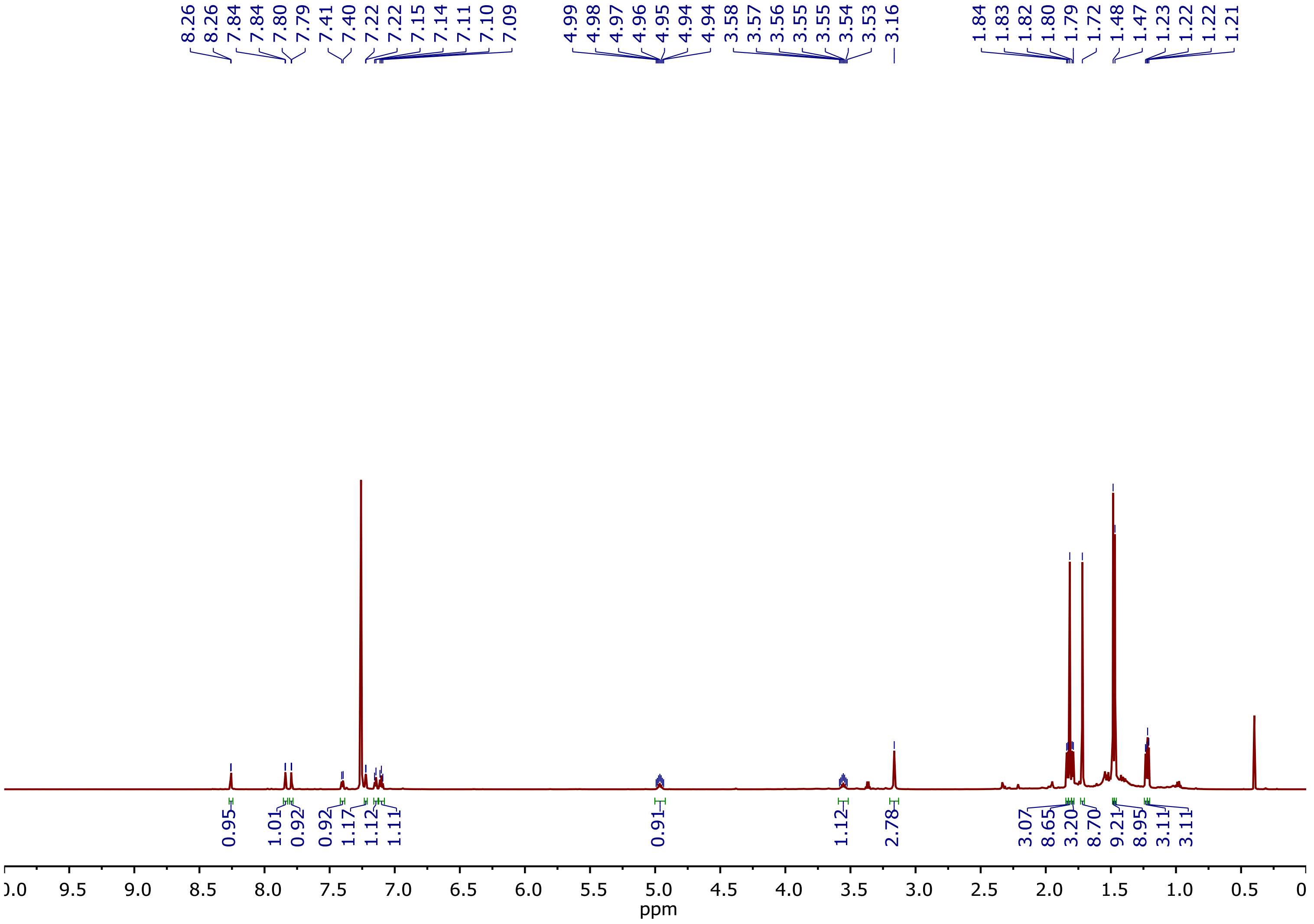

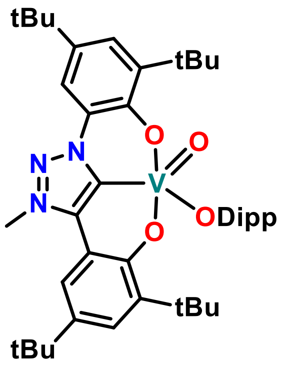

Figure S 16: ^{1}H NMR of **4** in C_6D_6 at 298K.

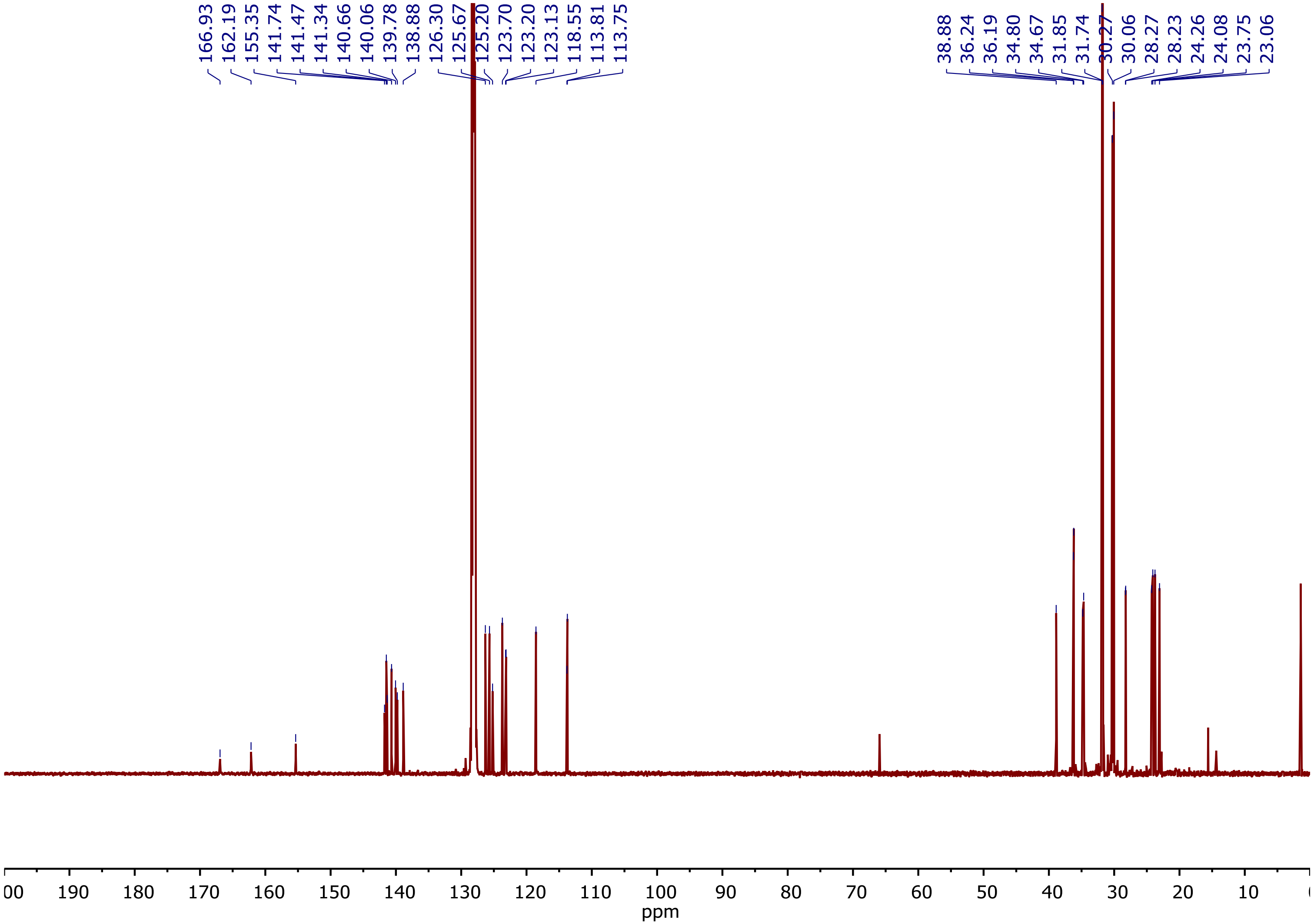

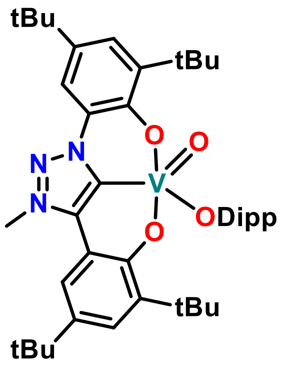

Figure S 17: ^{13}C NMR of **4** in C_6D_6 at 298K.

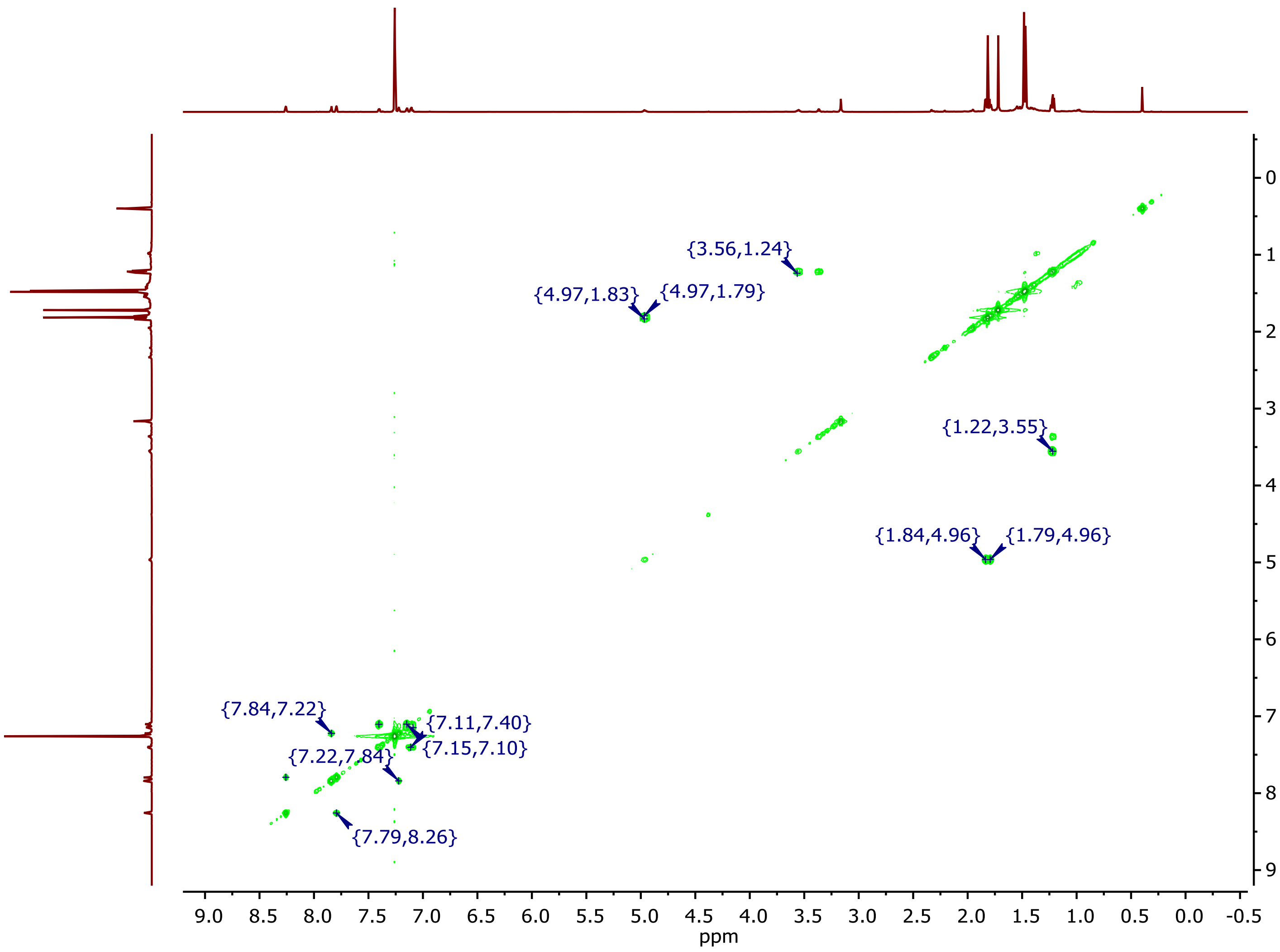

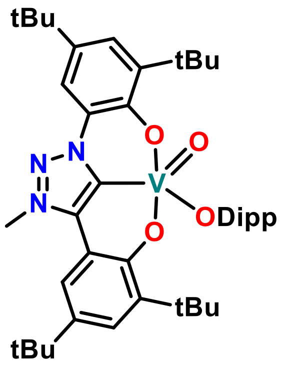

Figure S 18: ^{1}H-^{1}H COSY of **4** in C_6D_6 at 298 K

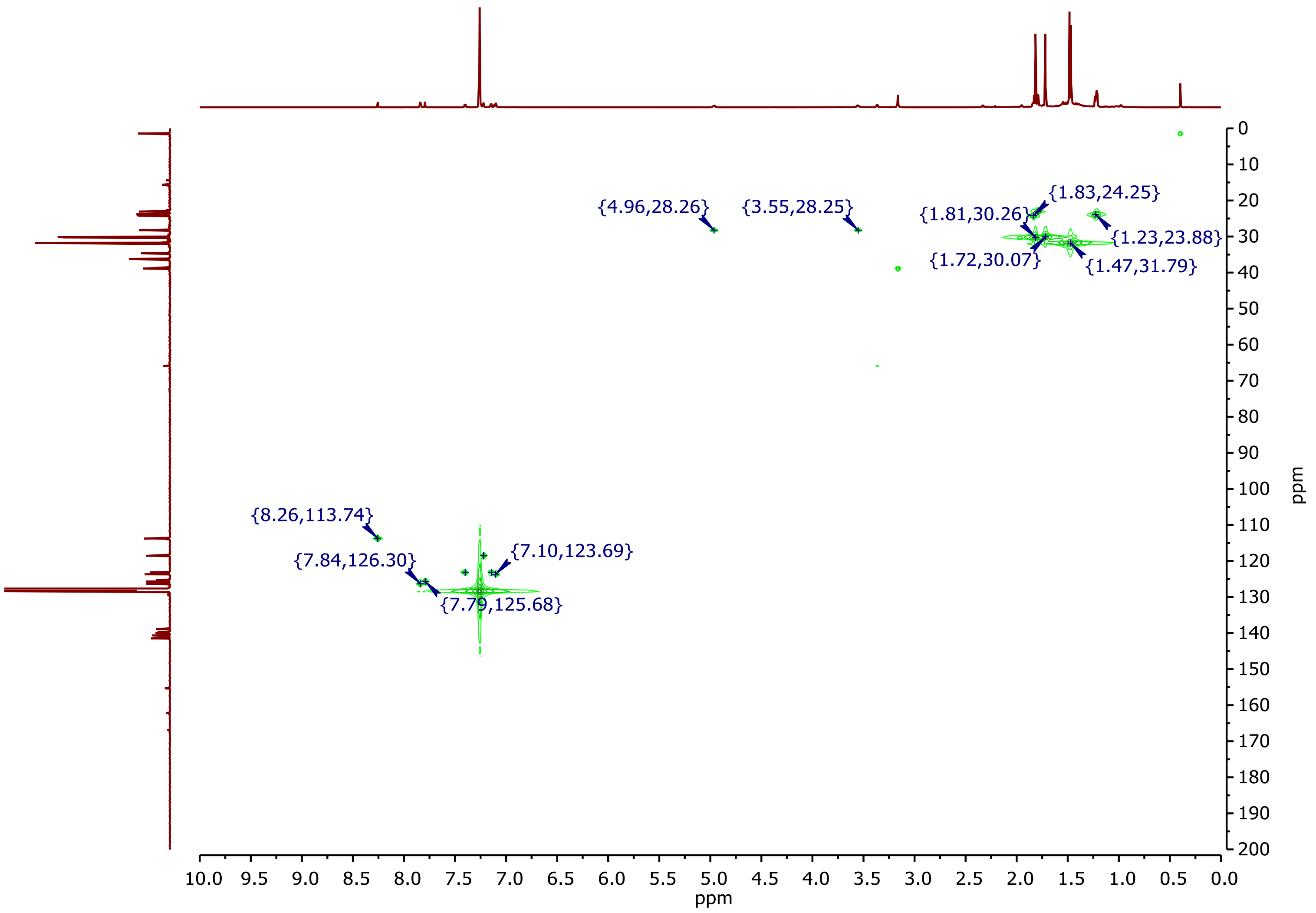

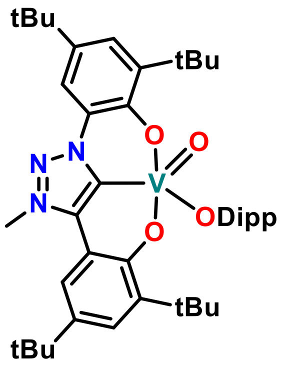

Figure S 19: ^{1}H-^{13}C HSQC of **4** in C_6D_6 at 298 K

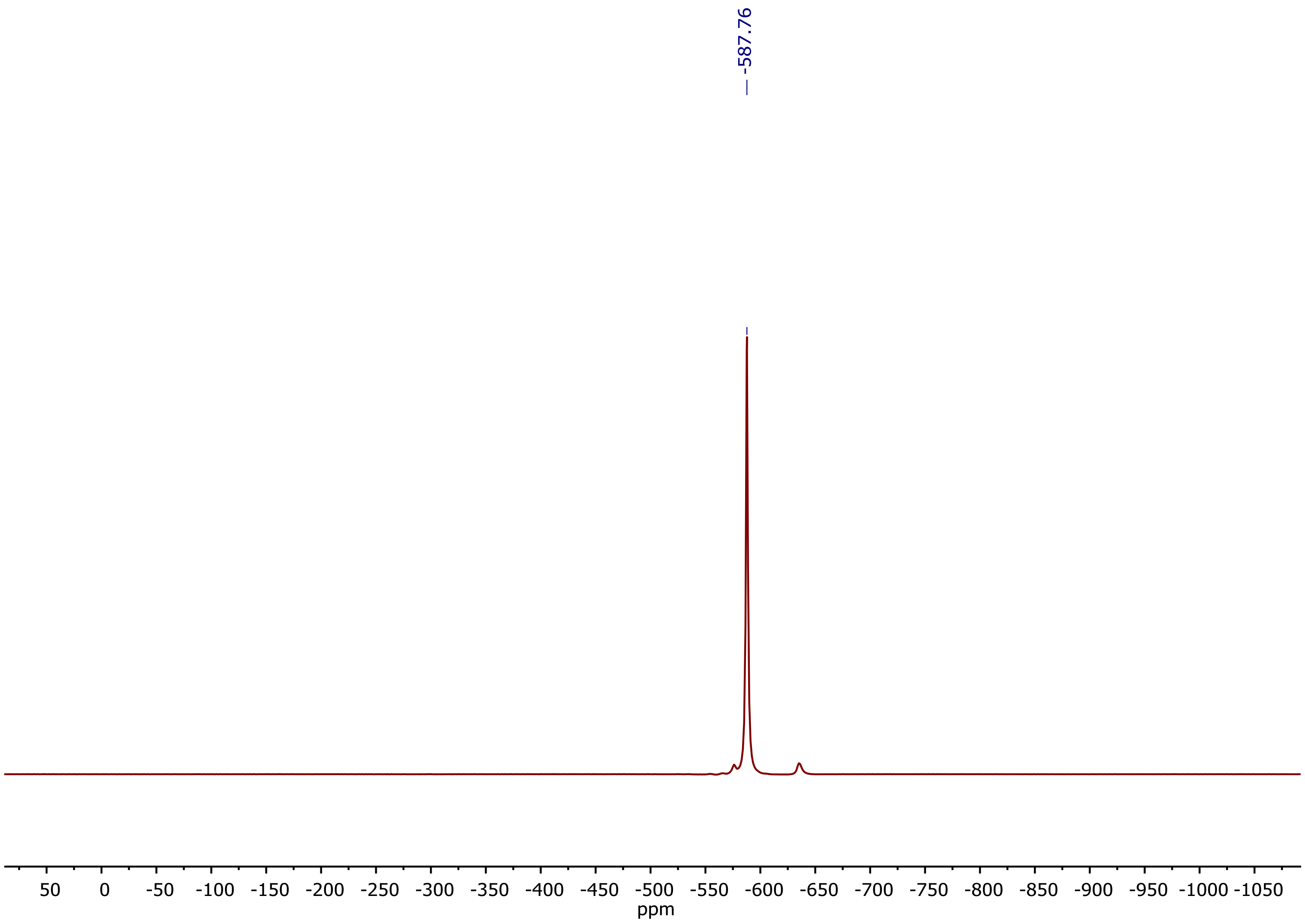

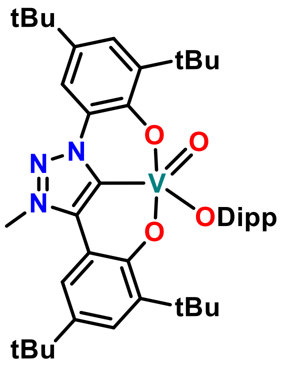

Figure S 20: ^{51}V NMR of **4** in C_6D_6 at 298 K

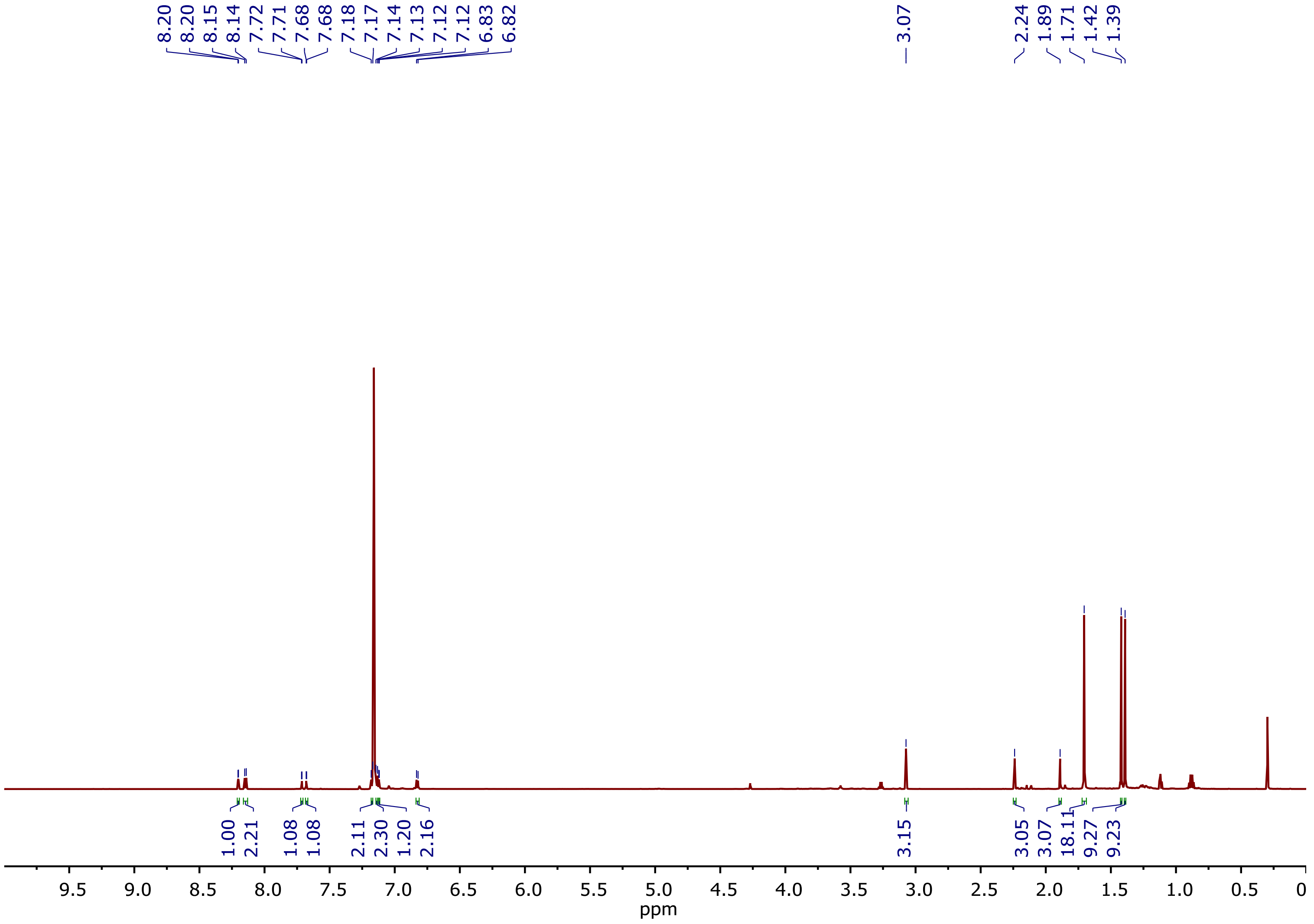

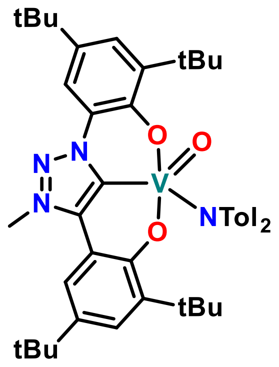

Figure S 21: ^{1}H-NMR of **5** in C_6D_6 at 298 K

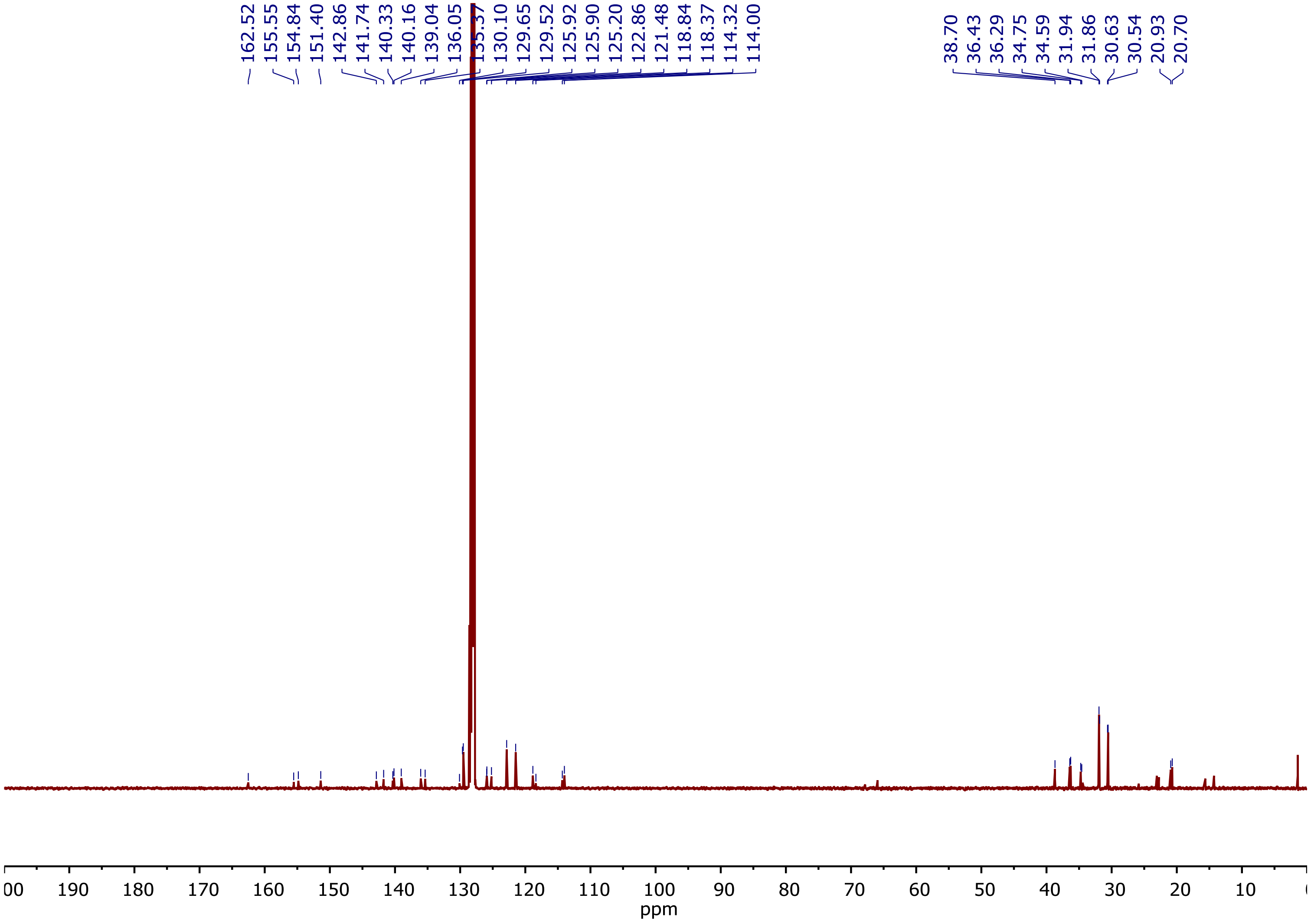

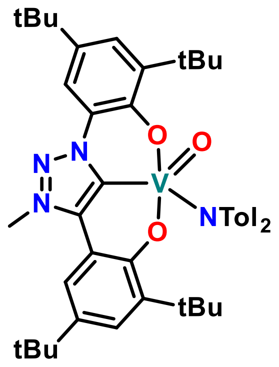

Figure S 22: ^{13}C NMR of **5** in C_6D_6 at 298 K

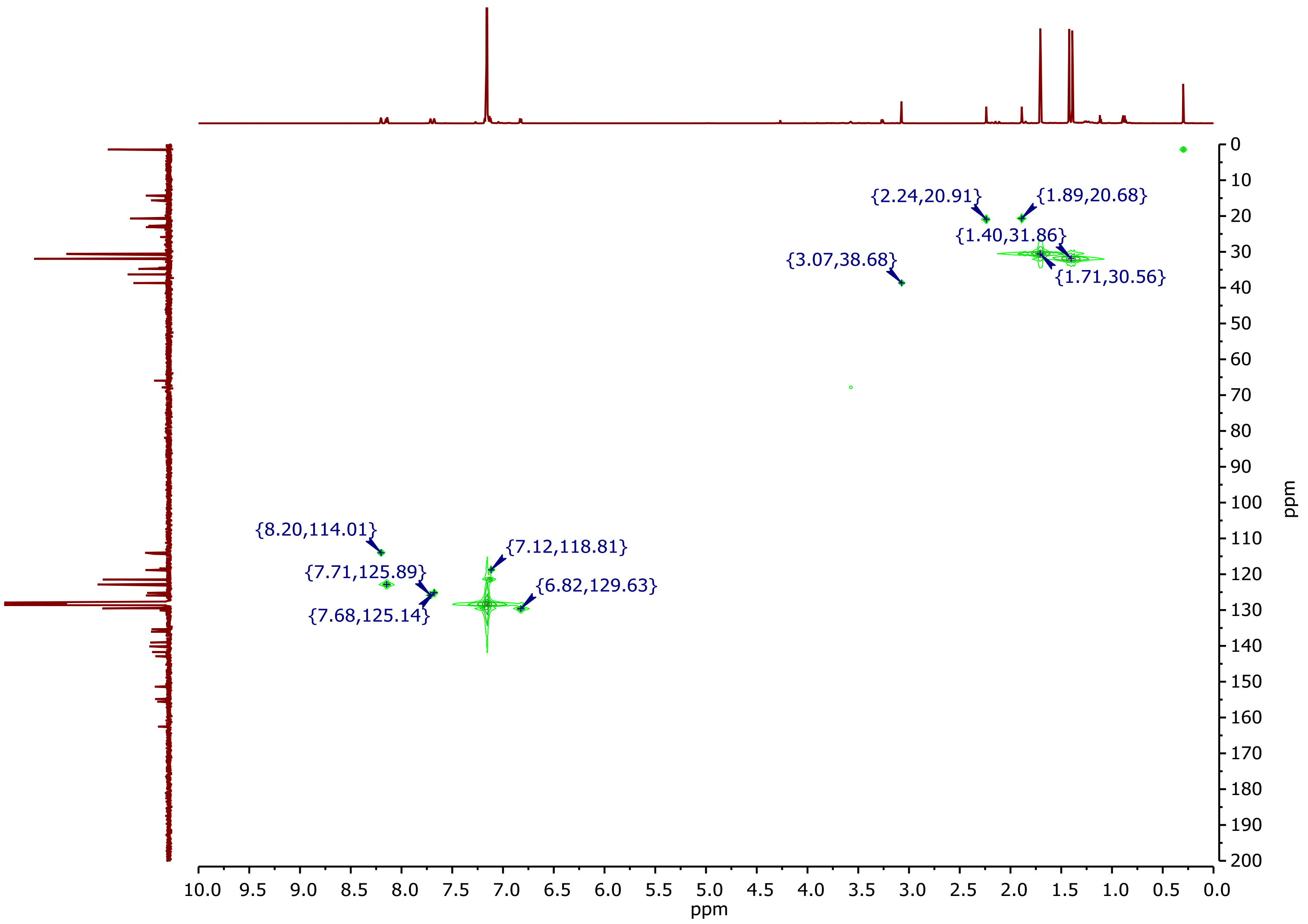

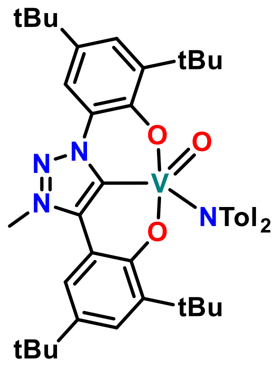

Figure S 23: ^{1}H-^{1}H COSY of **5** in C_6D_6 at 298 K

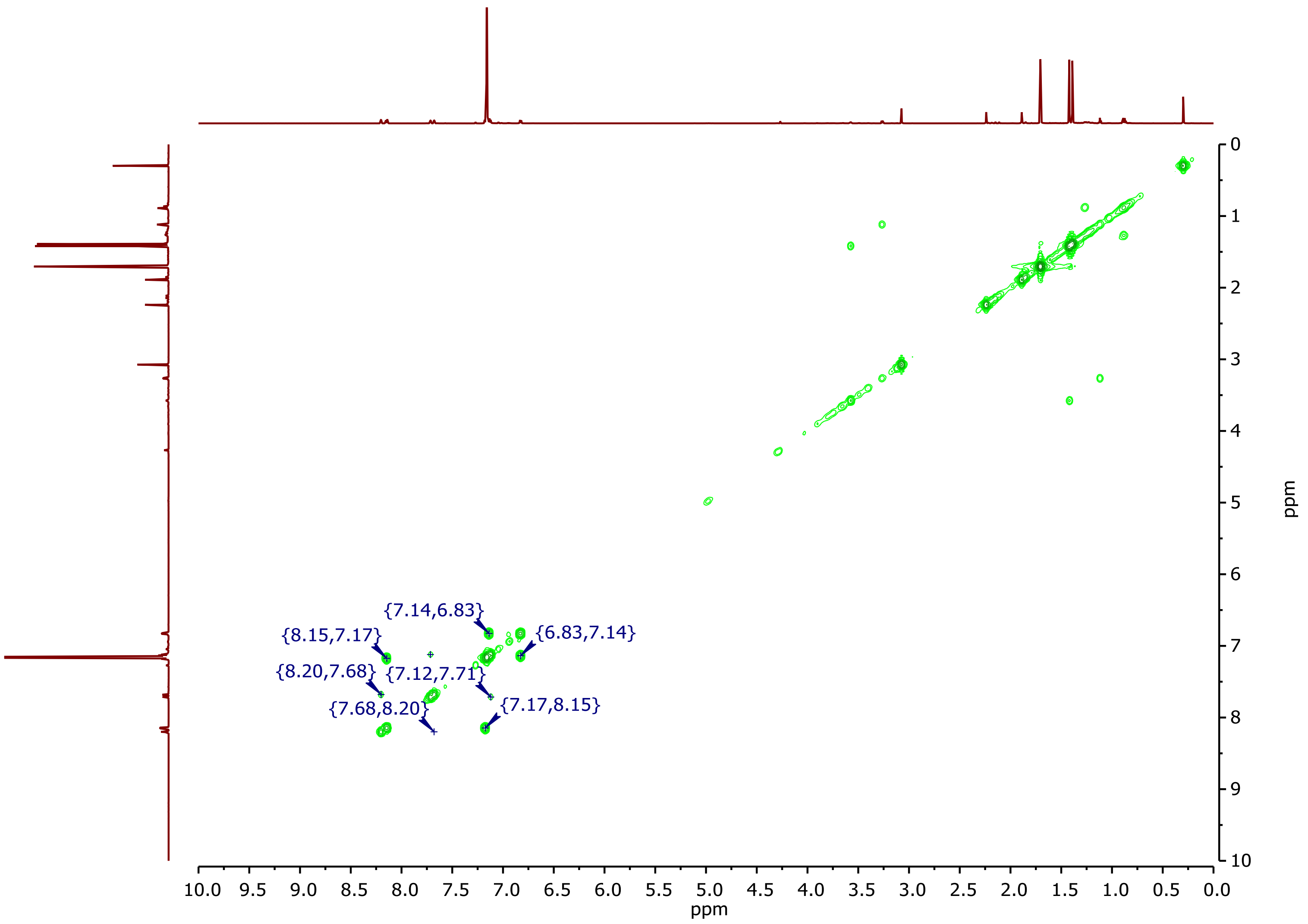

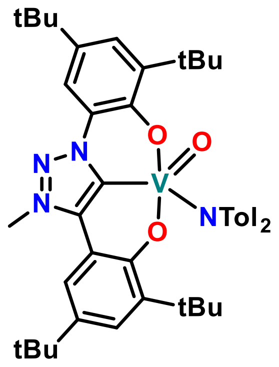

Figure S 24: ^{1}H-^{13}C HSQC of **5** in C_6D_6 at 298 K

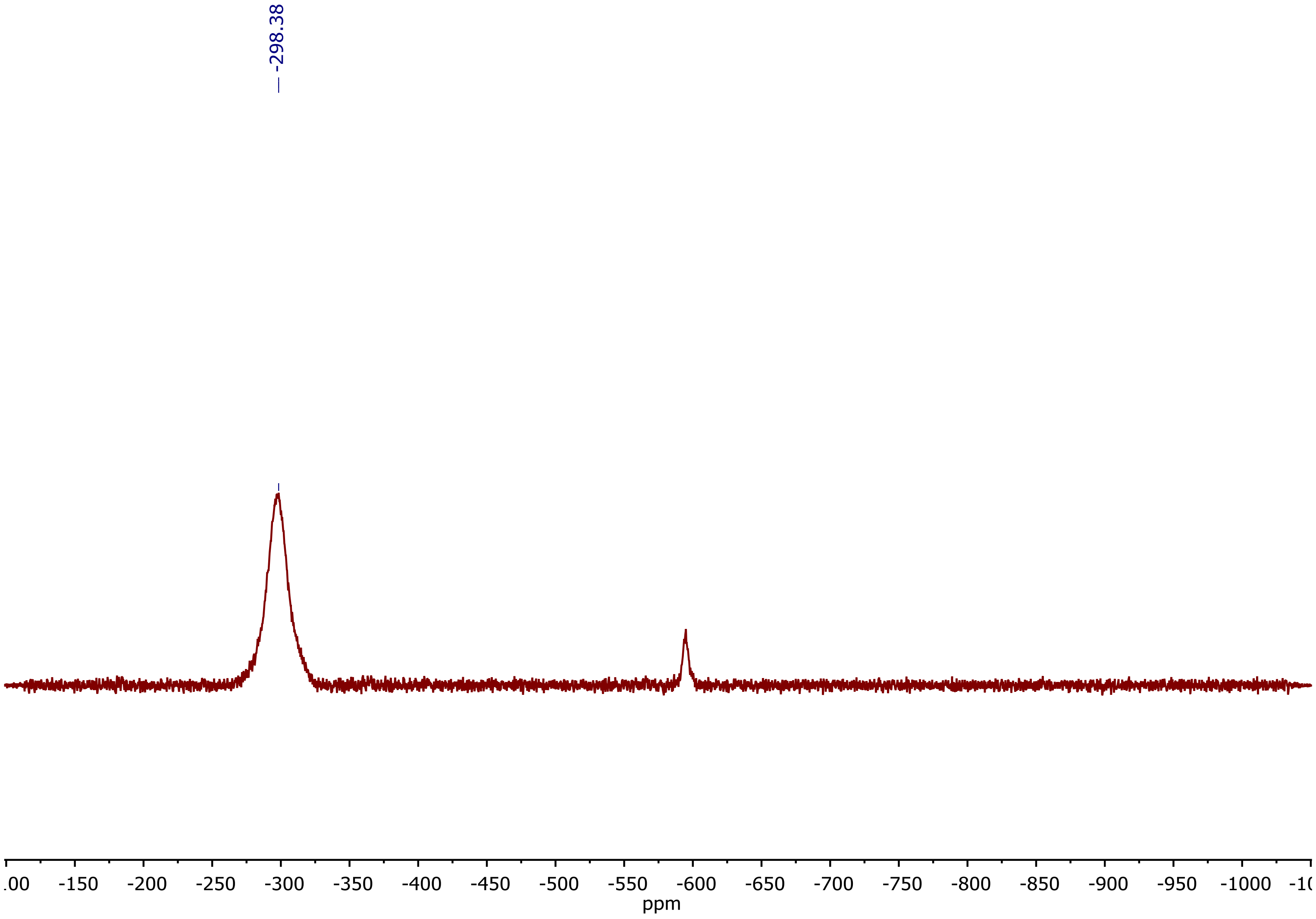

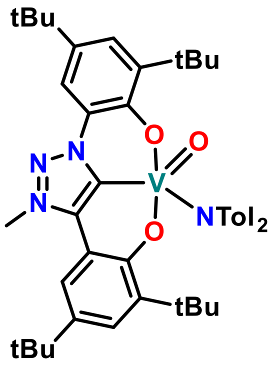

Figure S 25: ^{51}V NMR of **5** in C_6D_6. The peak at 594 ppm belongs to an unknown impurity.

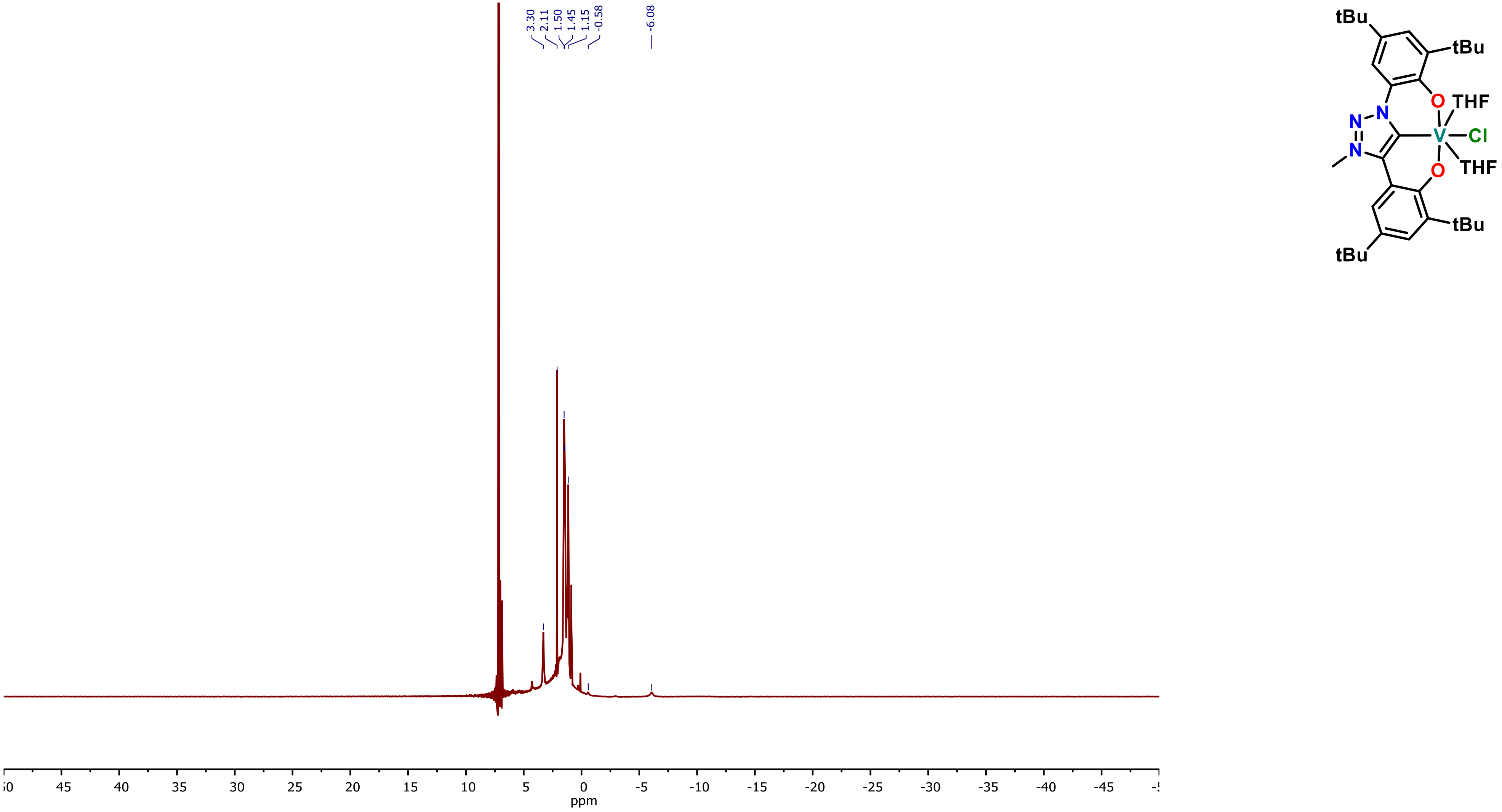

Figure S 26: ^{1}H NMR of **6** in C_6D_6 at 298 K.

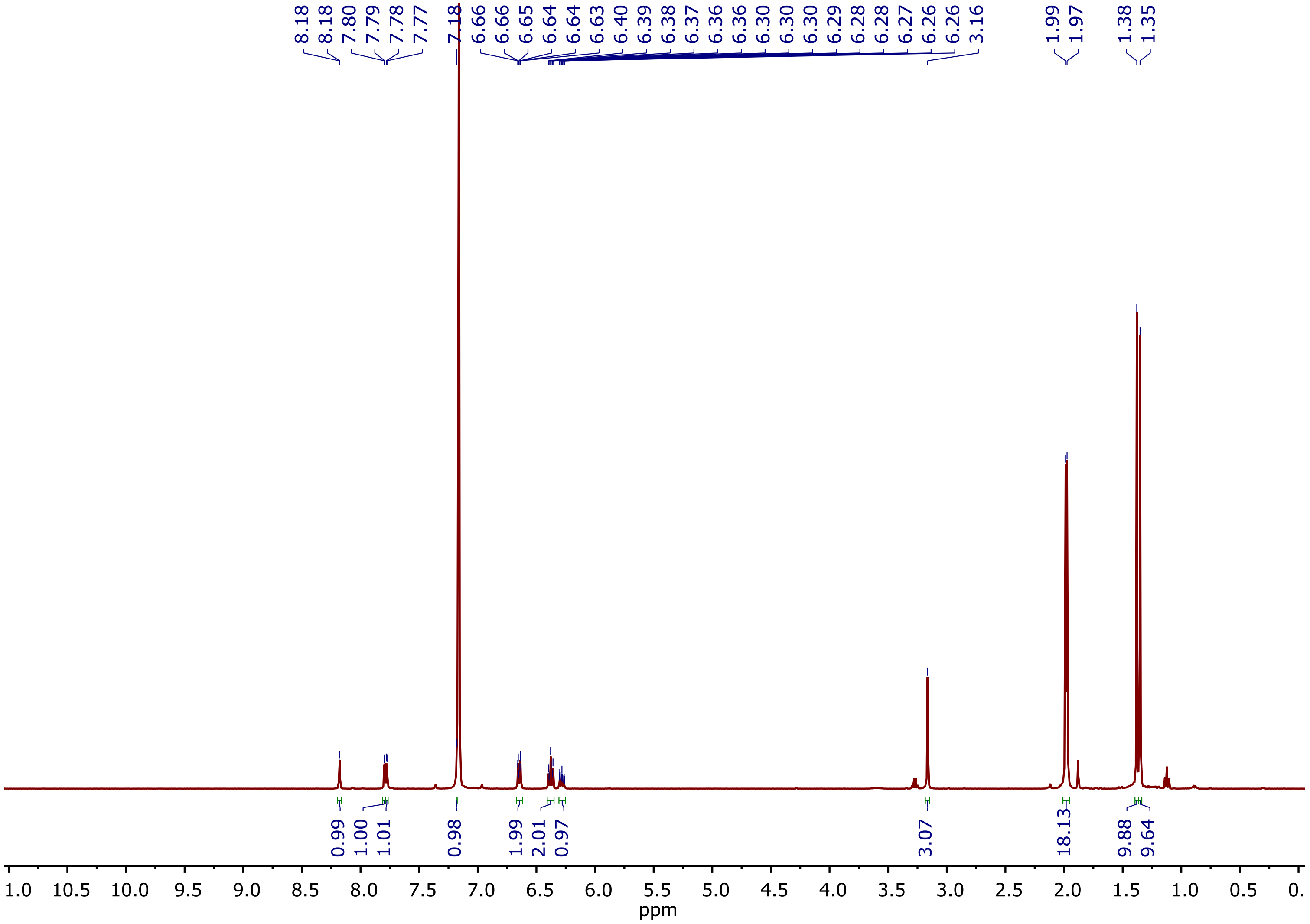

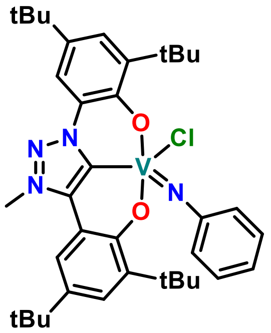

Figure S 27: ^{1}H NMR of **7** in C_6D_6 at 298 K.

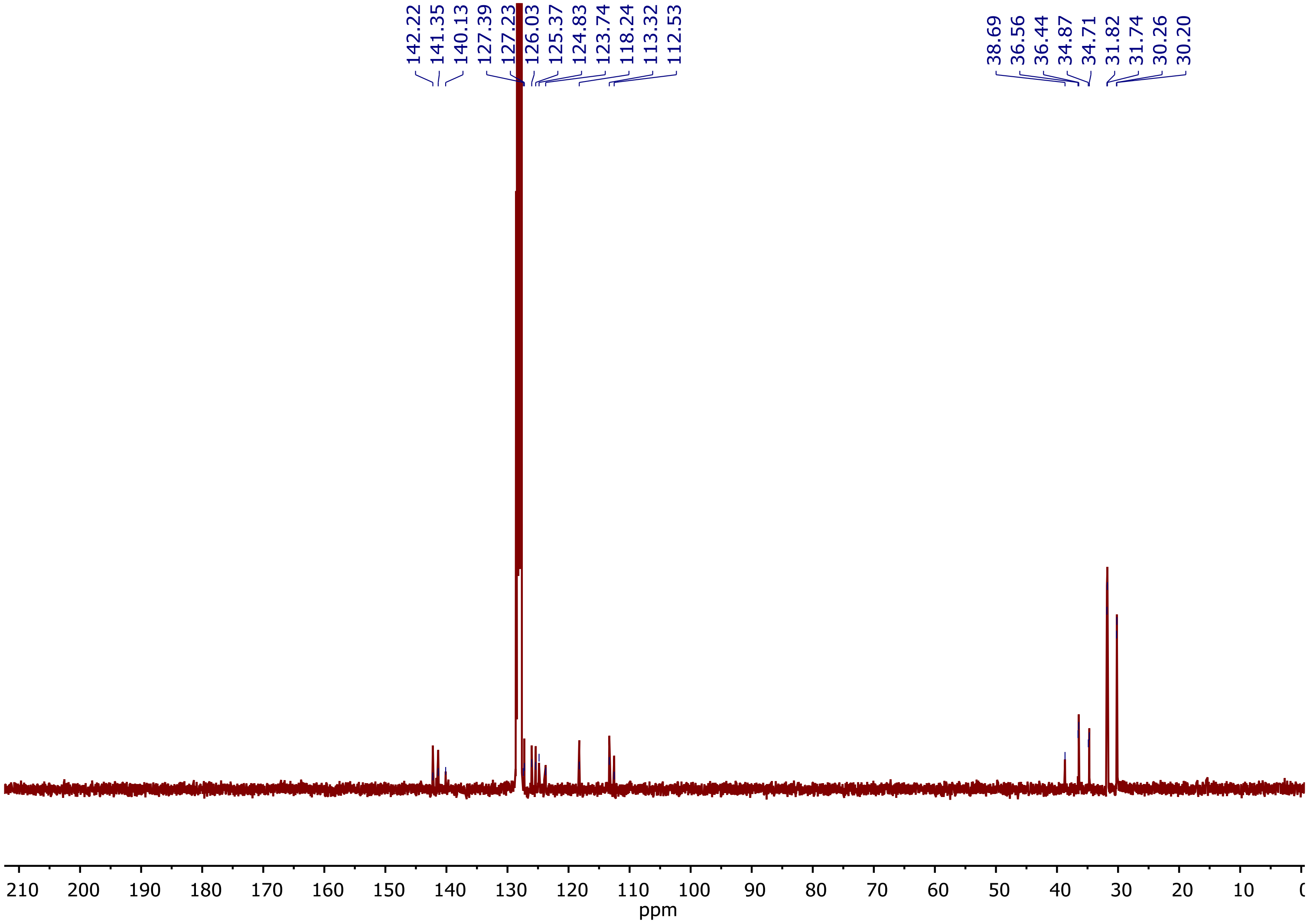

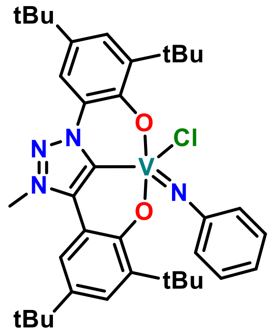

Figure S 28: ^{13}C NMR of **7** in C_6D_6 at 298 K.

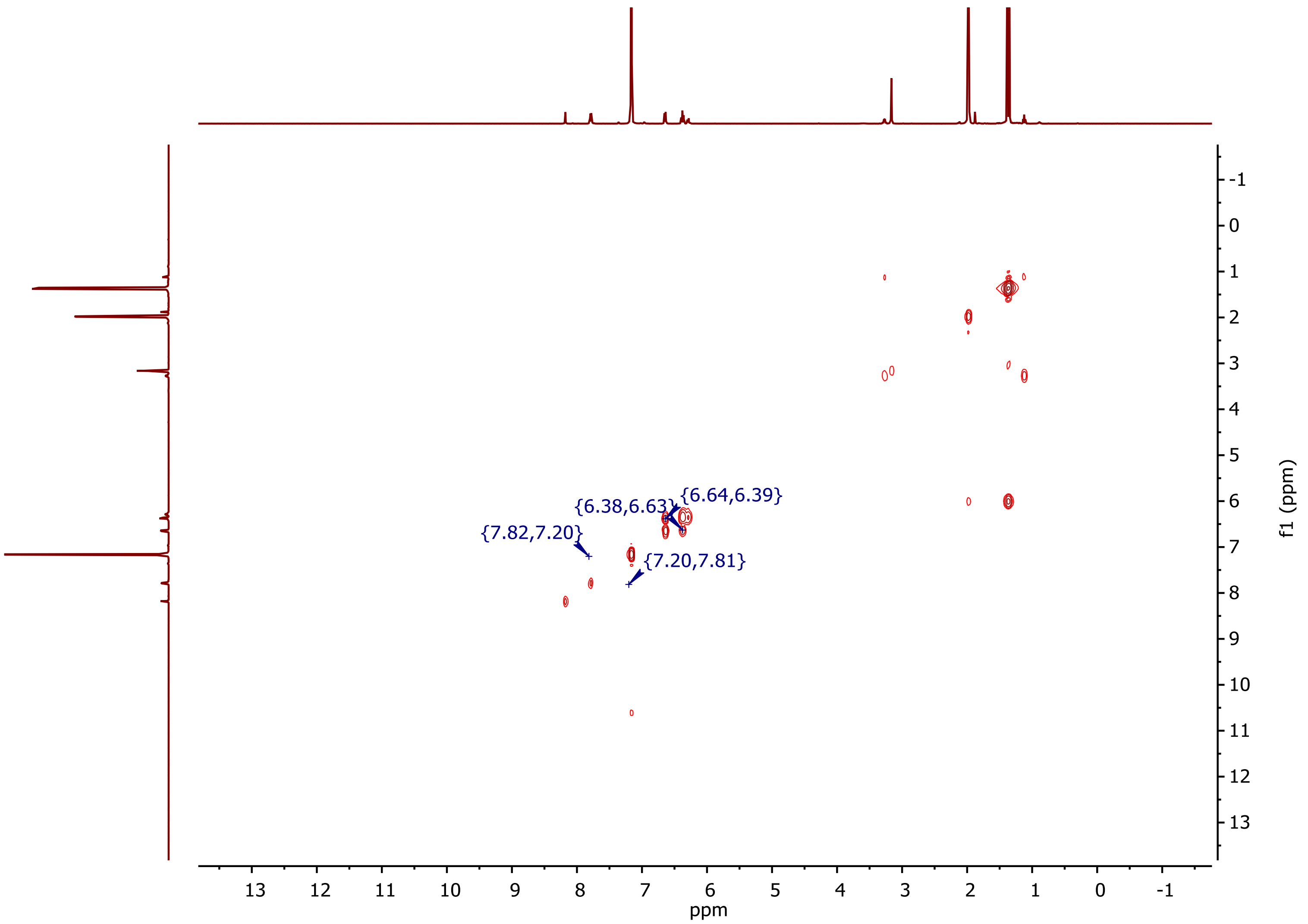

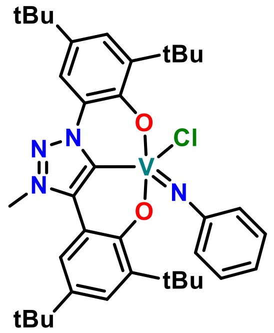

Figure S 29: ^{1}H ^{1}H COSY NMR of **7** in C_6D_6 at 298 K.

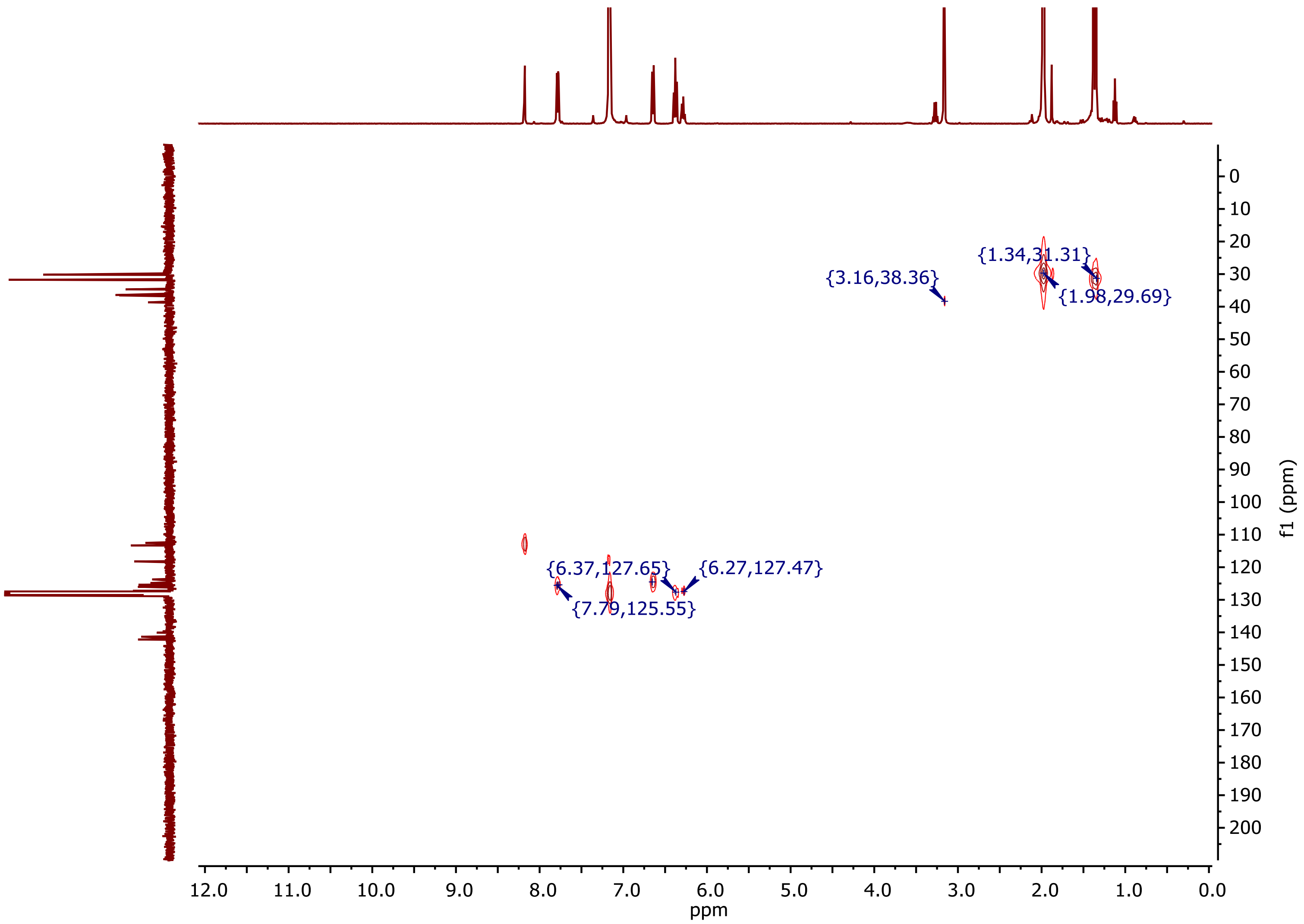

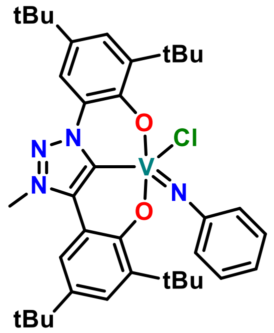

Figure S 30: ^{1}H ^{13}C HSQC NMR of **7** in C_6D_6 at 298 K.

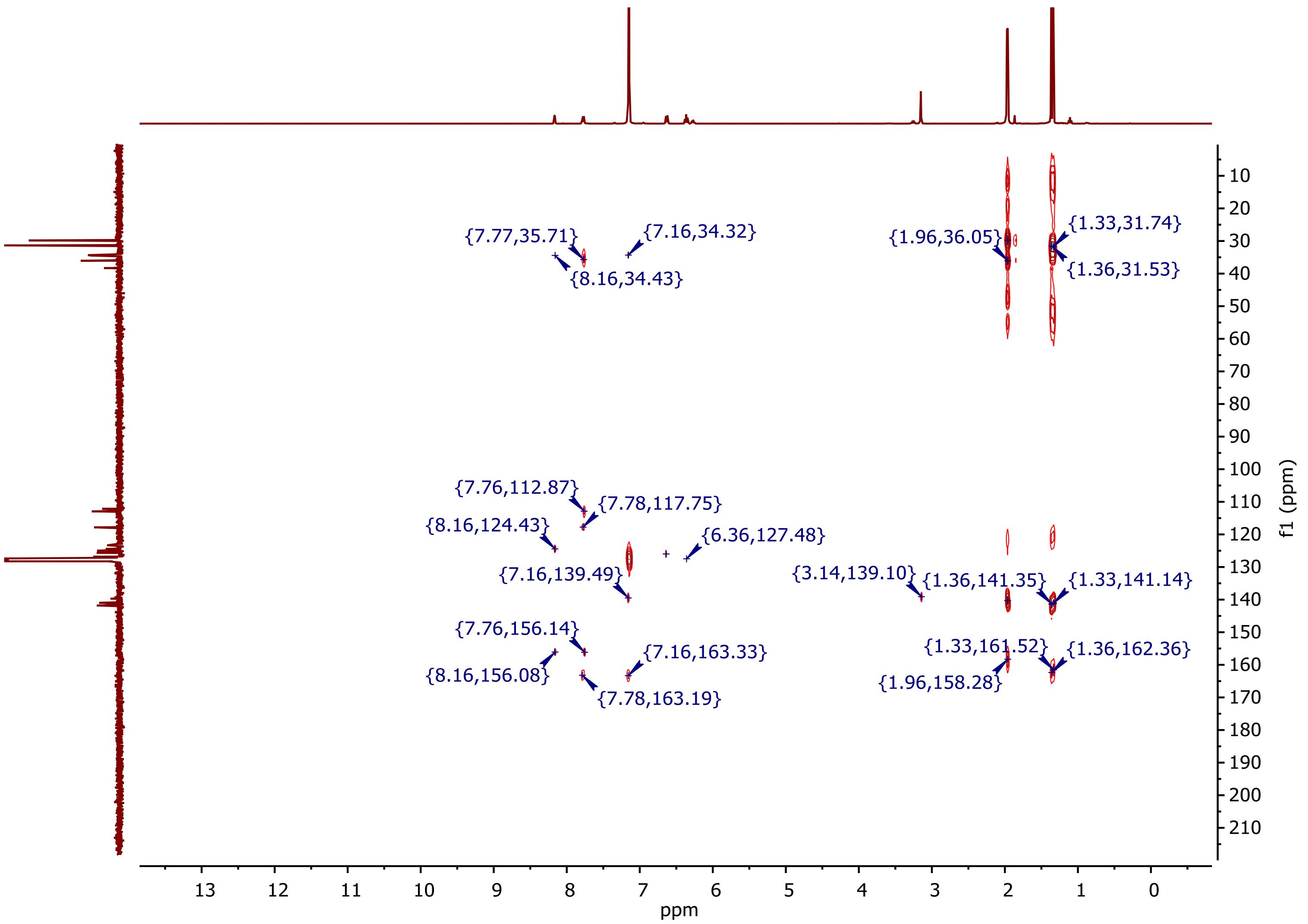

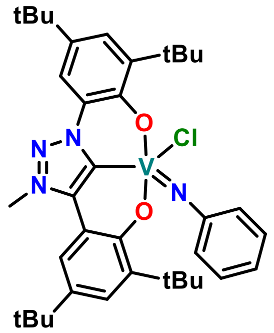

Figure S 31: ^{1}H ^{13}C HMBC NMR of **7** in C_6D_6 at 298 K.

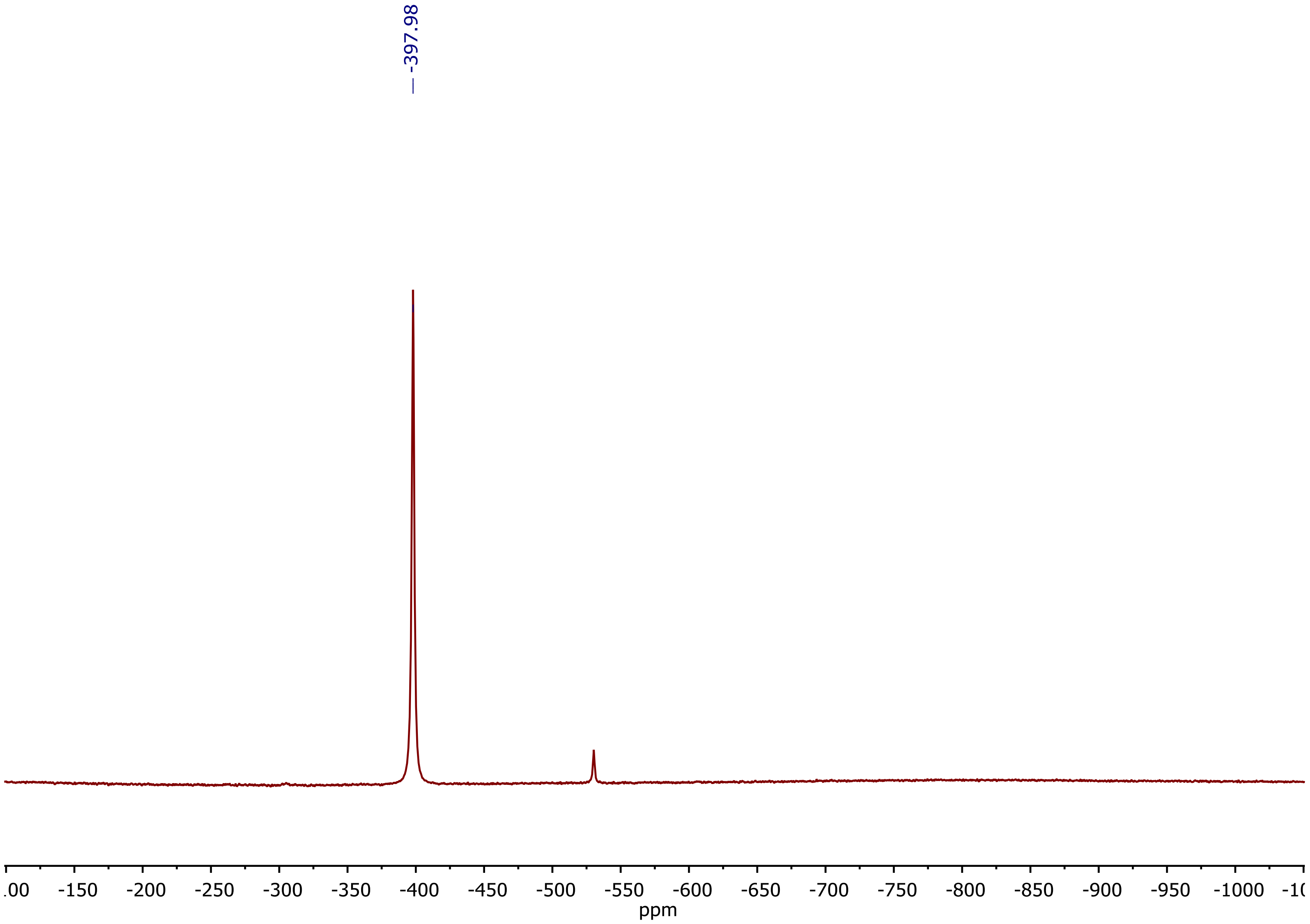

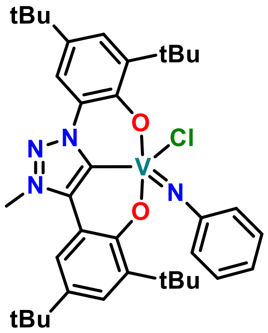

Figure S 32: ^{51}V NMR of **7** in C_6D_6 at 298 K.

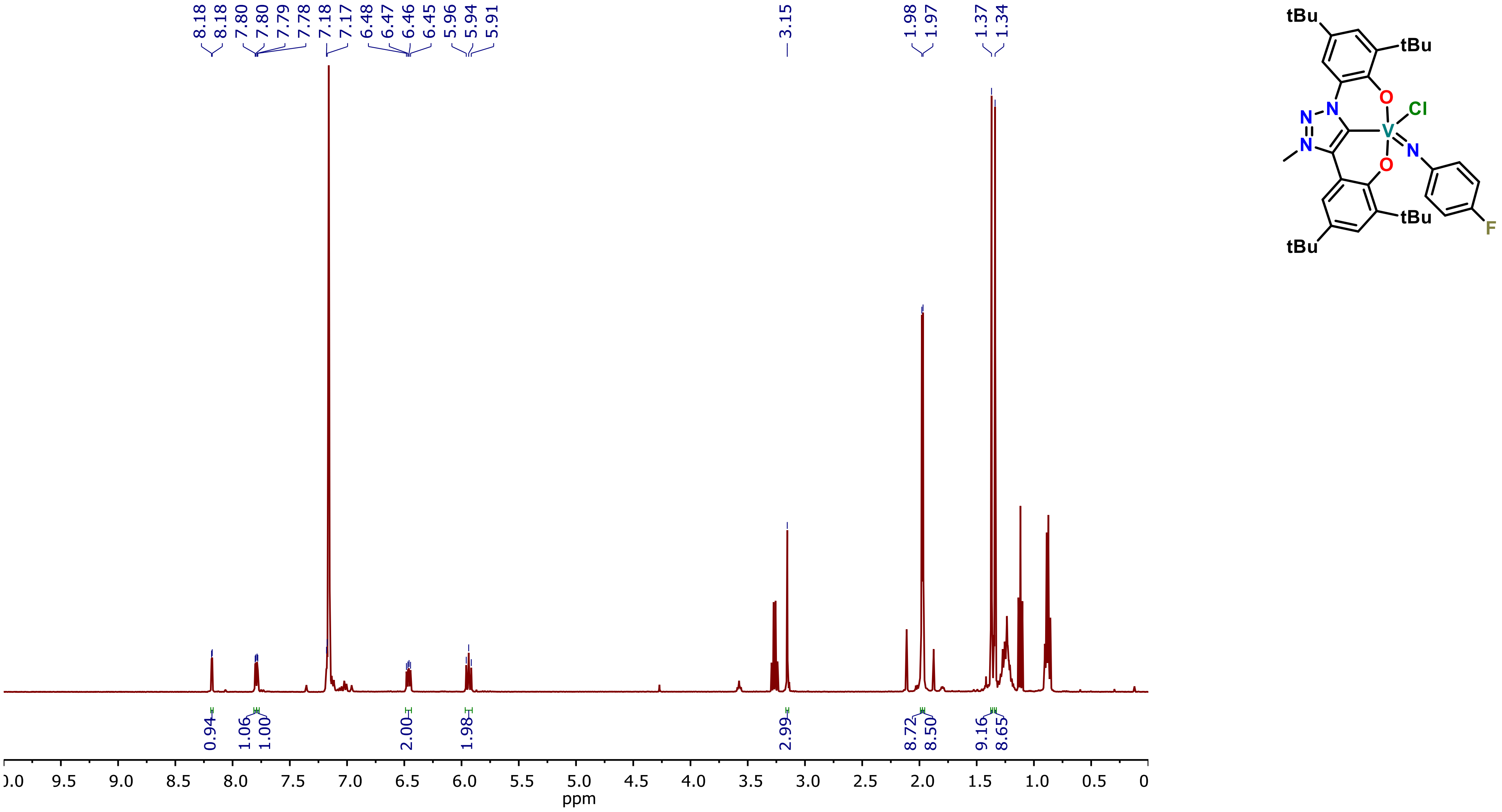

Figure S 33: ^{1}H NMR of **8** in C_6D_6

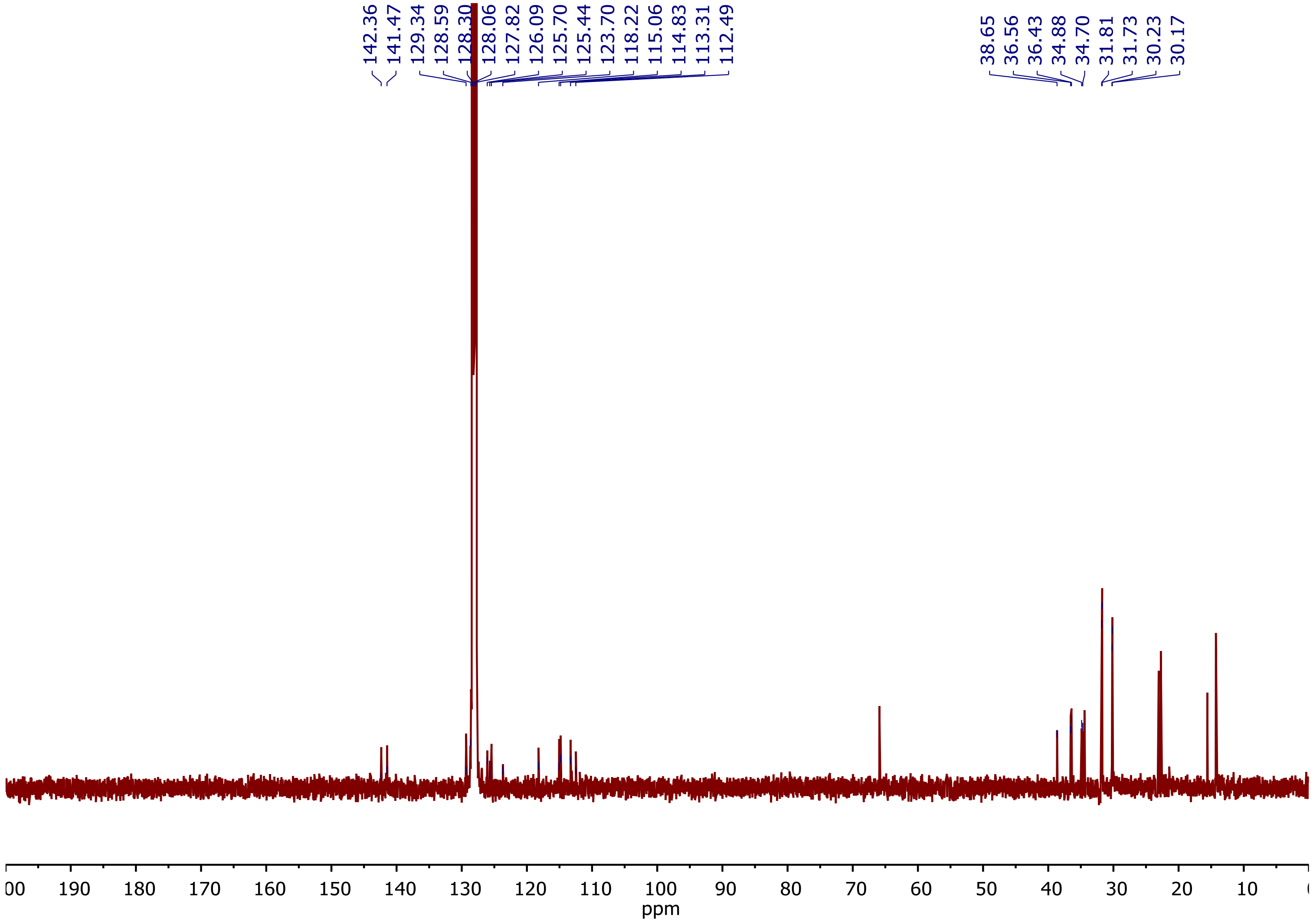

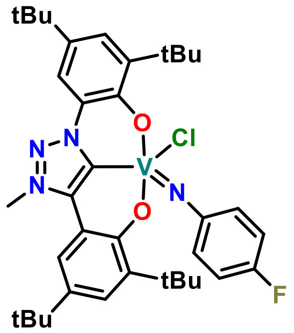

Figure S 34: ^{13}C NMR of **8** in C_6D_6

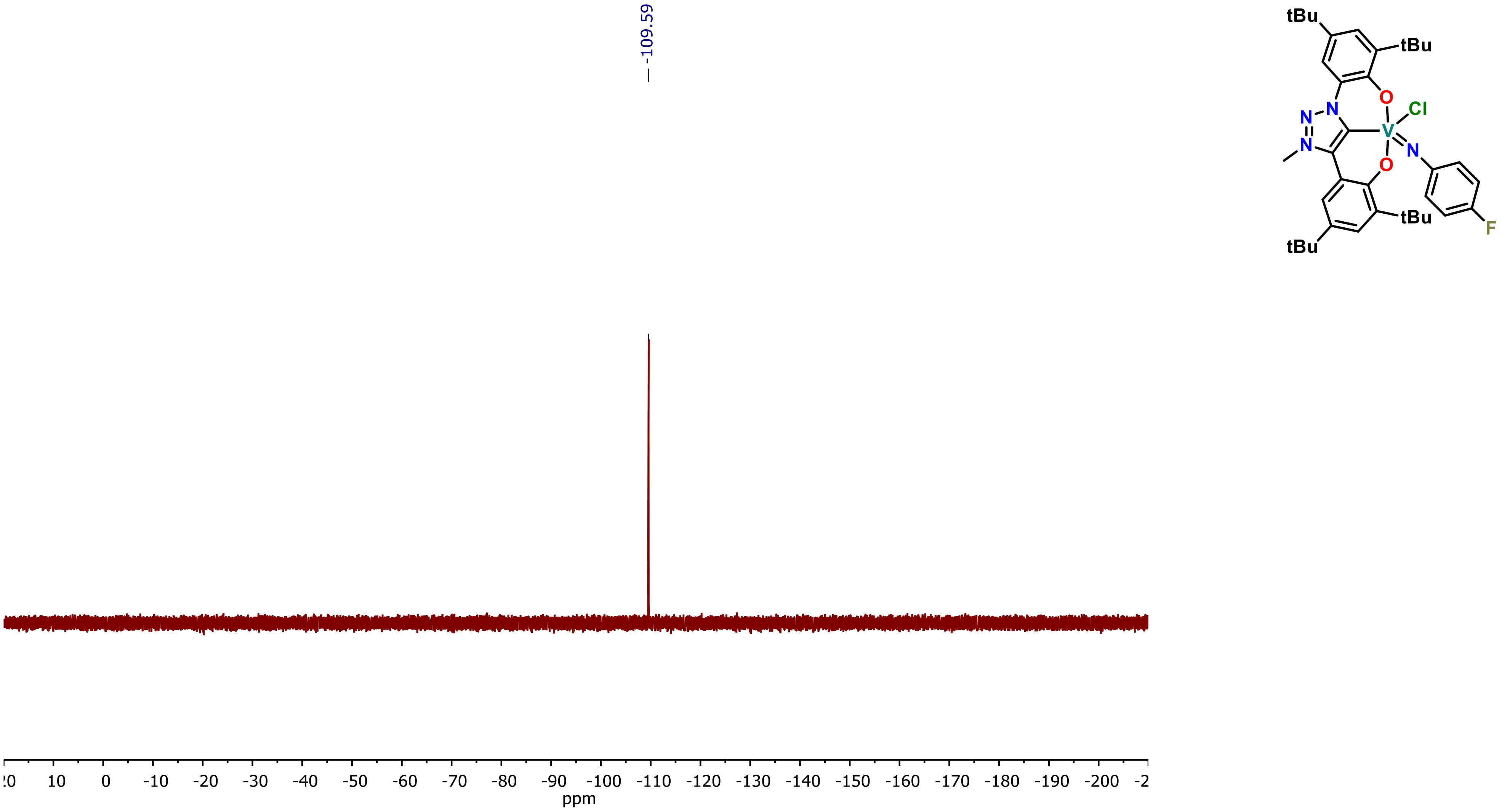

Figure S 35: ^{19}F NMR of **8** in C_6D_6

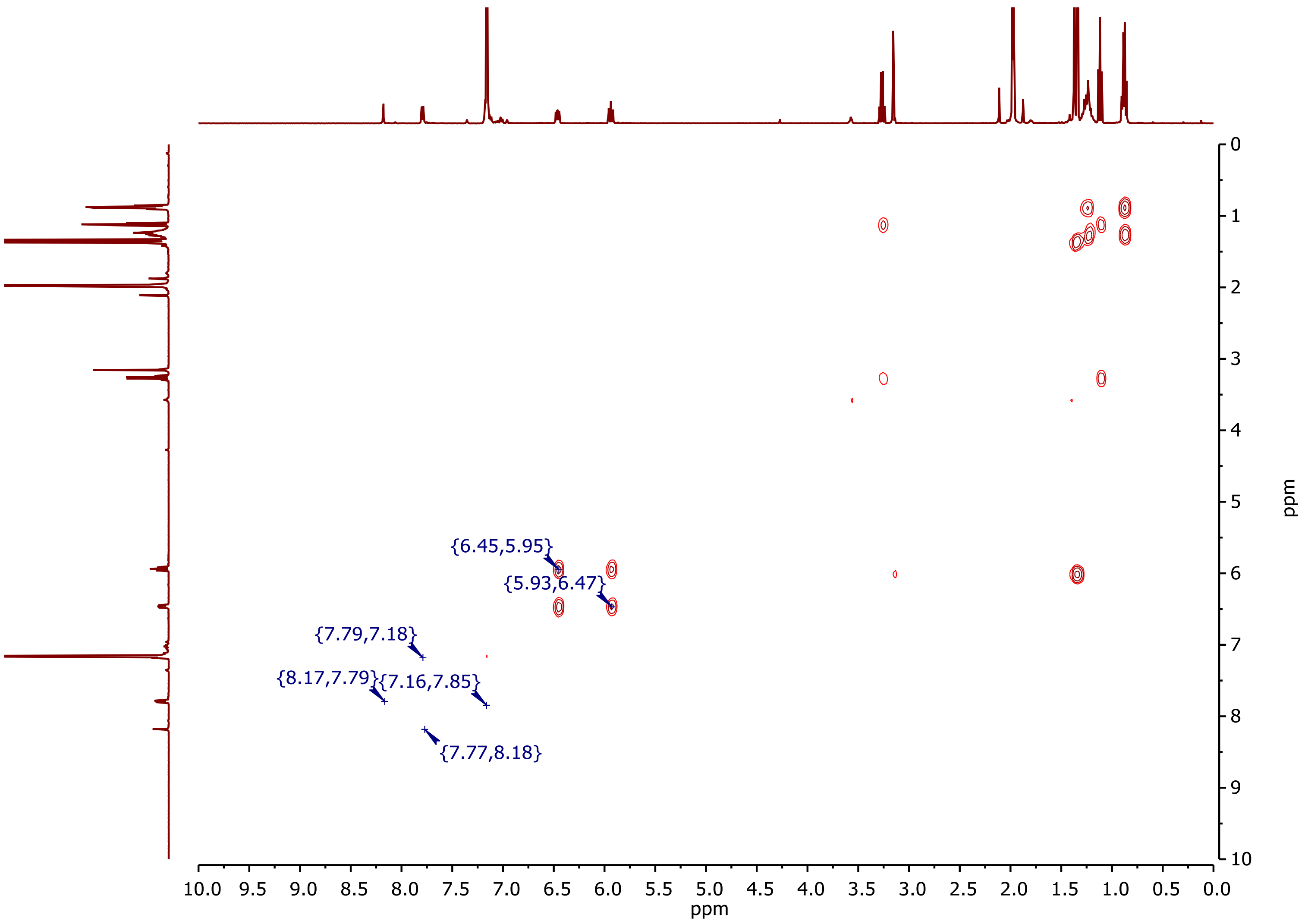

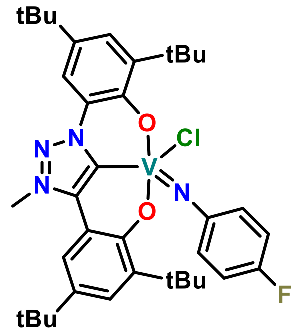

Figure S 36: ^{1}H ^{1}H COSY of **8** in C_6D_6

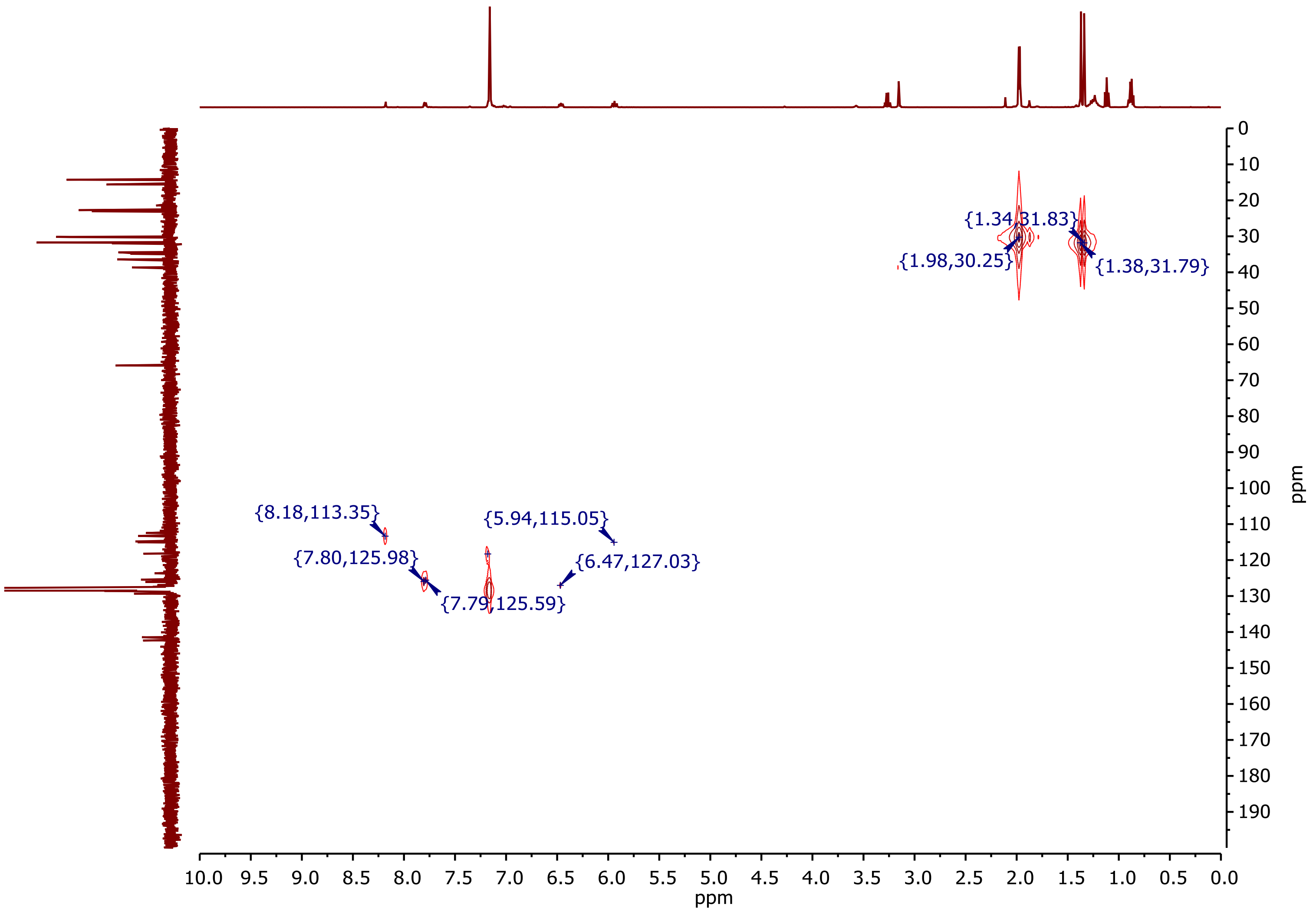

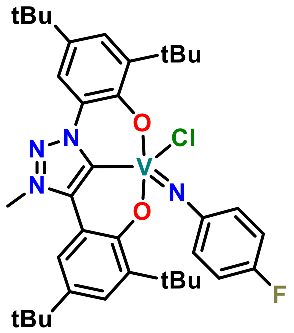

Figure S 37: ^{1}H ^{13}C HSQC of **8** in C_6D_6

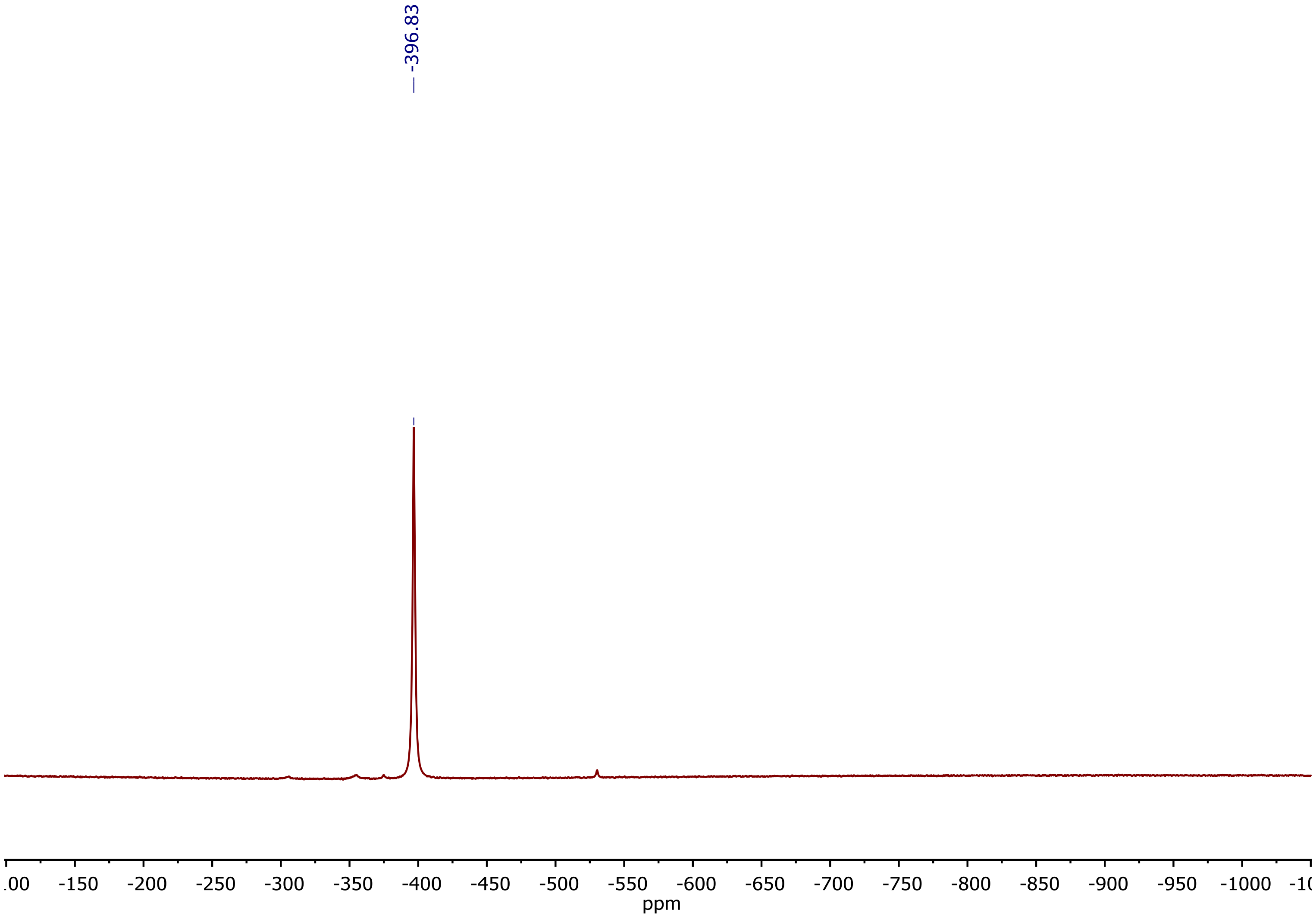

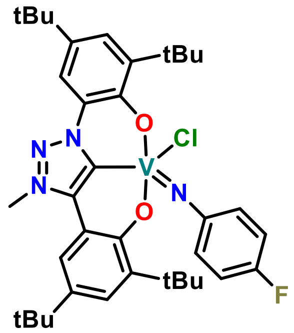

Figure S 38: ^{51}V NMR of **8** in C_6D_6

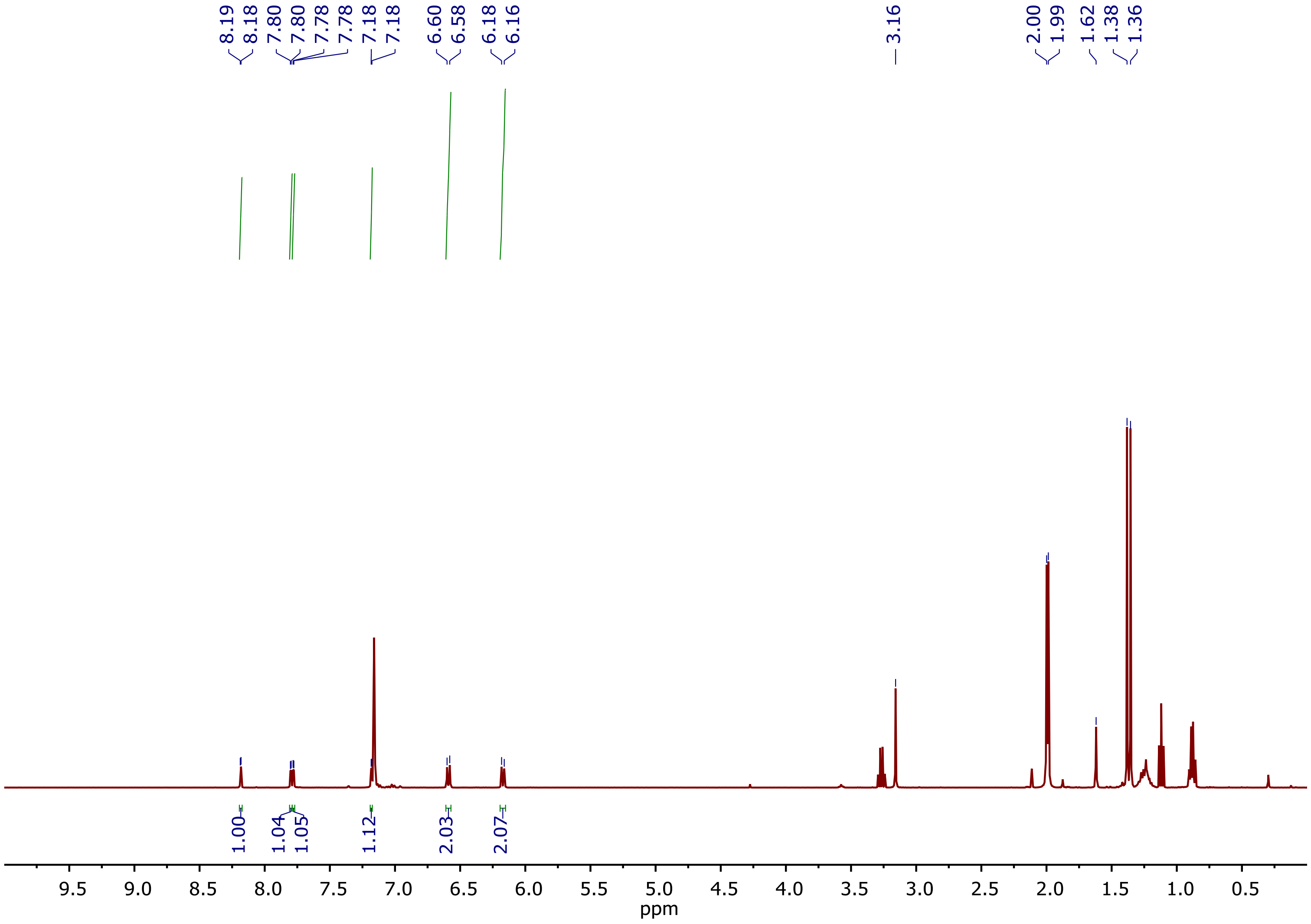

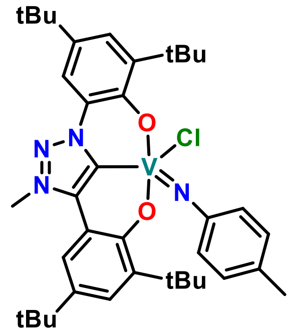

Figure S 39: ^{1}H NMR of **9** in C_6D_6

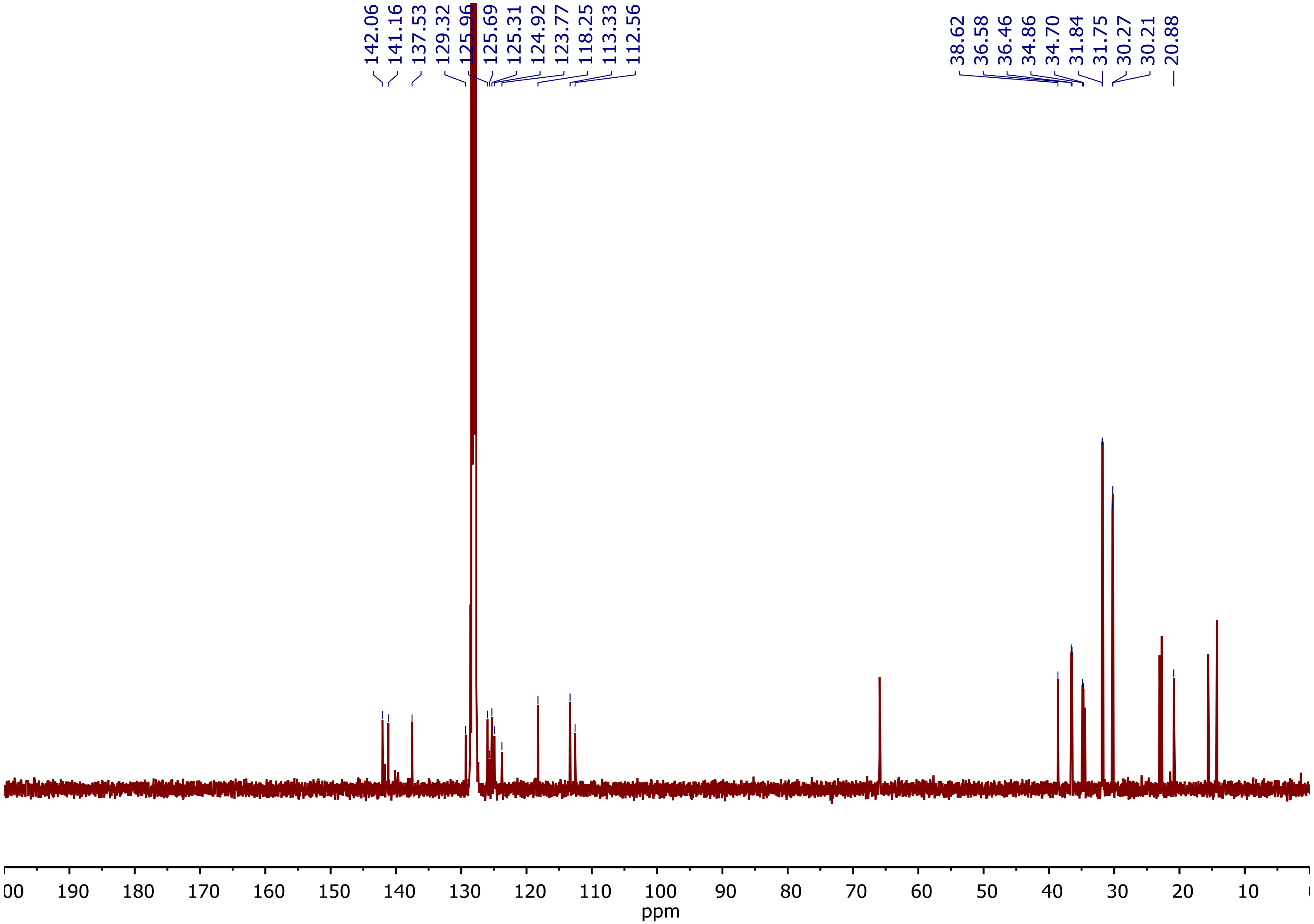

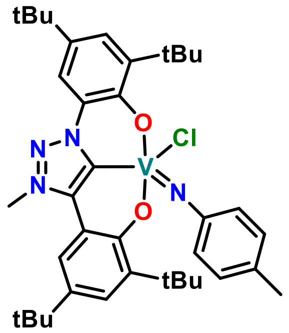

Figure S 40: ^{13}C NMR of **9** in C_6D_6

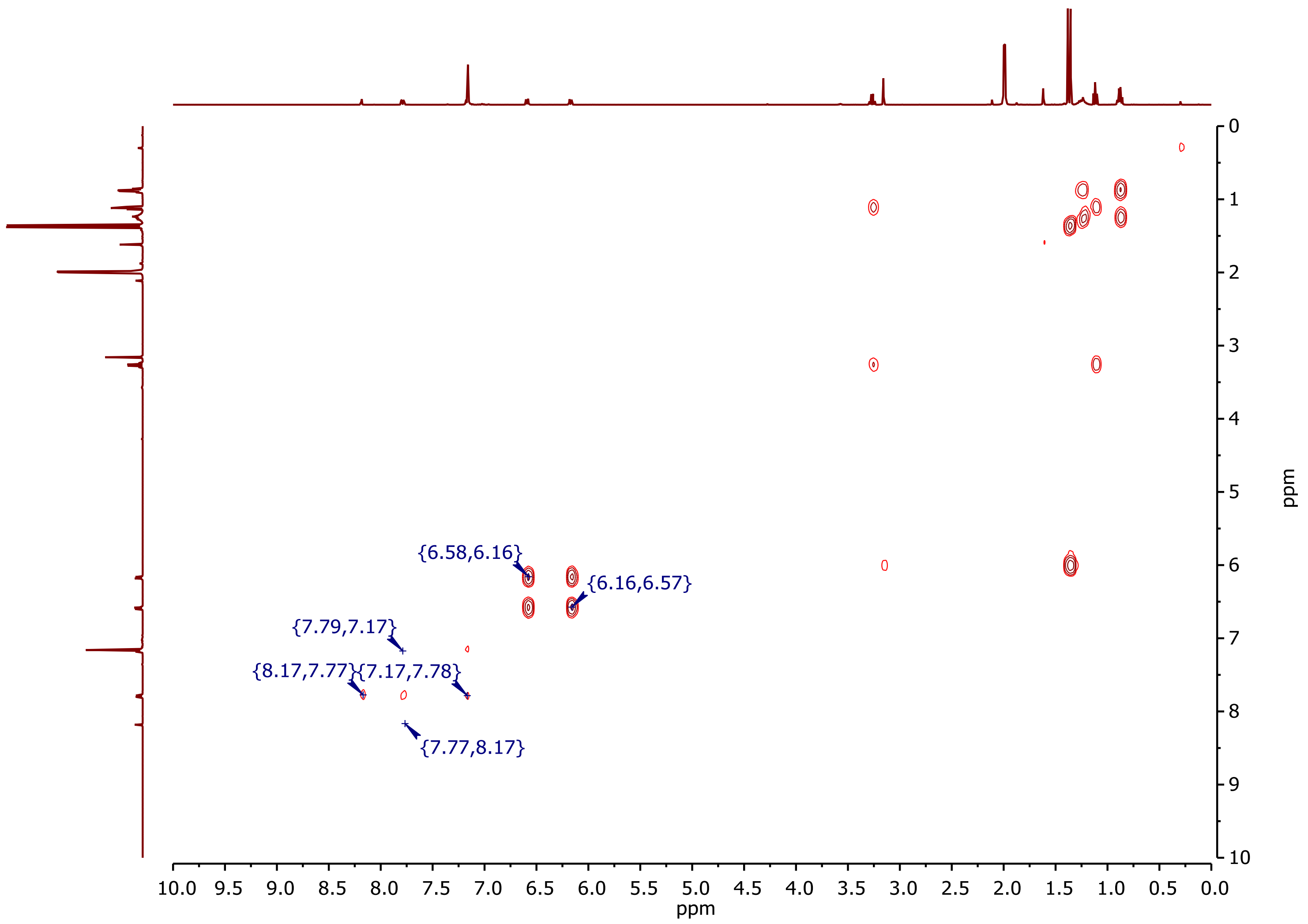

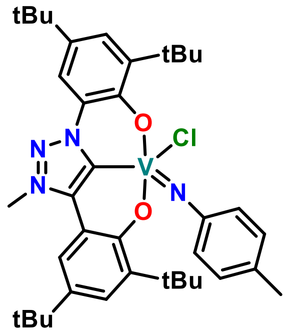

Figure S 41: ^1H ^1H COSY of **9** in C_6D_6

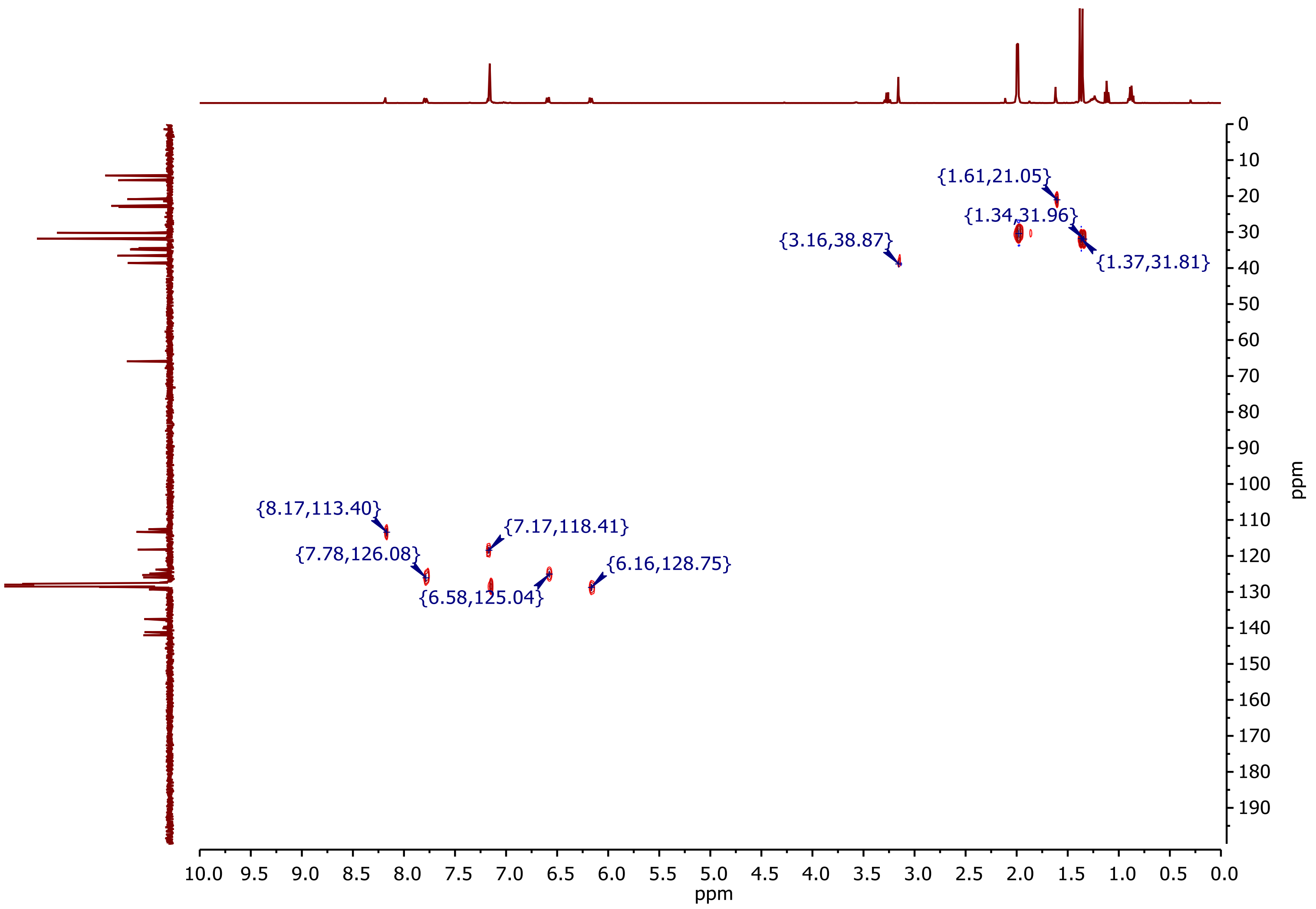

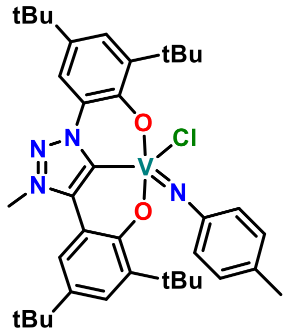

Figure S 42: ^{1}H ^{13}C HSQC of **9** in C_6D_6

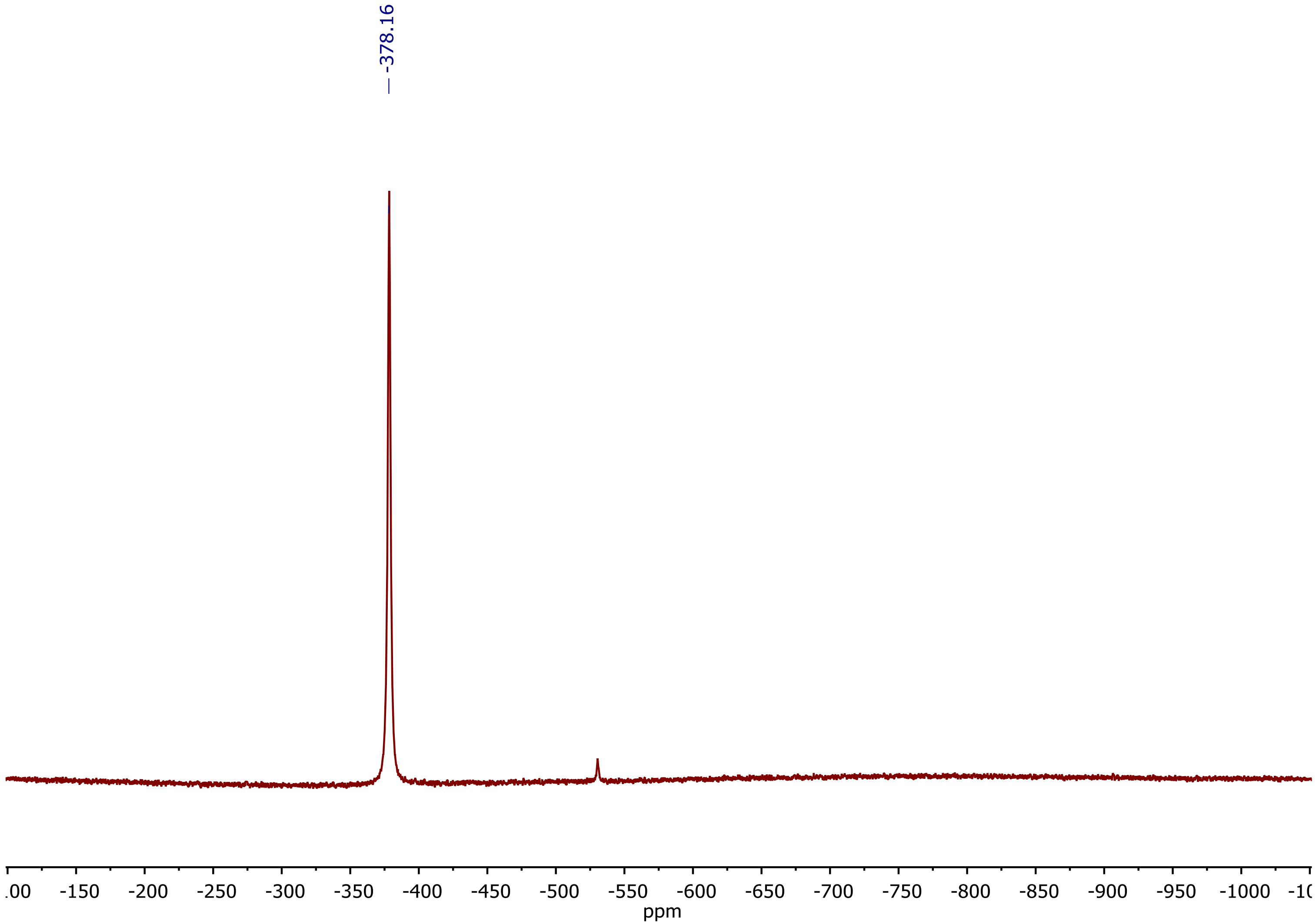

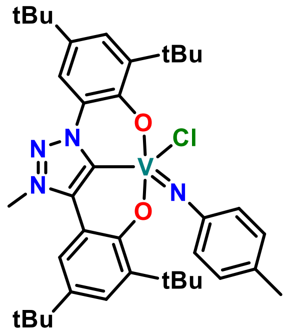

Figure S 43: ^{51}V NMR of **9** in C_6D_6

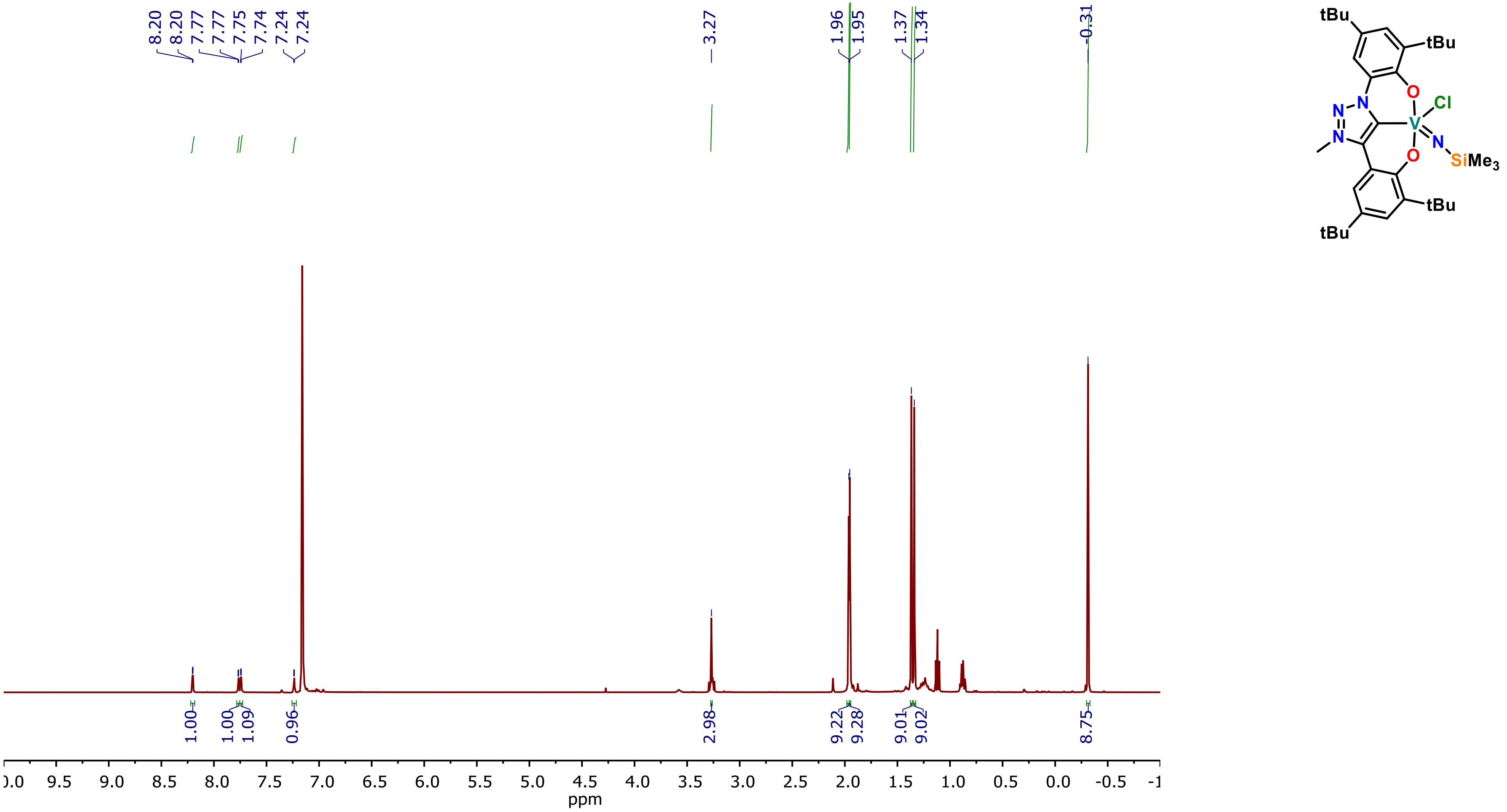

Figure S 44: ^{1}H NMR of **10** in C_6D_6

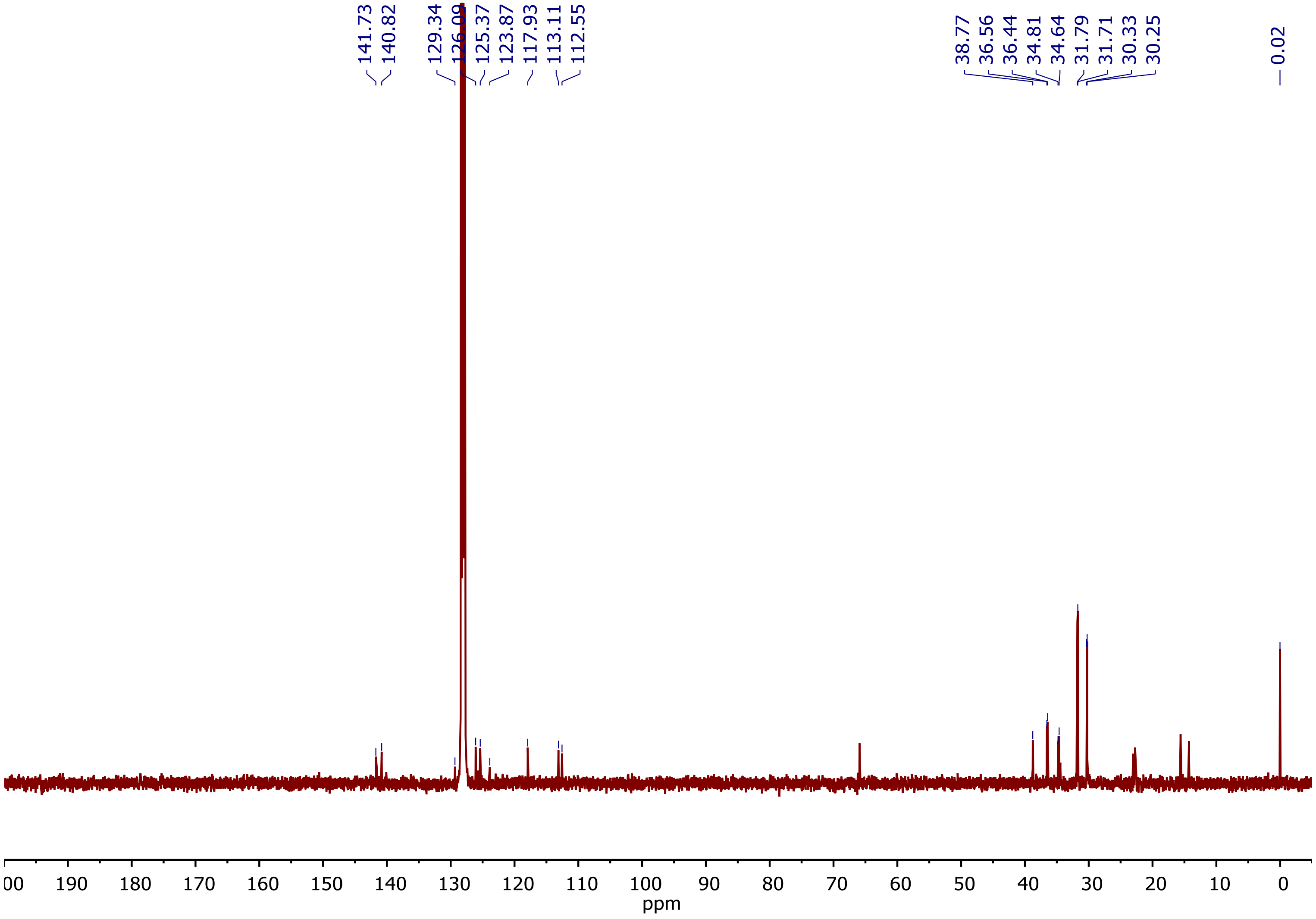

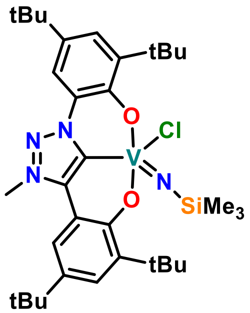

Figure S 45: ^{13}C NMR of **10** in C_6D_6

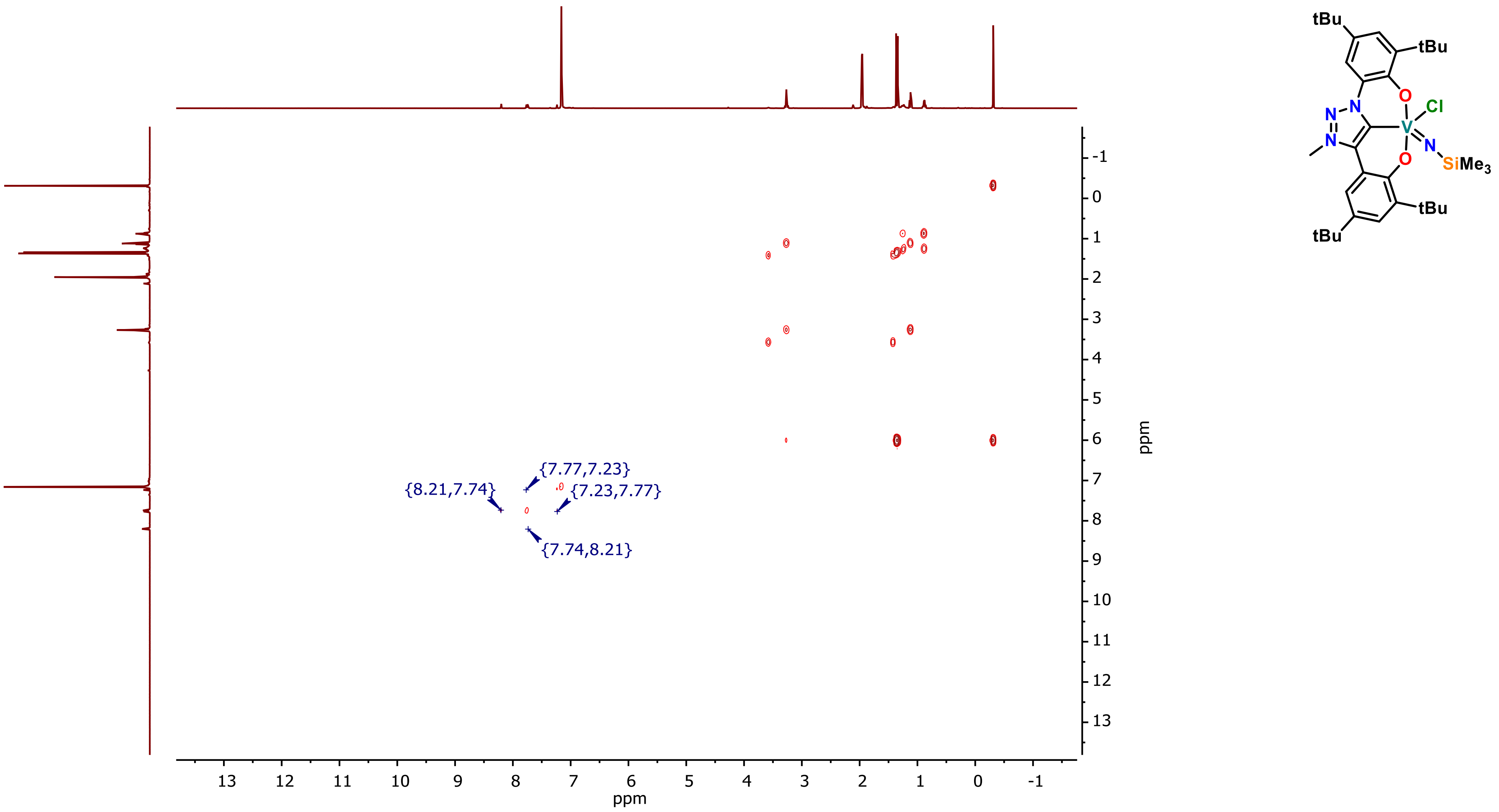

Figure S 46: ^1H ^1H COSY of **10** in C_6D_6. Cross peaks in the aliphatic region arise from solvent impurities (diethyl ether, pentane and THF as well as from large T1 noise from the ^{t}Bu and $SiMe_3$ groups.

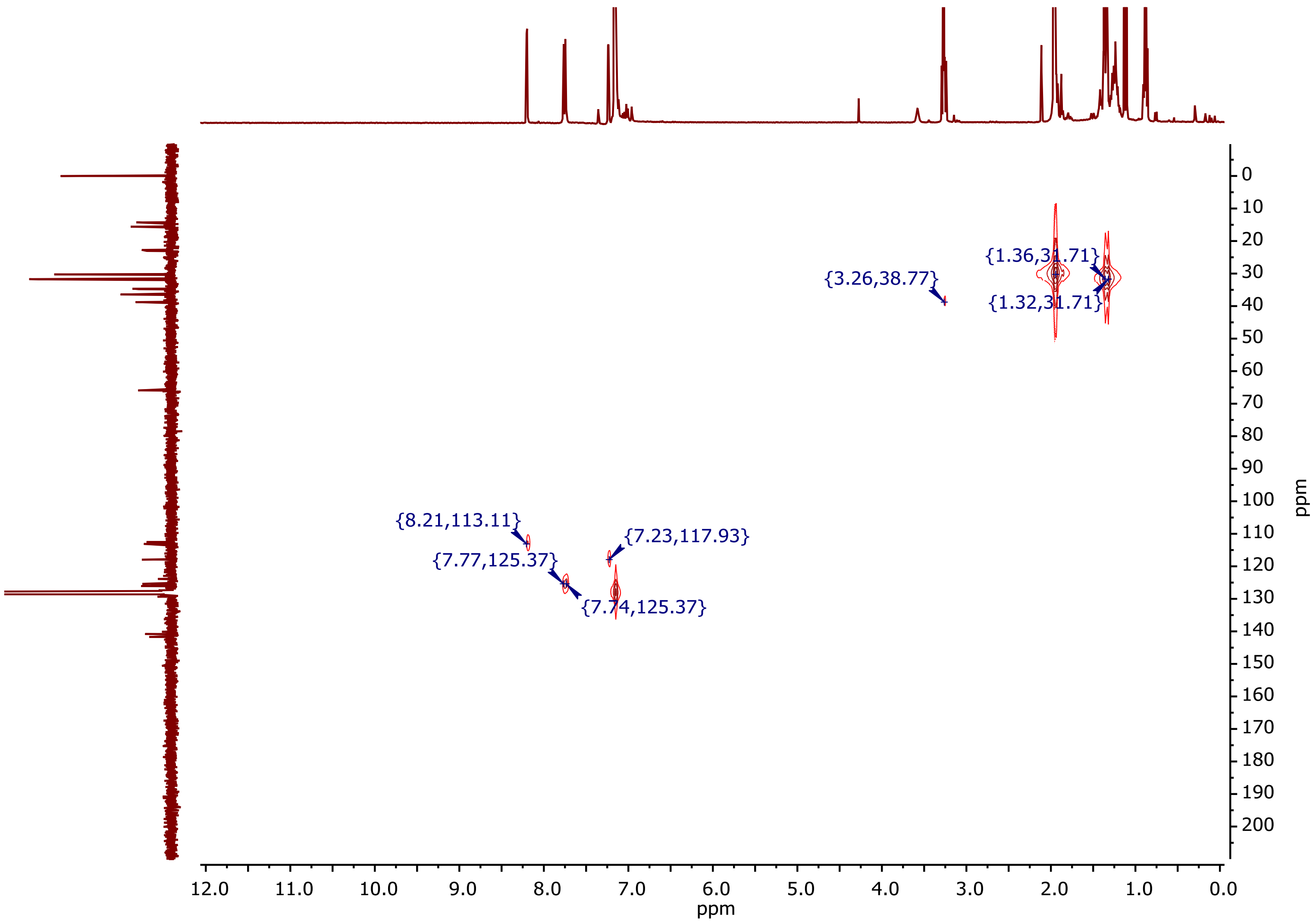

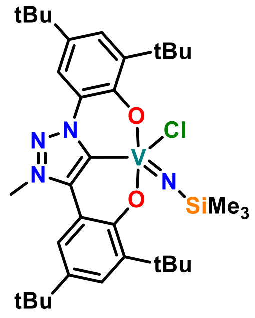

Figure S 47: ^{1}H ^{13}C HSQC of **10** in C_6D_6

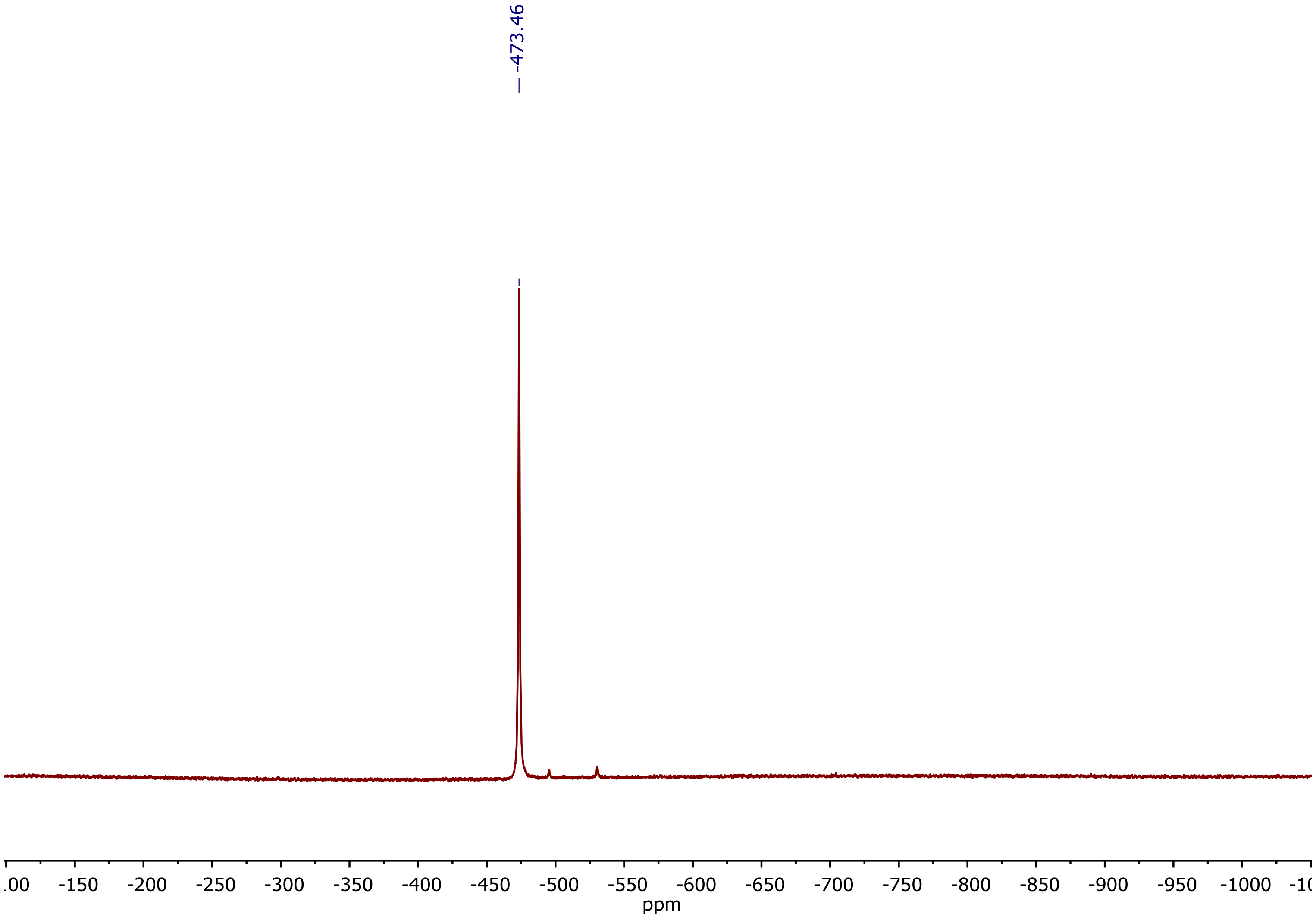

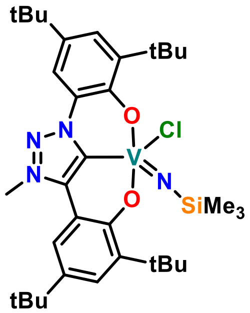

Figure S 48: ^{51}V NMR of **10** in C_6D_6

2. IR spectroscopy

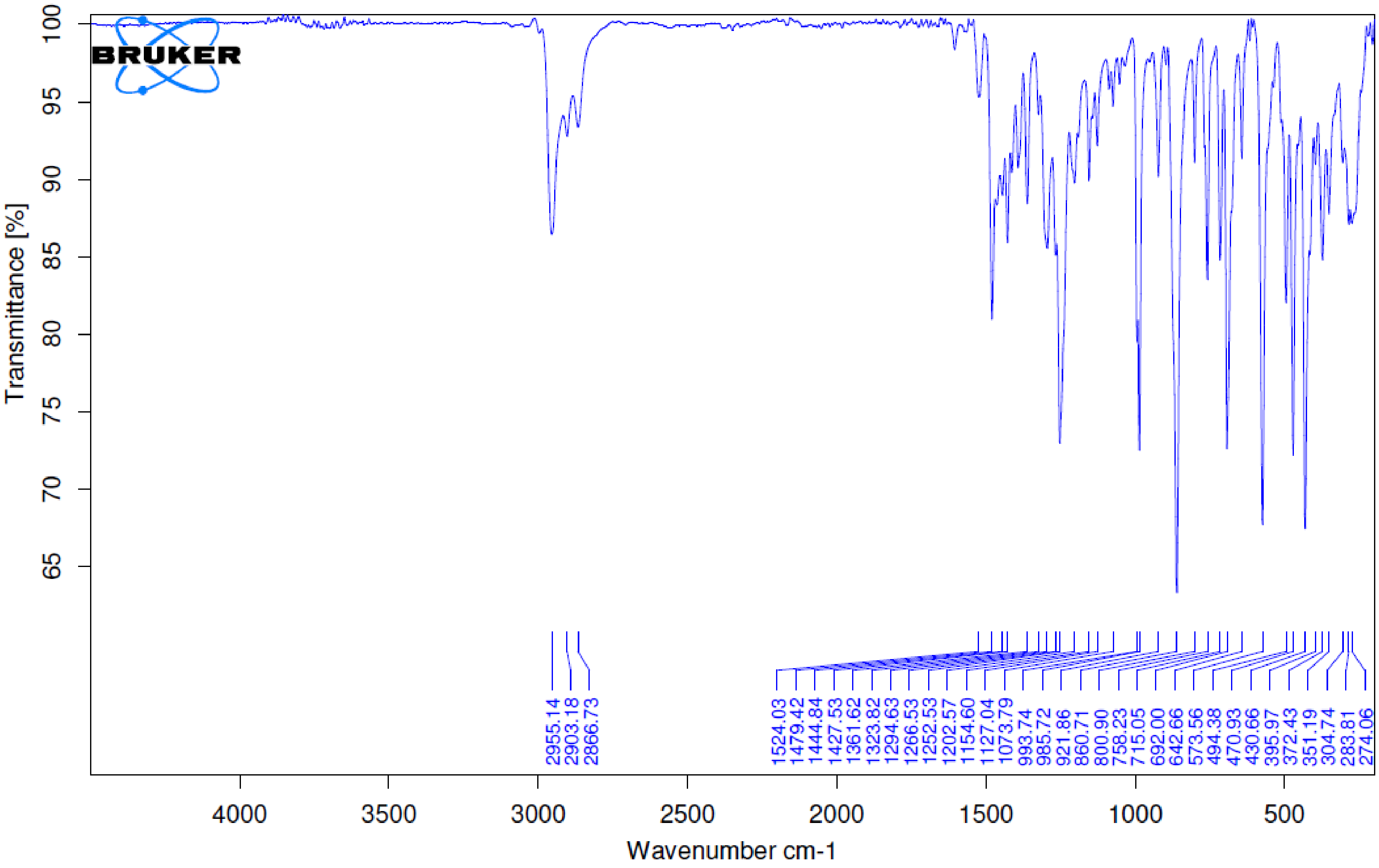

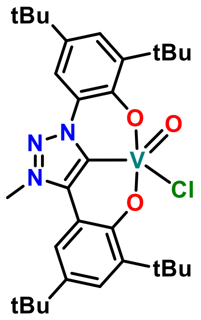

Figure S 49: ATR-IR spectrum of **1** at 298K.

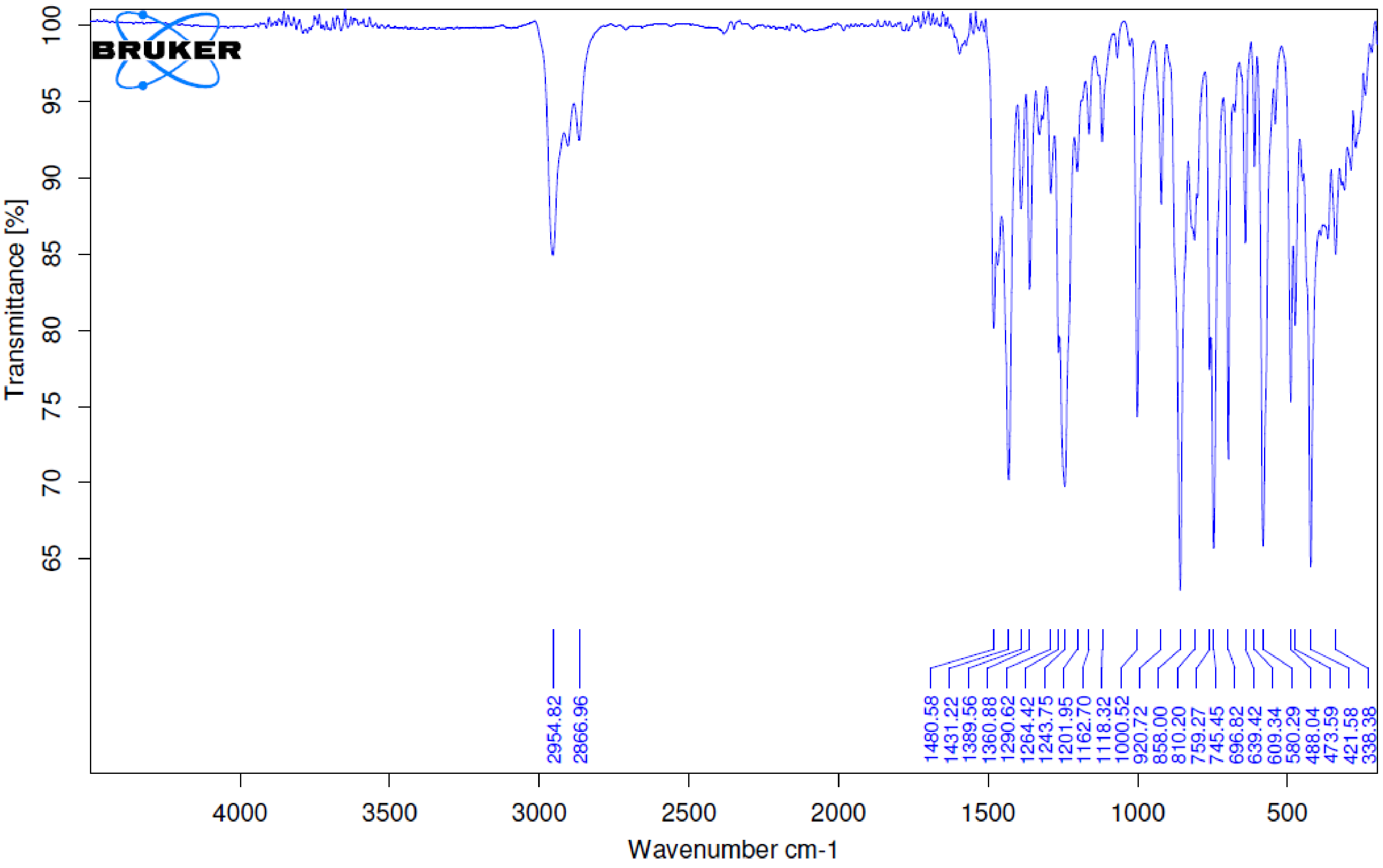

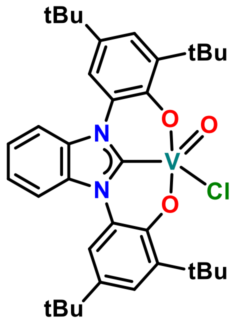

Figure S 50: ATR-IR spectrum of **2** at 298K.

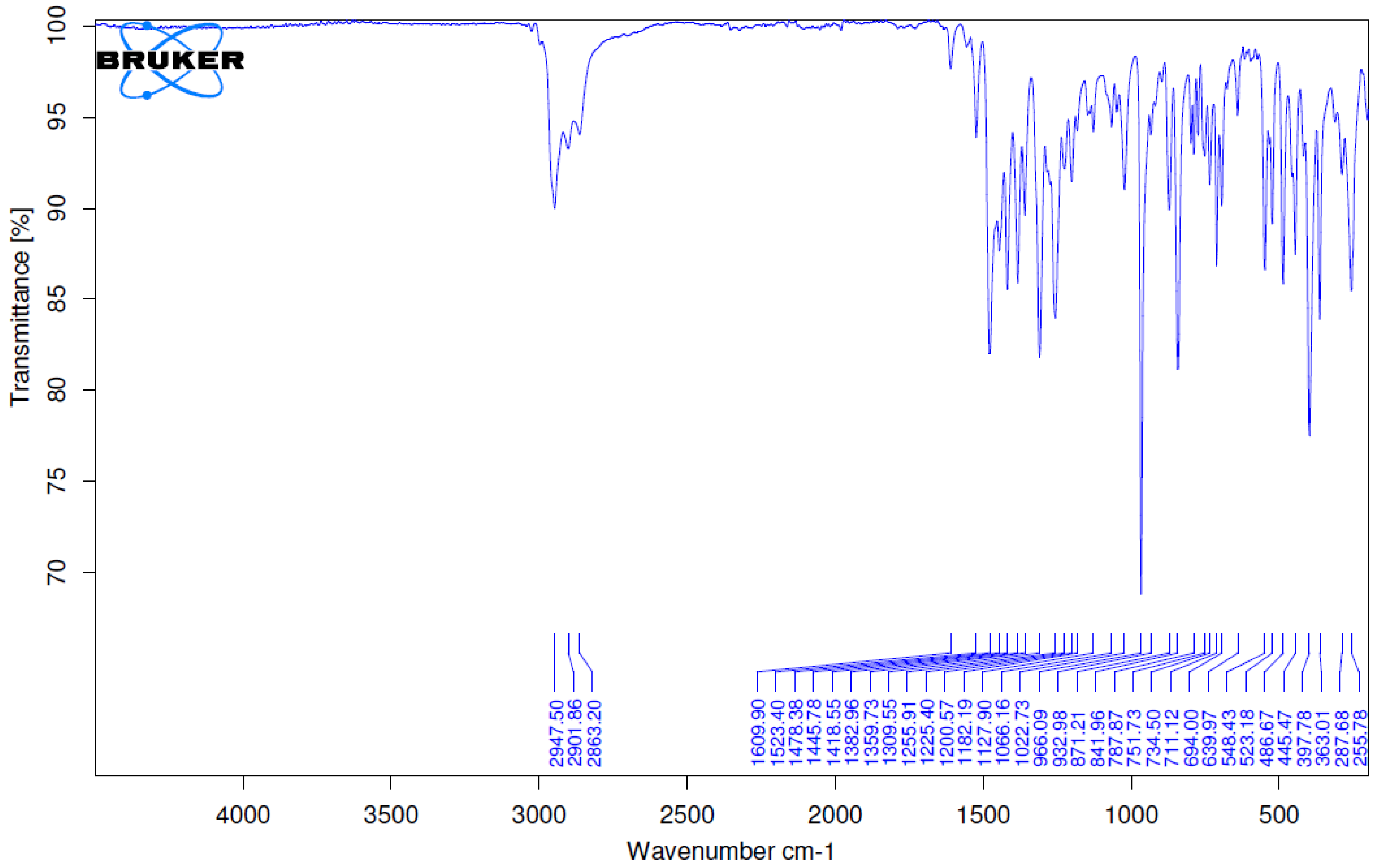

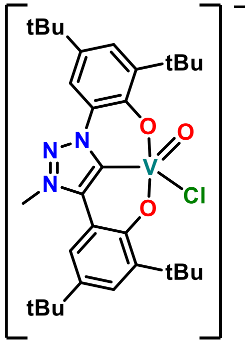

Figure S 51: ATR-IR spectrum of **[Co(Cp*)$_2$][1]** at 298K.

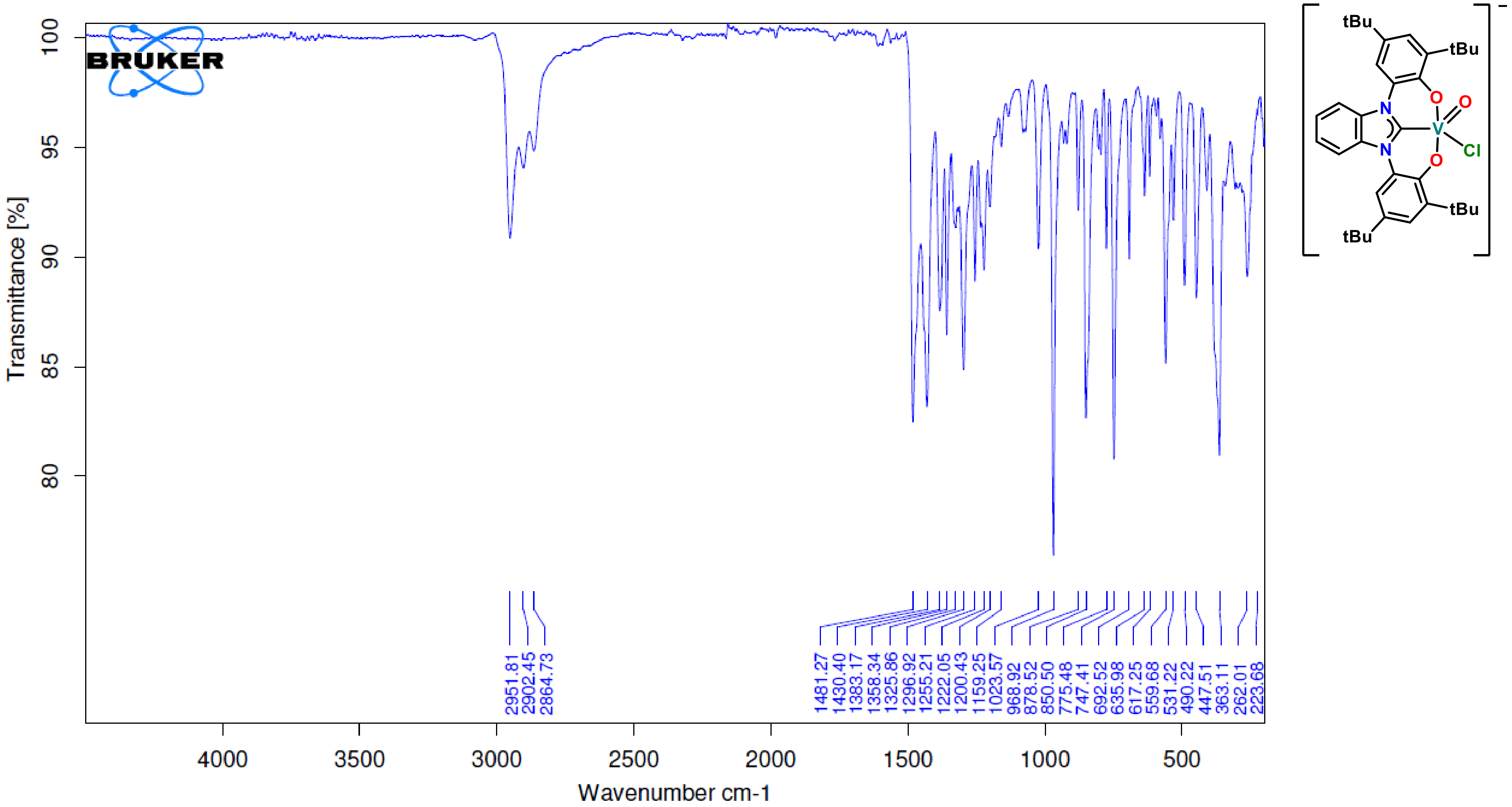

Figure S 52: ATR-IR spectrum of **[Co(Cp*)₂][2]** at 298K.

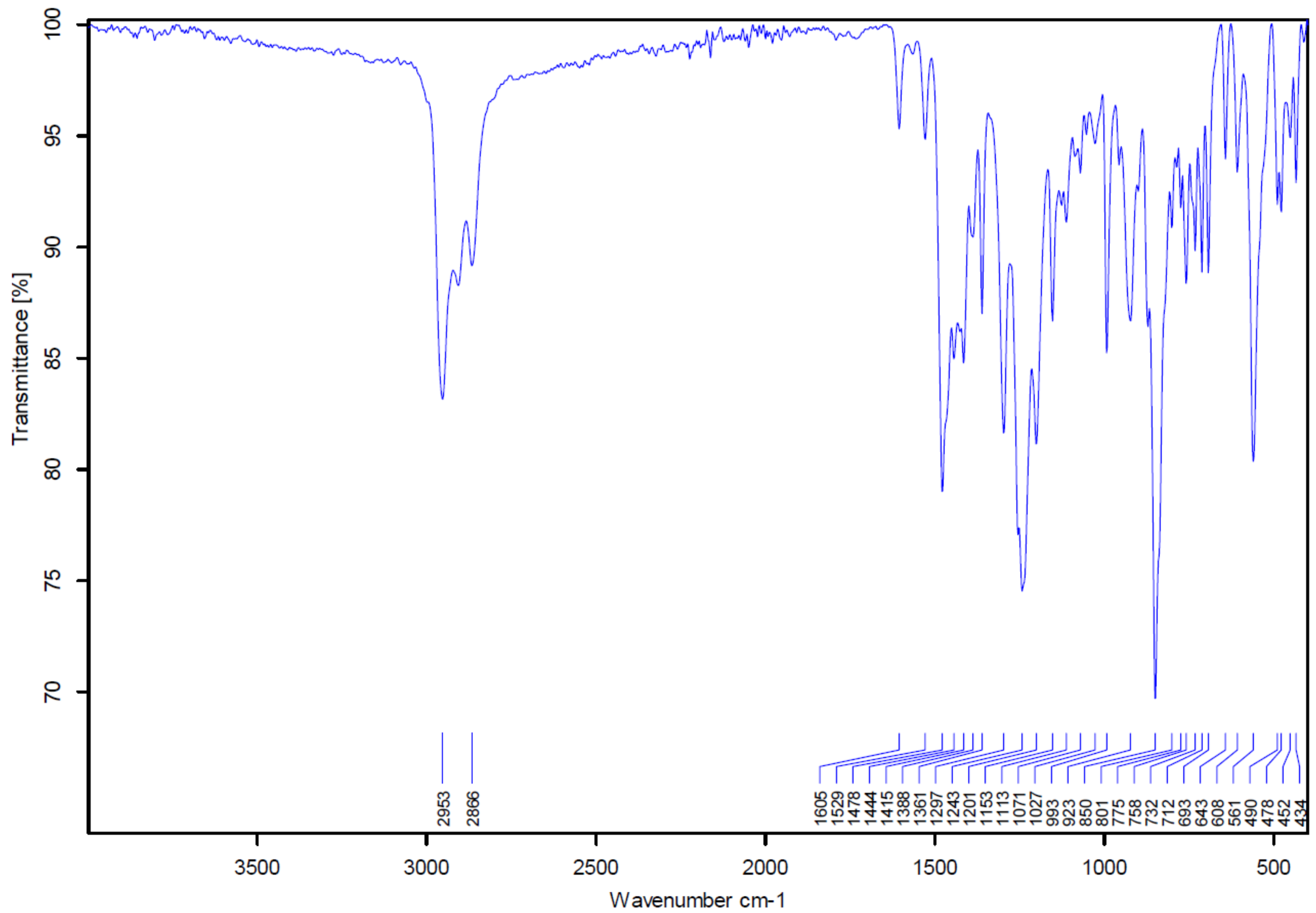

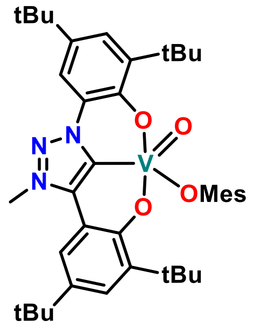

Figure S 53: ATR-IR spectrum of **3** at 298K.

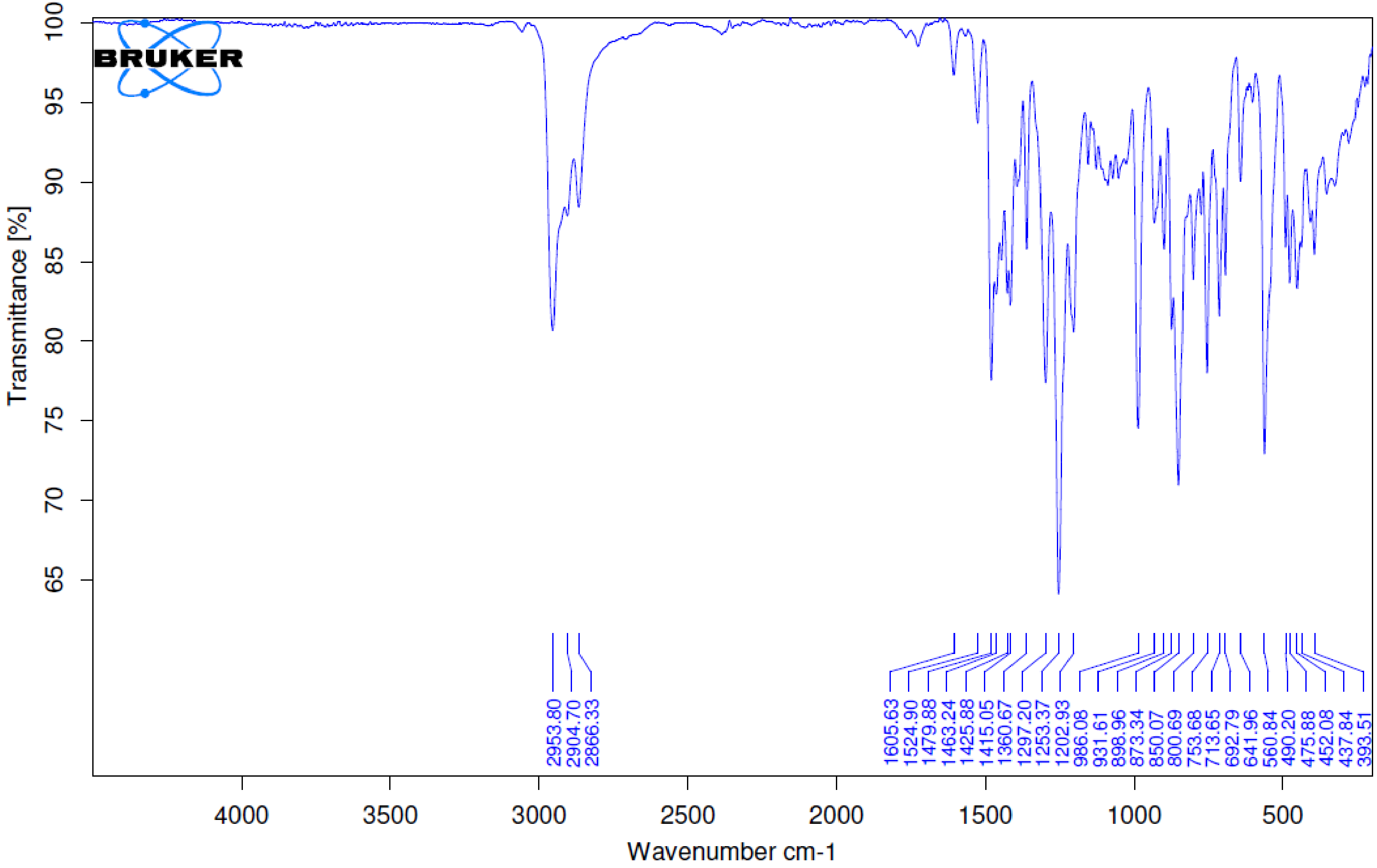

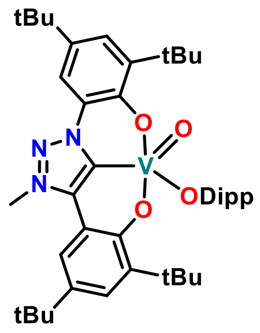

Figure S 54: ATR-IR spectrum of **4** at 298K.

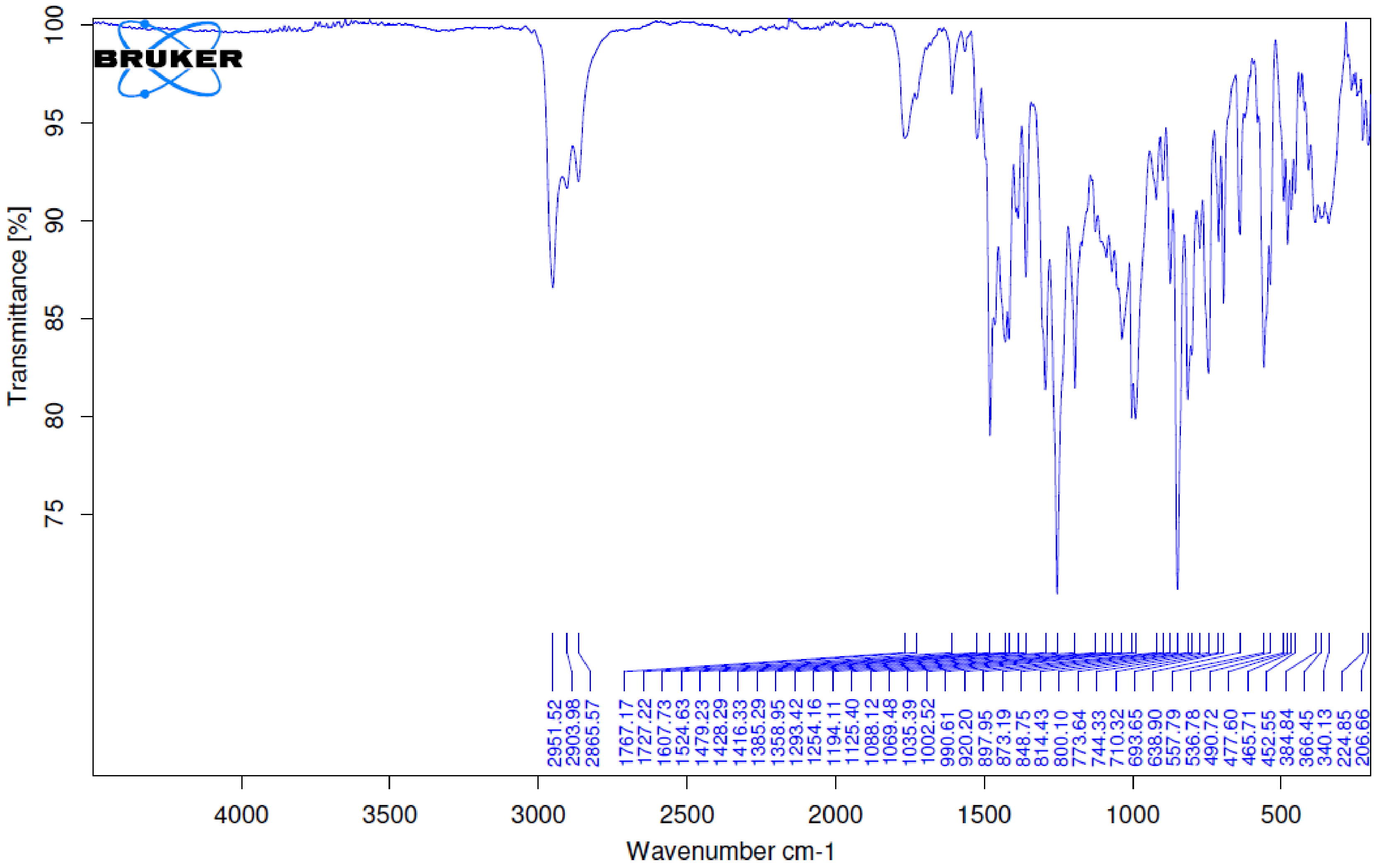

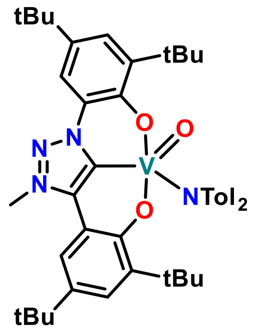

Figure S 55: ATR-IR spectrum of **5** at 298K.

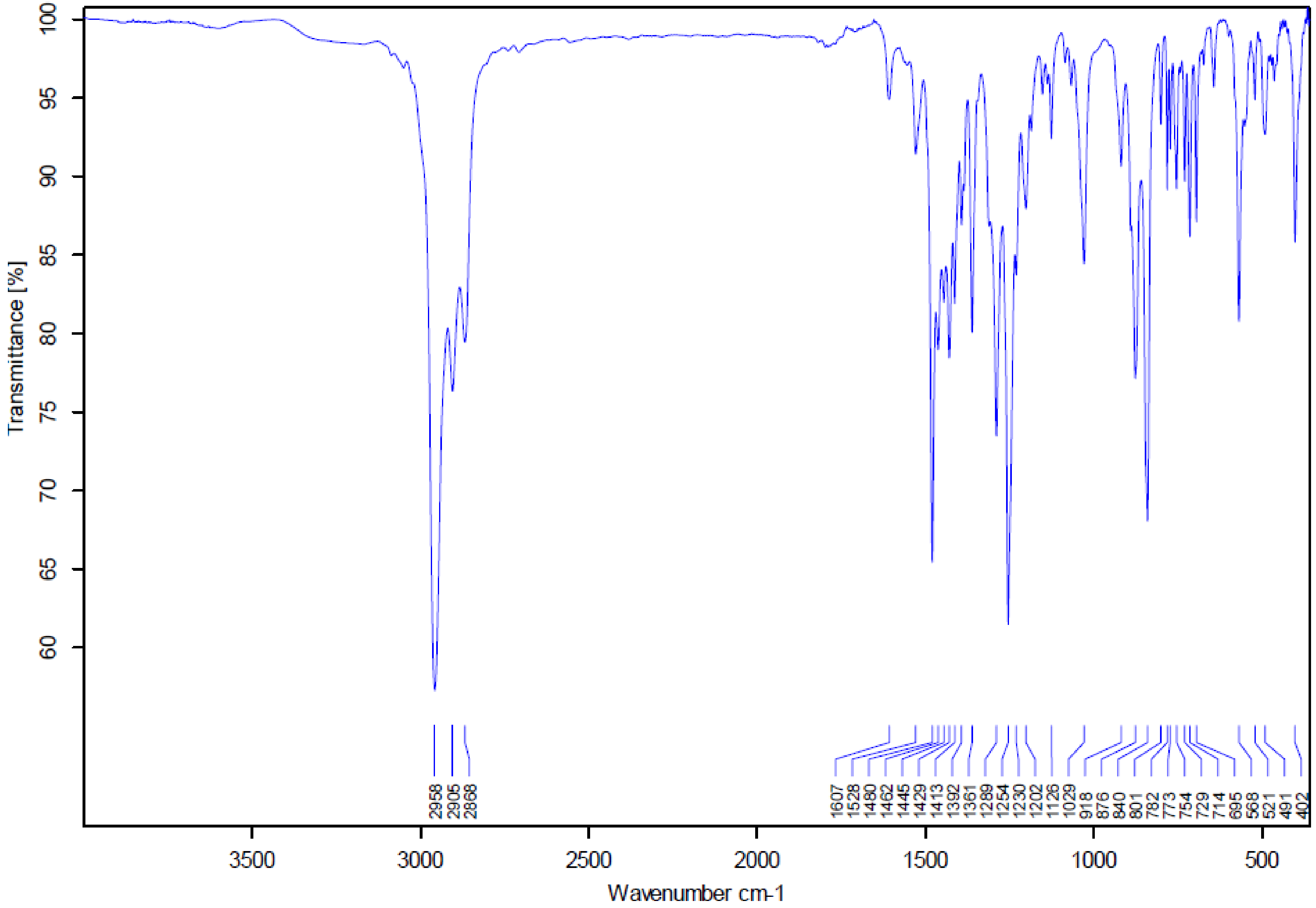

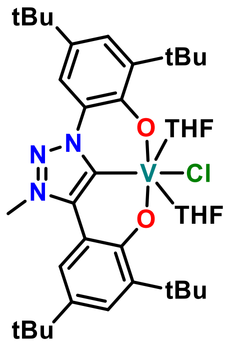

Figure S 56: ATR-IR spectrum of **6** at 298K

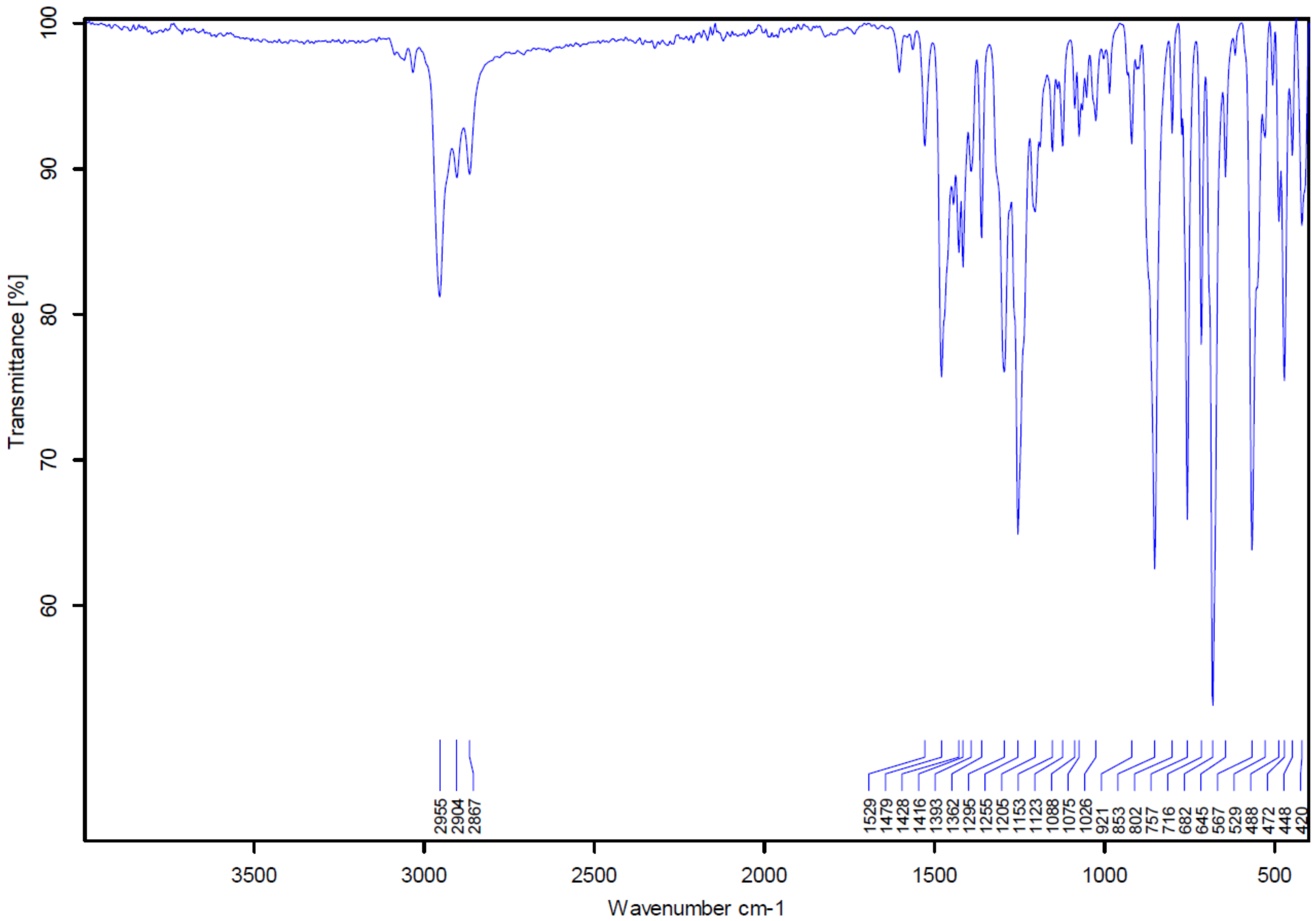

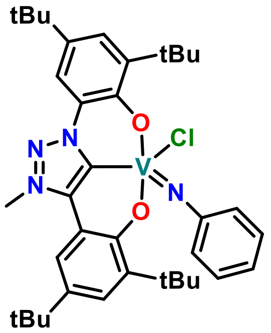

Figure S 57: ATR-IR spectrum of **7** at 298K

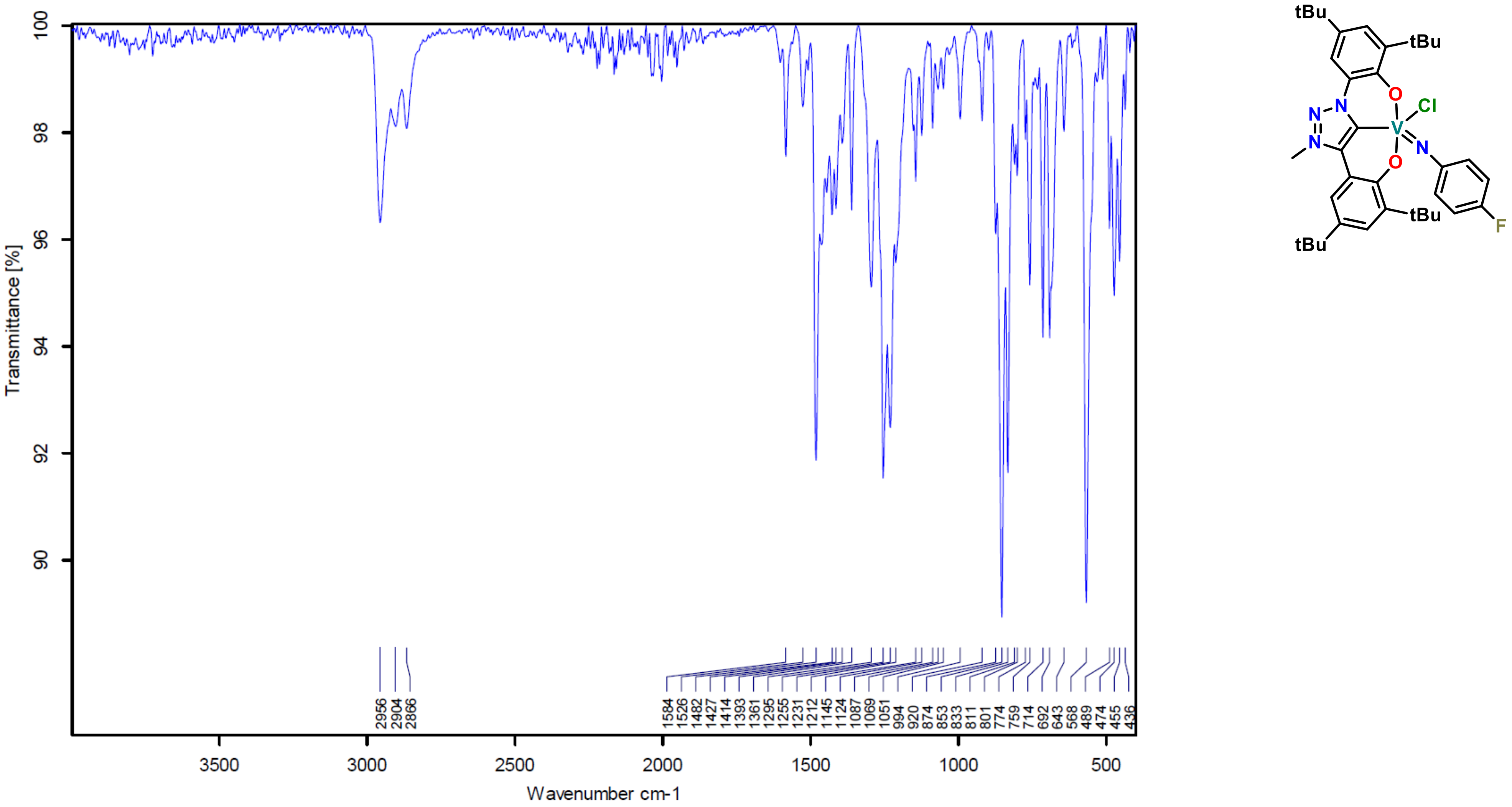

Figure S 58: ATR-IR spectrum of **8** at 298K

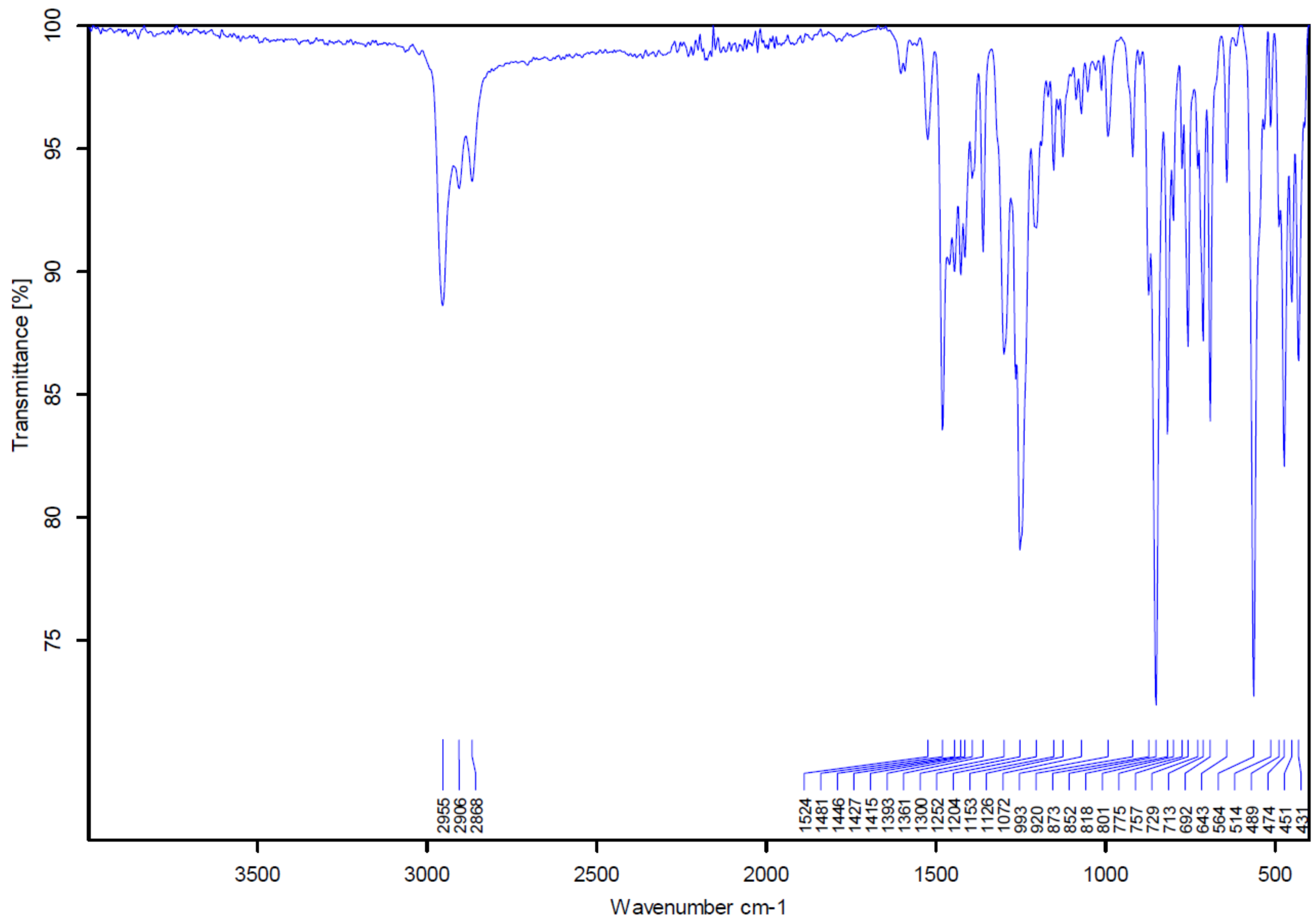

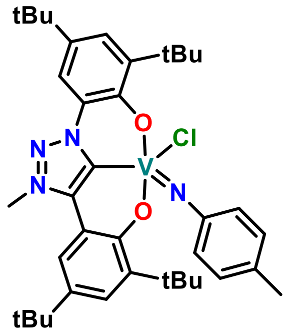

Figure S 59: ATR-IR spectrum of **9** at 298K

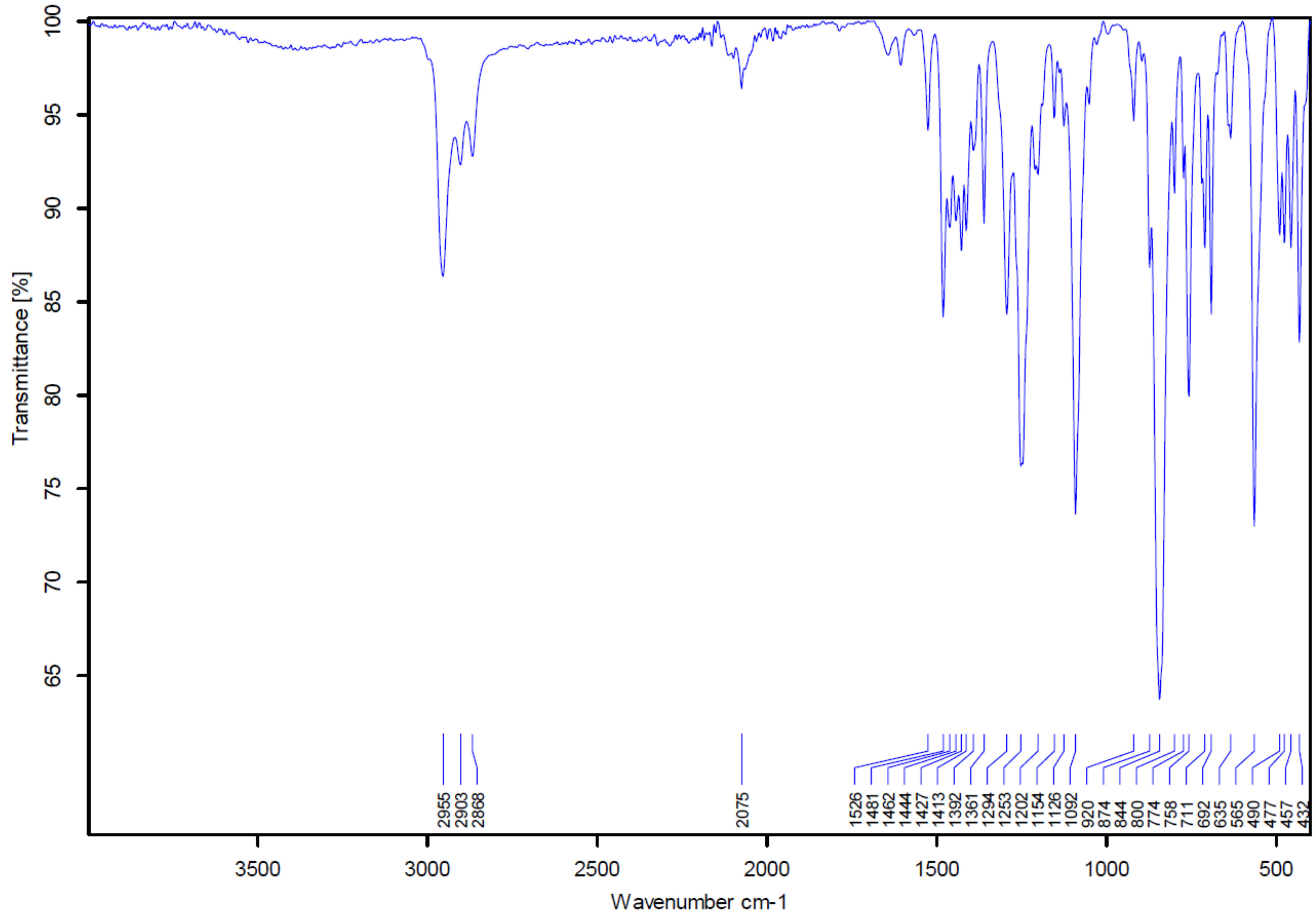

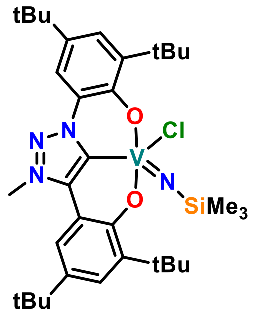

Figure S 60: ATR-IR spectrum of **10** at 298K

3. UV-Vis spectra

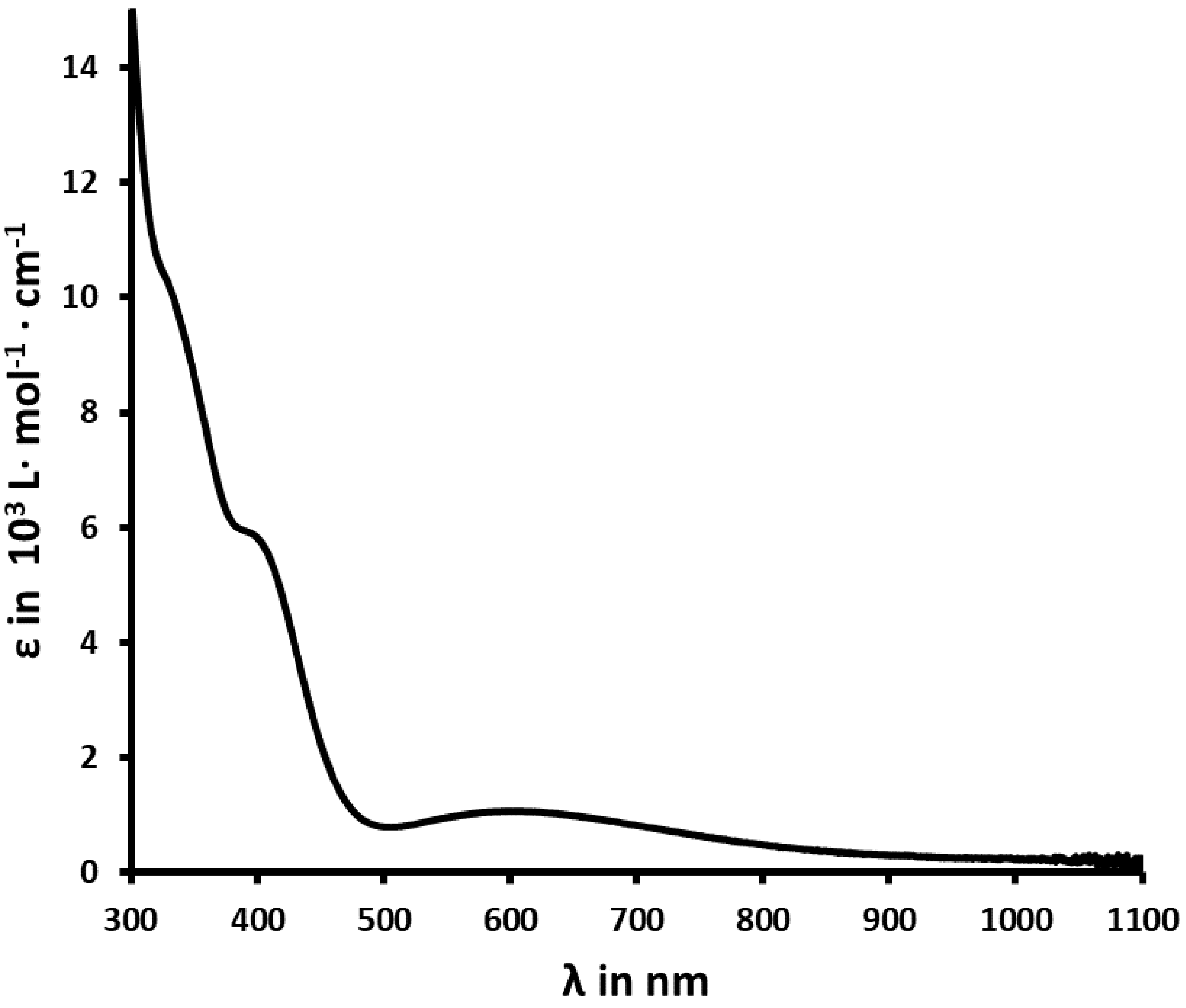

Figure S 61: UV-Vis of **1** in CH_2Cl_2 at 298K.

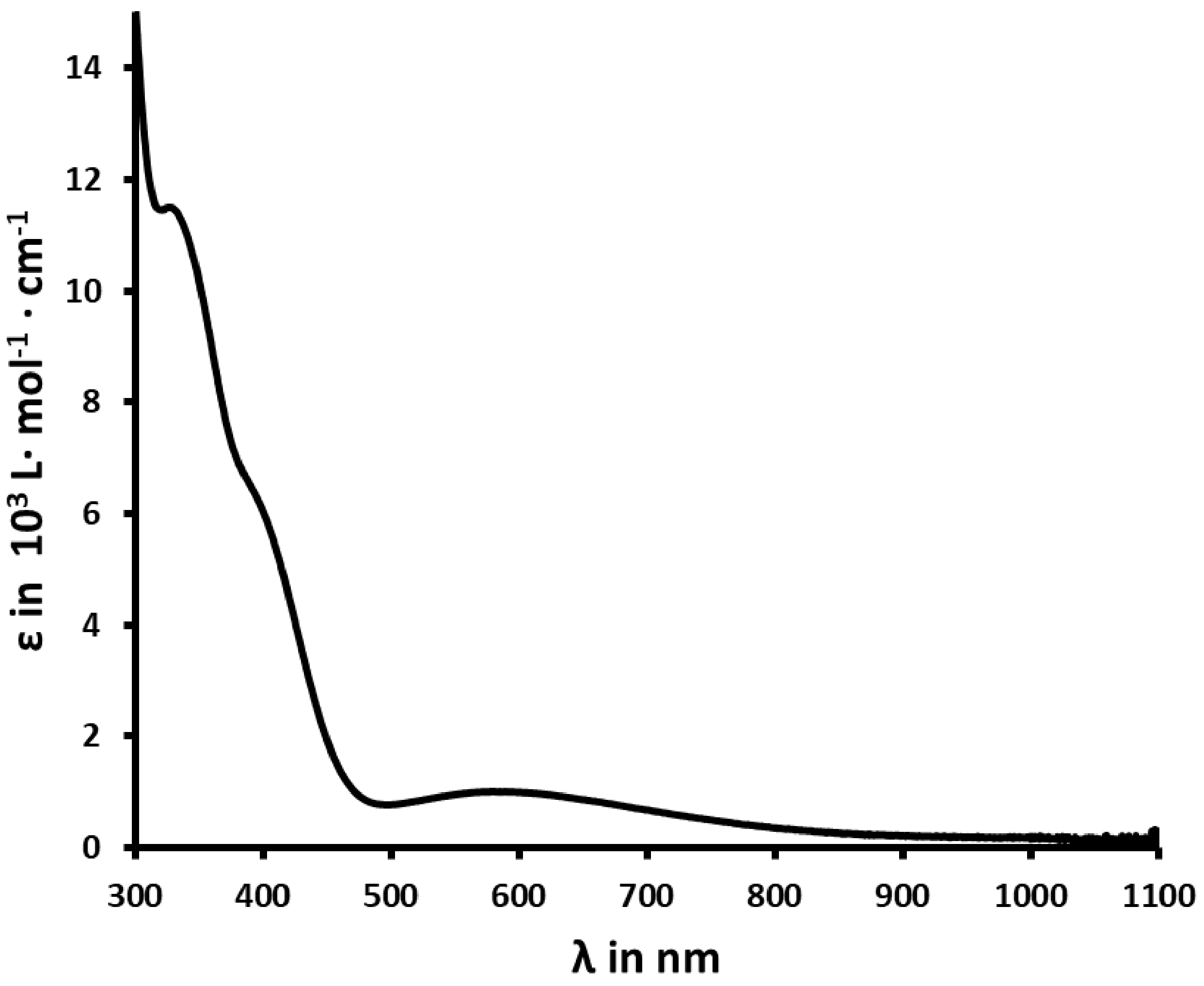

Figure S 62: UV-Vis of **1** in THF at 298K.

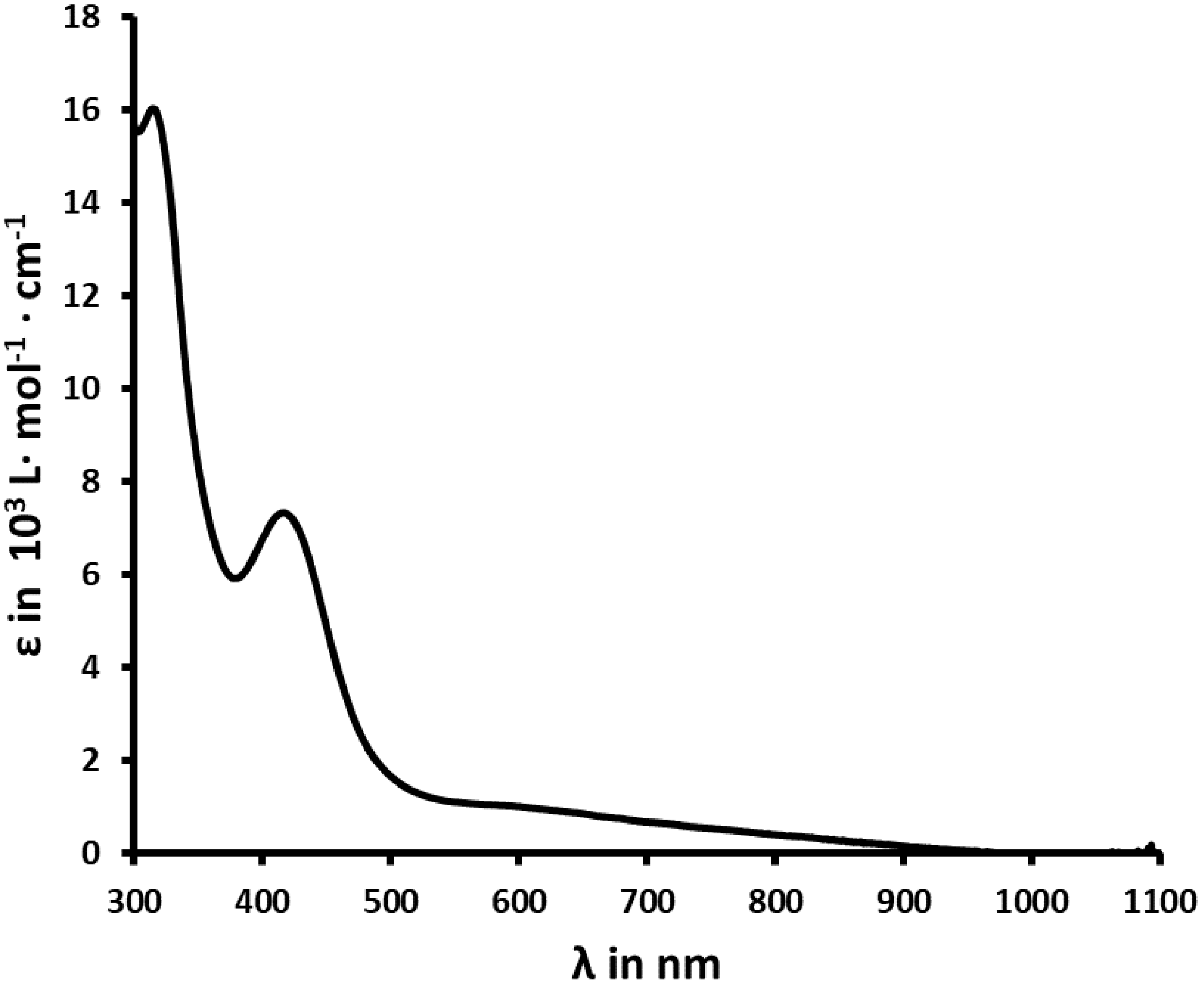

Figure S 63: UV-Vis of **2** in CH_2Cl_2 at 298K.

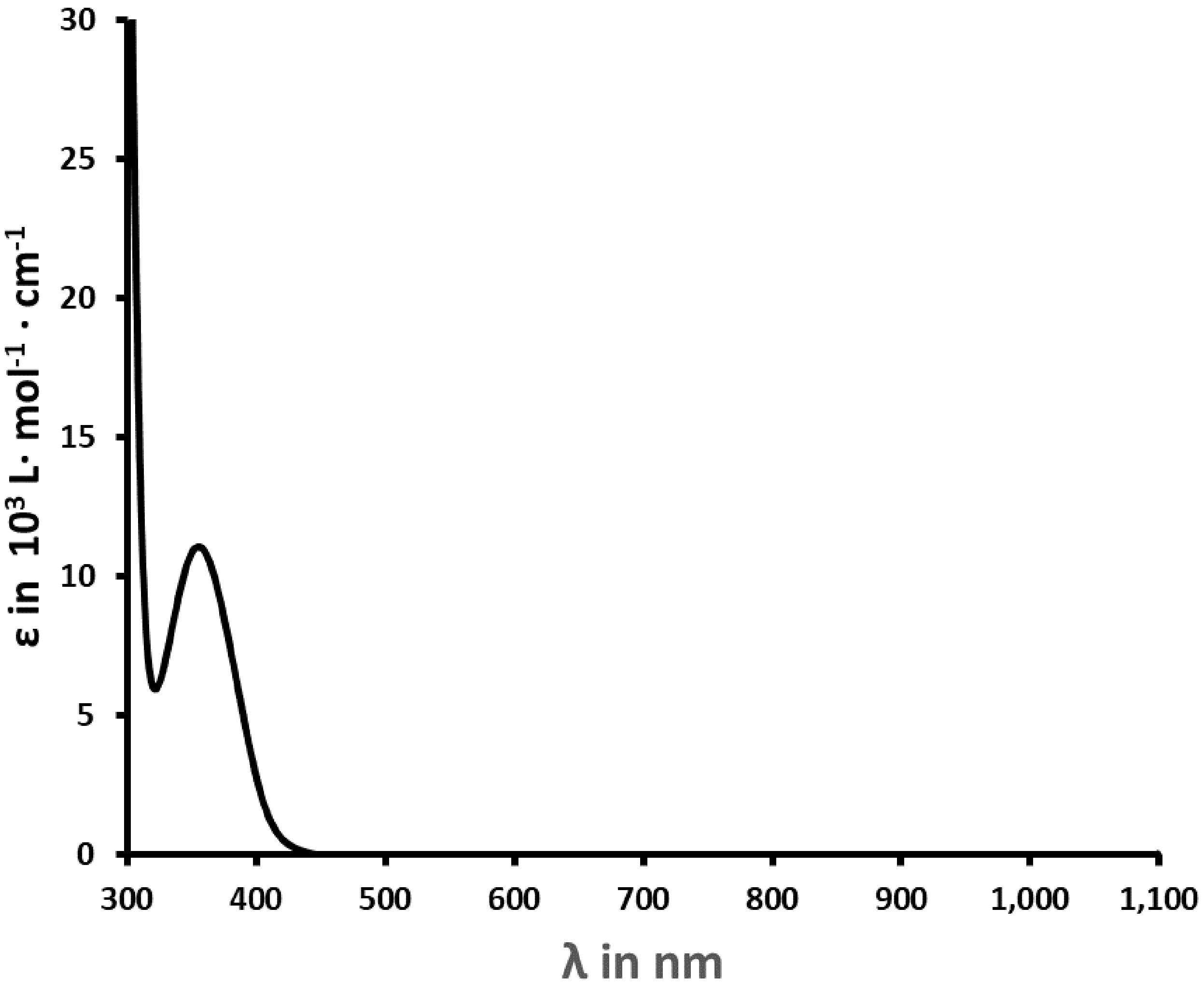

Figure S 64: UV-Vis of **[Co(Cp*)$_2$][1]** in CH_2Cl_2 at 298K.

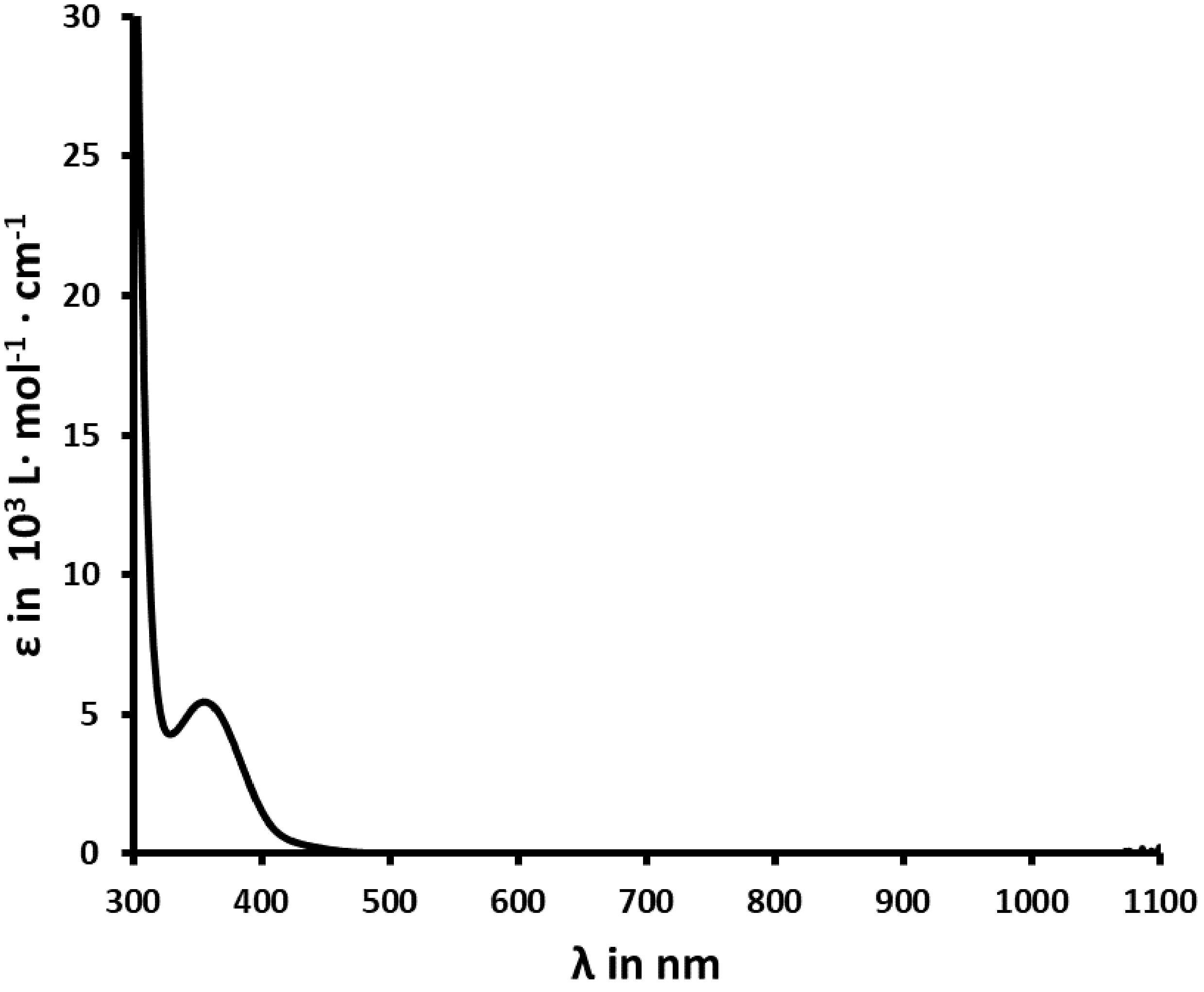

Figure S 65: UV-Vis of **[Co(Cp*)$_2$][2]** in CH_2Cl_2 at 298K.

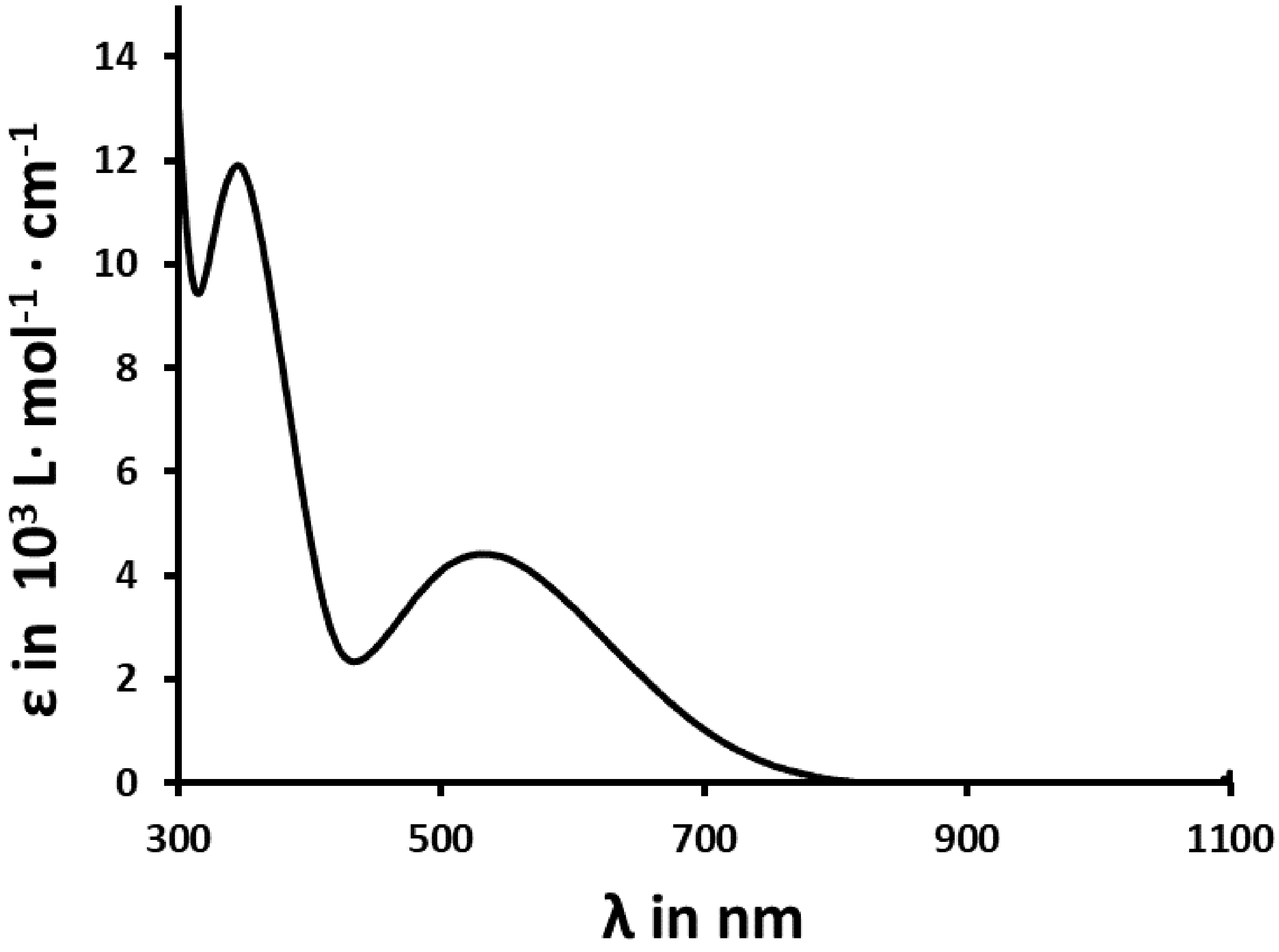

Figure S 66: UV-Vis of **4** in THF at 298K.

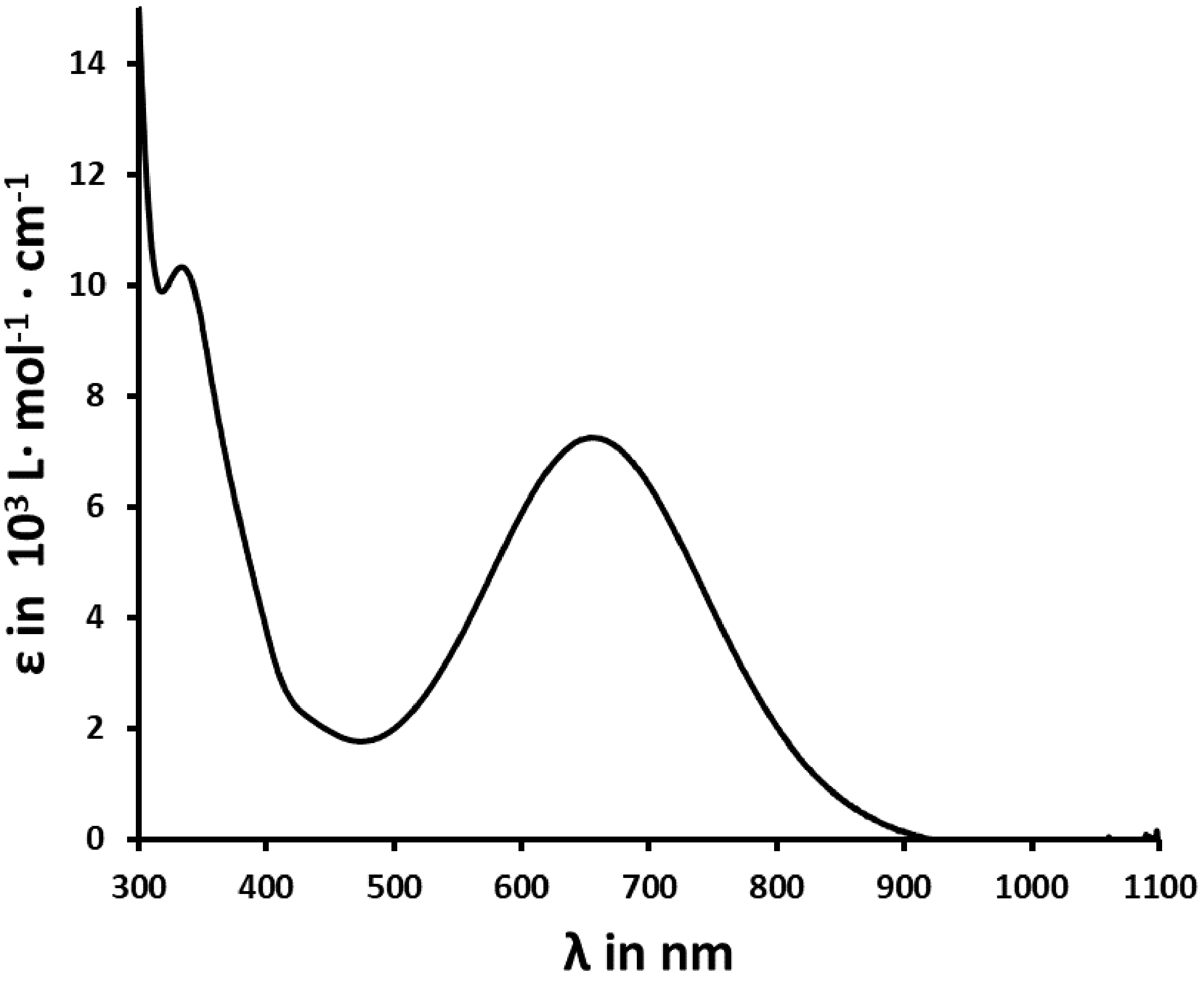

Figure S 67: UV-Vis of **5** in THF at 298K.

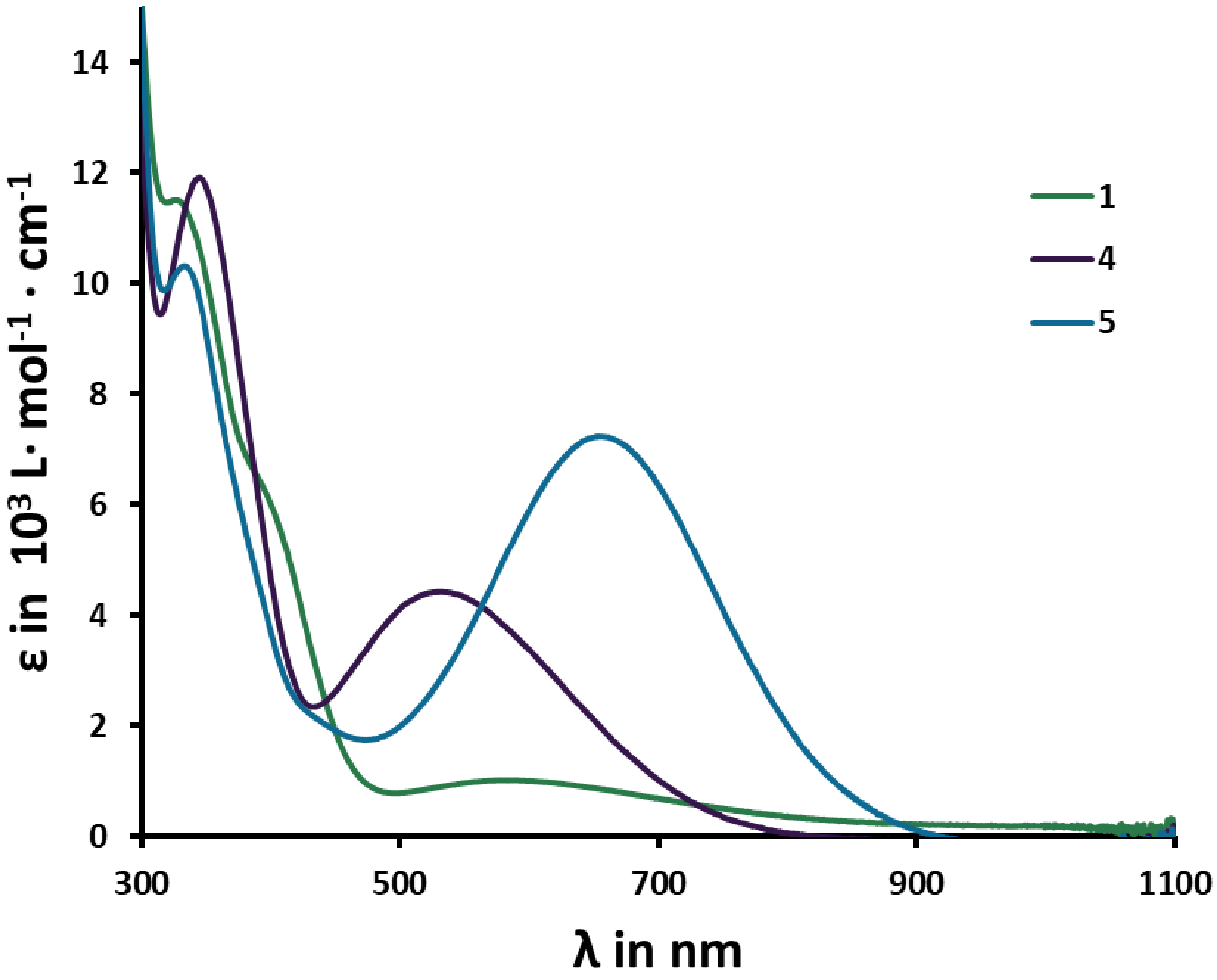

Figure S 68: Combined UV-Vis spectra for **1**, **4** and **5** in THF at 298 K.

4. Electrochemistry (Cyclic Voltammetry)

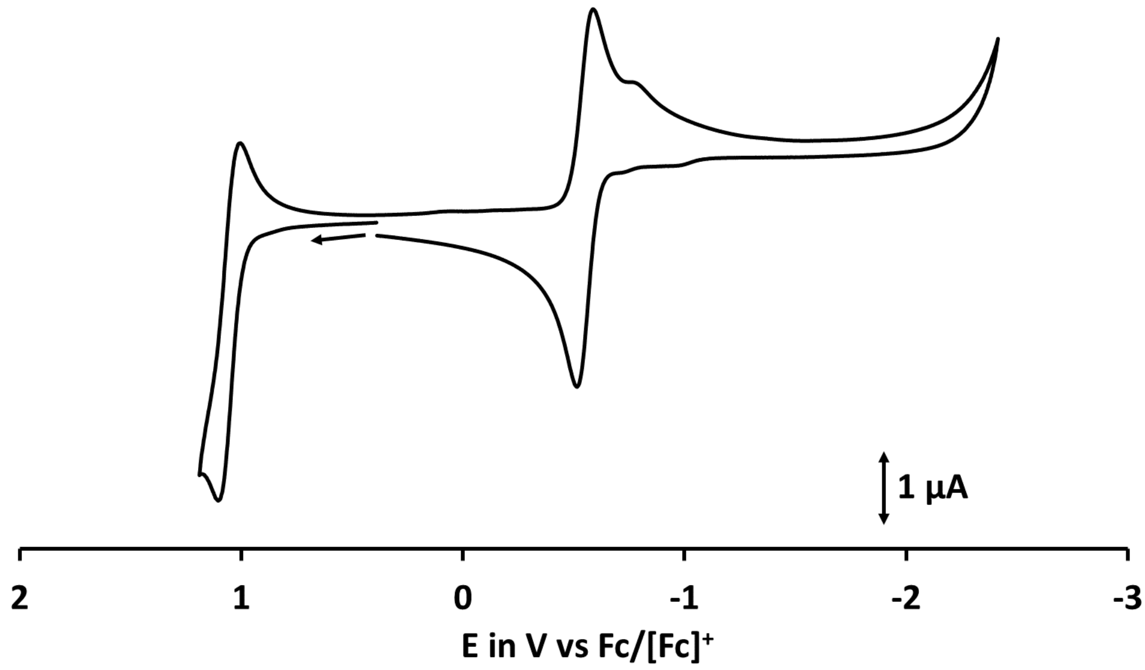

Figure S 69: Cyclic voltammogramm of **1** in 0.1 M NBu_4PF_6 in CH_2Cl_2 at 298K. Scan Rate 100 mVs^{-1}

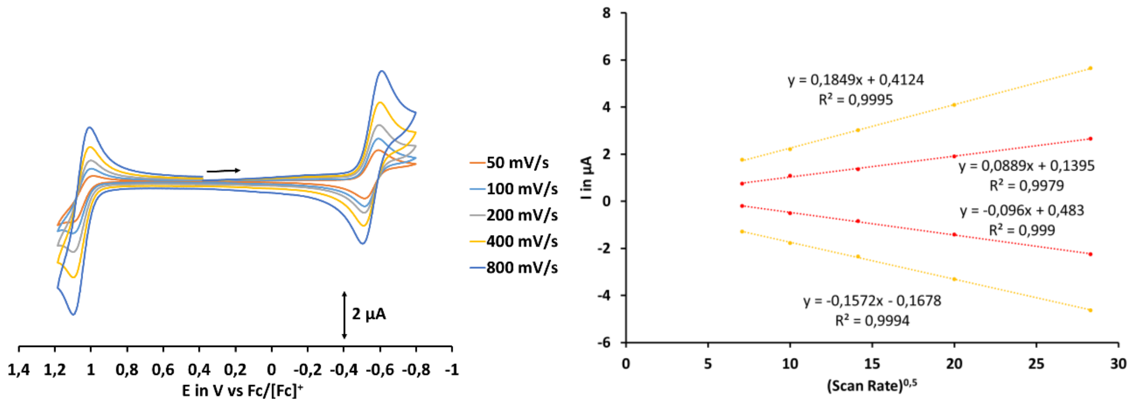

Figure S 70: Reversibility plots of the first reduction and the first oxidation of **1** Please note that the yellow lines in the left diagram belong to the first oxidation, while the red lines belong the first reduction process.

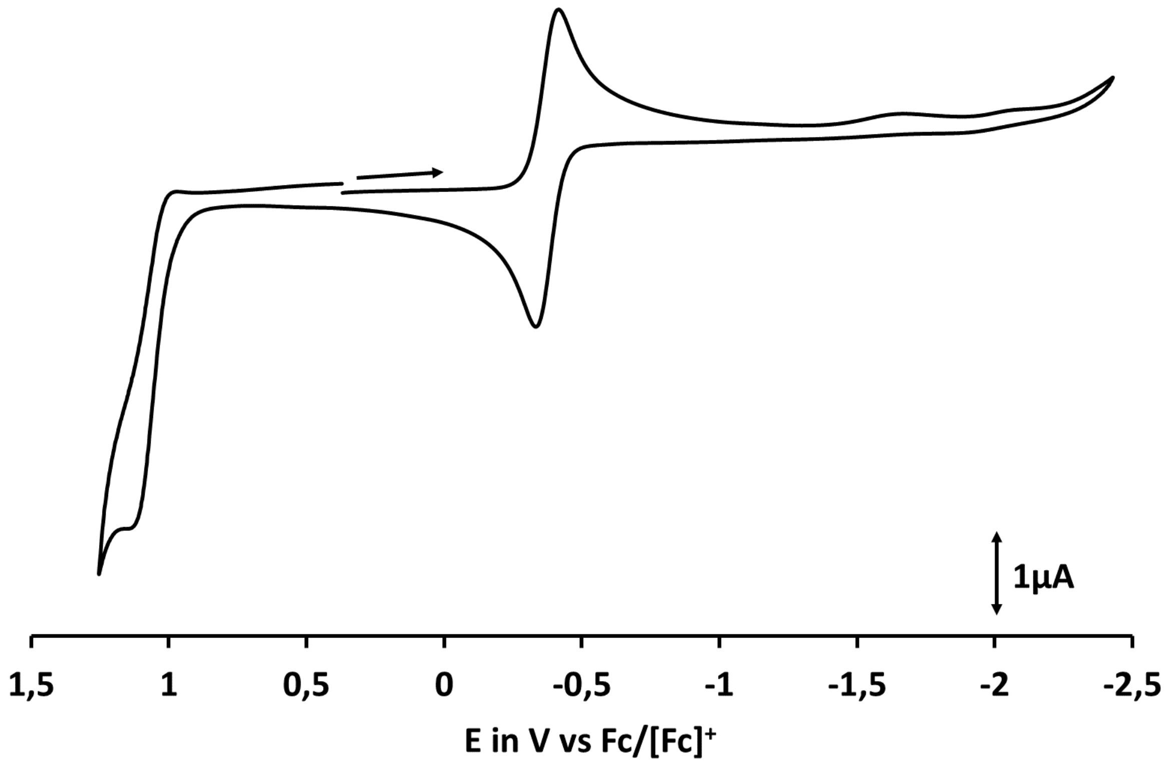

Figure S 71: Cyclic voltammogramm of **2** in 0.1 M NBu_4PF_6 in CH_2Cl_2 at 298K. Scan Rate 100 mVs^{-1}

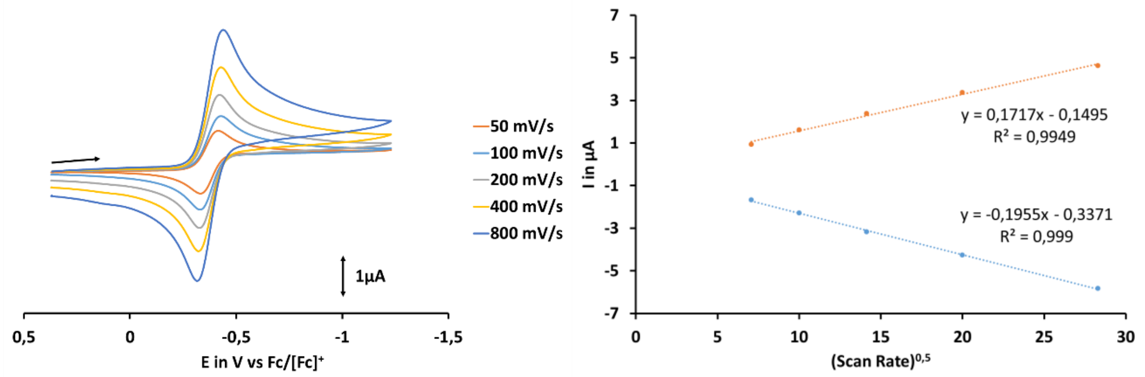

Figure S 72: Reversibility plots of the first reduction in **2**.

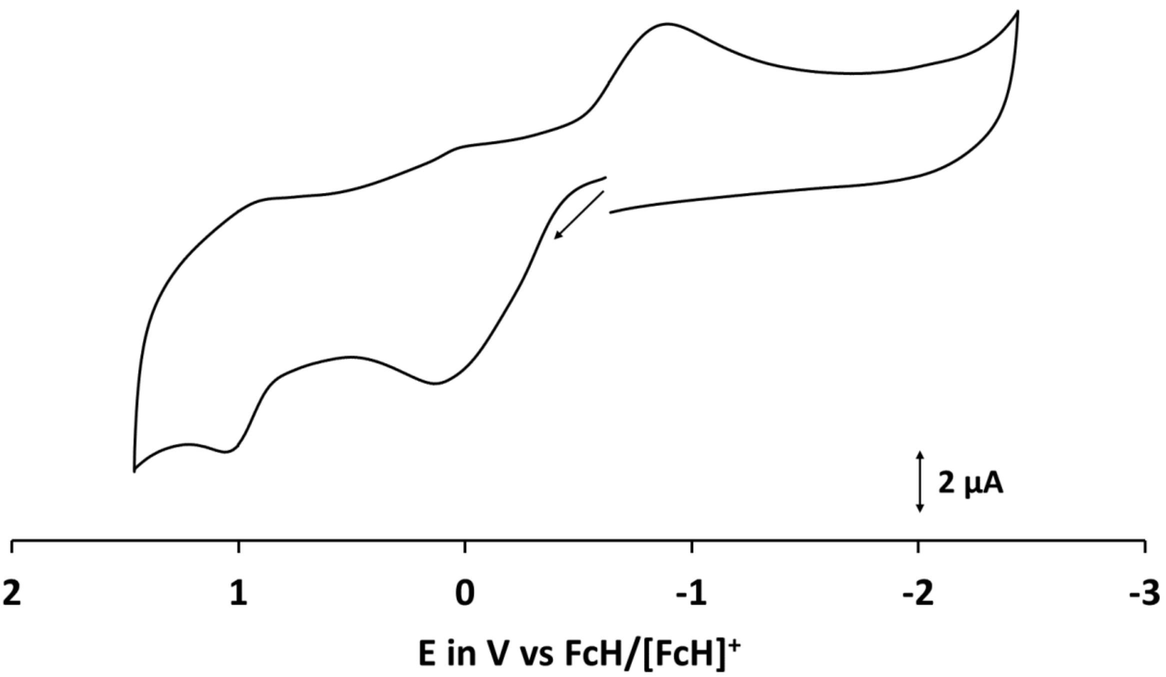

Figure S 73: Cyclic voltammogramm of **6** in 0.1 M NBu_4PF_6 in MeCN at 298K. Scan Rate 100 mVs^{-1}

5. Quantum Chemical Calculations

All calculations were performed with ORCA v. 4.2.1.[1] The geometric parameters of **1**, **2**, as obtained from the solid-state structures, were optimized at the ZORA-PBE-D3BJ/ZORA-def2-SVP level of theory.[2] For vanadium, the all-electron ZORA-def2-TZVP basis set was used.[3] Scalar relativistic effects were modeled with the "Zeroth Order Regular Approximation" (ZORA).[4] The RI approximation with the related auxilary basis set SARC/J was used to speed up the calculations.[5] Tighter-than-default scf ("*tightscf*") and optimization criteria ("*tightopt*") were chosen in conjunction with finer-than-default grid values ("*grid6*"; "*finalgrid7*"). All calculated structures were verified as true minima by the absence (N^{imag} = 0) of negative eigenvalues in the harmonic vibrational frequency analysis. In case of compounds **2**, two conformers were each obtained, whereas the calculations converged to only one minimum for compounds **1**. TD-DFT calculations were performed to model the experimental UV-Vis spectra. 50 roots were calculated at the ZORA-PBE/ZORA-def-TZVPP//ZORA-PBE-D3BJ/ZORA-def2-SVP as well as ZORA-PBE0/ZORA-def-TZVPP//ZORA-PBE-D3BJ/ZORA-def2-SVP level of theory. For the PBE0 functional, the chain of spheres approximation (COSX) was used with finer than default grid value (gridx4). The TD-DFT calculations were performed using the Tamm-Dancoff approximation; the weight of the orbitals given below to illustrate the nature of the calculated absorption bands was found to be (typically considerably) larger than 0.75.

Table S 1: Energies of calculated compounds; values are given in [Eh].

	E(SVP)	*G*(SVP)	*E*(TZVPP)	*E*(PBE0, TZVPP)
**1^{-}_d**	-3009.91345		-3011.74072	3011.87911
**1_s**	-3009.84908	-3009.23916	-3011.67189	-3011.78633
**1_t**	-3009.78805		-3011.61206	-3011.73874
**2^{-}_d**	-3108.09705	-3107.4607	-3110.01755	-3110.17175
**2^{-}_isomer_d**	-3108.10613	-3107.47197	-3110.02848	-3110.18464
**2_t**	-3107.97446	-3107.33828	-3109.89223	-3110.03536
**2_isomer_t**	-3107.98012	-3107.34394	-3109.89886	-3110.04327
**2_s**	-3108.02928	-3107.38985	-3109.94584	-3110.07388
**2_isomer_s**	-3108.03519	-3107.39575	-3109.95238	-3110.08265
**9_q**	-3398.97129	-3398.15215	-3398.15215	-3401.39744
**9_t**	-3399.05277	-3398.22861	-3401.27253	-3401.48935
**9_s**	-3399.03494	n.a.	-3401.25527	-3401.4498
**9_s_UKS**	-3399.0423	n.a.	-3401.26217	-3401.44772

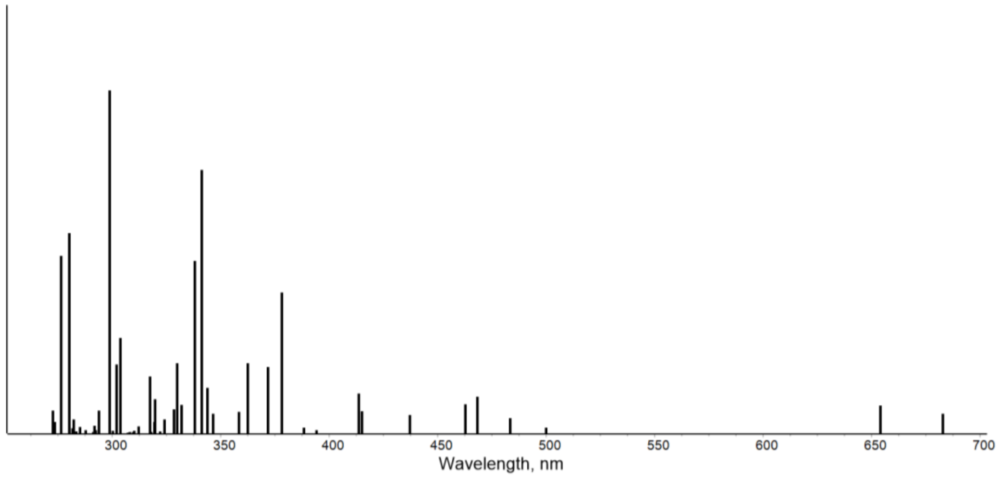

Figure S 74: Calculated (TD-DFT, PBE) transitions for **1**.

LMCT (MO156 → MO157), 683 nm LMCT

LMCT (MO155 → MO157), 654 nm LMCT

LLCT (MO156 → MO158), 483 nm LLCT

LLCT (MO155 → MO158), 468 nm LLCT

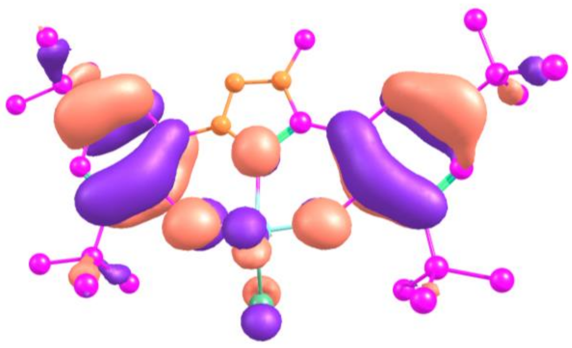

MO155

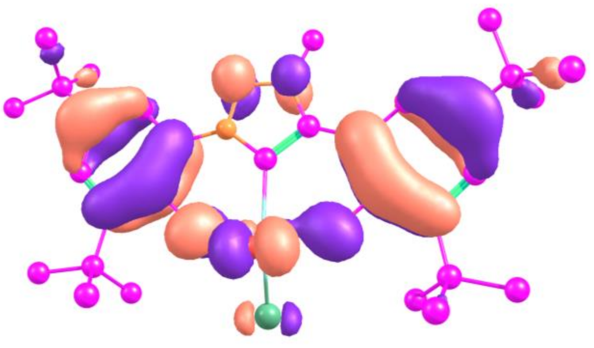

MO156

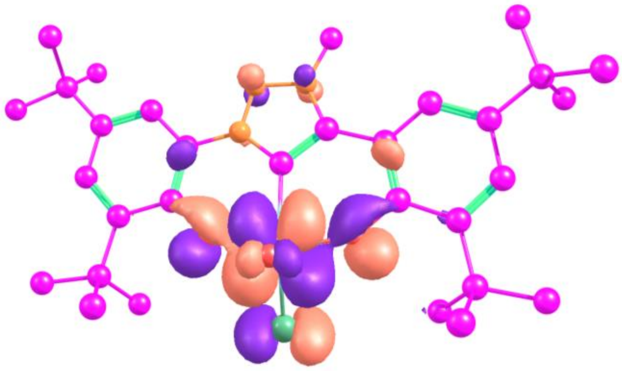

MO157

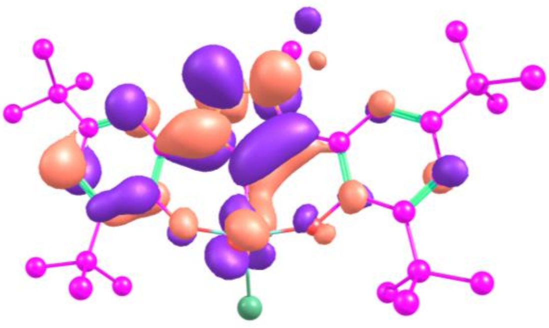

MO158

Figure S 75: Molecular orbitals associated with calculated (TD-DFT, PBE) transitions for **1**.

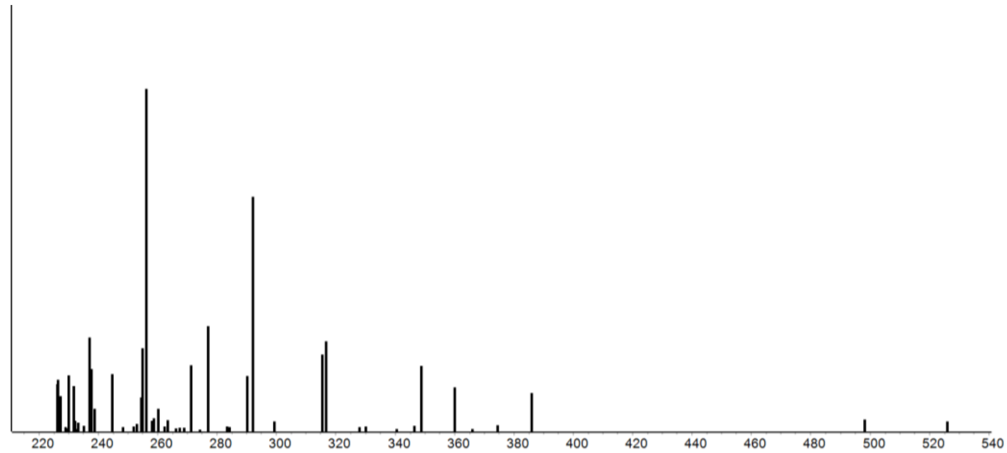

Figure S 76: Calculated (TD-DFT, PBE0) transitions for **1**.

LMCT (MO156 → MO157), 526nm LMCT

LMCT (MO155 → MO157), 498 nm LMCT

LLCT (MO156 → MO158), 386 nm LMCT

LLCT (MO155 → MO158), 357 nm LMCT

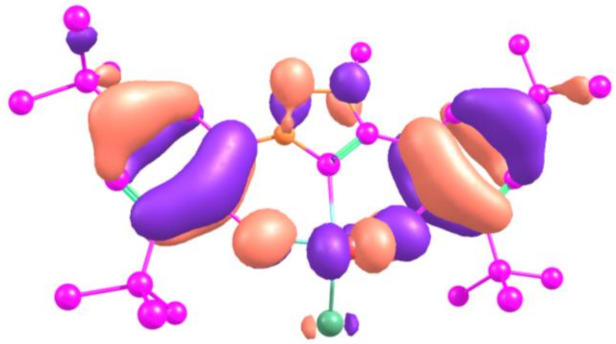

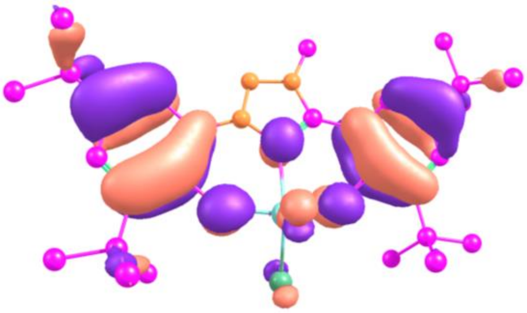

MO155 **MO156**

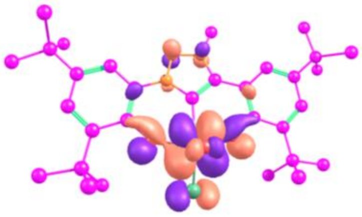

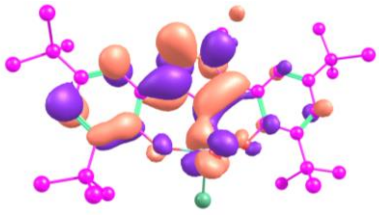

MO157 **MO158**

Figure S 77: Molecular orbitals associated with calculated (TD-DFT, PBE0) transitions for **1**.

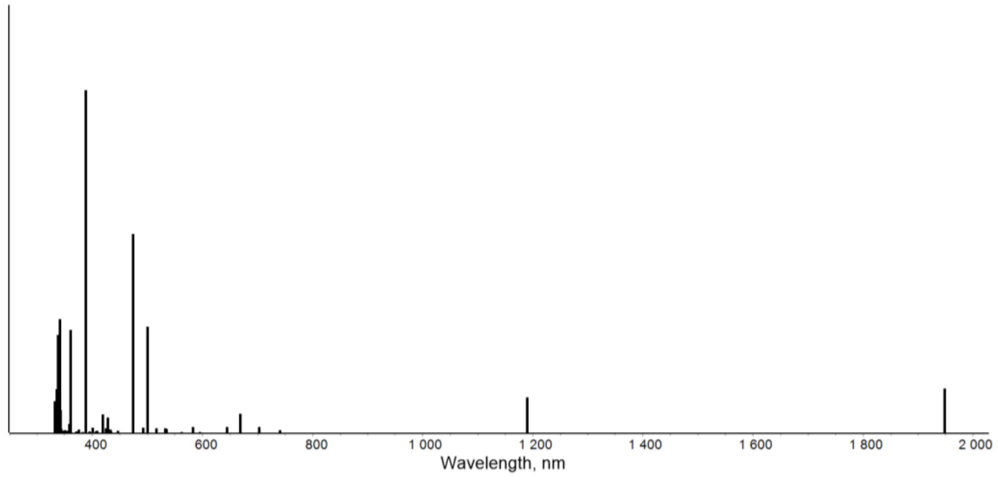

Figure S 78: Calculated (TD-DFT, PBE) transitions for **1-**.

LLCT (MO155 → MO158), 500 nm

LLCT (MO156 → MO158), 473 nm

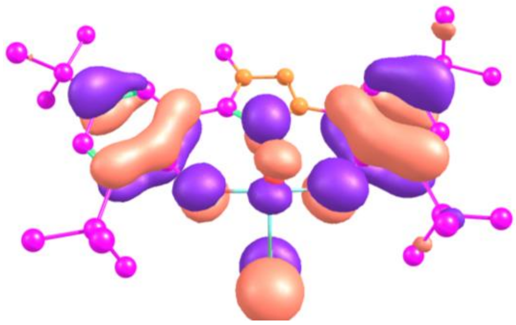

MO155

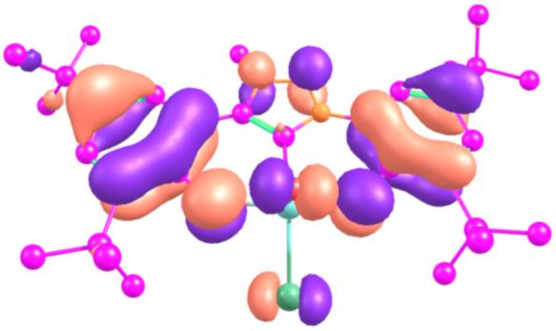

MO156

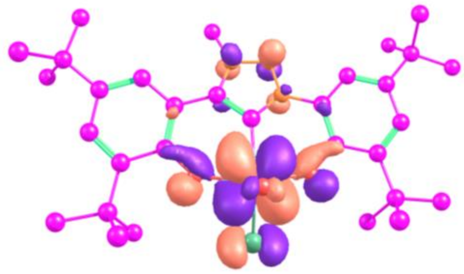

MO157 (SOMO)

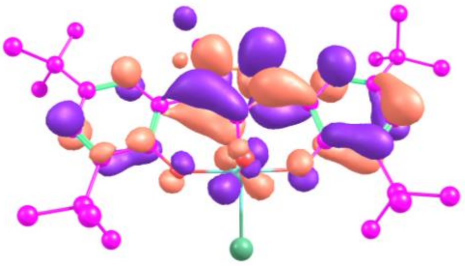

MO158

Figure S 79: Molecular orbitals associated with calculated (TD-DFT, PBE) transitions for **1**$^{-}$.

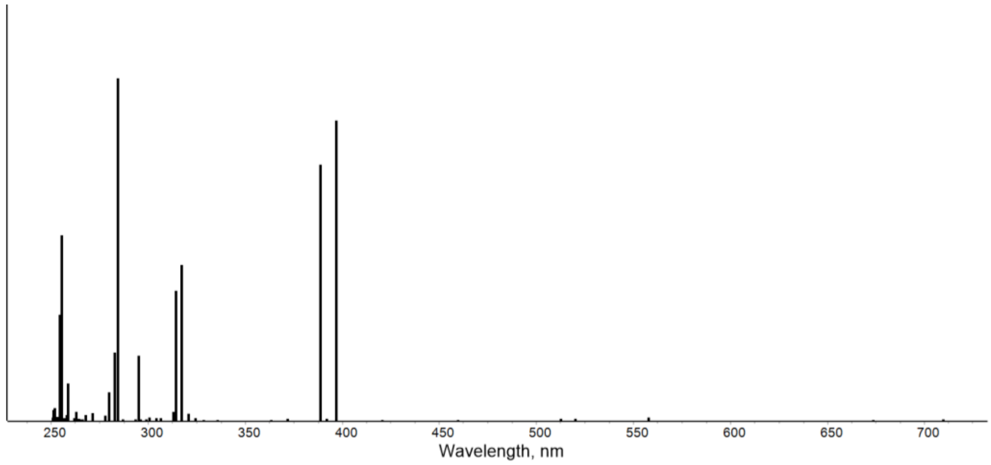

Figure S 80: Calculated (TD-DFT, PBE0) transitions for **1**$^{-}$.

LLCT (MO156 → MO158), 386 nm

LLCT (MO155 → MO158), 357 nm

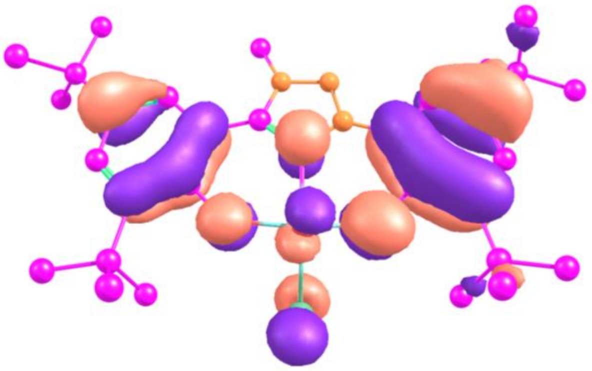

MO155

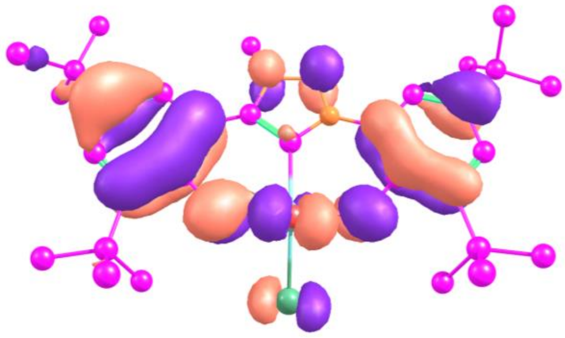

MO156

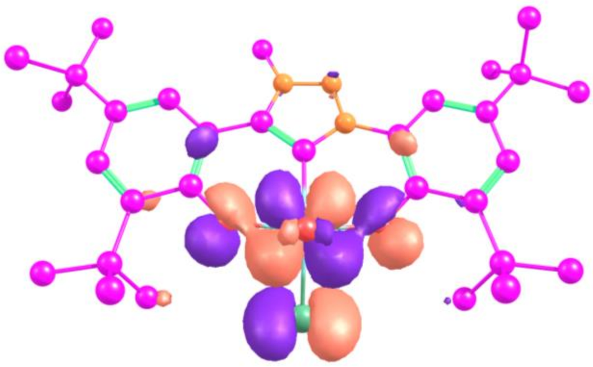

MO157 (SOMO)

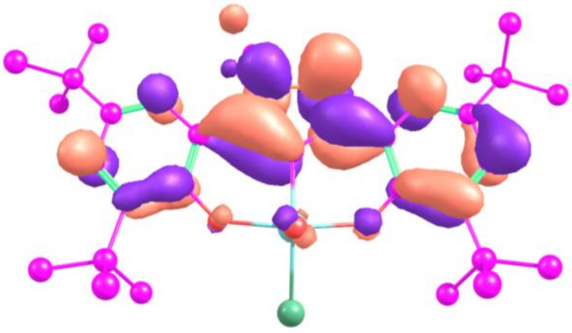

MO158

Figure S 81: Molecular orbitals associated with calculated (TD-DFT, PBE0) transitions for **1**$^{-}$.

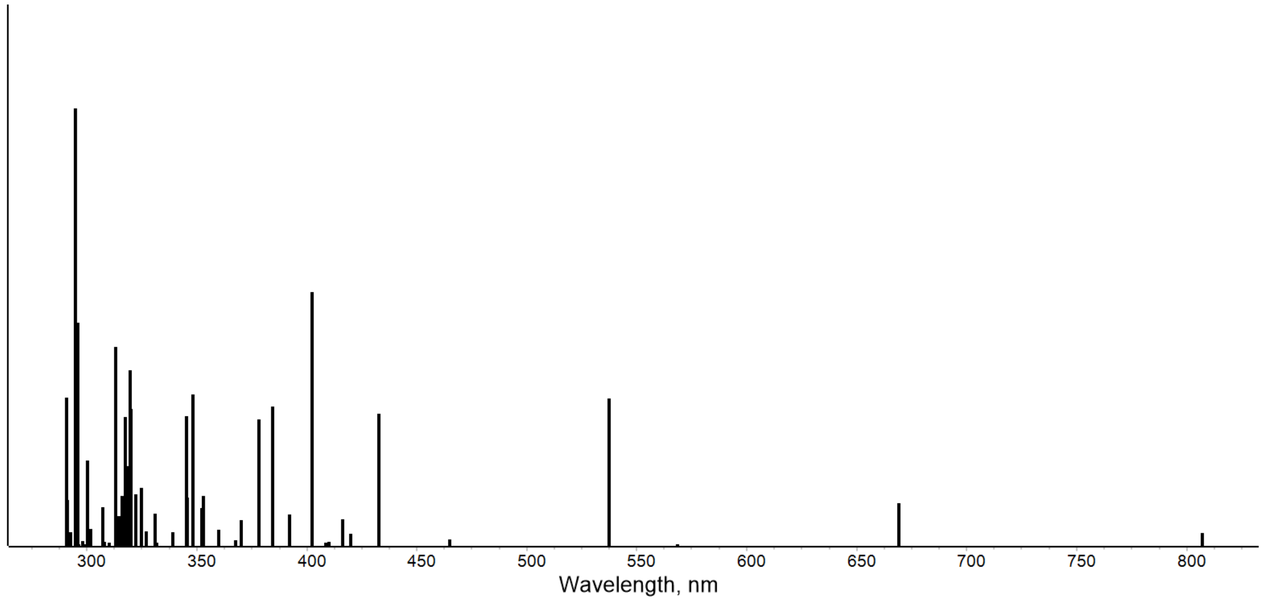

Figure S 82: Calculated (TD-DFT, PBE) transitions for **2**.

LMCT (MO166 → MO167), 807 nm

LMCT (MO165 → MO167), 669 nm

LLCT (MO166 → MO168), 537 nm

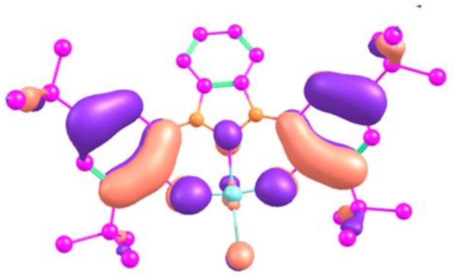

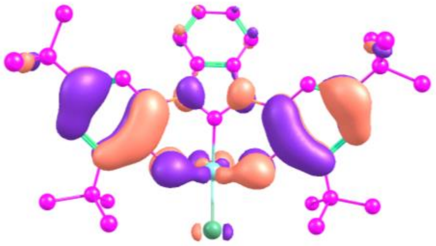

MO165 **MO166**

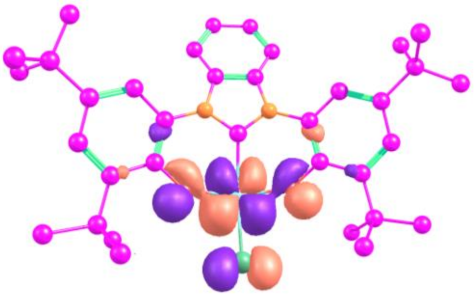

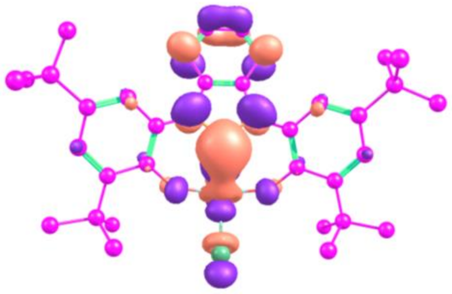

MO167 **MO168**

Figure S 83: Molecular orbitals associated with calculated (TD-DFT, PBE) transitions for **2**.

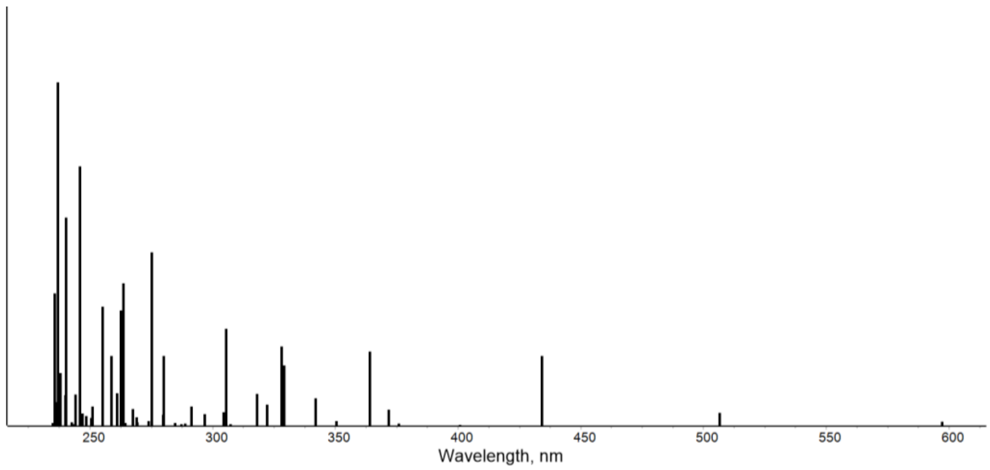

Figure S 84: Calculated (TD-DFT, PBE0) transitions for **2**.

LMCT (MO166 → MO167), 597 nm LMCT

LMCT (MO165 → MO167), 506 nm LMCT

LLCT (MO166 → MO168), 434 nm LLCT

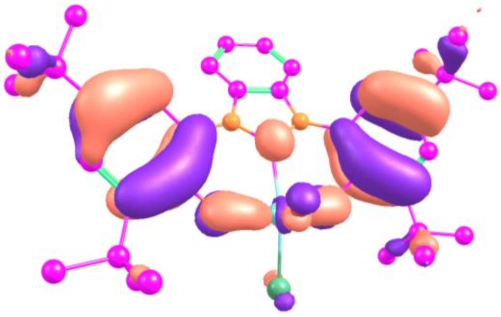

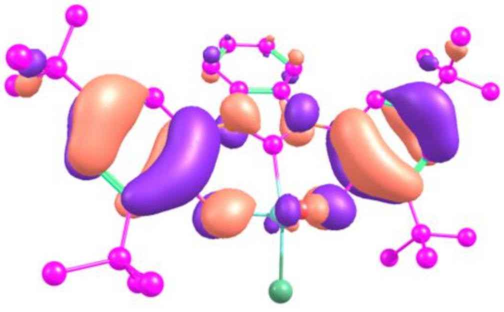

MO165 **MO166**

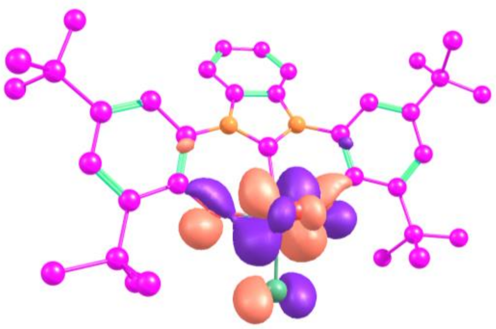

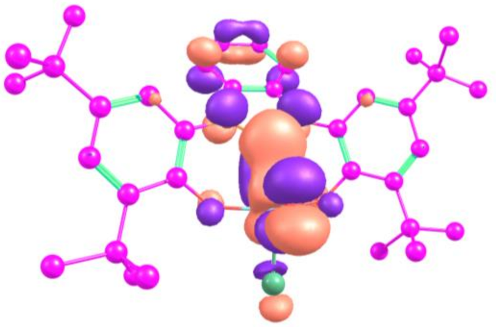

MO167 **MO168**

Figure S 85: Molecular orbitals associated with calculated (TD-DFT, PBE0) transitions for **2**.

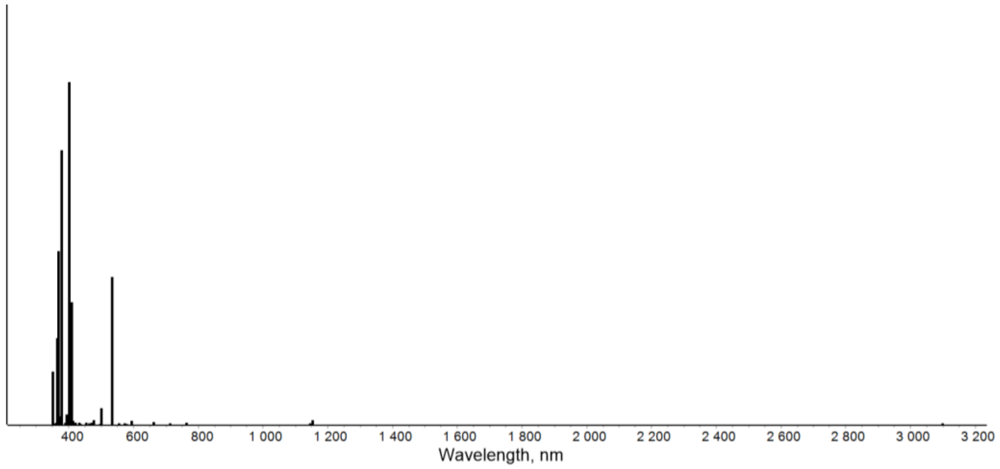

Figure S 86: Calculated (TD-DFT, PBE) transitions for **2**$^-$.

LLCT (MO165 → MO167), 532 nm

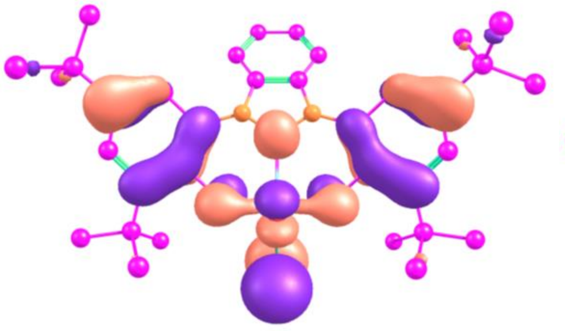

MO165

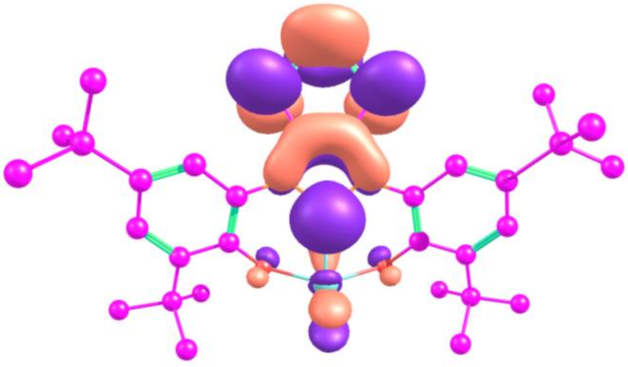

MO167

Figure S 87: Molecular orbitals associated with calculated (TD-DFT, PBE) transitions for **2**$^{-}$.

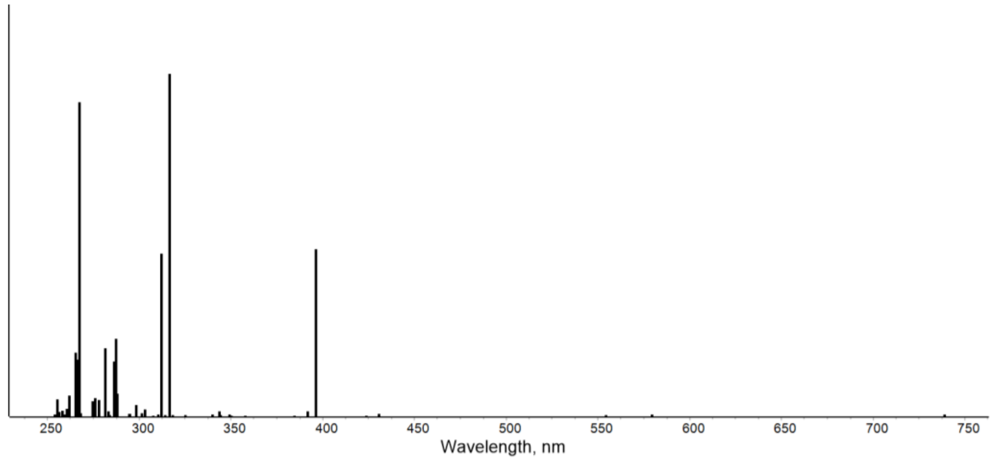

Figure S 88: Calculated (TD-DFT, PBE0) transitions for **2**$^{-}$.

LLCT (MO165 → MO167), 396 nm

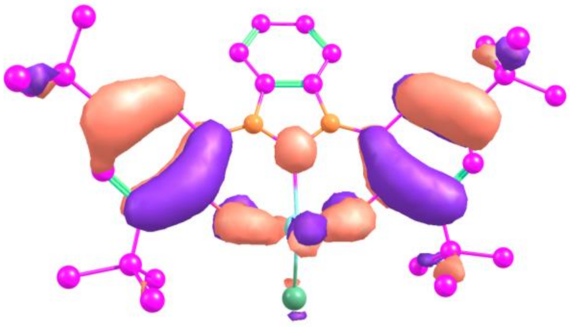

MO165

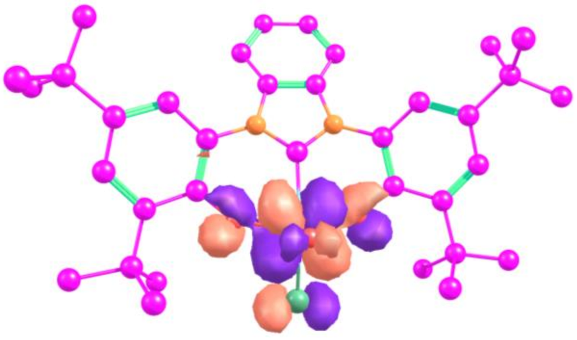

MO166

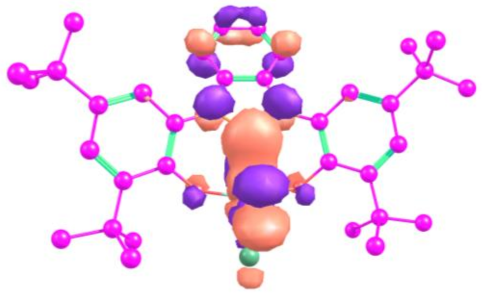

MO176

Figure S 89: Molecular orbitals associated with calculated (TD-DFT, PBE0) transitions for **2**$^{-}$.

XYZ Coordinates

1⁻_d

V	3.36031568666981	15.41141833953448	12.11578300482809
O	3.50131634989984	14.75983172769659	10.27949774355623
N	5.99003923719238	13.91933919676474	11.31743988702488
C	5.88469522685000	14.11346890057776	13.50019389355470
C	5.19456603051946	14.44592386534269	12.31561165241519
C	5.47987997429701	14.42118666773571	14.86142913763688
C	4.12918190011037	14.90252425731538	15.03913425355397
C	3.67084687065672	15.19195460505227	16.37884642178260
C	4.45376660594690	14.40584637724942	9.46720162779355
C	5.74117775810144	13.96462873168490	9.91940120364776
C	6.33129318962552	14.28921291734943	15.98805722352480
H	7.36796507878097	13.98524312495264	15.83831047140159
C	4.23341445359935	14.42191412007491	8.04232892975002
C	4.55971217307814	15.01189267145841	17.44118474216059
H	4.20703090378915	15.23411600497886	18.45461949820884
N	7.01988447858215	13.42882344248854	13.08957170456021
C	1.20743586699852	14.68710833958696	16.04288129208206
H	1.30205025320645	14.56635616559817	14.95108874982593
H	0.18101097056478	15.03792121517415	16.26362013377016
H	1.34188474440412	13.70126517225815	16.52646455165390
C	2.23542439582477	15.70651820507623	16.59526440542105
C	2.87873158517977	14.91484954156691	7.50026717297828
N	7.09627825444031	13.30905663712113	11.75563743845057
C	6.74289110519667	13.54102716671266	9.03034479947689
H	7.69389914108935	13.20683683368108	9.45863952767161
C	1.72647337776531	14.03949213249875	8.05524599064278
H	1.88108483645370	12.97864653075860	7.78268083880734
H	0.76453769155663	14.36770013292387	7.61653169530197
H	1.65152106114623	14.11777030588887	9.15232465591067
C	5.89945751538395	14.57478632805354	17.28463634366801
C	1.92604140770994	15.92205146055028	18.08994191544628
H	2.00935900570238	14.98369072913324	18.66991402419105
H	0.88902950212307	16.28916442348993	18.19662621482544
H	2.59358261142158	16.67440414406059	18.55027751071207
C	6.52526492780425	13.54647126578226	7.64737663632358
C	5.26228536746205	13.99364441892965	7.19207251535837
H	5.07488158627850	14.01012465778314	6.11552917854342
C	6.80685930419476	14.44431640464213	18.52016601000289
C	2.66226460280803	16.39027262728386	7.92030614680284
H	2.67282900333565	16.49977168713380	9.01618703433256
H	1.68599838365600	16.75060446863723	7.54378492126080
H	3.45418693067696	17.03419019650941	7.49488060268135
C	2.06091762496735	17.06692778359017	15.87487012554292
H	2.76127087459365	17.81666969227456	16.28751060243172
H	1.02984242073763	17.44126101842542	16.02113060063991
H	2.24439062829780	16.97330671523710	14.79317722182791
C	8.22353452364801	13.96381479278450	18.15534989613310
H	8.20396252044454	12.97026164668753	17.67117087009638
H	8.84174825674334	13.87982930493593	19.06816041689356
H	8.72924816743911	14.66985086225103	17.47175700768370
C	6.19187434828129	13.42313115528661	19.50614391379289
H	5.18014154325029	13.72929391460590	19.82466584556901
H	6.81844995012950	13.32224169189409	20.41346815173639

H	6.10390213571824	12.42911470474391	19.03174940118856
C	8.89224248273452	13.97115760475612	6.89215989017490
H	8.65592942158753	15.02652131326994	6.66645839959086
H	9.71622916049561	13.64841169097356	6.22656788827599
H	9.25842201839082	13.92809465324393	7.93277515642192
C	7.64345373483344	13.07975163077696	6.69713028066163
C	7.22290510273390	13.15899690524668	5.21798407011114
H	6.34568221534044	12.52073715780588	5.00870070541037
H	8.05199161005285	12.81496114445743	4.57219707740944
H	6.97102387737606	14.19280086191551	4.92047868963751
C	2.82036000561694	14.84915815001514	5.96156718984878
H	3.59506990075455	15.48072099058428	5.48802534086640
H	1.83593671004415	15.21734425005356	5.61938155423594
H	2.93903149283883	13.81563210160958	5.58571991052925
C	8.01090059561022	11.61153494896495	7.01617568397247
H	8.34938626917840	11.49976021675045	8.06111278721584
H	8.82311266593676	11.25424598671500	6.35371524916899
H	7.13480843088100	10.95264112622278	6.87827505953902
C	6.93060855765150	15.81838691481213	19.21989765802731
H	7.37660545110681	16.56348572351565	18.53693514894612
H	7.56767901782616	15.74694872085447	20.12269931745895
H	5.94341652694329	16.20259117940375	19.53056077263020
O	3.31304691184045	15.06892377175742	14.04212345387295
Cl	1.09796836294486	14.82619101724858	12.05868836700373
O	3.62434686827106	16.98313146190742	11.98274319512791
C	8.08056096090182	12.79632653390738	13.84699086519666
H	8.76733474602299	13.54193536989002	14.28713528110797
H	7.65420493007046	12.18015286773140	14.65559048932353
H	8.64027808267981	12.15763475979869	13.14756756415363

1_s

V	3.19761222524894	14.85617613980170	12.17146778913163
O	3.64249657951492	14.86055831446618	10.37304479033076
N	6.09163315565675	14.03818737621171	11.31358195979519
C	5.99898363458586	14.24776814055339	13.49599685203444
C	5.20826612776049	14.33844866450736	12.33015578992541
C	5.56537204532675	14.48387618697107	14.86107041663321
C	4.25163489439058	15.03143096126365	15.01204017044507
C	3.74051686379365	15.33365406240282	16.31494285168782
C	4.53046836331288	14.43444491733926	9.49412189404158
C	5.81051943895671	13.99144934469293	9.92098717650527
C	6.33618825432506	14.20173094156217	16.01617929023463
H	7.31444810309191	13.73366581596803	15.91038782870782
C	4.23145739893886	14.41659185750115	8.09647792190710
C	4.56906576700919	15.05079947585174	17.40649303398374
H	4.18868828781028	15.27313891652586	18.40833263098733
N	7.26506226781627	13.91275876581136	13.05243329925978
C	1.28027607933376	14.90537319010708	15.97230566920364
H	1.42116534878421	14.67114637944987	14.90593555229871
H	0.26395999223331	15.32127668184029	16.09808488429362
H	1.33702703320239	13.96233519075188	16.54537098491411
C	2.32927738560857	15.92199158361379	16.48836029154489
C	2.85122725200111	14.87448006187961	7.59444274679907
N	7.32700742663655	13.78337172504649	11.72942869600555
C	6.78144637864103	13.53495493059565	9.01797432145420
H	7.74458645896384	13.20147574756730	9.41673128419252

C	1.75772520295882	13.94675282476745	8.18081713625482
H	1.91744392121642	12.90263083251727	7.85642551264403
H	0.76293398729157	14.26791709823633	7.82169397897938
H	1.74531094262039	13.96968283238137	9.28168444391693
C	5.85974472700202	14.47860227036640	17.29857105151130
C	2.01530720425124	16.21310969877617	17.96887507035504
H	2.03505060250347	15.29805205999387	18.58853373353109
H	0.99984773140598	16.63979613268806	18.04578211985884
H	2.71706924654586	16.94709068667017	18.40569972566757
C	6.50766803965395	13.51190230136699	7.64485581642244
C	5.23288456290517	13.95811501399600	7.22631896206160
H	5.00521940216715	13.94115209054653	6.15843778948142
C	6.66800327485841	14.17970649728054	18.57236767848603
C	2.59056832478144	16.33971441127897	8.02301310365044
H	2.55663853703290	16.45262578067564	9.11894613371052
H	1.61844574251229	16.67708633854151	7.61914452950774
H	3.37490239391987	17.00861285752830	7.62425681714928
C	2.20842512662780	17.25470831849214	15.70935481725581
H	2.96722256305150	17.97999127362812	16.05563586109765
H	1.21060789006289	17.69703182528522	15.88407177996087
H	2.32580491554440	17.11359532471705	14.62271927760100
C	8.04531914874835	13.56865079420003	18.25629702025969
H	7.95478848622648	12.60517316350598	17.72211665045245
H	8.59354384039650	13.37439388114975	19.19513552686396
H	8.66612144261147	14.25044428816249	17.64640962451798
C	5.88165111546538	13.17905740965140	19.45158523541186
H	4.89512882231138	13.57977366316588	19.74265838111307
H	6.44048577335826	12.95479381559256	20.37884993765858
H	5.71199653204201	12.23007525120204	18.91230105932266
C	8.84402740961964	13.88750537724226	6.79180681459678
H	8.61560446095658	14.94174520644815	6.55392061109769
H	9.63065245485558	13.53980701106571	6.09696944508832
H	9.26059125189773	13.85637992072167	7.81390316011258
C	7.57933252812171	13.00667823815877	6.66242349767393
C	7.09907188065815	13.05925589238021	5.20055651566893
H	6.21033002896677	12.42400799275180	5.03614243525554
H	7.89834907671664	12.69104581793250	4.53306255956366
H	6.85111958425400	14.08877713387269	4.88572227052533
C	2.75593302397687	14.80740909545503	6.05804649804548
H	3.49771551697623	15.46344812734892	5.56701107966561
H	1.75363932914950	15.14658298988339	5.74234574401103
H	2.89145683904345	13.77930997663739	5.67573160680827
C	7.93422168316340	11.54122689601584	7.00681689992478
H	8.32548383255662	11.44680880238369	8.03499743905417
H	8.70716918329761	11.15734869307909	6.31567695630701
H	7.04494505503602	10.89118914014920	6.92470648292572
C	6.88816616561924	15.49451178207620	19.35676652299043
H	7.45084003690013	16.22618082620298	18.74974967989851
H	7.45998077095091	15.30161809193508	20.28313048947600
H	5.93149529049566	15.96366918880794	19.64516521068344
O	3.50596374434936	15.26386093780447	13.94729949057897
O	2.42454053373906	13.47682221455567	12.29591689782847
Cl	1.83015383092360	16.63314276245236	11.90562908562499
C	8.51287238541271	13.73283531613011	13.77896396317681
H	8.68876181890109	14.59390660224361	14.44303407695393
H	8.48974134572465	12.80500754658324	14.37458330837621

H	9.31576312971899	13.66784278903291	13.03082715806422

1_t

V	3.14788073191411	14.89857270963997	12.14980064139321
O	3.56630948861174	14.94035333069374	10.23282172839999
N	6.01226722243709	14.03984541917154	11.30677303685771
C	5.93025495334569	14.26885680174630	13.48653396191971
C	5.13530279770044	14.34348143074870	12.32390388020204
C	5.51065683743106	14.51039909208535	14.85490638300768
C	4.16594782825560	15.04205741200301	15.05334001290032
C	3.69590790259874	15.30415589170021	16.40497487161985
C	4.47003288669708	14.47734714798602	9.43663717622754
C	5.74528050491358	13.99375561240355	9.91354072182160
C	6.31205480545207	14.24544066869850	15.98624088730204
H	7.30221477842254	13.81158540590284	15.85750743459478
C	4.22666914350844	14.43936097842045	8.00828233014613
C	4.56086157959328	15.02951103671544	17.45884174408912
H	4.21528264992614	15.22671040072048	18.47810524275550
N	7.20201179422116	13.91318720829274	13.04188914646855
C	1.23341538592078	14.86241554690214	16.07856940040230
H	1.34998924106491	14.69740076579700	14.99677958593898
H	0.21795750169894	15.25939150782365	16.25908370310157
H	1.31403431152207	13.88764946787956	16.59315302557092
C	2.28264166877300	15.86185751532228	16.63107638374424
C	2.88049097720513	14.93003394961082	7.45482611429152
N	7.25872896423811	13.77132165741991	11.72260067261949
C	6.72268837550534	13.51283776393607	9.03738528362820
H	7.67064553220788	13.16565376027800	9.45937273506023
C	1.73403402875051	14.06602734336319	8.04121318750346
H	1.86219651593105	13.00491090675042	7.76031754898617
H	0.76668828705965	14.41173739803434	7.63354380300134
H	1.68812516425232	14.13394407281045	9.13896819303012
C	5.86755829507372	14.49529607509828	17.29054033356573
C	1.99048943719926	16.06721582363130	18.13043402030579
H	2.04257538691152	15.12130517839162	18.70034278421269
H	0.96734455765776	16.46521433808367	18.24674549569056
H	2.68150915163566	16.79515164810602	18.59402955493974
C	6.48967522069073	13.47899132323945	7.65203350337738
C	5.23802226285289	13.94752535525919	7.17946665519632
H	5.05084649266873	13.92144595564494	6.10415926596784
C	6.72915897765707	14.20842723809244	18.52737748272583
C	2.66715485338213	16.42078256149386	7.82479793268420
H	2.62084241434307	16.57622202668705	8.91422029217340
H	1.71575619626656	16.77394709531243	7.38665327502173
H	3.48143281410582	17.04420434901732	7.41231591132714
C	2.13621652985503	17.23540751723693	15.92727425286550
H	2.88105560497626	17.95314731407704	16.31716967867992
H	1.13100358720605	17.64619042851364	16.13372132010683
H	2.25493411678046	17.16051215863445	14.83473198641294
C	8.11102094111433	13.63694991531550	18.16099689489340
H	8.02920242324728	12.67501842360601	17.62279587997956
H	8.69354892921292	13.45066996678327	19.08036267633242
H	8.69431354896693	14.33968623767183	17.53822688062514
C	5.99852279186241	13.18120084983743	19.42597093549190
H	5.01333146666760	13.55293289984683	19.75629616604455
H	6.59818952496081	12.96812209205653	20.32979705132805

H	5.83617673016482	12.23091502439914	18.88700682150570
C	8.85653242749518	13.80574668555846	6.87733799608399
H	8.65447493431715	14.85902638800608	6.61342048891661
H	9.65922395993361	13.43213488107480	6.21533718643409
H	9.23747860558548	13.78497506706206	7.91329789098016
C	7.58242055584555	12.94187126218676	6.71582810495148
C	7.15507440996837	12.97656919800309	5.23716426581569
H	6.26173079341689	12.35377589630235	5.05219424192487
H	7.97110177962521	12.58252465150096	4.60590321174278
H	6.93756236404167	14.00418852353953	4.89532398725936
C	2.82267209162241	14.81161007163295	5.91956253413039
H	3.59630146243448	15.42709089824865	5.42487620950374
H	1.84026128674184	15.17152954977730	5.56702135041295
H	2.93375613863604	13.76673832915636	5.57625911692599
C	7.90153147439052	11.47653257004282	7.09852036605879
H	8.25746365144176	11.39275192998701	8.14016306100866
H	8.69109448783056	11.07181920136046	6.43901864277552
H	7.00595136867796	10.83842954223089	6.99575194024546
C	6.93885494038945	15.52539573867356	19.31364297266260
H	7.46052199642569	16.27630284289118	18.69392258976673
H	7.54830103862734	15.33903414421464	20.21681347894778
H	5.98096996403626	15.96570510558361	19.63994330392166
O	3.37946413415477	15.28764313381908	14.06671252153465
O	2.16832234429455	13.63988065738547	12.20638073697397
Cl	2.23615667099115	16.99614882143888	11.92966047506604
C	8.45063307433004	13.70531640144339	13.75870877982738
H	8.67444056819583	14.57851226654048	14.39239781392741
H	8.39956135875483	12.79736781001142	14.38314435322357
H	9.23985645817635	13.58434291143236	13.00304929193687

2-_d

V	3.68192619046694	16.11954370673028	12.05899268752137
O	3.79348578397051	15.48140776415086	10.20424206324646
N	6.40189517034208	14.94384581928129	11.24095329694807
N	6.25891995201833	15.18571109116724	13.43160399597809
C	5.51584807103927	15.11135821520467	12.27794358664712
C	5.69791841325095	15.10744081921519	14.74498310112199
C	4.32229553954805	15.45676107694190	14.95290682261760
C	3.81349811851796	15.35403150272957	16.30238131860152
C	4.68582148789766	14.90137939459429	9.45483855650405
C	6.01231383904640	14.59151323127542	9.91157593456239
C	6.48231129511535	14.57086039728702	15.78491116563249
H	7.47839693503403	14.19361847458307	15.54382920990208
C	4.35444544968194	14.52503458802930	8.10246523406443
C	4.65637672291073	14.87686509255090	17.31111797642992
H	4.25669897511202	14.80407459764478	18.32849383731444
C	7.62370270716902	15.24816836811135	13.11667876239846
C	1.38629725285685	14.85781166851231	15.77884855269471
H	1.51413643009887	14.99891921669668	14.69277066446776
H	0.33980293491747	15.10923953832979	16.03798060480922
H	1.55190599993953	13.79221802441244	16.02482701878310
C	2.35424305135911	15.75774080738895	16.58798281185176
C	2.95838512515036	14.86793022380840	7.54724386422178
C	7.71497761791417	15.09064514076964	11.70612513098305
C	6.90142329905699	13.84717028256080	9.11881873460005
H	7.84814549845351	13.51952456033673	9.55983904430176

C	8.76434318223297	15.55126960728840	13.88261512780284
H	8.68821815692458	15.74084211960456	14.95648656652292
C	1.86596223181641	14.15713327129461	8.38546608157312
H	2.02719869239684	13.06285698154737	8.38213655716370
H	0.86970450735054	14.35658616902309	7.94571418136219
H	1.85752310487162	14.51471579073992	9.42863935961234
C	5.98780549285431	14.45386670311556	17.08818595191096
C	8.94783144708151	15.23457167821997	11.04259697835192
H	9.01050253704760	15.18399063074890	9.95227542386415
C	9.99180896423357	15.66742309671823	13.21735201373938
H	10.89073761296128	15.91426692697817	13.79490195586776
C	10.08188877195369	15.51185055605099	11.81625910226161
H	11.04996339472240	15.64024522164202	11.31724509921474
C	2.00143849355458	15.60739964211715	18.08090982636504
H	2.08986607076427	14.56023995137669	18.42592647758466
H	0.95351620505128	15.92137101260972	18.23856668259845
H	2.63999421148553	16.24004818955657	18.72559509874792
C	6.57736660757056	13.46582902779774	7.80864084110916
C	5.30437684539662	13.84120427871758	7.32947070212624
H	5.03120367748504	13.56739320285882	6.30745545670077
C	6.81753091463398	13.87958452864108	18.24892697889794
C	2.73518102711973	16.39943879523426	7.59579106382788
H	2.79502614799761	16.77003330659039	8.63099370319089
H	1.73508000580002	16.64789796061901	7.19246194241733
H	3.49326664858528	16.92302472111967	6.98401572960176
C	2.13359108611462	17.23987159002653	16.19717524259648
H	2.80248808596925	17.90008990786510	16.77999096230239
H	1.08812802102033	17.53383102499879	16.40954761805499
H	2.32743798423532	17.39369089791212	15.12422681917529
C	8.23202249431418	13.46562074959346	17.80374102706519
H	8.20221077235458	12.68034445927565	17.02698731209496
H	8.79734749389897	13.06427907803886	18.66498487674293
H	8.80052358055779	14.32354380675105	17.40065511820422
C	6.10853799840562	12.63183248916601	18.82623680605637
H	5.09453692617926	12.87626596917805	19.18797155819270
H	6.68061424684931	12.20932496397227	19.67479081136127
H	6.00595726667061	11.85016166143458	18.05232590147284
C	8.92431041847913	13.41222361482130	6.88072226476359
H	8.77637426845629	14.39443511247173	6.39693719417712
H	9.66314739269433	12.83949012692390	6.28758547873433
H	9.36403258061576	13.59311528644891	7.87771385216142
C	7.58440540676236	12.64598253100905	6.98202780895918
C	7.07808942320708	12.37763902166519	5.55282558993855
H	6.14014202554639	11.79412427858200	5.55581523060640
H	7.83236942670689	11.79910207362745	4.98804454289107
H	6.89244930223967	13.31850333102708	5.00410057699330
C	2.79757193663225	14.41593700320427	6.08228266746992
H	3.53438530196800	14.89935160812639	5.41370907864383
H	1.78949406914011	14.69590036497205	5.72580716060668
H	2.89628705092511	13.31989111515034	5.97174987009395
C	7.82961407378269	11.28348202225244	7.67251557803547
H	8.22504393604560	11.41566473420015	8.69509614806817
H	8.55826867128734	10.67545813458235	7.10198025233245
H	6.88694351501693	10.71303008175022	7.75181423962100
C	6.95456435427904	14.94783402238130	19.35947447168006
H	7.46928686164226	15.84614288168395	18.97365896531092

H	7.53516262942305	14.55448913318069	20.21626985421287
H	5.96704255889600	15.26685690769709	19.73622896731037
O	3.52873529582143	15.84019864870166	13.99566049219593
Cl	1.36848494760113	15.83091725348745	11.91655657762335
O	4.19649078350144	17.63064114131736	11.96181820563100

2-_isomer_d

V	3.26615975192305	14.70186288065948	12.16180154754794
O	3.71979198530555	15.19991572834351	10.31543880209757
N	6.26189737942148	14.50825484843810	11.27118419329565
N	6.12038934948087	14.73517351111276	13.45454646597046
C	5.37595622346730	14.51505545295296	12.32114676115854
C	5.57045242564007	14.75863000274416	14.76970234813117
C	4.23141705384400	15.23117833840889	14.93496940329376
C	3.73519541813703	15.34796953441413	16.28274021385659
C	4.58582039671754	14.67500001160889	9.48773781412163
C	5.88508362662194	14.25441399046843	9.92021280250218
C	6.34150425875198	14.30824192097194	15.85527337037182
H	7.31673079534783	13.85797197883799	15.64995670594014
C	4.27294164619170	14.51279924672848	8.09435795768471
C	4.55908346923486	14.93532456484117	17.33795426160115
H	4.16902996026872	15.01702218764882	18.35844520997095
C	7.46384946234724	14.95673886436998	13.12539043196671
C	1.27888245758613	14.92778722162738	15.87640845442797
H	1.39870840033750	14.88593257900646	14.78200407380922
H	0.25328279464637	15.28227312377861	16.09406183812170
H	1.38506265135454	13.90658024979317	16.28623675352019
C	2.31163849472341	15.88645029444986	16.52017516664812
C	2.90369698514908	14.98348952935213	7.56753467147238
C	7.55509999889204	14.81011639309674	11.71586727648295
C	6.77556463808374	13.59868369395783	9.05912082323455
H	7.71065381517445	13.20403566540312	9.47278544601265
C	8.57538959544201	15.36517001886274	13.88317060725718
H	8.49214568523139	15.52780200280901	14.96148175271615
C	1.77560093627319	14.17061092526688	8.25057108565811
H	1.91142019882632	13.08800744255007	8.07302098338601
H	0.79453352884078	14.46833802155849	7.83371623458149
H	1.75232910491163	14.35258569387643	9.33693889245270
C	5.85320926297384	14.38968227502849	17.16551765823930
C	8.75862970735814	15.06947047583107	11.03712554604061
H	8.81439157756041	15.00843235534304	9.94644777444250
C	9.77836094312768	15.60056546189294	13.20339003473576
H	10.65841098210401	15.92472417308135	13.77192239546267
C	9.86866803867516	15.45523160610480	11.80111019136996
H	10.81770880008772	15.66841591894872	11.29447820806991
C	1.98846016927863	15.99830370053146	18.02283047927242
H	2.01487443901933	15.01540099092829	18.52925692332419
H	0.96849313659757	16.40591750513290	18.14513550193463
H	2.68593050216110	16.67773408321947	18.54802849388577
C	6.46396608371844	13.40804489539529	7.70310151004456
C	5.21807183098944	13.89736204202855	7.25623536170726
H	4.95933920913872	13.77191001276609	6.20192063430168
C	6.65454984024163	13.91048277833649	18.38802247638061
C	2.70364632968291	16.48896249870299	7.87168176518804
H	2.68389704045760	16.66687987339530	8.95900991719520
H	1.74114138980973	16.83144872104361	7.44613622884045

H	3.51331318715159	17.09109551010843	7.41842319605369
C	2.16198433333896	17.29474141909322	15.89304387838303
H	2.90889110716279	17.99292541821397	16.31521285921281
H	1.15420748794888	17.69563547138870	16.11294833770491
H	2.28507540835799	17.24935090752490	14.79892045126111
C	8.02991758147406	13.34230536378833	17.99350719339641
H	7.93314486345147	12.46988016439471	17.32259123801324
H	8.57620465003974	13.01281308753871	18.89643465150695
H	8.65336909972264	14.09875863190118	17.48279970449045
C	5.86607239079851	12.80057897997846	19.12217141134320
H	4.87768394184941	13.16162797466694	19.45594039098065
H	6.41926720449148	12.44803028350158	20.01405992720243
H	5.69599936825262	11.93745734023337	18.45407969873326
C	8.82829110086845	13.38756594792939	6.82016179073133
H	8.72917113893260	14.43194812661900	6.47321590804003
H	9.55880048930903	12.87205824802093	6.16734037232868
H	9.25127787663010	13.41200806089385	7.84027798619286
C	7.45847378061014	12.66971995788459	6.78919404684179
C	6.97767282903252	12.62109087919964	5.32727967862904
H	6.01848714430877	12.08187547194321	5.23126456010738
H	7.72298974231753	12.09439281462453	4.70307240370076
H	6.84199721090719	13.63500844527534	4.90976200131728
C	2.77977365006975	14.78572204186565	6.04414359191780
H	3.55297810575940	15.34822411214370	5.48775577568712
H	1.79268084584933	15.15371337559365	5.70983177358877
H	2.85134681939356	13.72062407427316	5.75492934318635
C	7.63346007063152	11.21674186713190	7.29056867869751
H	8.00705056731544	11.19141196954826	8.32938637990758
H	8.35177260825536	10.66226096428308	6.65602241699211
H	6.66755836873112	10.68120695671009	7.27271622957963
C	6.88130947147618	15.09879721908884	19.35211653200975
H	7.45387129427898	15.90128709743017	18.85295605685492
H	7.44346751671849	14.77666693266340	20.24989397316903
H	5.92408023865762	15.53388360735750	19.68862051839181
O	3.48097772270247	15.56110447107025	13.91655465705058
O	2.79366522673760	13.18028934599073	12.28296235849081
Cl	1.37674689331091	16.07589868247719	11.90001698257695

2_isomer_s

V	3.33201607774665	15.07789062662602	12.12603266584641
O	3.83978787927865	15.31482494462265	10.37302344276842
N	6.32442304009536	14.63745989813411	11.26085784889110
N	6.18406263995647	14.87116778183428	13.44671831013881
C	5.43857169695739	14.72022983323767	12.30490996974071
C	5.64055678431159	14.86607525248180	14.76334820900743
C	4.30495131990337	15.30392927675755	14.92894123689778
C	3.73708589279113	15.36052796235318	16.23652407236647
C	4.65900161591506	14.74939128095687	9.48946539036111
C	5.95183631113961	14.35268290766936	9.91623567219619
C	6.39048819853931	14.39376507235794	15.85423668421625
H	7.37556056127581	13.95922291605396	15.67214202169155
C	4.26734926175920	14.53548407868801	8.13853844911372
C	4.54456978753810	14.93389447271054	17.30146657906328
H	4.12170780037459	14.96927784357233	18.31000992040295
C	7.54548423229655	14.99407972466722	13.12409866038403
C	1.32517762645569	14.81506798884486	15.77793304913116

H	1.49881626109375	14.72789048269476	14.69419342549854
H	0.27827891117165	15.13693554341943	15.92676942927312
H	1.44395754129843	13.81415671937091	16.23015189957490
C	2.28670972673733	15.83283642637494	16.44089898055717
C	2.86946760973608	14.95735649988504	7.65151006506148
C	7.63535046637219	14.84333716774006	11.71864473390868
C	6.81612762943571	13.66351697289278	9.05539655806565
H	7.75999337483816	13.27326537744825	9.44765542505551
C	8.67551695720090	15.32727025475701	13.89199988162298
H	8.60241732291955	15.50338715292327	14.96819575155554
C	1.80148001591515	14.09984663803655	8.37537735443282
H	1.94978312296622	13.02677678122837	8.15875890524363
H	0.79229992121831	14.38433899554085	8.02548108581890
H	1.82987207461747	14.23946103572924	9.46713569465639
C	5.85537402930498	14.42795091310963	17.14818669386143
C	8.85617834836893	15.02144113299316	11.04401012443285
H	8.91929387160089	14.96603944894623	9.95406591658081
C	9.89335761968578	15.47839836469164	13.21853570793818
H	10.78929574713995	15.74440261011380	13.79097785583008
C	9.98239806390381	15.32805259347870	11.81669522127225
H	10.94622210208701	15.47949734473869	11.31742347617679
C	1.92758383157167	15.92329113787755	17.93666379964897
H	1.98338912597732	14.94141742592821	18.44063916081586
H	0.88939040504596	16.28504178949012	18.03889569156868
H	2.58205857487099	16.63149845585663	18.47695732191372
C	6.45373264052102	13.42925662648986	7.71852897092587
C	5.19290022058255	13.89408050168440	7.29403838075326
H	4.90025339152392	13.72596000579587	6.25567693885429
C	6.63078913740505	13.92676661333483	18.37840031927679
C	2.62640199363356	16.45829501578234	7.94196608989171
H	2.62592938519400	16.67286968682084	9.02285619534433
H	1.64290508319770	16.75962376780812	7.53763528067192
H	3.39963087248884	17.08209206823467	7.45775966371112
C	2.08263984656466	17.23540809267648	15.81829643044916
H	2.78482478821366	17.96645351226567	16.25868694904439
H	1.05427661518600	17.58513394331807	16.02235266455423
H	2.22369205364199	17.22465479735697	14.72545038103577
C	8.03675971225951	13.41979388001655	18.00965737693842
H	7.99645995065051	12.56668723259841	17.30859772730414
H	8.56118308195371	13.07582831753412	18.91870813763604
H	8.65406418767024	14.21548014839310	17.55376764651361
C	5.84959855686586	12.76470498874398	19.03554768201793
H	4.84192687482823	13.08095994127403	19.35657902121157
H	6.38634565095196	12.39298093916375	19.92774652571498
H	5.72837239395962	11.92350289103481	18.33009698428447
C	8.78265068651088	13.39519576600541	6.75812771018620
H	8.66686613504760	14.42870457831291	6.38560640405737
H	9.48769106717775	12.86563996728825	6.09138596359077
H	9.24872630066713	13.44554704515019	7.75840470075814
C	7.41948049307439	12.66542655900367	6.79508742526945
C	6.88641713110560	12.56569199993096	5.35418338956202
H	5.93128155476571	12.01307181245746	5.30650510019614
H	7.61418569343927	12.02393256699770	4.72429032112580
H	6.73161438409867	13.56266713686908	4.90407805951656
C	2.70832384361962	14.73776652103768	6.13471459834685
H	3.44623258873030	15.31846146041214	5.55161016642032

H	1.70231839381197	15.07158949408788	5.82532750377935
H	2.80122508366513	13.67307924861483	5.85398101918719
C	7.61677347149062	11.23287793973793	7.34432562404293
H	8.03449853961017	11.24080770555333	8.36659161444119
H	8.31215041730158	10.66292794588387	6.70080965933513
H	6.65563384120823	10.68998674363681	7.37933861916355
C	6.78086022202683	15.08628188758932	19.39093352904784
H	7.33808213322923	15.92950438463008	18.94493993299155
H	7.32930437671314	14.74653665199910	20.28873724435710
H	5.80020430620830	15.47003275584719	19.72181936813606
O	3.60357030612008	15.65233116265363	13.85416861105400
O	2.83322844081399	13.58101329227818	12.23571424885407
Cl	1.58284387885845	16.47909978486135	11.87274788186404

2_s

V	3.63749098273354	15.96848909623233	12.06901373552894
O	3.91010940885910	15.44621183927198	10.32724495637410
N	6.44740404006923	14.91480395550266	11.24880242890091
N	6.30737423347707	15.14799179221767	13.43971299858279
C	5.56333221484108	15.08863876440360	12.28600742030346
C	5.75837407337832	15.06559926795217	14.75204347632836
C	4.39523970150841	15.40940595518272	14.93793714650495
C	3.82504860948795	15.34606075847126	16.24860694490242
C	4.75000984380719	14.85953592690749	9.48970540124700
C	6.06800943630857	14.55459941390570	9.92377957542817
C	6.52543013148758	14.56081600580183	15.81918336346118
H	7.53012708475004	14.18570900298600	15.62011081336277
C	4.35593692548369	14.53000463733597	8.15849368979058
C	4.64960460478920	14.89431145934409	17.28695882584201
H	4.22447117836857	14.84330276761687	18.29376005622266
C	7.67567709995560	15.18909947951644	13.12378353700114
C	1.42582189076230	14.82223971077349	15.67184726846772
H	1.58750874692519	14.91565459394419	14.58563602169687
H	0.37110513615790	15.08343893057249	15.87545395342232
H	1.57749435516498	13.76667932378777	15.96208275790408
C	2.35981233008638	15.75247087992646	16.48329586466359
C	2.94877395088646	14.88770011927461	7.64911986331810
C	7.76504740194292	15.03860115146226	11.71752457418525
C	6.94487624168441	13.83454748093476	9.09780335730050
H	7.90541490073600	13.50275848892469	9.50024663019608
C	8.81908680753120	15.46481495367748	13.89630641661823
H	8.75155470408715	15.65584496291211	14.96982761621972
C	1.88590947918210	14.14856952300273	8.49761802579182
H	2.03804953052231	13.05502890510212	8.44777927243828
H	0.87592046176407	14.37022753083976	8.10648519009110
H	1.90816635908808	14.45873041648221	9.55500086538495
C	5.98689029055776	14.47274140125019	17.10713229292843
C	8.99821919123779	15.15975015048743	11.05093856115651
H	9.06497314716844	15.11967274789236	9.96098394826251
C	10.04627712730760	15.55679386327845	13.23032542281704
H	10.94969105883051	15.78360628786157	13.80782364189907
C	10.13471871364249	15.40673840209750	11.82889967392215
H	11.10566651258082	15.51900105036089	11.33315389333338
C	1.97051388449118	15.63545172846012	17.96964779741849
H	2.05329016895827	14.59795732089141	18.34151624144940
H	0.91829157751880	15.94663267235704	18.09298936590659

H	2.58666577466634	16.28865923648760	18.61412367626694
C	6.57671725231221	13.48178682218250	7.79081626107141
C	5.29352277723461	13.86425815451838	7.35061636489185
H	4.99694988742111	13.61259090704223	6.33052920660822
C	6.78591154316567	13.92900834285995	18.30343199951822
C	2.73197689466526	16.41812550192499	7.74069306978521
H	2.81098847156753	16.78133160109250	8.77719969120277
H	1.72520519670945	16.67611226252932	7.36468027580695
H	3.47495639432070	16.95555803369767	7.12412249929002
C	2.14877868451621	17.22465035583050	16.05224027604085
H	2.80007696403972	17.90020502934354	16.63567796039459
H	1.09945958523040	17.52015551441966	16.23415069268198
H	2.36257414001933	17.37194421159229	14.98222605603515
C	8.21539254772761	13.51774839007516	17.90726298776942
H	8.21785413204722	12.71342487736329	17.14953073469147
H	8.75505510748583	13.13865323690138	18.79314264470950
H	8.79347934612708	14.37200368873199	17.50967453518980
C	6.06336172882642	12.68884278158007	18.87975372899938
H	5.04064201535879	12.93247966634922	19.21612198439638
H	6.61679430720213	12.28677940577503	19.74829986093963
H	5.98567284340903	11.89046109301441	18.12035229523580
C	8.89057435659980	13.46342391007115	6.79315577056838
H	8.72586058466380	14.45688655468246	6.33919374869314
H	9.60743388224802	12.90936092846015	6.15966603943277
H	9.36881905264249	13.61609179431508	7.77722092619895
C	7.56025430846344	12.68415908823660	6.91574509326129
C	7.00941779783114	12.44343086275021	5.49840698852319
H	6.07680330085247	11.85185955872023	5.51508744831478
H	7.74867580174276	11.88018843162028	4.90159897114431
H	6.80713975551580	13.39247466802489	4.97033201483791
C	2.75393416412789	14.47120826257622	6.17823470114400
H	3.47149202001905	14.97374554454401	5.50434715213873
H	1.73833197879037	14.75814611617004	5.85347190568148
H	2.84864360091631	13.37904297642217	6.03819819611155
C	7.82749563820628	11.31134775909515	7.57652579543284
H	8.26229015308138	11.42120221778604	8.58579331359822
H	8.53566270823568	10.71898293859276	6.96811416748036
H	6.89116280083411	10.73396448261750	7.67552064748688
C	6.87792979729373	15.02510174626609	19.39104575393403
H	7.39306961783117	15.92227921739427	19.00370484289386
H	7.44163608231549	14.65425053305732	20.26687567481305
H	5.87924377132296	15.33760626221139	19.74239804262415
O	3.66769107319848	15.78369638172989	13.89905179562639
Cl	1.42196824459045	15.57730535662772	11.96025570571083
O	3.97431937650406	17.51439657551386	11.94683761624974

2_isomer_t

V	3.23782586235472	15.03511162570453	12.12481031070296
O	3.66884483096868	15.23397321944831	10.20727349282116
N	6.20473765720031	14.61075442431611	11.25605352907044
N	6.06509686895158	14.84623095019217	13.43997380839254
C	5.31406138007764	14.66468561388319	12.30183030641350
C	5.54475765570954	14.83116358356253	14.75777883838610
C	4.18951125164393	15.27603918483765	14.98291997567461
C	3.71066284580535	15.34808240468614	16.35121784339551
C	4.55219955194237	14.71269513681396	9.42602691629303

C	5.85464822618611	14.31752897091206	9.91448347639660
C	6.33605702764862	14.35906950148213	15.81582079485441
H	7.31051199927073	13.91997072155900	15.59085930089837
C	4.25819100349347	14.50603842789194	8.02242682498633
C	4.56487438055859	14.91984325389586	17.36393000038824
H	4.20486644236023	14.96180175533998	18.39628633561708
C	7.42588919376461	15.01675649955351	13.11146958483389
C	1.26403041563043	14.87932038159809	15.98420216516969
H	1.38675609798676	14.82178476275324	14.89181539106233
H	0.23783263787380	15.23008197551209	16.19715129640007
H	1.37103991910005	13.86229590877930	16.40291682402339
C	2.28605176277466	15.84958309114465	16.63133755315046
C	2.89818241080074	14.94458444816591	7.45987208104796
C	7.51531179677826	14.86503688039636	11.70838365963815
C	6.75463390941807	13.63592071453340	9.08965430605049
H	7.68096518906176	13.24698287667376	9.52352933937389
C	8.54183014777706	15.40528761700654	13.87136883351697
H	8.46566892895880	15.58398585871948	14.94707409111092
C	1.77181886742707	14.12950147041623	8.14719787321057
H	1.90153276261391	13.04817360443765	7.96027552130411
H	0.79327204634975	14.43290281183082	7.73265070446273
H	1.74993741237234	14.29564808931373	9.23511198036048
C	5.86619631954493	14.39388748394633	17.13577578828889
C	8.72160095913535	15.09719876930234	11.02700121441092
H	8.78115943675435	15.04226890957559	9.93668933733671
C	9.75043365417520	15.60858324975781	13.19204740206151
H	10.63575787813370	15.91682792062714	13.75976791994005
C	9.83901941454306	15.45716191336821	11.79205181081095
H	10.79206991088542	15.64965182908956	11.28630005946909
C	1.99620183634271	15.90607153736041	18.14356160358619
H	2.05419510842562	14.90968751516598	18.61882266773593
H	0.97104521200453	16.28449062180428	18.30054811501919
H	2.68604592010071	16.58836236789934	18.67366096179956
C	6.46196432958817	13.40132624125744	7.73420116291068
C	5.22286578015988	13.87378483980164	7.23447532168087
H	4.99596393770392	13.71012333215034	6.17903117648784
C	6.69415235283729	13.87964964814407	18.32054095599909
C	2.68127905707537	16.45951655321606	7.70807134145774
H	2.63958351197729	16.69765158886801	8.78264577584462
H	1.72452570754601	16.77123053653996	7.25093791290371
H	3.48864534886659	17.05199338294734	7.24007877402660
C	2.09969858455520	17.27677699905395	16.05564369064627
H	2.83550407836022	17.97483286705492	16.49526609229089
H	1.08958021573460	17.64492694025352	16.31176428903945
H	2.20144618504030	17.29438547339058	14.95904255732850
C	8.06950284527986	13.34441355153941	17.88298546730887
H	7.97722336044153	12.49194900153356	17.18626290121858
H	8.63033474678025	12.99033990900757	18.76582872749236
H	8.68054938499324	14.12671215255681	17.39674460418649
C	5.92261561277884	12.73437825342523	19.02009123981336
H	4.93936182083822	13.06985740616108	19.39245162755289
H	6.49930425342105	12.35671696775973	19.88419259221893
H	5.75045534693531	11.89323039558911	18.32546707829697
C	8.84108062375168	13.34835783684416	6.91507673430394
H	8.75927517683243	14.38338281996506	6.53792006682914
H	9.57614605614477	12.81118192657579	6.28835357300076

H	9.25031860922448	13.39022759605361	7.94012687934167
C	7.47013935040983	12.63192451887831	6.86770054522439
C	7.02366526838869	12.53812471594832	5.39723318487407
H	6.06900802821550	11.99324692582680	5.29063975096045
H	7.78380694837417	11.99038096651511	4.81267045977085
H	6.90539235856944	13.53638449540129	4.93921248334619
C	2.80745786670696	14.69447866728614	5.94213614599365
H	3.58019353560713	15.25236391991368	5.38183374653387
H	1.82289936746297	15.03701762634550	5.57867363638252
H	2.89599666573635	13.62236351461176	5.68796418510222
C	7.61897042521671	11.19679598803932	7.42890263034227
H	7.97642601115099	11.20018987069273	8.47360464987995
H	8.34499255987382	10.62186801334190	6.82519311322978
H	6.65225180468242	10.66353315369718	7.40606282592538
C	6.91588127755885	15.03959583204078	19.32138299874554
H	7.46637870181391	15.87115700172715	18.84635562534577
H	7.50334008299748	14.68799323325954	20.18919200769488
H	5.96169973459815	15.44212141911490	19.70283674656891
O	3.41323204769295	15.60323694878929	14.00717712408149
O	2.45187036783515	13.65164044844422	12.20317206399199
Cl	1.98378764533644	16.95469514517991	11.85440609835962

2_t

V	3.57292186627723	15.90804461555780	12.07145439687799
O	3.75540775558408	15.39258570462326	10.16811537344813
N	6.35062122669788	14.88425453825057	11.24493486334130
N	6.21041816556436	15.11595713121066	13.43569131275798
C	5.46442704076232	15.03581287763316	12.28394003085023
C	5.67323036961128	15.04055062778813	14.74661033340948
C	4.29622313755417	15.41845926174303	14.98953191615752
C	3.81163876185632	15.38162633254249	16.36019054571205
C	4.65775501663743	14.85277205103175	9.42481590128141
C	5.98323712532858	14.53324573177413	9.92007509861686
C	6.46790377287749	14.52258908610192	15.78364387843165
H	7.45417577718735	14.12282267756829	15.54215107392436
C	4.35628453188606	14.53668810980034	8.04017867205530
C	4.67191481470113	14.91628430346930	17.34957998839631
H	4.30494598204038	14.87974375700617	18.37955445956167
C	7.58337474092552	15.19088770693496	13.11627455817408
C	1.37125531746369	14.88348773580611	15.94917440851977
H	1.46983950843869	14.93199232324999	14.85288617468432
H	0.33849155678455	15.17765665881363	16.21182418683196
H	1.51744020987061	13.83673396739192	16.27289479846583
C	2.37208935570543	15.82171057978299	16.67102148201966
C	2.98147674016072	14.90102912354322	7.45819956535228
C	7.67283009994493	15.04187998047381	11.71104741959386
C	6.88524543565939	13.81381804586919	9.12602574856243
H	7.82392015666457	13.47102817730326	9.56985389767731
C	8.71643260539859	15.51215527121426	13.88293229910989
H	8.64486447121669	15.70005026439394	14.95704432377107
C	1.87284615817503	14.12370914491328	8.21344809610862
H	2.03383015436815	13.03371786502060	8.12493475918007
H	0.89024305960321	14.35816266211736	7.76437580512218
H	1.83026924826834	14.38952782331554	9.28189421209494
C	5.99109240057806	14.45064329684783	17.09743318550121
C	8.89594697069192	15.21068178081864	11.04022135962519

H	8.95982246717437	15.17036538069795	9.94982935460550
C	9.93932860750156	15.64945286458027	13.21299812097804
H	10.83554577118234	15.90870454912023	13.78797458721812
C	10.02781967658098	15.50132558500543	11.81317488100388
H	10.99181789517944	15.64725873381539	11.31272490912251
C	2.07653022788630	15.75699841224867	18.18221767623244
H	2.15532641618325	14.72863984397690	18.58001784612566
H	1.04219621619069	16.09889446298990	18.36132591125242
H	2.74780529654450	16.41226697183767	18.76733952886411
C	6.58246487623241	13.47217003659733	7.79803451105150
C	5.32536862710487	13.87574782499375	7.28415400092185
H	5.08980834505523	13.63396871757783	6.24592850093848
C	6.82777504612783	13.88007236635359	18.24997737213497
C	2.74173637646087	16.42713761422723	7.58482927321077
H	2.73728163006082	16.75175190295951	8.63627453618274
H	1.76332750412355	16.68371141793376	7.13974753112532
H	3.52103661916410	16.99281845956915	7.04237518407037
C	2.15679693560812	17.28579861913049	16.21049901260438
H	2.85824249485294	17.96637182782665	16.72660557821618
H	1.12838894665015	17.60296658816507	16.46150488510823
H	2.29465873739263	17.39440186321297	15.12412668385575
C	8.22519127383005	13.42762288612120	17.78953743687494
H	8.17029244363304	12.62593078067222	17.03121098830871
H	8.79120950303548	13.02970029741845	18.65015147809428
H	8.81118127188504	14.26516383156003	17.36889860241515
C	6.09219871771676	12.66015926426906	18.85532567751272
H	5.09343901026127	12.93257146604601	19.23784553217809
H	6.67312456823054	12.24122204790485	19.69731081012976
H	5.95817762998736	11.86621975360882	18.09935054944781
C	8.94502509694996	13.42694265998586	6.93202220031227
H	8.82019156637086	14.42398995648410	6.47341564484577
H	9.68569032720800	12.86369013779998	6.33543746896708
H	9.37210401091384	13.56733235869270	7.94092745525493
C	7.59866977828376	12.66506814625686	6.97573260948763
C	7.12539872542901	12.43440556796109	5.52897410942044
H	6.18547136419562	11.85580694514358	5.49094963263839
H	7.88969033507256	11.86121443933595	4.97500843203235
H	6.96767764747390	13.38636529026596	4.99126251627978
C	2.88426855280752	14.53340592381277	5.96460149556149
H	3.64211450849920	15.05898670805471	5.35494279188639
H	1.89080432914506	14.83006454724491	5.58528048853849
H	2.98904296310013	13.44639169392193	5.79349709869237
C	7.80917845590554	11.28714085793975	7.64892363979844
H	8.19073786627011	11.38861454903389	8.68012824848083
H	8.54079391122564	10.68778596232565	7.07649424025305
H	6.86124476286200	10.72241850995342	7.69465839550257
C	6.99981654213572	14.97019394609702	19.33545262170150
H	7.52398890782875	15.85316926970116	18.92838781302735
H	7.59216582502942	14.57666259972408	20.18171269003926
H	6.02882440406457	15.31000696935431	19.73508125068079
O	3.49851105616995	15.77512978926202	14.04360049567043
Cl	1.41535582671618	15.13297814181626	12.00208155444907
O	3.88134557002204	17.46767544550006	11.93819402147767

9_q

V	7.52693884165833	5.30478316760423	4.83393130157467

Cl	6.80892915313863	7.06664419746982	6.31374777027479
O	8.41098599709191	6.73445148501441	3.51747475486025
O	6.70064732005897	3.91827973384275	6.17019028628970
O	9.34237352197695	5.10552558238367	5.66466303738903
N	9.34832662379617	3.21813420069351	3.50507775403560
O	5.87750684554752	5.37489648085421	3.71025511559335
N	9.46343860249709	2.32573703002869	2.45680924745777
N	8.23256505096400	2.32834527296787	1.91542876971426
C	5.95718025290900	3.41418949212359	2.28883794919320
C	11.63906605468324	2.69061555547839	4.08761481872843
H	11.63032885269996	1.99297732909270	3.24749037674596
C	7.34536861152994	3.18328676820189	2.59657293122226
C	10.46400630457422	3.43001860074752	4.32597072867114
C	8.09974773456147	3.79526521133200	3.61665209341860
C	5.29740300256925	4.60695214357997	2.85313693745798
C	3.95716116826047	4.93889040326189	2.40701847154837
C	12.76234283453130	2.83814476078107	4.90599807366866
C	11.54948843982716	4.50786312923443	6.29103152896327
C	5.19132882810865	2.54886843048907	1.47258434133370
H	5.63520907776831	1.60491786714263	1.14354581066274
C	10.38796266169854	4.38644892248060	5.42929270415179
C	12.67936779826740	3.74653697828771	6.00123095792808
H	13.55465908525549	3.84534724983952	6.65021650749931
C	3.30051875208755	6.24729133600797	2.88367500583779
C	3.86987457790299	2.82741397104606	1.10727364370189
C	3.09210990281374	1.82246625013560	0.24054084024008
C	14.06131281134952	2.05442861786176	4.67510301590160
C	11.49135138456166	5.45683629488551	7.50177176455011
C	1.90157244951924	6.43594302482852	2.26560426681837
H	1.93458384709972	6.46314632889753	1.16101352460451
H	1.47870658788414	7.39640327020972	2.60943568837715
H	1.20062610842998	5.63822245611637	2.57249421443239
C	15.22210144399694	3.05276932233578	4.44789745374912
H	15.03162537696398	3.67944628167974	3.55846805019386
H	16.17207546522616	2.50909649519620	4.29058697890359
H	15.35856500757185	3.72696720311169	5.31123865055528
C	3.29443868277109	4.04626056080532	1.56574712763989
H	2.27564757365369	4.28588265197911	1.25457629009087
C	5.50126699086819	4.13524119271272	6.97460758935769
H	4.66575034475400	4.34769883878216	6.28621289049797
H	5.68549501595124	5.03624833982333	7.58608025713773
C	14.36337085743529	1.19013055187159	5.92313411292159
H	14.47593837981029	1.80646472326152	6.83196768561093
H	15.30244305115011	0.62378323507845	5.78104321547074
H	13.54849144567626	0.46702424611775	6.10543661172806
C	11.23961059362093	6.91193464196806	7.03238508332523
H	12.02049833566057	7.23268269716383	6.31852185316730
H	11.27042259778478	7.59534842778082	7.90089458035595
H	10.25260432377391	7.00762575522070	6.55471377112333
C	8.08141189602100	1.61738418258784	0.66229512641775
H	9.08791464950610	1.47217532489069	0.24038416155843
H	7.46958551439831	2.21170328877978	-0.03610386470569
H	7.60755401943497	0.62764389340020	0.80250215418794
C	3.84983560616871	1.59434296513363	-1.08931524273945
H	3.95454384371414	2.54064393852824	-1.64950459731197
H	3.30436769330104	0.87451715547000	-1.72729534362639

H	4.86296570633357	1.18918711212143	-0.91936205232420
C	13.97170021233947	1.12630734023791	3.45061108948581
H	13.17529384915509	0.36985653590676	3.56787547757791
H	14.92721958445208	0.58800019887612	3.31811627009372
H	13.77451936669682	1.69240050117960	2.52277854945144
C	3.14437070823642	6.25020265125560	4.42605753974403
H	2.58408142003965	5.35827838664308	4.76286774589251
H	2.57465209698711	7.14438874319630	4.73991384725402
H	4.12304188633038	6.27754523744054	4.93108165787945
C	8.30103687547349	6.65540575547872	2.07375989432694
H	7.23908327640928	6.47826291713171	1.82495650624021
H	8.89402919937814	5.79142466870144	1.72304731565063
C	4.17523233127891	7.45371959093861	2.45550476716660
H	5.16526825857753	7.40940905274118	2.93602376252049
H	3.68676189615339	8.39734692577654	2.76036140977442
H	4.30356771634034	7.47493330952078	1.35750794698848
C	10.35000950586680	5.01805378159171	8.45553888214967
H	9.36759588176217	5.10571974062943	7.96489993579677
H	10.34384116078553	5.66315884479032	9.35335456570621
H	10.49873361138881	3.97381117547409	8.78770939659667
C	6.77030503689572	2.31444174374258	7.87942781587779
H	7.33799494881319	2.85997091563554	8.65573784250515
H	6.82540726379572	1.23569485102975	8.10356661514754
C	7.32181068472046	2.65501425513124	6.49932393734219
H	8.41432068352432	2.79597588156943	6.46920666139617
H	7.03639487953686	1.90605399365113	5.73281017419103
C	12.80873075085879	5.43712619093561	8.30032156062467
H	13.03816859280568	4.43219919851724	8.69962556015450
H	12.72314294151795	6.12410915757986	9.16076972441677
H	13.66787614210011	5.77388453130592	7.69176094093283
C	2.97694950030953	0.47989448773340	1.00173955217040
H	3.96963259827916	0.05533327746142	1.23313247964211
H	2.42436016031766	-0.26246070490615	0.39632613562561
H	2.43900882518160	0.61530532353957	1.95684476628206
C	5.33181238945484	2.84790018664401	7.78390695488801
H	4.87175101238647	3.03748773857776	8.76836217824923
H	4.69209323454707	2.12540449915788	7.24370615422425
C	8.91639056191979	8.03475008751911	3.92305420301075
H	10.01051562136530	7.95377396333712	4.08358651684219
H	8.41528952963297	8.28047931733668	4.87497887547832
C	8.80792448398198	8.00465047162841	1.55038675746861
H	8.27631470040158	8.31942071577200	0.63632578987760
H	9.88670753631246	7.95034328718954	1.31436143759525
C	1.67087965667979	2.31234239311021	-0.09354233234997
H	1.68578936739161	3.26159632212134	-0.65834073218024
H	1.06388989294299	2.46270679859294	0.81686912639988
H	1.15456336711230	1.56117410323188	-0.71754311892345
C	8.57340192723485	8.94030228344612	2.74788141734307
H	9.19317576789044	9.85246367420922	2.71685806166973
H	7.51120860326595	9.24265312896606	2.80589660766859

9_s_UKS

V	7.53599768793587	5.26632023871845	4.82510520212553
Cl	6.86433081161813	7.05205616254341	6.23386060541083
O	8.36747437175672	6.65119845252561	3.44979204455561
O	6.73657974847685	3.90344781137958	6.22457677809548

O	9.22705904946877	5.00630791988779	5.57357743931808
N	9.33198683841866	3.11526473403186	3.52056414453774
O	5.98780067720816	5.20023204874094	3.77881135651244
N	9.41115263626873	2.16314381584830	2.57105229229962
N	8.18563367137431	2.15263574144456	2.03070526942633
C	5.93807298758266	3.33384074566141	2.27802789835465
C	11.66493293928973	2.68107671398239	4.09675881716343
H	11.68385060353098	1.96750812881585	3.26965980865928
C	7.32563586657488	3.06149313041637	2.62266516407807
C	10.46422692631326	3.38086729205290	4.32725859063476
C	8.08861318958582	3.72468084961399	3.61398659807705
C	5.33113285183572	4.49529546915900	2.88116170256284
C	4.01491192003344	4.89762133185921	2.48644824793603
C	12.78043573175501	2.89764295509864	4.90926634190057
C	11.47484864818566	4.54120561875219	6.24392510450309
C	5.16293580527010	2.54615333869138	1.39623649600473
H	5.58479435520263	1.62486737451131	0.98845548637646
C	10.34883395725038	4.33895845553227	5.37883886308081
C	12.64678996608981	3.82373753120338	5.97396595873905
H	13.51077006455856	3.98410135377850	6.62625722803953
C	3.39792003249625	6.19997061762268	3.03532626953616
C	3.85176703741726	2.88706978448677	1.04123816038628
C	3.05509652728913	1.97180087873351	0.09247798068441
C	14.11838741996494	2.16990090972856	4.69641810968681
C	11.36165919921672	5.50538356081412	7.44042530739665
C	1.99734308564260	6.45743392202198	2.44615428527572
H	2.01903068697296	6.54693830454701	1.34474812924362
H	1.60243163269109	7.40683288326773	2.84918638648856
H	1.28074787878299	5.66017513612165	2.71531765794279
C	15.23074480232698	3.21249179674355	4.43560887888210
H	15.00783141661249	3.80525745376439	3.53042984939990
H	16.20632101615605	2.71236461658206	4.28960117970127
H	15.33600164089175	3.91655886690161	5.27953825206573
C	3.32049542664877	4.07741941636654	1.58392519315266
H	2.31100364719207	4.37555019881895	1.29286107792341
C	5.58316044116890	4.14182110782793	7.08267074250373
H	4.72542602995562	4.39073242653432	6.43591604613209
H	5.81434880834240	5.02070611195019	7.71041844302458
C	14.46604155352376	1.35374411819259	5.96354190393035
H	14.55033200250134	1.99958494797543	6.85497942170333
H	15.43095206216590	0.82870102267720	5.83534816480077
H	13.68664530259308	0.59826926637113	6.16904706818226
C	11.03669574153978	6.93735088970072	6.94987968572204
H	11.79853992346805	7.28544832792298	6.22838807552697
H	11.03363255036886	7.63637730592744	7.80654108768026
H	10.04495435185259	6.98051754509499	6.47415592181313
C	7.99199984471915	1.24467623833087	0.91445714444978
H	8.98384074505224	0.85981463382127	0.63618230313594
H	7.54785287409109	1.78030205095052	0.05943332001162
H	7.33931646838529	0.39912656957434	1.19357006036746
C	3.81366702540816	1.82671806900591	-1.24755663384985
H	3.94545317477279	2.81036541283666	-1.73271014240072
H	3.25727612109750	1.17053661311872	-1.94250617623732
H	4.81643301967783	1.38608833570718	-1.10418997663043
C	14.06685150868238	1.20548383125641	3.49790449351722
H	13.30428552044897	0.41860946465376	3.63914619625105

H	15.04451200976491	0.70560823659744	3.37564614597462
H	13.84234424012811	1.73698212238308	2.55572040160848
C	3.26020941358844	6.13045633417314	4.57570896994633
H	2.68516568326818	5.23553414203216	4.87721644663334
H	2.71972003112844	7.02156692853897	4.94501124137638
H	4.24613769845625	6.11213622051586	5.06616181464191
C	8.19858823871114	6.53203609515952	2.01278323961116
H	7.11919250569764	6.41452281245340	1.80939123120227
H	8.72251220894026	5.62076833524192	1.67429237438995
C	4.29848341736566	7.40385643244033	2.66012545879403
H	5.28953912877511	7.31754739119477	3.13334156904998
H	3.83613589660544	8.34467898149096	3.01131170450900
H	4.42363844068184	7.47284494863234	1.56377244480868
C	10.23710134553017	5.02032835912078	8.39051543802384
H	9.25760753476762	5.03524714001779	7.88604783982871
H	10.17625550594189	5.68403118789947	9.27264661506658
H	10.44386699741968	3.99464061626414	8.74790820284932
C	6.84378198487373	2.29378339212273	7.93027618038508
H	7.43616309052001	2.82410635905840	8.69869215473799
H	6.89157116409709	1.21227554219409	8.14222406540053
C	7.36734697767428	2.64049462946521	6.54070676782404
H	8.45899763063427	2.78229207156358	6.49311227536042
H	7.06829584162454	1.89394375300810	5.77728544463482
C	12.67192293887508	5.56558173877766	8.24841605902196
H	12.95096136326120	4.57941595065595	8.66206633398527
H	12.54407595571956	6.25858533572242	9.09887976218750
H	13.51625348719163	5.93845908268225	7.64041970510641
C	2.89508158578354	0.57817818543680	0.74393292511325
H	3.87433586212598	0.11020545256091	0.94915848040287
H	2.32927670073134	-0.10183105886207	0.07994168811937
H	2.35323041817109	0.65390599269509	1.70350273694849
C	5.41063027189421	2.84559699812920	7.87516808090716
H	4.97316367810393	3.02540582683847	8.87170858295057
H	4.75007320999344	2.14201350977058	7.33521398331392
C	8.95405088579148	7.93482859784717	3.79363688746086
H	10.04511295957962	7.79861336691529	3.93334546210716
H	8.49338533206777	8.24715394006020	4.74541429215087
C	8.77103904493700	7.82697560844898	1.42709192053885
H	8.23190743647586	8.14277030096910	0.51791479719085
H	9.83668730228707	7.69718142843200	1.16282116028249
C	1.65124732747828	2.52809832030537	-0.20870848579447
H	1.69928559217526	3.51760686247481	-0.69763680435003
H	1.04409542347832	2.62693718137874	0.70880993143005
H	1.11528695862428	1.84380089765311	-0.89061889054061
C	8.63065983698677	8.81689733615119	2.59508935507036
H	9.30385461881241	9.68703928760558	2.51323665505349
H	7.59128999755591	9.18680997558112	2.67057104889267

9_s

V	7.63891514005946	5.21309167561456	4.82051282637724
Cl	6.83939185462997	7.06156689020761	6.14609471197810
O	8.33517804253266	6.59726376611751	3.39877146964091
O	6.84170630991579	3.90549817852216	6.25239005433687
O	9.30373727737341	5.04895063012537	5.51947533270068
N	9.40870185042173	3.06027607911082	3.59455647564382
O	6.04886912581628	5.08732583768753	3.86007423224160

N	9.49085043515203	2.08265187013259	2.64851112412931
N	8.27939370245441	2.09992898155855	2.08759168533232
C	6.04260926797453	3.31025977343168	2.25282657017275
C	11.71662963098144	2.59381403570330	4.22587477652539
H	11.72376565076599	1.81838358996367	3.45669791836957
C	7.42707896426856	3.03320097043164	2.62758632426670
C	10.52657008716795	3.33111212439473	4.39615597177697
C	8.16693609473992	3.70481291847127	3.64602892941813
C	5.39859373926916	4.40267354357181	2.93401182631691
C	4.05214555231722	4.75733907978513	2.62400343650053
C	12.83573233074858	2.85923989451468	5.01991670872899
C	11.57067279326846	4.64318539295091	6.20113353292764
C	5.29810172720896	2.58904121323526	1.29465655297269
H	5.75524726523393	1.74069866855866	0.78267398716872
C	10.43857087268630	4.36027320685302	5.37930858706123
C	12.73182904310536	3.88258701806944	5.99270235506457
H	13.60589207760709	4.09170842131769	6.61611261988614
C	3.37415735113467	5.93334884313293	3.35382938330612
C	3.96896779226414	2.91175807728415	0.97392031673062
C	3.22026102833905	2.07809776186688	-0.08242233876615
C	14.15698942515209	2.08554077260580	4.87085252347890
C	11.50483583034815	5.74608008969018	7.27611591793915
C	1.93230100058017	6.15284899594082	2.85670826130116
H	1.89643039593067	6.39175156098237	1.77805897814896
H	1.48565238038122	7.00435847720888	3.39993284959163
H	1.29069164475642	5.27122888550323	3.03803607948845
C	15.29014027422645	3.07585745939291	4.51335384115846
H	15.07448274020903	3.59033896678141	3.55985411567149
H	16.25307480062495	2.54224254385473	4.40804344708818
H	15.41645821173320	3.84945083354811	5.29068652701152
C	3.38056449589939	3.99842588834669	1.64841464606243
H	2.34912714139674	4.26639524400436	1.40939358944446
C	6.71126938414680	4.17671751602076	7.67918215465982
H	6.66157740416238	5.27318540604380	7.78700922450613
H	7.61644716779470	3.78852783627091	8.18612757882508
C	14.49064985893450	1.38226811003787	6.20738406836434
H	14.59245691601137	2.10561464299046	7.03507909316376
H	15.44298394011697	0.82591665210059	6.12600434362119
H	13.69537223015066	0.66675943323091	6.48296786307767
C	11.18756406206197	7.11061375586720	6.61762807749463
H	11.93476480032012	7.35393944800888	5.84024062261315
H	11.21278555137052	7.91145695195736	7.37939463549279
H	10.18520254244128	7.10877522074573	6.16283713792005
C	8.08278721621577	1.14825502604974	1.00621047517321
H	9.04954117499566	0.65256497078741	0.83836074856654
H	7.76813303712874	1.66820229546360	0.08653664057792
H	7.32557520967179	0.39386437304235	1.27875598077076
C	3.97057221450392	2.16516377073116	-1.43229725307930
H	4.02765265371880	3.21123587054975	-1.78254335408786
H	3.45230612453019	1.56943004865951	-2.20657676458029
H	5.00357495355642	1.78252798168409	-1.35091245665162
C	14.08061615233076	1.01650942697449	3.76608146924294
H	13.30029470742912	0.26405729485205	3.97909903041869
H	15.04635744982173	0.48520098822836	3.69057231911298
H	13.86655403503927	1.46392851541072	2.77893393379241
C	3.31502461699375	5.64663355823591	4.87592429666435

H	2.77993020874033	4.69894511458507	5.07256923587685
H	2.76896169968442	6.45902211756921	5.38982317070869
H	4.32414146319821	5.59404952469996	5.31483836601041
C	8.12724894986661	6.41462371618050	1.97164835286026
H	7.05106860449079	6.22181102585014	1.81141372175601
H	8.69712570682455	5.52562739988402	1.65164384879689
C	4.16728657717891	7.24139948319145	3.10476936395972
H	5.17820127583676	7.18656879522691	3.53887985955443
H	3.64462444086871	8.09262217927770	3.57846775941806
H	4.24497464547530	7.44922515482786	2.02139118325680
C	10.40147074954661	5.40729525353454	8.30730367352794
H	9.40847963383627	5.39553664227411	7.83266174756812
H	10.38639773920298	6.16845392856638	9.10901115686352
H	10.58844429695553	4.42216680617138	8.77229187132992
C	5.53944579962751	2.16554725719184	7.20732188724572
H	6.25410136354407	1.44105008448031	7.63984838963756
H	4.57109039955209	1.65306267663089	7.07780412762688
C	6.07016181499112	2.72810892730293	5.88821813418473
H	6.73274175040268	2.04285211301247	5.33279468742714
H	5.25518974622265	3.05381979279031	5.21689149901190
C	12.83882199126878	5.88347844830220	8.03462576830920
H	13.11323864890433	4.95325263596597	8.56430373806728
H	12.74524611408542	6.68168568968763	8.79224397088447
H	13.67145942445891	6.15917504136685	7.36233724129770
C	3.15919648247560	0.60218457416532	0.37645777152642
H	4.16817229345157	0.17286711472801	0.50941411447665
H	2.62726339054537	-0.01688581404045	-0.36972943817117
H	2.62779647533957	0.51279574648702	1.34072510498595
C	5.45505789590869	3.42149564531043	8.08932040386104
H	5.42951789893222	3.19749280178164	9.16926772717880
H	4.55756219793800	4.01606067311751	7.83619979489782
C	8.78567621157686	7.95561037068171	3.67570633972973
H	9.88602324469029	7.93454743521257	3.80206423675565
H	8.30482654251281	8.25898548729433	4.62064620026451
C	8.58489136433858	7.72149932557971	1.32126497774718
H	8.01685920515605	7.94648523899302	0.40263675303971
H	9.65707611719140	7.67164374255014	1.05566571299833
C	1.77905440809770	2.57431920709303	-0.29977250623127
H	1.75487369387884	3.61985695542916	-0.65558131930245
H	1.18054811148310	2.51224232799462	0.62665751044738
H	1.27912045792559	1.95066263396169	-1.06235706623676
C	8.36271871925117	8.74567998903001	2.44551633368521
H	8.94687280479169	9.67244740478743	2.31526312513743
H	7.29308568762565	9.01636407685428	2.52119045517567

9_t

V	7.49418379352451	5.31368049096934	4.81808490808056
Cl	6.86611591774995	7.12646348567669	6.18623484450566
O	8.44712293115285	6.64116600860764	3.47314423325273
O	6.60666391664989	3.95673462805983	6.16978498039713
O	9.17553891538972	4.97360495479526	5.63427436547653
N	9.31761190599147	3.16396814462300	3.49120205438352
O	5.98032008182590	5.28073052957290	3.68231342519265
N	9.41245727977028	2.23623341585342	2.52875916400037
N	8.18609666434258	2.20566005317473	1.99127647904207
C	5.91558754942798	3.33563968896265	2.28170757287223

C	11.67272658738095	2.80238457016848	4.02097494062728
H	11.70771786924318	2.13483481083404	3.15662319693523
C	7.30927150586540	3.08402080892662	2.61087008225625
C	10.45096546542338	3.44505805297181	4.30011587455999
C	8.06736079722815	3.73892877614363	3.60377956376755
C	5.31381457488418	4.52514453091350	2.83928694691834
C	3.98292177567042	4.88863575597618	2.44410745225736
C	12.78819619478967	3.01329948421303	4.83268329306320
C	11.43456283249592	4.52527763264157	6.27429830756112
C	5.13517270822402	2.49241002843560	1.45768822580864
H	5.56125460912504	1.55045940351746	1.10232896129253
C	10.30539822155550	4.34665504023812	5.39966652607557
C	12.62660365167843	3.86802708524147	5.95337044916769
H	13.48889953261226	4.01781328971474	6.61060550230048
C	3.35044864293252	6.20617161271035	2.93710005885410
C	3.81585788263234	2.80044441014049	1.10785792997598
C	3.00988025984679	1.82929557933423	0.22500011547741
C	14.15107987501230	2.35318934412283	4.56664273750153
C	11.29694997592500	5.39400906425407	7.53994136705709
C	1.93741072285029	6.41265144442196	2.35741186607443
H	1.94178212180537	6.44854281131540	1.25276785000109
H	1.53297145199300	7.37443977522906	2.71975153284165
H	1.23743183004476	5.61875038497722	2.67576330210337
C	15.21959117704089	3.45266019373682	4.36197117344127
H	14.96503107617718	4.09047247190887	3.49660951356821
H	16.21258538018090	3.00153079304809	4.17858977630525
H	15.30536998344781	4.10705723970304	5.24704619537567
C	3.28441287891473	4.01433405717230	1.59861691887745
H	2.26579643135921	4.28549065552240	1.31282193109386
C	5.41741185300381	4.21743233861099	6.96984784271495
H	4.58566843013011	4.43883928701558	6.28102487484376
H	5.62120349775888	5.11865880112016	7.57564938060787
C	14.54325595385805	1.47585162869772	5.77871660017271
H	14.61225155008103	2.06911518893529	6.70729755373729
H	15.52639722909638	0.99784595065896	5.61109703623862
H	13.79588906328412	0.67931176223429	5.94412557476076
C	10.92255064181722	6.84903499156951	7.16781333134554
H	11.66548752845640	7.27813071333571	6.47052905335265
H	10.90938433308716	7.47705736194474	8.07779487618659
H	9.92410105101412	6.90090544369405	6.70691211961027
C	8.00610547636081	1.33370188093969	0.84475458738102
H	9.00744795692266	1.03584686462411	0.50091093225780
H	7.47949024009942	1.87328124494058	0.04081137039608
H	7.43083607512115	0.42973683604378	1.11195712203238
C	3.74550697862770	1.61796468230418	-1.11903680634945
H	3.86190398861807	2.57545671521193	-1.65757694998999
H	3.18223310339244	0.92233042979711	-1.76873008608971
H	4.75367224317696	1.19166714236793	-0.96996590959955
C	14.12480026650319	1.46221218660256	3.31184759538541
H	13.39526819981310	0.63842274562648	3.41088265545683
H	15.11985287327366	1.00985932025644	3.15096752458344
H	13.86967229660702	2.04071685968039	2.40577816408470
C	3.23520866466556	6.21163661644037	4.48054363989778
H	2.67074859739216	5.32883853265996	4.83365344862593
H	2.69308795573586	7.11576237959376	4.81427890151575
H	4.22828109255076	6.22315612842535	4.95654398568371

C	8.17611211892735	6.69648953894602	2.05100687012626
H	7.12957131187359	7.02217429741319	1.90386410138981
H	8.28599204478691	5.67660386270722	1.64405810846057
C	4.22347695463855	7.40532054247692	2.49224698537457
H	5.22005419110718	7.35746662295349	2.95881991189591
H	3.74868262810167	8.35480462581558	2.80095803258934
H	4.33827213772416	7.42104439605487	1.39285683369624
C	10.19328429908461	4.80033408531910	8.45184785593074
H	9.21432935445470	4.81806605848082	7.94656174227914
H	10.11034244034705	5.39469993631755	9.38041538221526
H	10.43585917198322	3.75879612461783	8.73216102599509
C	6.64483675104399	2.39682990207118	7.92359342523766
H	7.21129950368649	2.94850078428605	8.69652479625303
H	6.68117460108089	1.32213127722286	8.16979089300699
C	7.21893155669964	2.70036521070063	6.54408411690051
H	8.31171722093923	2.83758219482779	6.52835713753096
H	6.94045458920121	1.93308014297380	5.79338918012622
C	12.60794640291079	5.43361583507369	8.34819230815084
H	12.92145175785803	4.42795216879053	8.68261174424034
H	12.46103338101434	6.05305963662335	9.25075864157357
H	13.43694959138634	5.88136813362627	7.77029943074865
C	2.87292637180211	0.47106783033345	0.95218621099743
H	3.85960667486992	0.02256598266169	1.16523659353218
H	2.30185471978292	-0.24812440684649	0.33561724938070
H	2.34684922226733	0.59387672098704	1.91569021579623
C	5.21582689971213	2.94898803179696	7.80029449545841
H	4.74475034942989	3.16302379282383	8.77444983651945
H	4.57251522832432	2.22813359591602	7.26236262726936
C	9.30975571801292	7.73613125955772	3.88698236588118
H	10.30954934670891	7.31933598185734	4.11473951664153
H	8.87223855421804	8.16279413716946	4.80527708967758
C	9.18224721921614	7.70772740210063	1.50609913813030
H	8.82826206227949	8.19167452229877	0.58008102381188
H	10.14839051722329	7.21516261978319	1.28926721409600
C	1.59625592575600	2.35806096270146	-0.07993327271190
H	1.62685828767509	3.32102787319586	-0.62057506918139
H	1.00436595346389	2.50021622436363	0.84187795722208
H	1.05447600151798	1.63487183307336	-0.71562105130045
C	9.33000244947452	8.67979117479753	2.68777169479459
H	10.25124499247674	9.28506441842438	2.64524105632872
H	8.46673238432381	9.36937212454397	2.72915954674709

6. EPR Spectroscopy

Simulated EPR-Spectra for complexes **[Co(Cp*)$_2$][1]** and **[Co(Cp*)$_2$][2]** were obtained using the garlic function (non linear least squares fitting) of the EasySpin package in MATLAB®.[6] The lowest RMSD values were found at g_{iso} = 1.9715, a = [274.1915 MHz, 268.8744 MHz, 258.604 MHz], Lorentzian linewidth = 1.344 MHz and 28.9 ns correlation time for **[Co(Cp*)$_2$][1]** and at g_{iso} = 1.9666, a = [256.8 MHz 267.7 MHz, 261.9 MHz], Lorentzian linewidth = 1.616 MHz and 31.0 ns correlation time for **[Co(Cp*)$_2$][2]**.

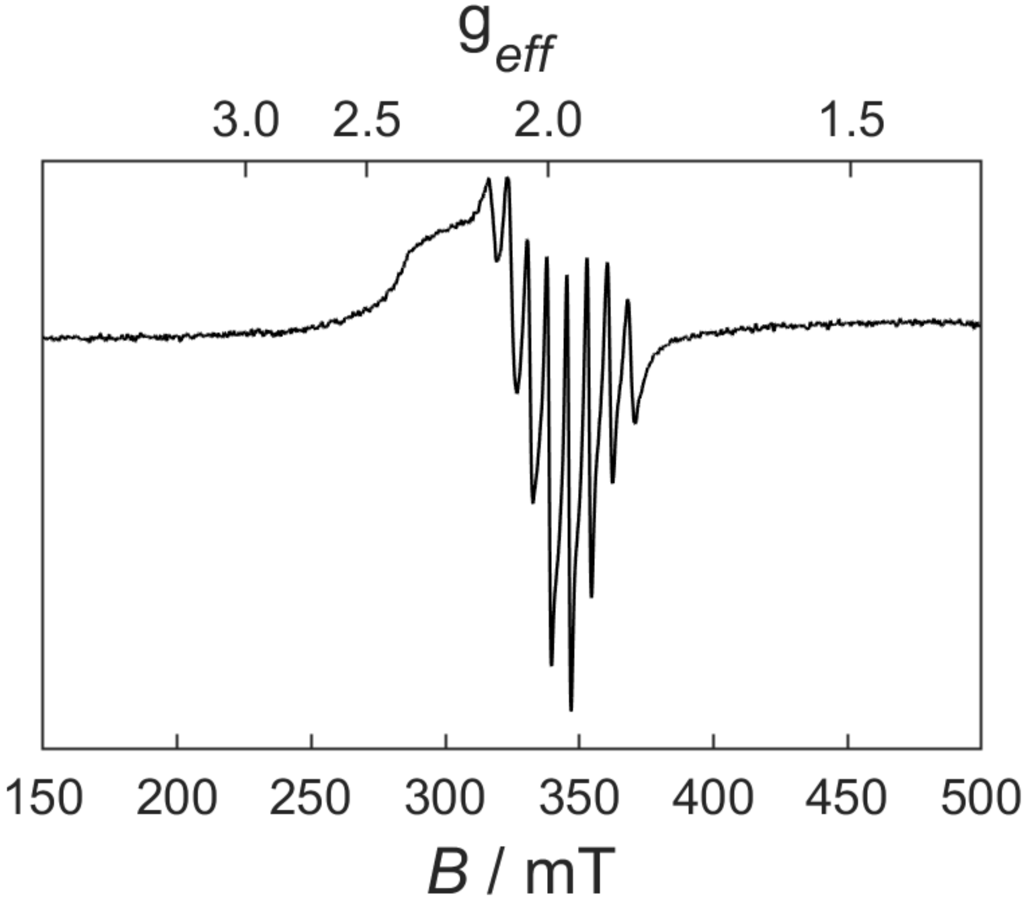

Figure S 90: X-band EPR spectrum of a 5mM solution of **6** in toluene at 300 K. The eight-line signal observed at g_{iso} = 1.9681 results most likely from a V(IV) impurity.

7. Crystallographic details

Table S 2: Crystallographic details

	**1***	**2**	**[Co(Cp*)$_2$][1]****	**[Co(Cp*)$_2$][2]****	**3****	**5**	**6*****	**8**	**10****
Chemical formula	$C_{31}H_{43}N_3O_3Cl_1V_1$<br>$C_7H_8$	$C_{35}H_{44}N_2O_3Cl_1V_1$	$C_{20}H_{30}Co_1$<br>$C_{31}H_{43}N_3O_3Cl_1V_1$	$C_{20}H_{30}Co_1$<br>$C_{35}H_{44}N_2O_2Cl_1V_1$	$C_{40}H_{54}N_3O_4V_1$	$C_{45}H_{57}N_4O_2V_1$	$2(C_{39}H_{59}N_3O_4Cl_1V_1)$	$C_{37}H_{47}N_4O_2F_1Cl_1V_1$<br>$C_4H_{10}O_1$	$2(C_{34}H_{52}N_4O_2Si_1Cl_1V_1)$
M_r	684.20	627.11	921.44	956.48	691.80	752.88	1440.56	759.29	1326.55
Crystal system	Monoclinic	Orthorhombic	Monoclinic	Orthorhombic	Monoclinic	Triclinic	Triclinic	Monoclinic	Monoclinic,
Space group	*P*2$_1$/*n*	Pbca	*P*2$_1$/*n*	*Pbca*	*P*2$_1$/*c*	*P*-1	P-1	*P*2$_1$/*n*	*P*2$_1$/*n*
a (Å)	10.707(2)	13.701(1)	12.5450(5)	19.5719(6)	29.460(4)	13.1076(8)	15.404(3)	16.8943(10)	18.8519(15)
b (Å)	22.859(4)	16.588(1)	19.1392(8)	23.6798(8)	14.896(2)	13.5852(9)	16.355(3)	14.5056(9)	10.7090(10)
c (Å)	15.010(3)	29.021(3)	22.4206(9)	24.1624(7)	20.399(2)	14.0621(9)	17.174(3)	17.7402(12)	40.126(3)
α (°)	90	90	90	90	90	118.469(3)	90.146(7)	90	90
β (°)	92.85(1)	90	103.168(2)	90	96.179(4)	94.955(3)	96.074(8)	107.698(2)	99.247(2)
γ (°)	90	90	90	90	90	104.110()	97.907(8)	90	90
V (Å^3)	3669(1)	6595.8(9)	5241.7(4)	11198.3(6)	8900(2)	2073.7(2)	4261.0(14)	4141.7(5)	7995.5(11)
Z	4	8	4	8	8	2	2	4	4
Densitiy (g cm^{-3})	1.239	1.263	1.168	1.135	1.033	1.206	1.123	1.218	1.102
F(000)	1456	2656	1964	4072	2960	804	1544	1616	2832
Radiation Type	MoKα	MoKα	MoKα	MoKα	MoKα	MoKα	MoKα	MoKα	MoKα
μ (mm^{-1})	0.382	0.418	0.586	0.550	0.259	0.282	0.333	0.349	0.376
Crystal size	0.09x0.08x0.06	0.04x0.03x0.004	0.15x0.13x0.05	0.20x0.15x0.01	0.11x0.10x0.02	0.08x0.07x0.06	0.25x0.15x0.03	0.45x0.10x0.07	0.45x0.25x0.10
Meas. Refl.	101324	27019	82542	109455	71804	142685		56058	78415
Indep. Refl.	6470	5999	9202	9863	7840	9522		7658	14841
Obsvd. [$I > 2\sigma(I)$]	5363	4402	8231	6483	4512	7698		303	9958
R_{int}	0.0909	0.1015	0.0424	0.1203	0.1753	0.1031		0.1031	0.0957
R [$F^2 > 2\sigma(F^2)$]	0.0632	0.0613	0.0426	0.1028	0.0725	0.0443		0.0452	0.0741
wR(F^2)	0.1774	0.1327	0.1275	0.3127	0.1673	0.1153		0.1026	0.1769
S	1.092	1.073	1.027	1.18	1.016	1.055		1.019	1.026
$\Delta\rho_{max}$	2.453	0.385	1.484	0.7446	0.329	0.820		0.281	0.751
$\Delta\rho_{min}$	-0.844	-0.461	-0.720	-0.452	-0.428	-0.322		-0.345	-0.743
CCDC	2079467	2079465	2079472	2079471	2079474	2079469	2096422	2079473	2080686

* Due to moderate quality of the crystals a minor positive electron density remains close to the vanadium center and cannot be refined. This most likely resulted as an artefact from the measurement and is typical for heavy atom containing structures. ** Due to heavily disordered solvent molecules, the SQUEEZE algorithm was applied. For the cationic complexes **[Co(Cp*)$_2$][1]** and **[Co(Cp*)$_2$][2]** disordered dichloromethane was squeezed, for the neutral complexes **3** and **10** heavily disordered diethyl ether was removed from the lattice. ***Due to the high sensitivity of this compound (less than 30 seconds in air) and the propensity of the compound to only form small and disordered crystals, these values are only given for orientation.

Table S 3: Selected Bond lengths and angles

	**1**	**2**	**[Co(Cp*)$_2$][1]**	**[Co(Cp*)$_2$][2]**	**3**	**5**	**8**	**10**
V1 – C1	2.055(3)	2.131(3)	2.070(3)	2.153(7)	2.067(3)	2.0830(18)	2.048(2)	2.048(4)
V1 – O1	1.823(2)	1.830(2)	1.935(2)	1.916(6)	1.853(2)	1.9037(13)	1.835(2)	1.845(3)
V1 – O2	1.843(2)	1.827(2)	1.945(2)	1.901(6)	1.901(3)	1.9050(13)	1.858(2)	1.864(3)
V1 – O10/N40	1.583(3)	1.585(2)	1.603(2)	1.618(6)	1.589(3)	1.5865(13)	1.644(2)	1.621(4)
V1 – X*	2.273(1)	2.275(1)	2.366(1)	2.331(3)	1.811(2)	1.9161(15)	2.3045(7)	2.3184(12)
N1 – C8	-	1.426(1)	-	1.400(10)	-	-	-	-
C8 – C13	-	1.399(5)	-	1.410(11)	-	-	-	-
C13 – O1	-	1.351(4)	-	1.294(10)	-	-	-	-
C2 – C5	1.446(5)	-	1.438(4)	-	1.454(5)	1.449(2)	1.453(3)	1.460(5)
C5 – C10	1.405(5)	-	1.415(4)	-	1.418(5)	1.413(3)	1.412(3)	1.409(5)
C10 – O1	1.347(4)	-	1.322(3)	-	1.340(4)	1.337(2)	1.343(3)	1.337(4)
N1 – C11	1.435(4)	-	1.427(3)	-	1.430(5)	1.425(2)	1.433(3)	1.426(5)
C11 – C16	1.389(5)	-	1.402(3)	-	1.400(5)	1.402(2)	1.392(3)	1.399(5)
C16 – O2	1.345(4)	-	1.322(3)	-	1.341(4)	1.330(2)	1.342(3)	1.345(5)
O1 – V1 – O2	146.4(1)	142.5(1)	145.2(1)	146.1(3)	146.81(12)	148.01(6)	145.98(8)	144.69(12)
C1 – V1 – X*	149.6(1)	154.2(9)	143.4(1)	139.1(2)	151.07(15)	147.31(8)	152.21(7)	150.57(1)
O10/N40 – V1 – C1	99.6(2)	98.1(1)	107.6(1)	109.1(3)	100.05(13)	104.23(7)	94.81(9)	94.86(17)
O10/N40 – V1 – X*	110.8(1)	107.7(1)	109.1(8)	111.8(2)	108.68(13)	108.45(7)	112.94(7)	114.5(14)
τ_5	0.05	0.20	0.03	0.12	0.07	0.01	0.10	0.10

*X = Cl1, O40, N10

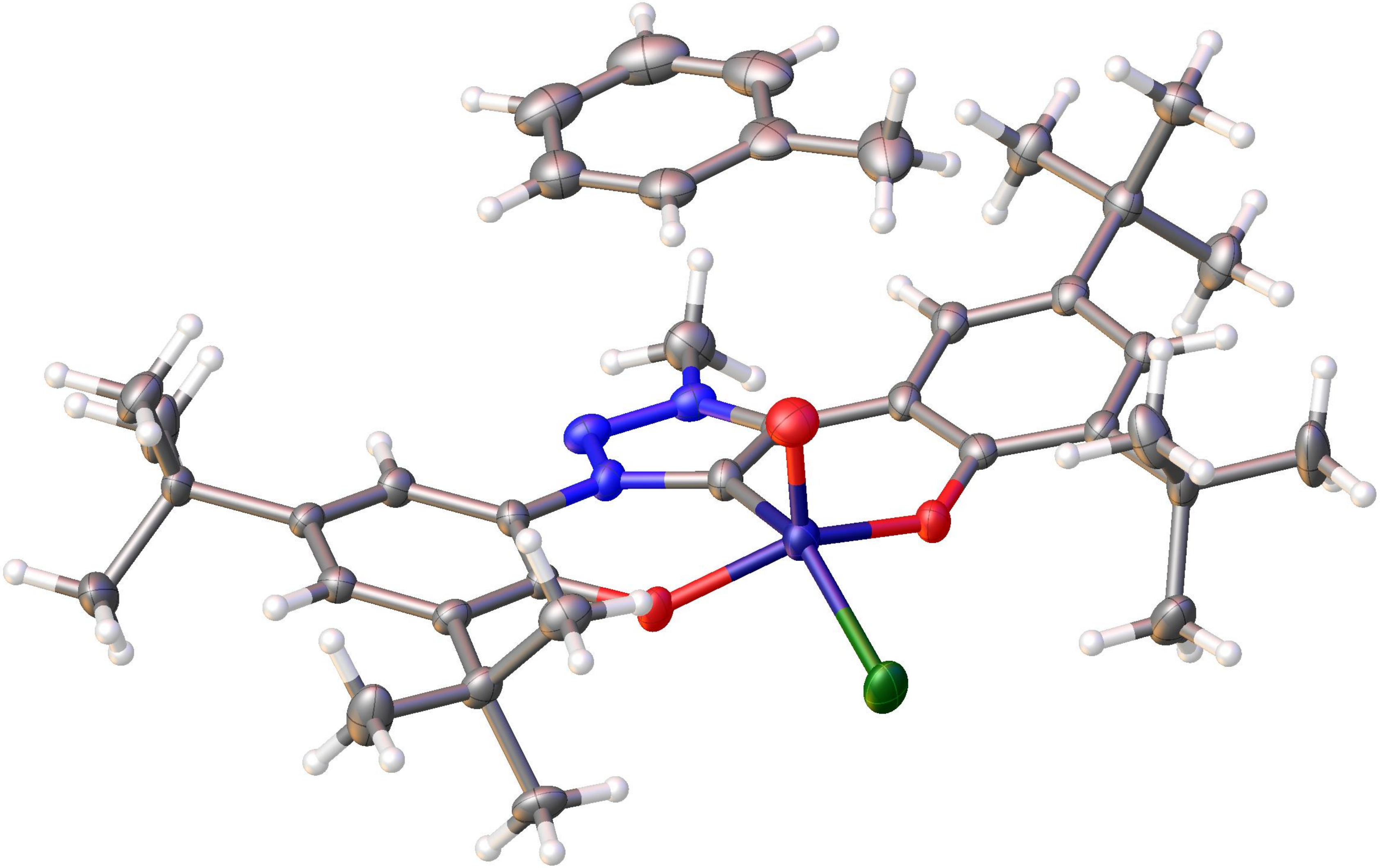

Figure S 91: Ellipsoid plot of **1** with all solvents in the asymmetric unit. Ellipsoids are shown at a probability level of 50 %.

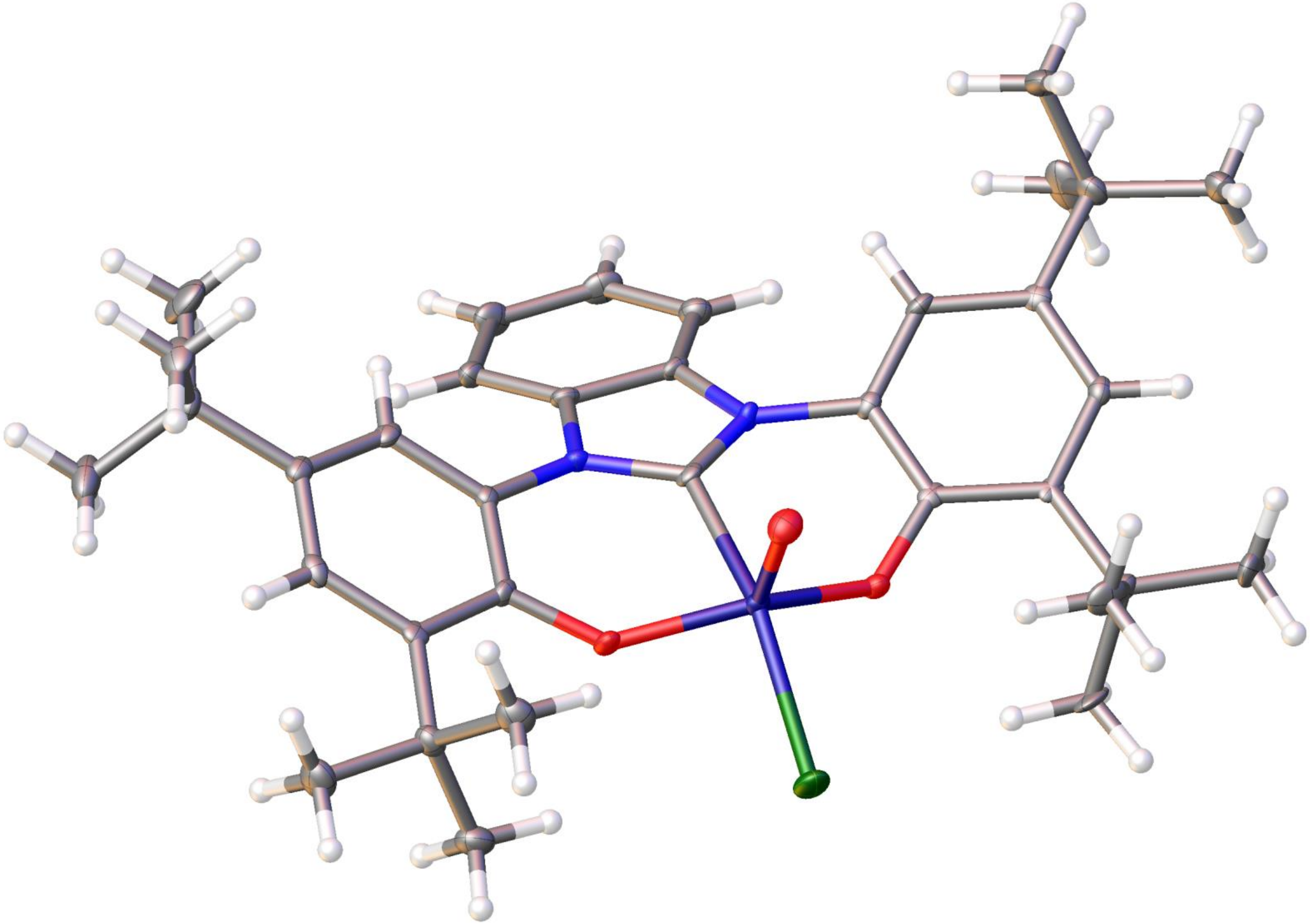

Figure S 92: Ellipsoid plot of **2**. Ellipsoids are shown at a probability level of 50 %.

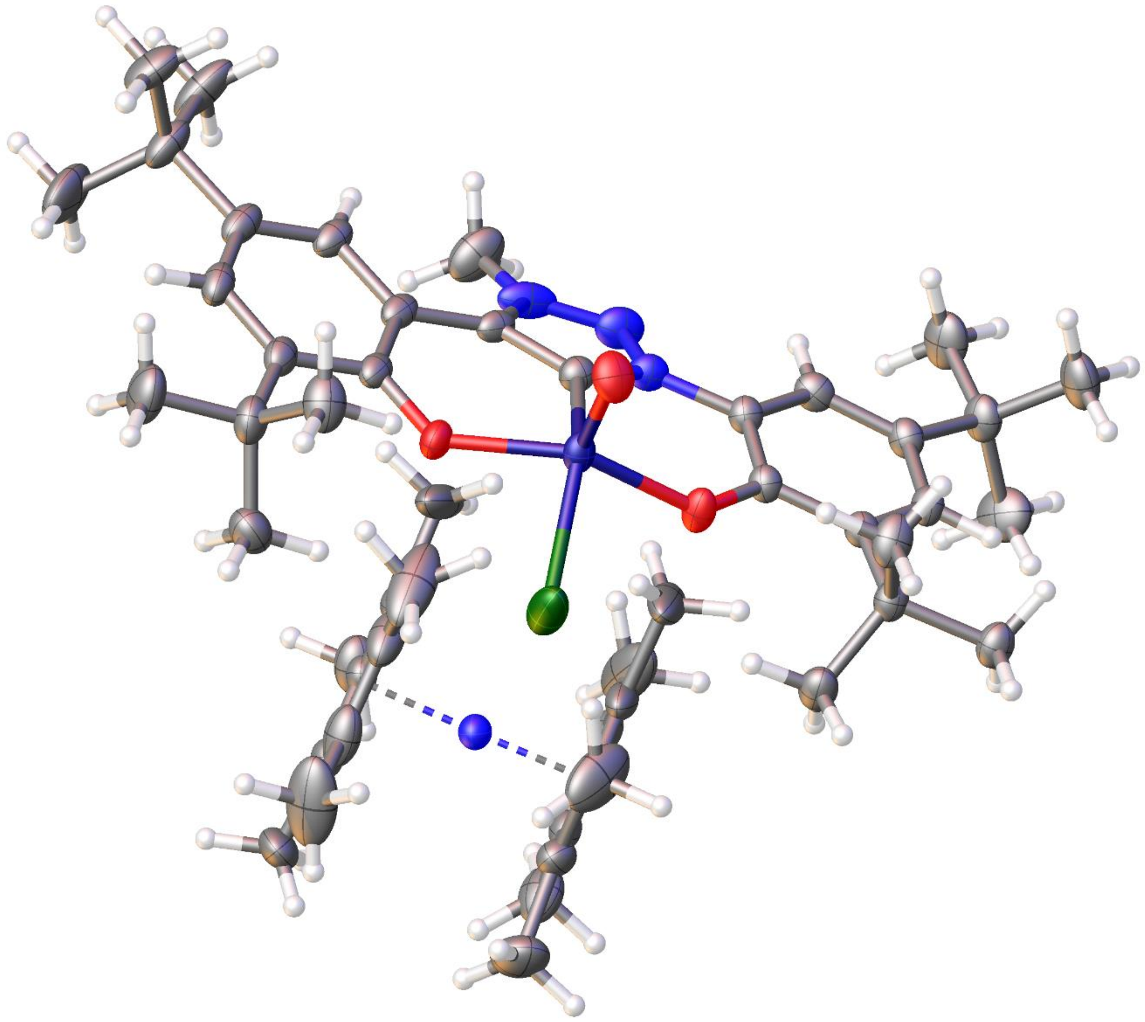

Figure S 93: Ellipsoid plot of **[Co(Cp*)$_2$][1]** including the decamethyl cobaltocenium counterion. Ellipsoids are shown at a probability level of 50 %. A heavily disordered molecule of dichloromethane could not be sufficiently modeled and was squeezed from the lattice.

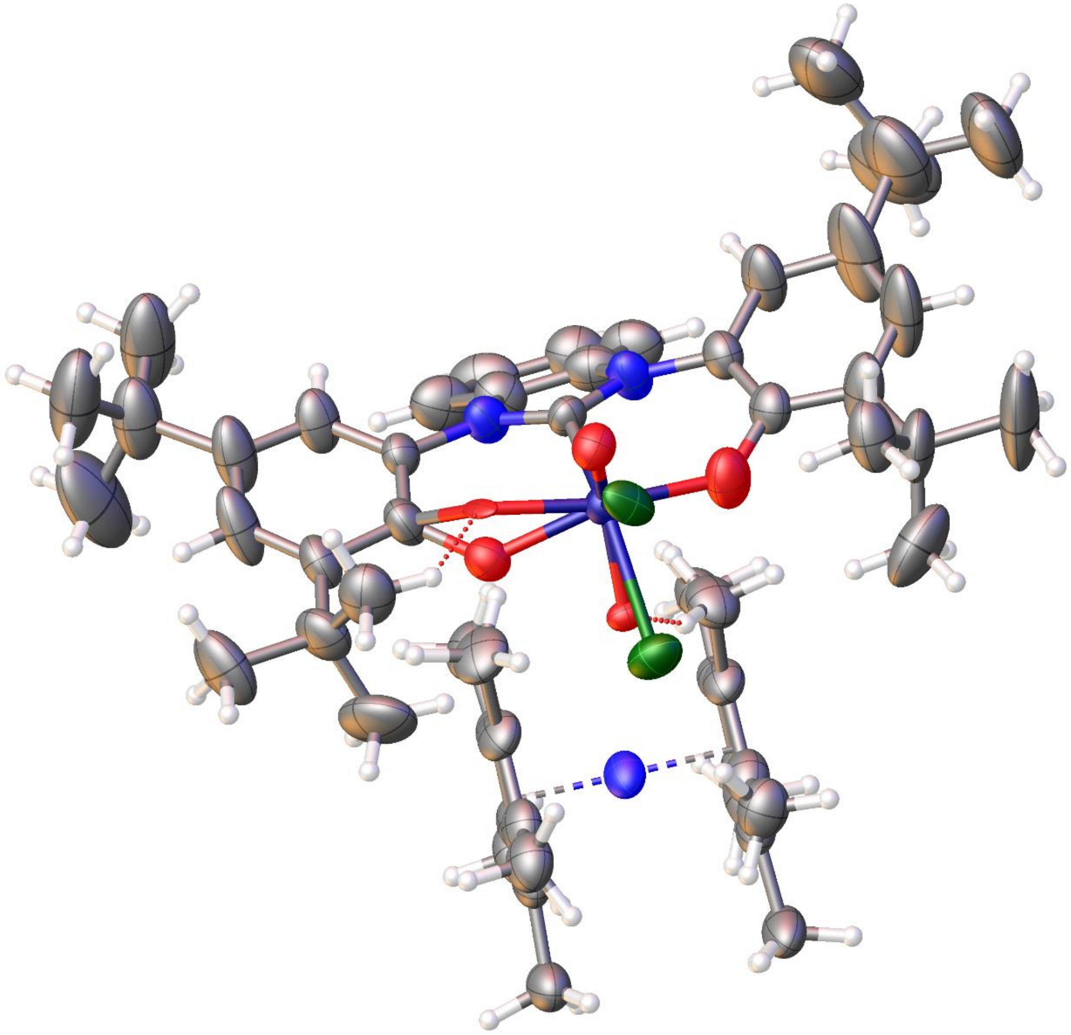

Figure S 94: Ellipsoid plot of **[Co(Cp*)$_2$][2]** including the decamethyl cobaltocenium counterion. Ellipsoids are shown at a probability level of 50 %. The full molecule disorder (2nd part is less than 10%) could not be completely resolved and was only refined for the central vanadium atom, the vanadium oxygen and one phenolate oxygen atom. A heavily disordered molecule of dichloromethane could not be sufficiently modeled and was squeezed from the lattice.

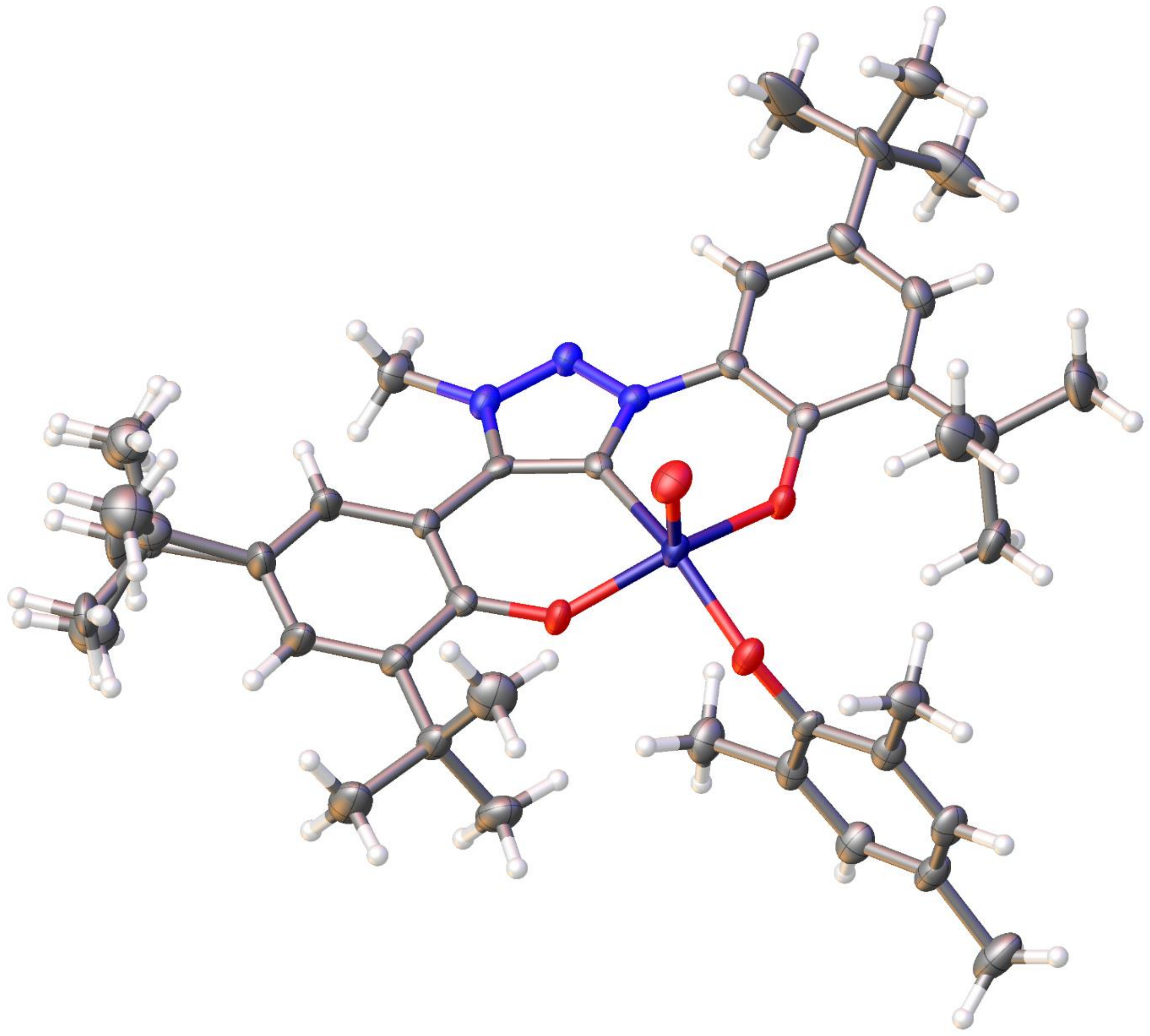

Figure S 95: Ellipsoid plot of **3**. Ellipsoids are shown at a probability level of 50 %. One ^{t}Bu group showed a rotational disorder. A heavily disordered molecule of diethyl ether could not be sufficiently modeled and was squeezed from the lattice.

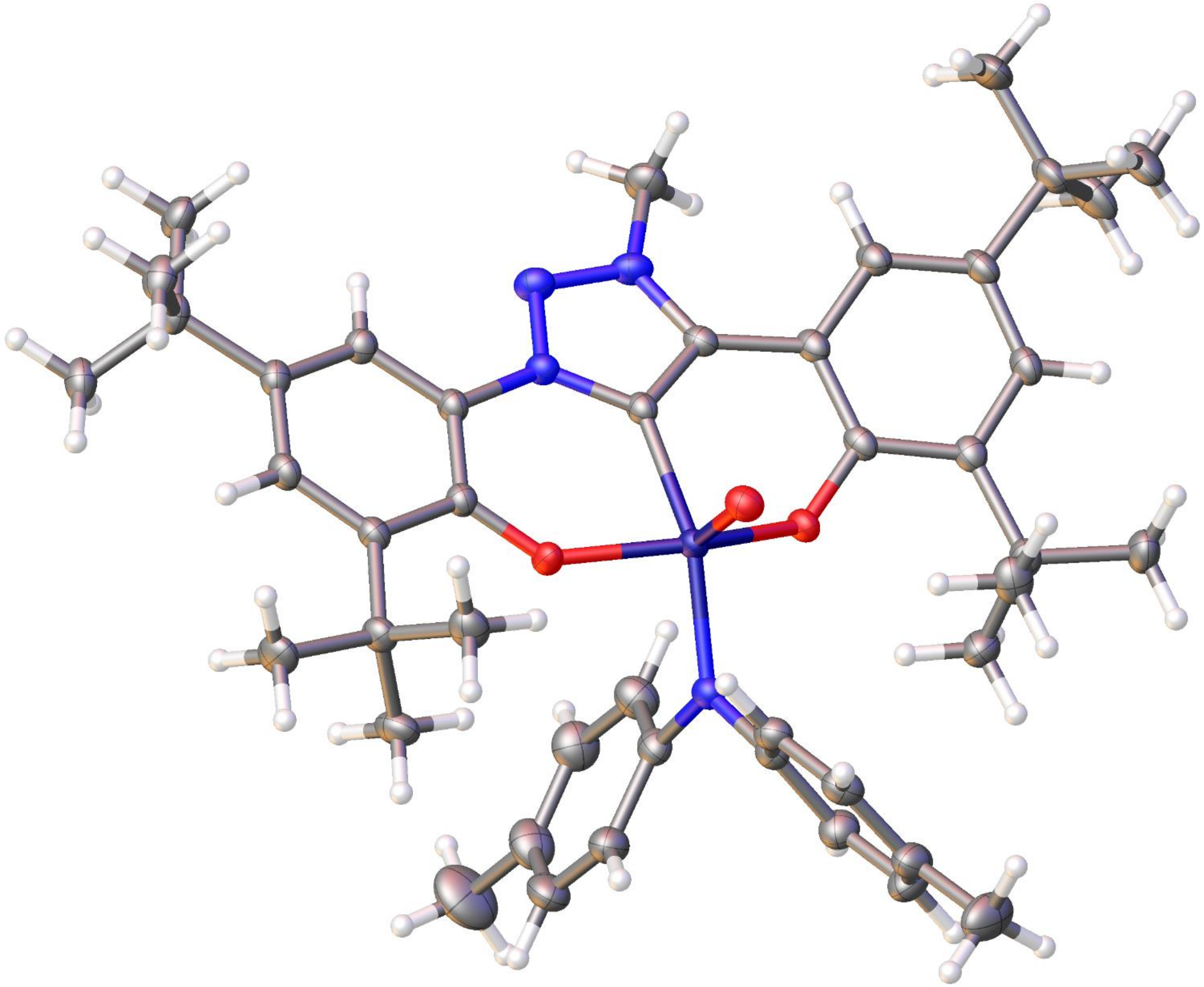

Figure S 96: Ellipsoid plot of **5**. Ellipsoids are shown at a probability level of 50 %.

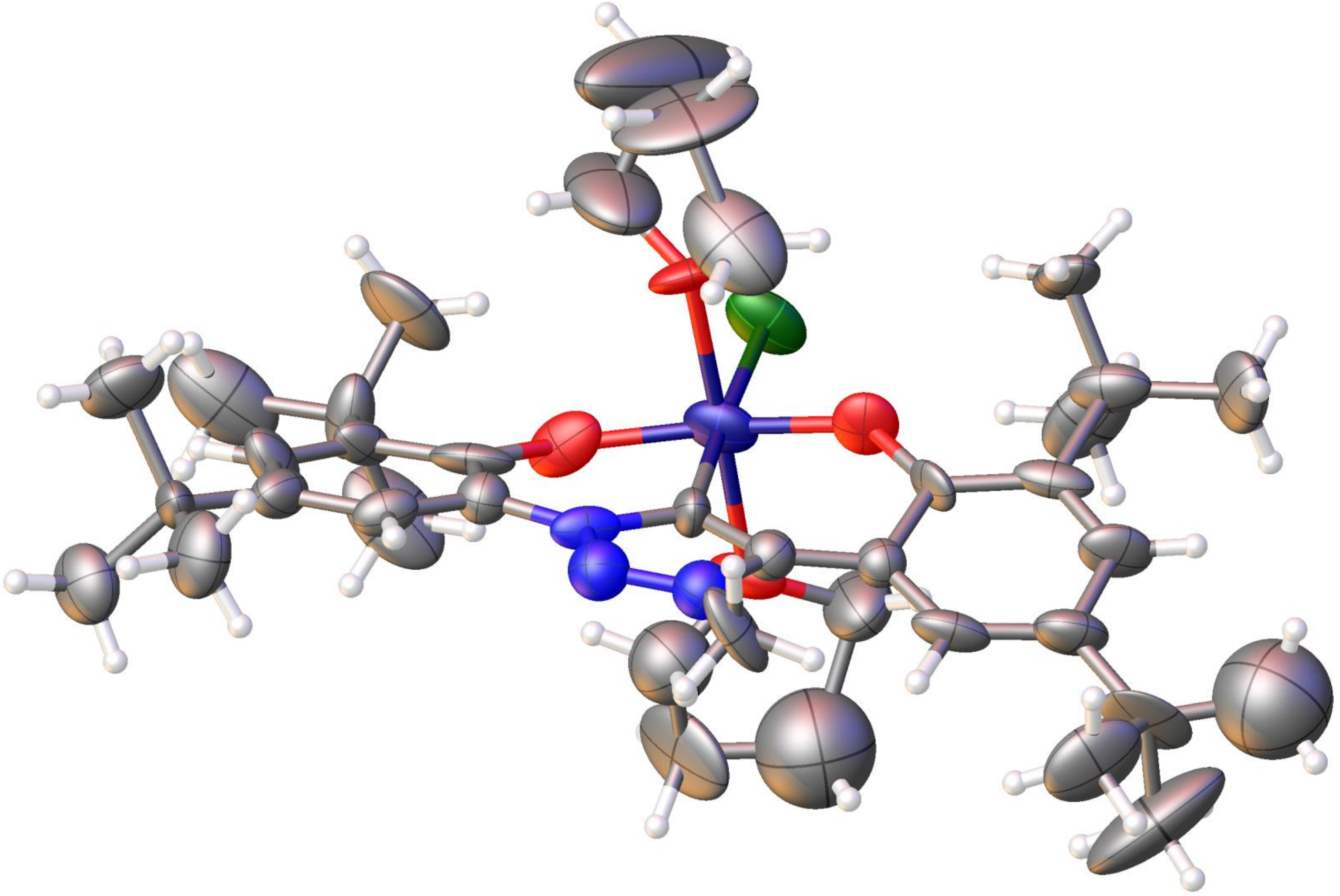

Figure S 97: Ellipsoid plot of **6**. Ellipsoids are shown at a probability level of 50 %. Due to the very low crystal quality, it was only possible to get sufficient data up to 35° in 2θ. The model as such cannot be considered reliable however it matches well with other analytical means suggesting that at least the connectivity and overall conformation of the molecule are correctly described.

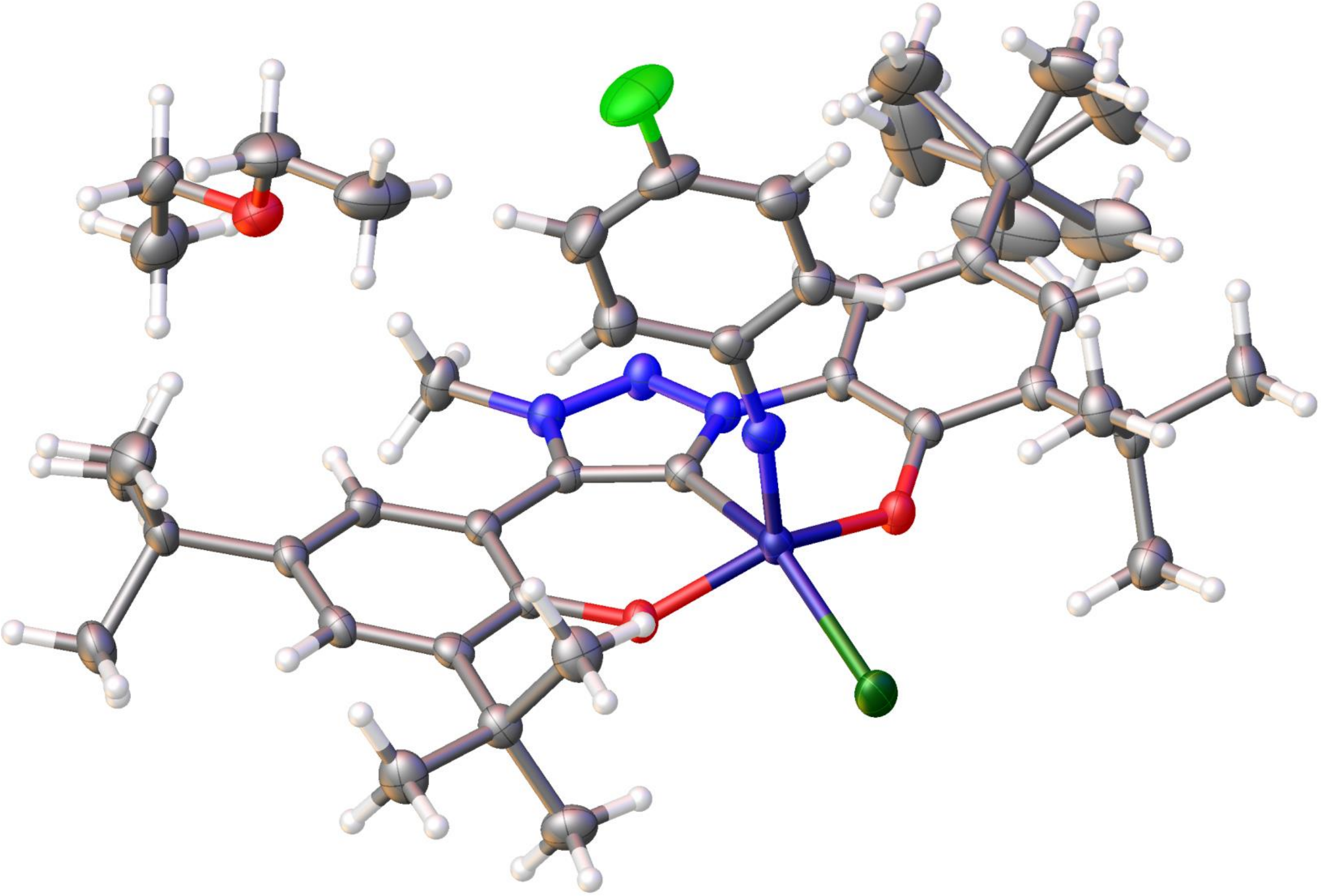

Figure S 98: Ellipsoid plot of **8**. Ellipsoids are shown at a probability level of 50 %. One of the ^{t}Bu groups showed a rotational disorder.

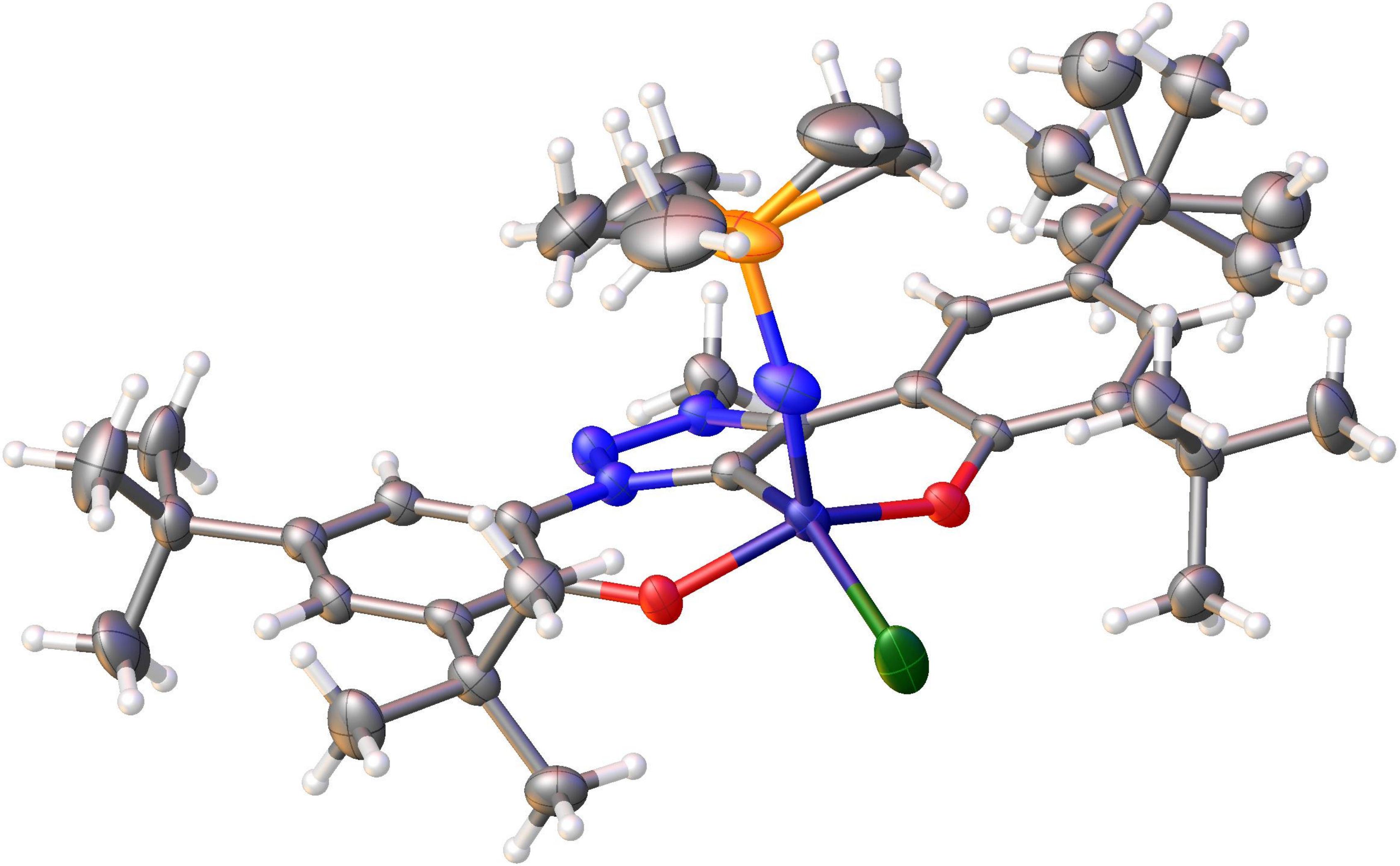

Figure S 99: Ellipsoid plot of **10**. Ellipsoids are shown at a probability level of 50 %. The TMS group and one of the ^{t}Bu groups showed a rotational disorder. A molecule of heavily disordered diethyl ether could not be sufficiently modeled and was therefore squeezed from the lattice.

8. Literature

(1) a) F. Neese, *Wiley Interdiscip. Rev.: Comput. Mol. Sci.* **2018**, *8*, e1327; b) F. Neese, *Wiley Interdiscip. Rev.: Comput. Mol. Sci.* **2012**, *2*, 73-78.

(2) a) S. Grimme, S. Ehrlich, L. Goerigk, *J. Comput. Chem.* **2011**, *32*, 1456-1465; b) S. Grimme, J. Antony, S. Ehrlich, H. Krieg, *J. Chem. Phys.* **2010**, *132*, 154104; d) J. P. Perdew, *Phys. Rev. B* **1986**, *33*, 8822-8824; e) F. Weigend, R. Ahlrichs, *Phys. Chem. Chem. Phys.* **2005**, *7*, 3297-3305; g) J. P. Perdew, K. Burke, M. Ernzerhof, *Phys. Rev. Lett.* **1996**, *77*, 3865-3868.

(3) D. A. Pantazis, X.-Y. Chen, C. R. Landis, F. Neese, *J. Chem. Theor. Comput.* **2008**, *4*, 908-919.

(4) a) E. v. Lenthe, E. J. Baerends, J. G. Snijders, *J. Chem. Phys.* **1993**, *99*, 4597-4610; b) C. van Wüllen, *J. Chem. Phys.* **1998**, *109*, 392-399.

(5) F. Weigend, *Phys. Chem. Chem. Phys.* **2006**, *8*, 1057-1065.

(6) S. Stoll, A. Schweiger, *J. Magn. Reson.*, **2006**, *178*, 42-55.
